# Transcriptomic analysis of a *Clostridium thermocellum* strain engineered to utilize xylose: responses to xylose versus cellobiose feeding

**DOI:** 10.1038/s41598-020-71428-6

**Published:** 2020-09-03

**Authors:** Albert E. Tafur Rangel, Trevor Croft, Andrés Fernando González Barrios, Luis H. Reyes, Pin-Ching Maness, Katherine J. Chou

**Affiliations:** 1 Grupo de Diseño de Productos Y Procesos (GDPP), Department of Chemical and Food Engineering, Universidad de los Andes, Bogotá, Colombia; 2 Grupo de Investigación CINBIOS, Department of Microbiology, Universidad Popular del Cesar, Valledupar-Cesar, Colombia; 3 Biosciences Center, National Renewable Energy Laboratory, Golden, CO USA

**Keywords:** Biotechnology, Computational biology and bioinformatics, Microbiology, Molecular biology, Systems biology

## Abstract

*Clostridium* (*Ruminiclostridium*) *thermocellum* is recognized for its ability to ferment cellulosic biomass directly, but it cannot naturally grow on xylose. Recently, *C. thermocellum* (KJC335) was engineered to utilize xylose through expressing a heterologous xylose catabolizing pathway. Here, we compared KJC335′s transcriptomic responses to xylose versus cellobiose as the primary carbon source and assessed how the bacteria adapted to utilize xylose. Our analyses revealed 417 differentially expressed genes (DEGs) with log_2_ fold change (FC) >|1| and 106 highly DEGs (log_2_ FC >|2|). Among the DEGs, two putative sugar transporters, *cbpC* and *cbpD*, were up-regulated, suggesting their contribution to xylose transport and assimilation. Moreover, the up-regulation of specific transketolase genes (*tktAB*) suggests the importance of this enzyme for xylose metabolism. Results also showed remarkable up-regulation of chemotaxis and motility associated genes responding to xylose feeding, as well as widely varying gene expression in those encoding cellulosomal enzymes. For the down-regulated genes, several were categorized in gene ontology terms oxidation–reduction processes, ATP binding and ATPase activity, and integral components of the membrane. This study informs potentially critical, enabling mechanisms to realize the conceptually attractive Next-Generation Consolidated BioProcessing approach where a single species is sufficient for the co-fermentation of cellulose and hemicellulose.

## Introduction

*Clostridium (Ruminiclostridium) thermocellum* is a thermophilic and anaerobic bacterium recognized for its superior, natural ability to directly ferment cellulosic biomass and grow on crystalline cellulose^1–4^. This gram-positive bacterium secretes cellulase and other enzymes that are either integrated into the multi-subunit, extracellular enzyme complexes called cellulosomes or free cellulase. These enzymes collectively eliminate the need for exogenously supplied and separately produced hydrolytic enzyme cocktails during the biochemical conversion of plant biomass to the desired product. While the bacterium expresses certain xylanases to break down hemicellulose^5,6^, it cannot grow on the soluble pentose sugars including xylose released from hemicellulose^7,8^. Although the bacterium has been considered a promising candidate for consolidated bioprocessing (CBP), which combines enzyme production, cellulose hydrolysis, and fermentation in one integrated step, CBP pertaining to *C. thermocellum* by convention is limited to utilizing only the cellulosic component of the plant biomass^2,9^. An even more practical and economical alternative, next-generation CBP (NG-CBP), was therefore proposed to directly ferment both cellulose and hemicellulose in the whole plant biomass using a single species, *C. thermocellum*^10^. Following along this concept, the bacterium has been engineered to ferment xylose via the integration of the xylose isomerase and xylulose kinase (encoded by *xylA* and *xylB* genes, respectively), and has demonstrated simultaneous co-fermentation of xylose with 6-carbon soluble (i.e., glucose, cellobiose) and insoluble substrates (i.e., Avicel or microcrystalline cellulose)^10^. The co-fermentation of both cellulose- and hemicellulose-derived substrates resulted in ethanol, acetic acid, and hydrogen (H_2_) as the dominant fermentation products and with lactic acid and formic acid reported in lower amounts^10^.

The transcriptome in a cell changes dynamically and actively depending on many factors including, but not limited to, the stage of development, environmental conditions^11^, and the presence of particular carbon sources^12–15^. Several studies have shown the transcriptional behavior of *C. thermocellum* in response to cellulose utilization^16,17^, furfural^18^, heat^18^, and ethanol stress^11^. In *C. thermocellum*, cell surface-bound cellulosomes, as well as the cell-free, long-range cellulosomes, mediate the deconstruction of cellulosic compounds^19,20^. The selective expression of the cellulosomal proteins is influenced by substrate availability^21–24^ and growth rates^25^. Similarly, as a result of carbon source availability, other genes differentially expressed in *C. thermocellum* are likely involved in the catabolism of the carbohydrates via glycolysis and pyruvate fermentation, ATP generation^17^, transcriptional regulation, initiations, sigma factors, signal transducers^20^, and more.

However, although previous studies in *C. thermocellum* have reported changes in gene expression as a response to carbon sources (e.g., pretreated switchgrass^21,22^, cellobiose^20,26^, cellulose^16,20^, pectin or xylan^27^), the transcriptional behavior in a *C. thermocellum* strain engineered to consume xylose has not been fully characterized. We hypothesize that since *C. thermocellum* does not naturally metabolize xylose, the introduced xylose-catabolizing pathway must induce cellular changes that help the bacteria facilitate the sensing, uptake/transport, and utilization of a substrate non-native to the cells. In this study, we set forth to compare differential gene expression by transcriptomic analyses via RNA-seq of an engineered, xylose-catabolizing strain (KJC335) grown in xylose (5 g/L) versus cellobiose (5 g/L) as the primary carbon source. As continued strain development for industrial applications requires an in-depth understanding of *C. thermocellum* physiology at utilizing five-carbon substrates, analyses performed herein provide insights into how the strain adapts to available xylose and how the adaptation contributes to xylose utilization. The knowledge of how the bacteria reallocate their cellular resources to grow on xylose is essential for rational re-engineering of the bacterium to efficiently co-utilize five-carbon (C5) and six-carbon (C6) simple and complex substrates. In this study, we examine the differentially expressed genes (DEGs) involved in cellulosomal components, xylose catabolic pathways, redox-mediating enzymes, sugar transporters, non-native substrate-sensing mechanisms, and chemotaxis. To our knowledge, this is the first study examining the transcriptional response in an engineered *C. thermocellum* strain capable of fermenting xylose as the primary carbon source.

## Results and discussion

### RNA-seq analyses and assembly

To determine the system-wide changes in gene expression in *C. thermocellum* cultures grown in xylose versus cellobiose, RNA-seq was performed on the KJC335 strain^10^ cultured and fed with the respective sugar as the primary carbon source in defined medium and harvested at mid-exponential phase. Our RNA-seq data were obtained from three biological replicates grown in either cellobiose or xylose. The *xylA* and *xylB* genes from *Thermoanaerobacterium ethanolicus* were previously integrated into the Δ*hpt* strain genome^10^. RNA-Seq analyses of 20 to 30 million paired-end sequencing reads per sample were obtained and aligned, resulting in in-depth coverage of the *C. thermocellum* transcriptome. Approximately 98% of the reads were mapped for KJC335 grown in cellobiose, and 97% of the reads were mapped for the strain grown in xylose using a publicly available genome as the reference (GenBank: NC_017304.1).

**Figure 1:**
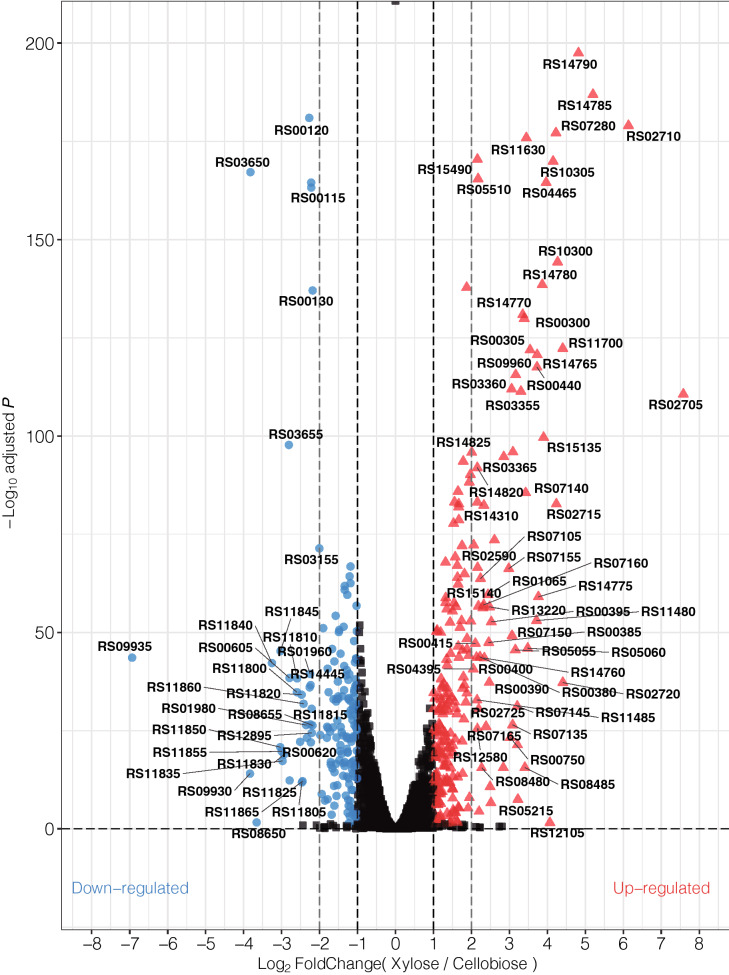
A volcano plot representing DEGs in the engineered *C. thermocellum* strain (KJC335) capable of growing on xylose as the main carbon source. DEGs were obtained from comparisons made between cultures grown in D-xylose vs. D-cellobiose. Down-regulated genes (log_2_ FC < − 1 and adjusted *p* value < 0.05) are shown in blue, and up-regulated genes (log_2_ FC > 1 and adjusted *p* value < 0.05) are shown in red. RS numbers in bold correspond to the numerical suffix of locus tags in *C. thermocellum* DSM 1313 (e.g., RS10310 = CLO1313_RS10310). The figure was created using the R package ggplot2^30^.

The degree of differentiation between the two conditions was represented using the absolute value of log_2_ fold change (FC). Log_2_ FC ≥|1| considering both up- and down-regulation was noted, and the absolute value of log_2_ FC ≥|2| was chosen to be the threshold for highly differentially expressed genes^17,28,29^. Based on the data, 417 DEGs with log_2_ FC >|1| and adjusted *p* value < 0.05 were identified when comparing strain KJC335 grown in xylose to the same strain grown in cellobiose (Fig. 1). When using a cut-off of log_2_ FC >|2| (Fig. 1), 106 out of 417 genes were categorized as highly differentially expressed genes (Supplementary Table S1) with 35 highly down-regulated and 71 up-regulated genes. Remarkably, the most down-regulated gene (CLO1313_RS09935, a hypothetical protein) displays a log_2_ FC >|6|, and the most up-regulated gene (CLO1313_RS02705, 4Fe-4S ferredoxin) shows a log_2_ FC of 7.57.

### Gene ontology analysis

To understand the functions of the DEGs and determine how the cells are impacted by xylose metabolism, the DEGs were mapped to gene ontology (GO) terms. Two strategies were used to perform the GO analysis. The first strategy consisted of determining the GO terms for all the genes with Log_2_ FC >|1| and adjusted *p* value < 0.05. This provides a *global view* of GO terms for the genes, which were then analyzed by the node score assigned by the software Blast2GO (Table 1). A higher node score indicates a greater association with the GO term. Results showed that DEGs were highly associated with GO terms for specific biological processes including carbohydrate metabolic processes, phosphorylation, chemotaxis, oxidation and reduction processes, and transport. GO terms associated with cellular component fall mainly in integral components of the membrane and much less in cytoplasm and others. GO terms for molecular function were obtained for ATP binding, transferase activity, and metal ion binding.

**Table 1:** A global view of the GO terms associated with DEGs and their corresponding node score and category.

GO term	Node score	Category
Cytoplasm	30.60	Cellular component
Plasma membrane	28.44	
Non-membrane-bounded organelle	16.96	
Integral component of membrane	96.86	
Carboxylic acid metabolic process	19.81	Biological processes
Carbohydrate metabolic process	34.15	
Nucleobase-containing compound biosynthetic process	4.44	
Organonitrogen compound biosynthetic process	12.90	
Oxidation–reduction process	30.98	
Phosphate-containing compound metabolic process	20.69	
Signal transduction	49.60	
Small molecule biosynthetic process	2.23	
Amide biosynthetic process	11.18	
Transmembrane transport	37.36	
Organic substance catabolic process	8.91	
Cellular macromolecule biosynthetic process	18.55	
Gene expression	18.08	
Cellular protein metabolic process	20.28	
Chemotaxis	24.00	
Transferase activity	33.95	Molecular function
Metal ion binding	33.16	
Hydrolase activity	107.80	
Cofactor binding	27.36	
Nucleic acid-binding	26.36	
Oxidoreductase activity	36.34	
ATP binding	43.00	

The node score was assigned by the software Blast2GO.

**Figure 2:**
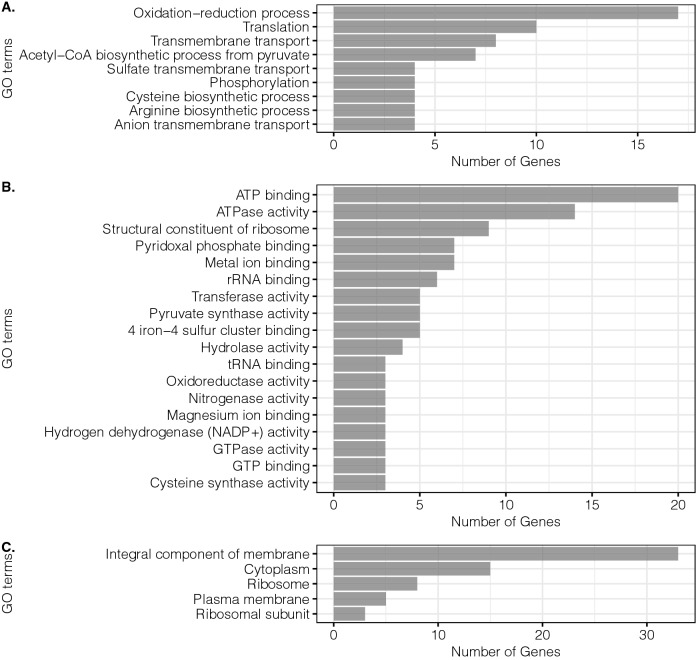
Main GO terms for down-regulated genes in KJC335. (**A**) Biological process. (**B**) Molecular function. (**C**) Cellular Component. The figure was created using the R package ggplot2^30^.

**Figure 3:**
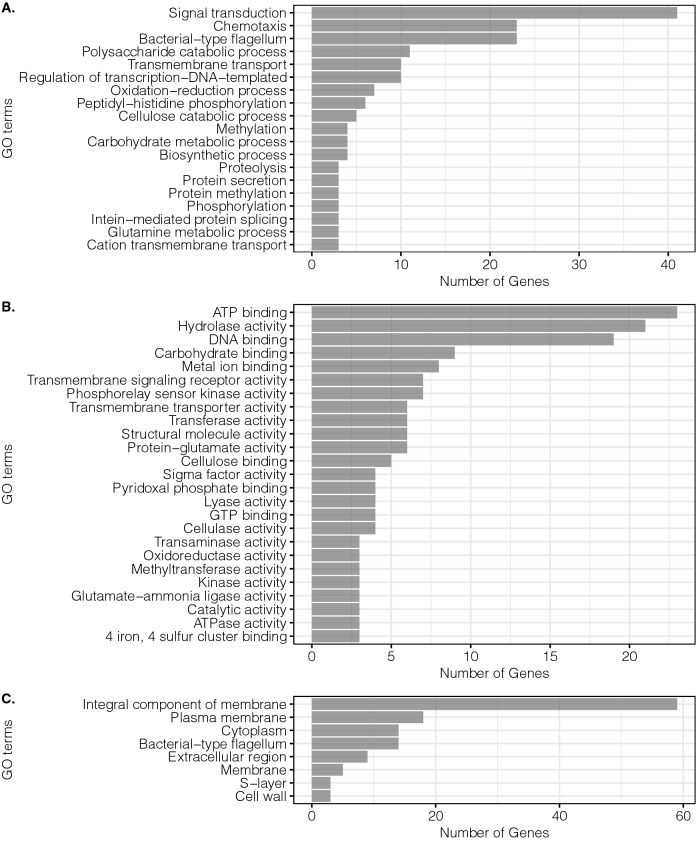
Main GO terms for up-regulated genes in KJC335. (**A**) Biological process, (**B**) molecular function, (**C**) cellular component. The figure was created using the R package ggplot2^30^.

To distinguish the up-regulated from the down-regulated adaptations to xylose metabolism, the second strategy consisted of a *differential analysis* that considers down- and up-regulated DEGs independently. Figures 2 and 3 show the most significant GO terms (at least three genes predicted in the same GO term) classified in biological processes, molecular function, and cellular components for down- and up-regulated genes, respectively. It is important to note that terms with a similar function were grouped under the same parent terms. For instances, DNA binding includes sequence-specific DNA binding, hydrolase activity takes into account hydrolase activity acting on glycosyl bonds, and hydrolase activity hydrolyzing O-glycosyl compounds, ATPase activity also includes ATPase activity coupled to transmembrane movement of substances, cellulosic activity includes cellulose 1,4-beta-cellobiosidase activity [reducing end] and simply cellulose 1,4-beta-cellobiosidase activity, signal transduction includes phosphorelay signal transduction system, bacterial flagellum includes bacterial-type flagellum-dependent cell motility and bacterial-type flagellum organization. For the down-regulated DEGs, the highest GO terms were mapped to oxidation–reduction processes, ATP binding and ATPase activity, and integral components of the membrane (Fig. 2).

Interestingly, for both up- and down-regulated DEGs, results showed that ATP binding is the molecular function with the highest number of genes involved (Figs. 2B,3B) and integral component of membrane tops the GO terms in cellular component (Figs. 2C,3C), indicating their significance in facilitating *C. thermocellum* adaption to xylose. In contrast, GO terms related to chemotaxis and motility were widely mapped for two categories on the up-regulated DEGs (Fig. 3A,C). Notice that depending on the gene, transmembrane transport and ATP-binding cassette (ABC) transporter genes were both up- and down-regulated based on GO analysis. We hypothesize that since *C. thermocellum* was not evolved to utilize xylose as the main carbon source for growth, the transcript levels of genes involved in the machinery presumed to facilitate xylose transport were considerably perturbed as a means to compensate for the inefficiencies in transporting xylose to support growth.

To identify how the DEGs may play a role during xylose metabolism in *C. thermocellum*, an analysis was performed to represent DEGs in metabolic pathways using the Kyoto Encyclopedia of Genes and Genomes (KEGG) database^31–33^. Complementary to GO analysis, Pathview^34^ was used as the bioinformatics platform to perform functional classification and pathway assignment of the up- and down-regulated DEGs (Log_2_ (FC) >|1| and adjusted *p* value < 0.05) shown in Fig. 1. In doing so, 67 of the 467 genes (Supplementary Table S2) could not be mapped since KEGG still uses an older locus tag system for *C. (Ruminiclostridium) thermocellum.* There is not an equivalent accession number for these genes in KEGG. The remaining DEGs were shown to involve around 30 KEGG pathways including carbon metabolism, ABC transporters, bacterial chemotaxis, amino acid metabolism (mainly cysteine and methionine metabolism), pyruvate metabolism, and biosynthesis of secondary metabolites (Supplementary Table S3).

### D-Xylose transport and assimilation in KJC335

**Figure 4:**
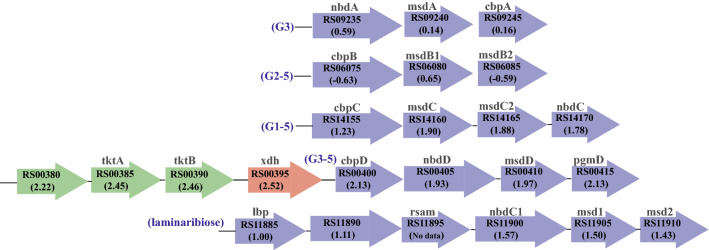
Five ATP-binding sugar transporters in *C. thermocellum* and the putative operons of the transport systems. The numbers in parenthesis are log_2_ fold changes. RS numbers in bold correspond to the numerical suffix of locus tags in *C. thermocellum* DSM 1313 (e.g., RS09235 = CLO1313_RS09235). Previous characterization of cellodextrin-binding proteins (Cbp) subunits revealed that CbpA binds only to cellotriose (G3), CbpB binds to cellodextrins ranging from cellobiose to a cellopentose (G2-G5), while CbpC and CbpD preferentially bind to cellotriose, -tetrose, and -pentose (G3–G5)^37^. Differential fold changes among the putative *cbpC*, *cbpD*, and *lbp* bearing operons suggest that one or more of these solute-binding proteins may bind promiscuously to xylose and facilitate xylose transport. Abbreviations: msd for membrane-spanning domain, nbd for nucleotide-binding domain, *tktA* for transketolase subunit A, *tktB* for transketolase subunit B, *xdh* for xylitol dehydrogenase.

Five putative ATP-dependent ABC sugar transporters were previously identified in *C. thermocellum*, and their purified solute-binding proteins were shown to interact with hexose sugars of varying lengths (cellobiose [G2] to cellopentaose [G5]) with different affinities^35^. To gain insight into which of the sugar transporter systems may be involved in xylose uptake, we examined the log_2_ FC of all five transporters in *C. thermocellum* previously reported by Nataf and co-workers^35^. In KJC335, our RNA-seq data showed that these neighboring genes, CLO1313_RS00400, CLO1313_RS00405, and CLO1313_RS00410 display a Log_2_ FC of 2.13, 1.93, and 1.97, respectively (Fig. 4), with CLO1313_RS00400 being the previously reported cellodextrin binding protein, CbpD. Although both CLO1313_RS00400 and CLO1313_09235 genes (CbpD and CbpA, respectively, in *C. thermocellum* ATCC 27405) display homology to the arabinose-binding protein, AraP, from *Geobacillus sterothermophilus,* CLO1313_RS09235 displays a log_2_ FC of only 0.59 and is not as responsive to the presence of xylose as CLO1313_RS00400. The up-regulation of the CbpD operon is consistent with a previous study by Verbeke et al*.* when the DSM 1313 derived strains without the ability to utilize xylose for growth were challenged with pentose sugar^29^. In this study, CbpD was suggested to be a xylose transporter^29^*.* Interestingly, when we modified the genomic copy of the *CLO1313_RS00405* gene encoding ATPase component of the CbpD transporter in the KJC335 genome (mutation of glycine at 315 residues of this protein to serine), improved growth on xylose (5 g/L) as the primary carbon source was observed (Supplementary Figure S1). This Gly315Ser point mutation is one of the six point mutations acquired from evolving a *C. thermocellum* strain bearing a plasmid-based expression of the same *xylAB* genes to grow on xylose (5 g/L) in a separately performed adaptive laboratory evolution, and this particular evolved mutant (a clonal isolate from a mixture of evolved mutants) displayed significantly faster growth on xylose (Supplementary Figure S2). However, when we deleted the gene *clo1313_RS00405* (same as *clo1313_0078* using the previous locus tag system) in KJC335, the growth of the mutant was not affected when either cellobiose (5 g/L) or xylose (5 g/L) was the primary carbon source (Supplementary Figure S3). The up-regulated expression of the operon bearing *cbpD* and our mutants growth data combined suggest that CbpD transporter can be involved in xylose uptake, but another transporter(s) may compensate for CbpD in its absence. Alternatively, it is possible that another ATPase subunit of an ABC transporter can supplement the activity of the deleted one, which has been suggested previously in other systems^36^. This may warrant further deletions of the other two genes encoding the transmembrane and solute binding proteins of the ABC transporter.

In addition to CbpD transporter, two other predicted sugar transporter operons containing CbpC (CLO1313_RS14155-170) and laminaribiose binding protein (Lbp, CLO1313_RS11885-910) also showed an increase in log_2_ FC while the CbpB (CLO1313_RS06075) containing operon was not highly differentially expressed (Fig. 4). Combining our data with the reported literature, the results suggest that CbpC, CbpD, and Lbp might play a role in xylose uptake to differential extents. Validation of specific ABC transporters involved in facilitating xylose uptake in vivo will likely require deletion or inactivation of the other transporters followed by extensive growth characterization and is not pursued herein.

Amongst the highly up-regulated DEGs responsive to D-xylose shown in Fig. 1, genes *clo1313_RS00385* and *clo1313_RS00390* encode for transketolase enzyme subunits TktA and TktB, which are part of the non-oxidative pentose phosphate pathway (PPP). Transketolase typically catalyzes the reversible reaction of xylulose 5-phosphate (X5P) and erythrose 4-phosphate (E4P) to fructose 6-phosphate (F6P) and glyceraldehyde 3-phosphate (G3P), which provides a pathway to convert PPP intermediates into glycolysis. The up-regulation of TktAB therefore suggests that the xylose catabolizing strain employs this particular pathway to metabolize D-xylose. Notice that there are another predicted TktAB encoding genes (*clo1313_RS01525* and *clo313_RS01530*) also annotated in the DSM 1313 genome and are generally higher in expression than CLO1313_RS00385-390 in strains without the engineered XylAB pathway. However, *clo1313_RS01525* and *clo1313_RS01530* genes displayed a log_2_ FC of − 0.14 and − 0.04, respectively, and are not as responsive to xylose as *clo1313_RS00385-390*. The transaldolase-encoding gene often found in the non-oxidative PPP in other bacteria is not found in the *C. thermocellum* genome.

**Figure 5:**
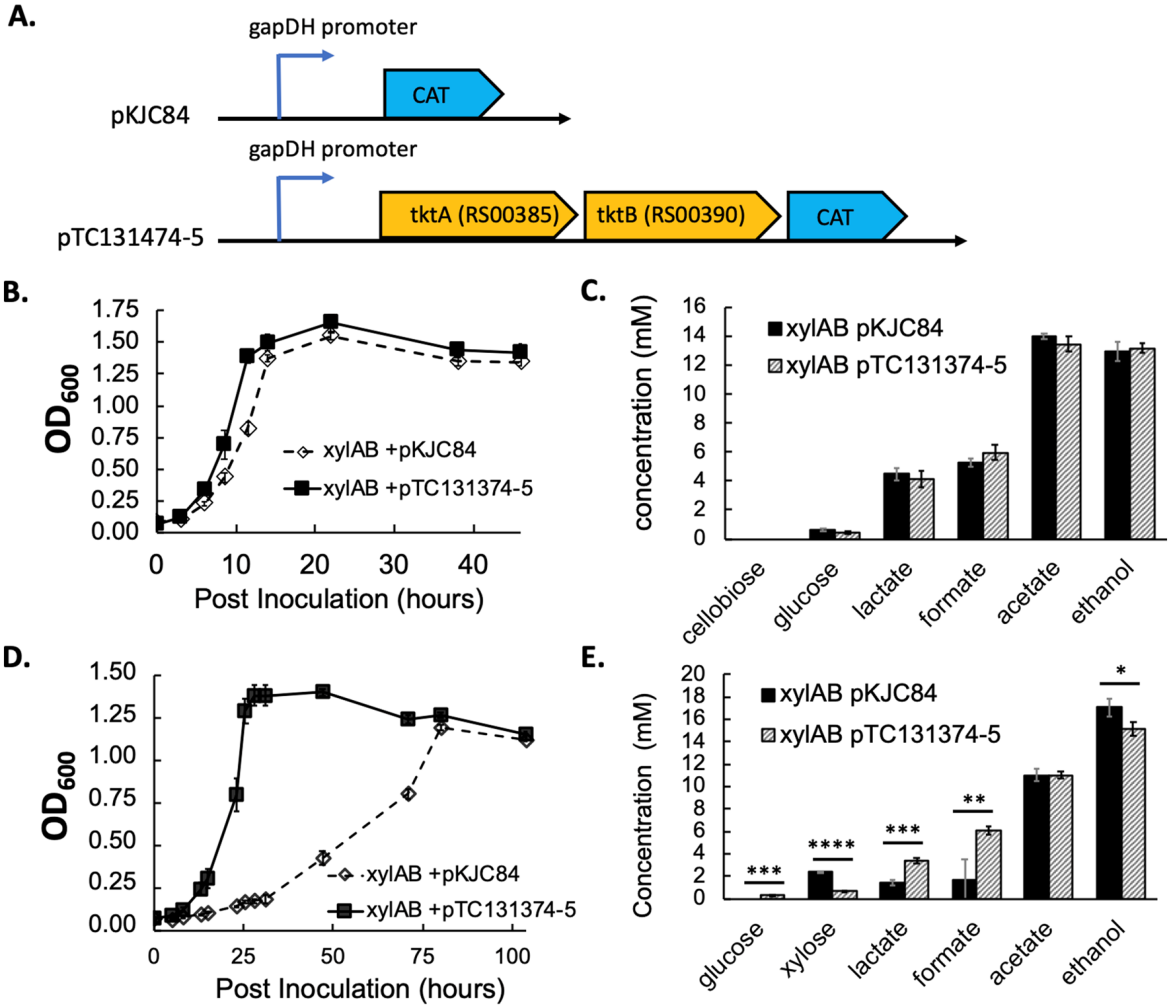
Effect of overexpressing transketolase in KJC335 on growth when either cellobiose or xylose is provided as the primary carbon source. (**A**) Constructs of a control plasmid pKJC84, which does not bear transketolase genes, and a plasmid, pTC131374-5, expressing *tktAB* genes. The non-coding regions upstream of glyceraldehyde phosphate dehydrogenase gene, also referred to as the “gapDH promoter,” is transcriptionally fused to the genes shown in the synthetic operons. Chloramphenicol resistance gene (CAT) is used for selection of the plasmid using thiamphenicol, a thermostable analog of chloramphenicol. (**B**) The growth curves of strains with or without expressing the *tktAB* genes on 5 g/L of cellobiose. (**C**) Concentrations of major metabolic end-products measured at the end of cellobiose fermentation shown in (**B**). (**D**) The growth curves of strains with or without expressing the *tktAB* genes on 5 g/L of xylose. (**E**) Concentrations of major metabolic end-products measured at the end of xylose fermentation shown in (**D**) (n ≥ 3, data reported as average ± SD). Student’s *t* tests were performed to assess significance, where (*) corresponds to a *p* value < 0.05, (**) to a *p* value < 0.01, (***) to a *p* value < 0.001, and (****) *p* value < 0.0001.

To test whether the up-regulated transketolase can be a rate-limiting step in xylose catabolism, we overexpressed *clo1313_RS00385*–*390* genes on a plasmid driven by a glyceraldehyde phosphate dehydrogenase promoter (*gapDH* promoter) in KJC335 (Fig. 5A). While our results showed that *tktAB* overexpression (using plasmid pTC131374-5) led to no major differences with the control when grown on 5 g/L of cellobiose (control refers to KJC335 bearing an expression vector, pKJC84, without *tktAB* genes, Fig. 5B and C), overexpression of this transketolase substantially improved the growth of *C. thermocellum* on 5 g/L xylose (Fig. 5D). The doubling time for KJC335/pKJC84 is 19.0 ± 5.7 h and the overexpression of the transketolase reduced the doubling time to 5.8 ± 0.6 h (data reported as average ± SD with n ≥ 3). Given the same amount of fermentation time, the overexpression strain had utilized nearly all the xylose by the end of fermentation. Still, the control strain left about 3.5 times more xylose in the culture supernatant than the overexpression strain (Fig. 5E). Furthermore, there was increased production of lactate and formate in the overexpressing strain at the end of xylose fermentation. Our data indicated that transketolase is a rate-limiting step for xylose utilization, and the up-regulated *tkt* gene expression serves a means for KJC335 to adapt to growth on xylose as suggested by the transcriptomic analyses.

**Figure 6:**
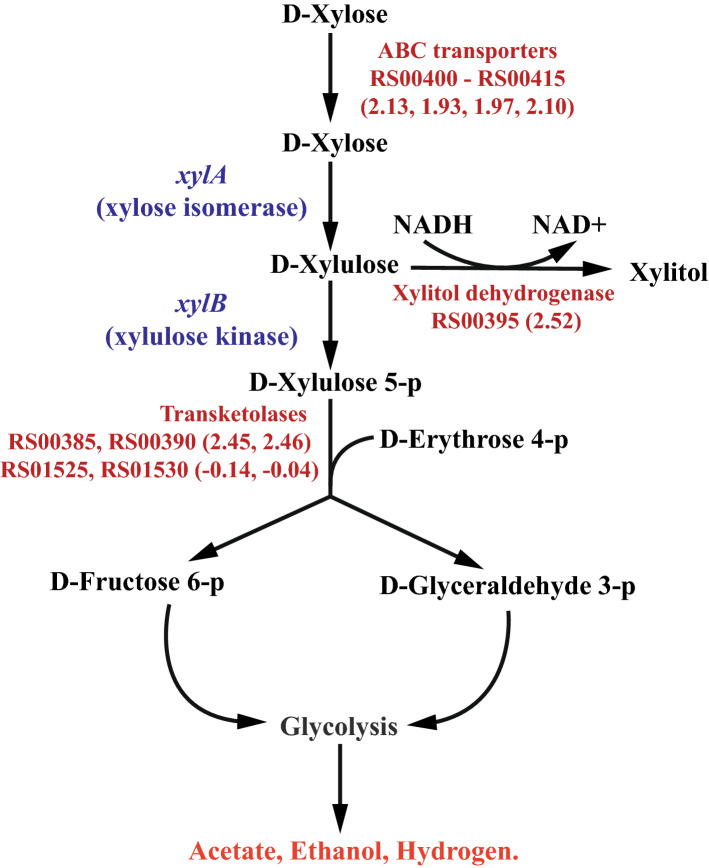
Proposed D-xylose utilization pathway in KJC335. Previous engineered genes from *T. ethanolicus* are shown in blue, and genes and pathways natives to *C. thermocellum* are in red. The numbers in parenthesis are log_2_ fold changes. RS numbers in bold correspond to the numerical suffix of locus tags in *C. thermocellum* DSM 1313 (e.g., RS00385 = CLO1313_RS00385).

On the other hand, the log_2_ FC of the predicted NADH-linked xylitol dehydrogenase gene (CLO1313_RS00395) is 2.52, and this significant up-regulation suggests an active xylitol formation pathway. It is also important to note that an active xylitol dehydrogenase (Xdh) will likely consume the NADH pool for the formation of xylitol and, as such, have a consequence on the redox balance inside the bacteria. Collectively, Fig. 6 depicts the xylose catabolic pathway proposed in KJC335. In this model, the xylose metabolism is catalyzed by the *xylA* and *xylB* genes from *T. ethanolicus* that are integrated into the bacterial genome^10^*.* D-xylulose-5-phosphate is then presumed to enter glycolysis via D-glyceraldehyde-3-phosphate and D-fructose-6-phosphate by the action of the *tkt* genes, CLO1313_RS00385 and CLO1313_RS00390 native to KJC335.

To potentially investigate the role of Xdh and whether xylitol is indeed produced to divert some of the carbon and electron flux away from PPP, we supplemented KJC335 culture 7.5 and 15 g/L of xylose, respectively, without adding cellobiose, and measured if or how much xylitol was produced. Interestingly, xylitol was detected in the culture supernatant at the end of 72-h xylose fermentation, and results showed that an increase in xylose consumption increased xylitol production as well as the xylitol molar yield per mole of xylose consumed (Table 2). The molar yield reached as high as 0.30 ± 0.04 when there was 15 g/L of xylose initially present. The increase in molar yield at higher initial xylose loading may suggest xylitol production as overflow metabolism since transketolase is likely limiting in converting the PPP intermediates into glycolysis. We also noticed that without supplementing xylose as a carbon source, xylitol is not secreted when KJC335 grew on only cellobiose at either 7.5 or 15 g/L after over 90% of the fed cellobiose was consumed.

**Table 2:** Xylitol production and yield at different initial xylose loadings.

Initial cellobiose (g/L)	0	0
Initial xylose (g/L)	7.5	15
Xylitol produced (g/L)	0.14 ± 0.11	3.10 ± 0.09
Xylitol produced (mM)	0.90 ± 0.72	20.3 ± 0.6
Xylose consumed (g/L)	3.9 ± 0.2	9.4 ± 0.9
Xylose consumed (mM)	26.0 ± 1.4	62.7 ± 5.7
Molar yield of xylitol/xylose	0.04 ± 0.03	0.30 ± 0.04

Engineering xylose transporters in an industrial *Saccharomyces cerevisiae* strain have shown to enhance xylose uptake^38^. The overexpression of genes involved in non-oxidative PPP, including transketolases, has been reported to improve the rate of xylose consumption^39,40^. Transketolases play an important role and appear essential for xylose assimilation and utilization^38^. These data suggest that further improving the xylose utilization may involve engineering strategies such as enhancing the xylose transport system and enhancing the pentose phosphate pathway^41^ by debottlenecking the rate-limiting step(s).

### Transcriptional response in cellulase and cellulosomal components

The cellulosome is a large cell-surface bound multi-enzyme complex that synergistically degrades plant cell wall polysaccharides. The *C. thermocellum* genome contains 69 genes encoding for cellulosomal cellulases, 8 non-catalytic cellulosomal proteins, and 27 cellulosome-free cellulases^17^. Among the up-regulated genes based on GO analysis (Supplementary Tables S4, S5 and S6), 5 DEGs were categorized in molecular function and involved in cellulase activity, cellulose binding, and cellobiosidase activity. These genes encode for a glycoside hydrolase (CLO1313_RS02025, log_2_ FC = 1.06), a cellulose 1,4-β-cellobiosidase, (CLO1313_RS09145, log_2_ FC = 1.24), another glycoside hydrolase (CLO1313_RS11090, log_2_ FC = 1.07), an endoglucanase (CLO1313_RS13955, log_2_ FC = 1.58), and a carbohydrate-binding protein (CLO1313_RS05110, log_2_ FC = 1.97). There are 5 DEGs that fall in the category of biological processes and are related to cellulose catabolic process. These genes encode for a cellulosome-anchoring protein (CLO1313_RS03225, log_2_ FC = 1.04), a cellulose 1,4-β**-c**ellobiosidase (CLO1313_RS09145, log_2_ FC = 1.24), a thermostable glucosidase B (CLO1313_RS05115, log_2_ FC = 1.94), a glycoside hydrolase (CLO1313_RS11090, log_2_ FC = 1.07), and an endoglucanase (CLO1313_RS13955, log_2_ FC = 1.58).

The expression profiles of *C. thermocellum* genes that encode cellulosomal enzymes and structural proteins have been reported to change in response to growth on different carbon sources^22,23,26,42–44^. Indeed, extracellular carbohydrate-sensing and signal-transduction mechanisms involving membrane-associated anti-σ factors (RsgI-like proteins) have been described in the closely related *C. thermocellum* strain, ATCC 27405^45,46^. In this proposed signal transduction mechanism, the extracellular C-terminal carbohydrate-binding domain (CBM) of an ORF, often annotated as glycoside hydrolase or membrane protein, senses (upon binding to) the target polysaccharide substrate. It then changes the protein conformation of the intracellular N-terminal subdomain and derepressed the σ-like factor that consequently induces the expression of σ-dependent genes coding for specific polysaccharide-degrading enzymes. Interestingly, CLO1313_RS09960 (encoding a peptidase) and CLO1313_RS05030 (encoding an α-L-arabinofuranosidase) in KJC335 were determined to be 100% identical to two of the nine RsgI-like carbohydrate sensing proteins (RsgI9, Cthe_0260, and RsgI5, Cthe_1273, respectively)^45^. They display log_2_ FCs of 3.16 and 0.93, respectively, while the rest of the seven RsgI-like glycoside hydrolases display the absolute values of the log_2_ FCs less than 0.70 (Supplementary Table S10). Besides these two genes, CLO1313_RS11345 determined to be 99% similar to Cthe_1471 in ATCC27405 encodes a glycoside hydrolase and was up-regulated in KJC335 (log_2_ FC = 1.01). Cthe_1471 is another type of putative anti-σ factor related to Rsi24 and contains a module that resembles a family 5 glycoside hydrolase (Rsi24C-GH5) previously reported to be a sensor in the signal transduction system in ATCC 27405^46^. These findings suggest that these anti-σ modules could sense the presence of xylose and trigger specific regulatory responses necessary to metabolize xylose. It is recognized that GH’s are a widespread group of enzymes that hydrolyze the glycosidic bond between two or more carbohydrates or between a carbohydrate and a non-carbohydrate moiety. In addition, four GH’s CLO1313_RS02025, CLO1313_RS09910, CLO1313_RS11090, and CLO1313_RS14535 were also found up-regulated (Supplementary Table S1), suggesting potential roles in ﻿extracellular carbohydrate-sensing in the presence of xylose^16,46^.

### Redox related transcriptional responses

Electron transfer in *C. thermocellum* is often speculated but not well delineated. Among a few putative ferredoxins displaying differential expression, the highest fold-change observed in Fig. 1 lies in the 7.57-fold up-regulation of CLO1313_RS02705, which is predicted to be a 4Fe-4S ferredoxin (Fd). While it is unclear which pathways are coupled to this Fd for electron transfer, pathways that may interact with Fds generally either provide electrons to generate reduced Fd (Fd_red_) or re-oxidize the reduced Fd for cellular functions. Based on a previous study^47^, there are five putative pyruvate:Fd oxidoreductase (PFOR) enzymes potentially involved in the oxidative decarboxylation of pyruvate to acetyl-CoA and transferring two electrons to generate reduced Fd (Fd_red_). Interestingly, we observed down-regulation of one of the five PFOR’s and the genes are CLO1313_RS00115-130 with log_2_ FC ranging between − 2.21 and − 2.18 (also referred to as PFOR1^48^, Supplemental Table S11). The absolute values of the log_2_ FC of the other four putative PFOR’s were below 1. Several other pathways have been predicted to re-oxidize Fd_red_ and these include two putative electron bifurcating [FeFe] hydrogenases^49^ which, stoichiometrically, acquire two electrons from NADH and two electrons from Fd_red_ to reduced four protons (H^+^) for the synthesis of two hydrogen molecules (H_2_)^50^. We observed that both bifurcating hydrogenases were down-regulated. In particular, the putative operon containing the tetrameric bifurcating hydrogenase (CLO1313_RS09510-530) displayed log_2_ FC around − 1.13 to − 0.96, while the putative trimeric bifurcating hydrogenase showing log_2_ FC between − 0.86 and − 0.21. The third [FeFe] hydrogenase shown to be NADPH-dependent^51,52^ was slightly up-regulated and with log_2_ FC below 1 (Supplemental Table S11). Other reactions that can potentially link to the ferredoxin include the reduction of NADP^+^ to generate NADPH via the electron-bifurcating NADH-dependent reduced Fd:NADP^+^ oxidoreductase (also known as the NfnAB complex with gene locus CLO1313_RS09340-345) and the membrane-associated reduced Fd: NAD^+^-oxidoreductase (also known as Rnf complex with gene locus CLO1313_RS00325-350). However, the expression of these genes did not meet the significance threshold of fold changes and display log_2_ FC between − 0.26 to 0.11 (Supplemental Table S11). In addition to the enzymes mentioned above, a highly down-regulated CLO1313_RS11835 (log_2_ FC = − 2.97), which according to KEGG, encodes for an oxidoreductase involved in N_2_ fixation.

### Sulfate transport and metabolism, and links to stress responses

Key genes in the assimilatory sulfate reduction pathway are present in *C. thermocellum*, and sulfur is required for the bacterial growth^51^. The genes, CLO1313_RS00605 and CLO1313_RS00620 with log_2_ FC of − 2.79 and − 2.22, respectively, encode a sulfate transporter subunit and sulfate ABC transporter ATP binding protein, respectively (Fig. 1). Immediately adjacent to these genes on the genome encode the sulfate activation enzymes (CLO1313_RS00630 and CLO1313_RS00635 with log_2_ FCs of − 1.55 and − 1.21 for sulfate adenylyltransferase and adenylylsulfate kinase, respectively) and the reduction to sulfite (CLO1313_RS00625 with log_2_ FC of − 1.94 for phosphoadenosine phosphosulfate reductase) involved in the super-pathway of sulfate assimilation and cysteine biosynthesis (Supplementary Figure S4, Supplementary Table S11). Previously, sulfate transporter has been suggested to be up-regulated during stress conditions^18^. While it is unclear how the xylose catabolism links to the down-regulation of sulfate transport, it is possible that the down-regulation of genes involving sulfur transporters is a reversal of the transcriptional response to cellobiose feeding. This sulfate transport/metabolism down-regulation was also observed in *C. thermocellum* cells exposed to methyl viologen^53^. In addition, the down-regulation of cysteine synthase (CLO1313_RS11805 with log_2_ FC = − 2.46), which is part of the cysteine biosynthesis pathway could be a result of down-regulated sulfate transport/metabolism. Cysteine is the sulfur donor for the biogenesis of the iron-sulfur (Fe–S) clusters that are found in the catalytic site of numerous enzymes and assists in protein folding and assembly by forming disulfide bonds^54^. Iron-sulfur clusters play critical roles of electron transfer, modulating enzyme activities, as well as being involved in substrate binding and activation of dehydratases^55^. Because iron and sulfur species can be potentially toxic to cells, Fe–S clusters are synthesized by specialized systems including cysteine desulfurases (*iscS*)^17^. Interestingly, a putative Fe–S cluster regulatory protein (IscR, CLO1313_RS00570 with log_2_ FC = − 1.53) was shown to be down-regulated. The potential gene regulation by IscR on Fe–S cluster synthesis, sulfur metabolism, and stress responses remains to be studied. The up-regulation of CLO1313_RS02710 (log_2_ FC = 6.13) was also observed under xylose growth. This gene appears to function as a membrane protein according to the BioCyc database. However, the KEGG database suggests this protein to contain a 4Fe–4S binding domain. Specific role or functions of this protein remains to be further studied.

### Chemotaxis and motility transcriptional response

**Figure 7:**
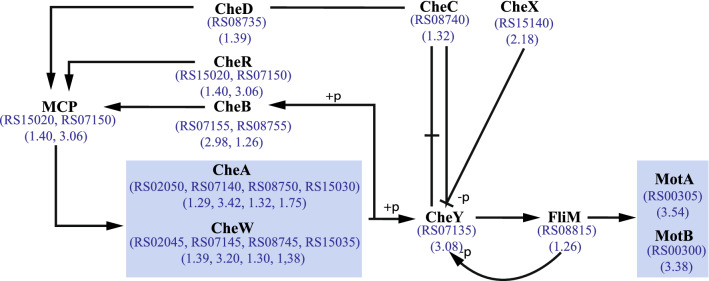
A general scheme for bacterial chemotaxis. The numbers in parenthesis are log_2_ FC. RS numbers in blue are the numerical suffix of locus tags in *C. thermocellum* DSM 1313 (e.g., RS08740 = CLO1313_RS08740). Blue boxed represents proteins that are grouped based on the KEGG map. Arrows represent activation, whereas the “T” shaped stick represents inactivation. The line with a “-” represents dissociation. The symbol “ + p” denotes phosphorylation and “-p” dephosphorylation. This general scheme is adapted from KEGG maps with modifications^32,33^. MCP symbolizes methyl-accepting chemotaxis proteins, the Che genes are involved in chemotaxis, Mot genes are involved in motility, and FliM is a flagellar motor switch protein.

Bacterial chemotaxis is a process by which microorganisms can move in response to chemical stimuli, which includes the ability to move toward the direction of nutrients in the environment^17^. Figure 7 summarizes the general scheme for chemotaxis in bacteria and presents a model for how DEGs in chemotaxis may be involved in KJC335 in response to xylose feeding. These genes are up-regulated and highly differentially expressed.

The four critical elements of the signal transduction system of bacterial chemotaxis are (1) Chemoreceptors (methyl-accepting chemotaxis proteins, or MCPs); (2) A histidine kinase, CheA; (3) A receptor-coupling protein, CheW; and (4) Receptor-modification enzymes, CheR, and CheB; all clustered into a protein complex. The chemoreceptors (MCPs) detect different environmental and intracellular signals, regulate the activity of CheA, and communicate the signal to the flagellar system via phosphorylation of its response regulator, CheY^56–58^. Mot genes encode for flagellar motor protein, and FliM is a flagellar motor switch protein. All the genes encoding the aforementioned proteins were differentially expressed in *C. thermocellum* during growth on xylose. Studies have reported that motility and chemotaxis protein expression increase when the bacteria are under stress conditions^17^.

On the other hand, interestingly, the up-regulated CLO1313_RS00400 in the presence of xylose, which has been previously reported as a sugar ABC transporter substrate-binding protein, is also denoted as the *rbsB* gene based on Pathview results. In *E. coli*, RbsB is the periplasmic ribose-binding protein of an ATP-dependent ribose uptake system. The ribose-binding protein is necessary for chemotaxis towards ribose. When ribose binds RbsB, it also interacts with the Trg sensory protein to mediate taxis to the sugar^59,60^. Trg proteins have been described to be related to respond to changes in the environment or transduce a signal from the outside to the inside of the cell.

Considering that *C. thermocellum* does not have the natural ability to use xylose as a carbon source, it is unclear if or how the bacterium actively senses xylose. It is also probable that the transcriptional responses in chemotaxis and motility are stress response mechanisms induced to search for a better carbon source. Our data collectively suggest that the bacteria’s motility can be enhanced in KJC335 to render free movements in the growth media supplemented with xylose as the bacteria search for a better or recognizable carbon source. These results suggest potentially increased motility of KJC335 during growth on xylose as a cellular strategy oriented towards enhancing the cells’ ability to sense the environment and appropriately respond to the ambient signals through activating the cellular motility systems.

Distinct from a previous study in which xylose was supplemented to *C. thermocellum* strains that cannot consume xylose and the added xylose inhibited *C. thermocellum* growth on cellobiose^29^, the study herein identifies genes perturbed in response to xylose- versus cellobiose-feeding in a strain engineered to grow on xylose. It is also important to note that there is a large difference in the specific growth rates between cultures grown in xylose (0.061 ± 0.005 h^−1^) versus cellobiose (0.149 ± 0.021 h^−1^). Although the cells were harvested for total RNA at the same physiological state (mid-log phase) to reduce the growth effect on gene expression, this growth rate difference may partially contribute to the differential gene expression profile observed. For instance, sulfur metabolism, iron-sulfur cluster synthesis, and repair mechanisms may be more active in cells with faster growth rates, since the bacteria are more metabolically active than those displaying slower growth rates. The downregulation of IscR may be linked to the differences in growth rates.

In summary, our transcriptomic analyses revealed candidate ABC transporters required for xylose transport and assimilation. Genes in the pentose phosphate pathway such as TktAB important in facilitating xylose metabolism was suggested and validated experimentally. Transcriptomic data also revealed potential xylose-sensing enzymes which signals the presence of carbon sources to the cells, and chemotaxis and mobility associated genes likely required for the bacteria to move toward the carbon food sources. The findings provide physiological insights into how an anaerobic thermophile adapts to grow on a carbon source not naturally metabolized by the bacteria and potential strategies to further engineer *C. thermocellum* for improved utilization of five-carbon substrates including xylose, xylo-oligomers, xylan, and hemicellulose to fully realize NG-CBP.

## Materials and methods

### Strains and growth conditions

Strain KJC335 was previously generated by integrating the *Thermoanaerobacterium ethanolicus xylAB* genes in the genome of Δ*hpt* strain (KJC315) derived from *Clostridium thermocellum* DSM1313^10^. This engineered strain was routinely maintained anaerobically in CTFUD media^61^ supplemented with 5 g/L cellobiose unless otherwise specified. All glass tubes containing the CTFUD medium are bubbled with ultra-high purity argon gas for 15 min, sealed, and then autoclaved before cells and carbon source were added from stocks (i.e., 10% w/v cellobiose and xylose stocks) that are also kept sterile anaerobically.

To construct the *tktAB*-expressing plasmid, *clo1313_RS00385-390*, encoding TktAB, were amplified off of purified *C. thermocellum* genomic DNA. The plasmid, pTC131374-5, was constructed by integration of the amplified DNA product into the PacI site of pTC84, a pKJC84 derivative with a PacI site between the *gapDH* promoter and CAT gene, using the NEBuilder HiFi DNA Assembly kit. The product was transformed by heat shock into NEB 5-alpha competent *E. coli*. Spectinomycin (50 mg/L) selected for the presence of the plasmid and colonies were screened by colony PCR. Colonies with the correct band size were selected for plasmid prep and confirmed by sequencing. *C. thermocellum* transformation protocol and detection of sugars and organic acids with HPLC follow the same methods reported previously^10^.

For growth curve measurements, 0.5 mL of cells were transferred into minimal media with either 0.5% (w/v) of cellobiose or xylose and grown overnight. On the next day, 0.5 mL of culture was transferred to minimal media containing the respective carbon source at 0.5% (w/v) and grown overnight. These cultures were used as the inoculum for the growth assays. 15 µg/mL of thiamphenicol was added to cultures carrying the chloramphenicol acetyltransferase (CAT) thiamphenicol resistance gene. OD600 of cultures was monitored over the course of multiple days, and high-performance liquid chromatography (HPLC) samples were taken at the beginning (0 h) and the last readings.

### Bacteria cells growth, harvesting, and total RNA purification

To compare the global gene expression profiles between cells grown in cellobiose vs. xylose, three biological replicates of the *xylAB*-expressing strain (KJC335) were grown in either cellobiose (5 g/L, n = 3) or xylose (5 g/L, n = 3) until harvest. Briefly, KJC335 was inoculated into glass tubes of 10.5 mL of CTFUD defined medium^62^ containing either cellobiose or xylose, and with close to 15 mL of argon gas in the headspace. The final concentrations of all components in the defined medium are: 3 g/L of sodium citrate tribasic dihydrate, 1.3 g/L ammonium sulfate, 1.5 g/L potassium phosphate monobasic, 0.13 g/L of calcium chloride dihydrate, 0.5 g/L of L-cysteine-HCl, 11.56 g/L of 3-morpholinopropane-1-sulfonic acid (MOPS) sodium salt, 2.6 g/L magnesium chloride hexahydrate, 0.001 g/L of ferrous sulfate heptahydrate, 0.001 g/L resazurin, and 5 g/L of either cellobiose or xylose. A 25 mL of 1,000 × vitamin solution containing 50 mg pyridoxamine HCl, 5 mg biotin, 10 mg of p-aminobenzoic acid (PABA), and 5 mg of vitamin B_12_ were prepared, and 1 mL of the solution was added to a liter of the defined medium. The number of cells to add from the seeding culture was calculated so that the initial OD_600_ was between 0.08 and 0.09 for all tubes and all cultures were harvested for total RNA purification at mid-log phase (e.g., OD_600_ between 0.45 and 0.50). Since there is a large difference in the specific growth rates between cultures grown in cellobiose (0.149 ± 0.021 h^−1^) versus xylose (0.061 ± 0.005 h^−1^), harvesting cells at the same physiological states for all samples were intended to minimize the growth effect on the transcriptomic responses to xylose. However, growth rate difference can unintendedly contribute to the differential gene expression profile observed. Carefully lining up the initial and harvest OD_600_ ensures that all cultures underwent the same number of doubling for comparison. The seeding culture was a fresh overnight culture also grown in defined media to minimize yeast extract carryover, and the culture was at its late log phase at inoculation. No sodium bicarbonate was added in any of the tubes.

To harvest the cells for total RNA purification, it was first chilled by swirling the anaerobic culture in dry ice/70% ethanol for 45 s and kept on ice for 5 min. The inactivated cells (1–1.5 mL) were then pelleted by centrifugation. The supernatant was discarded, and the cell pellets were lysed with lysozyme-EDTA using a Qiagen RNeasy Mini kit (Cat No./ID: 74104) following the manufacturer’s protocol. Each sample was treated with DNaseI (Qiagen, Cat No./ID: 79254) twice following the protocol to remove the genomic DNA in the total RNA completely. The quality of each purified total RNA sample was checked and validated by electrophoresis before it was sent out to commercial RNA-seq services for further sample processing and data analyses (Genewiz, South Plainfield, NJ).

### Transcriptome analysis

Clean raw data was obtained (accession number GSE137509, deposited to the Gene Expression Omnibus database) following the protocol of Genewiz. SARTools 1.5.0 (Statistical Analysis of RNA-Seq data Tools)^63^ was used for statistical RNA-Seq analysis. Differentially expressed genes (DEGs) were identified using the DESeq2^64^ in R version 3.5.2^65^. Two cutoffs were used to determine DEGs in DESeq2, the adjusted p-value < 0.05 and the log2 FC > 2. Gene annotation from BioCyc was used for all analyses^66^,⁠ using *Ruminiclostridium thermocellum* DSM 1313 as the organism database. The functional classification of the DEGs was performed using the gene ontology (GO) analysis by Blast2Go^67^. The visualization of DEGs and GO analysis were created using the R package ggplot2^30^. The pathway-based analysis further informed the biological functions of the DEGs, indicating the highly enriched metabolic or signal transduction pathways compared to the whole genome background. Pathview^34^ was used to map differential expressed genes on KEGG database^32,33^.

## Supplementary information

Supplementary Information.
