# Supplementary material for: Transcriptomic analysis of a Clostridium thermocellum strain engineered to utilize xylose: responses to xylose versus cellobiose feeding
Source: Sci Rep. 2020 Sep 3;10:14517. doi: 10.1038/s41598-020-71428-6 (PMC7471329; doi:10.1038/s41598-020-71428-6)
Supplement: Supplementary file 1 — Supplementary Information. [file 41598_2020_71428_MOESM1_ESM.pdf]

Supplementary Information

Transcriptomic analysis of a *Clostridium thermocellum* strain engineered to utilize xylose: responses to xylose versus cellobiose feeding

Albert E. Tafur Rangel[1,2], Trevor Croft[3], Andrés González Barrios[1], Luis H. Reyes[1*], Pin-Ching Maness[3*], Katherine J. Chou[3*]

[1] Grupo de Diseño de Productos y Procesos (GDPP), Department of Chemical and Food Engineering, Universidad de los Andes, Bogotá, Colombia.
[2] Grupo de Investigación CINBIOS. Department of Microbiology, Universidad Popular del Cesar, Valledupar-Cesar, Colombia.
[3] Biosciences Center, National Renewable Energy Laboratory, Golden, Colorado, United States

*Corresponding authors:

Katherine J. Chou; Katherine.chou@nrel.gov
Pin-Ching Maness; PinChing.Maness@nrel.gov
Luis H. Reyes; lh.reyes@uniandes.edu.co

Content

Supplemental Figure S1. 2
Supplemental Figure S2. 2
Supplemental Figure S3. 3
Supplemental Figure S4. 4
Supplemental Table S1. 5
Supplemental Table S2. 106
Supplemental Table S3. 108
Supplemental Table S4. 109
Supplemental Table S5. 113
Supplemental Table S6. 119
Supplemental Table S7. 121
Supplemental Table S8. 128
Supplemental Table S9. 136
Supplemental Table S10. 139
Supplemental Table S11. 140

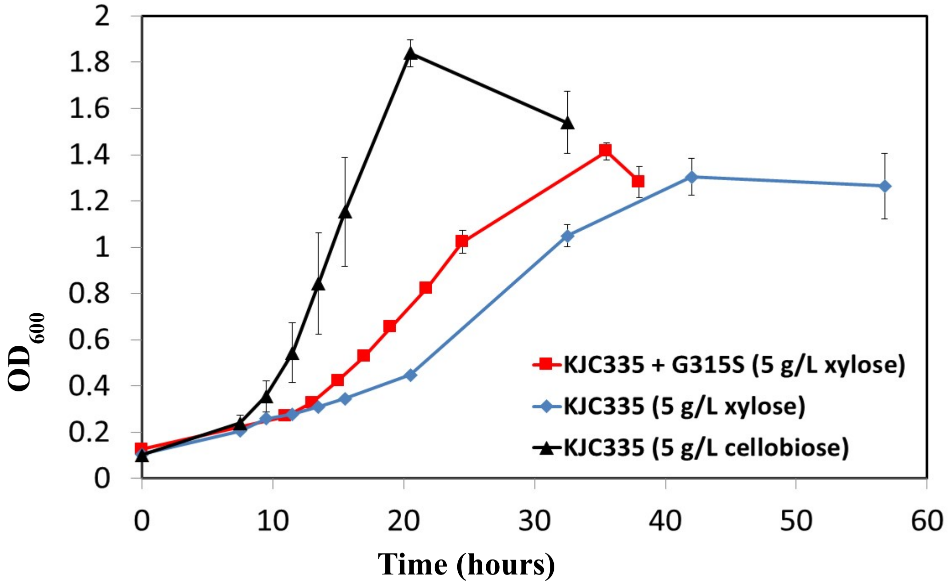

Supplemental Figure S1. Re-creating G316S point mutation acquired in the gene, CLO1313_RS00405 (nbdD gene), on the KJC335 genome improved the growth of the unevolved KJC335 strain in xylose (5 g/L). This data suggests that the CLO1313_RS00405 gene being part of the CbpD transporter operon is partially responsible for xylose uptake (n = 3, data is reported as average ± Stdev).

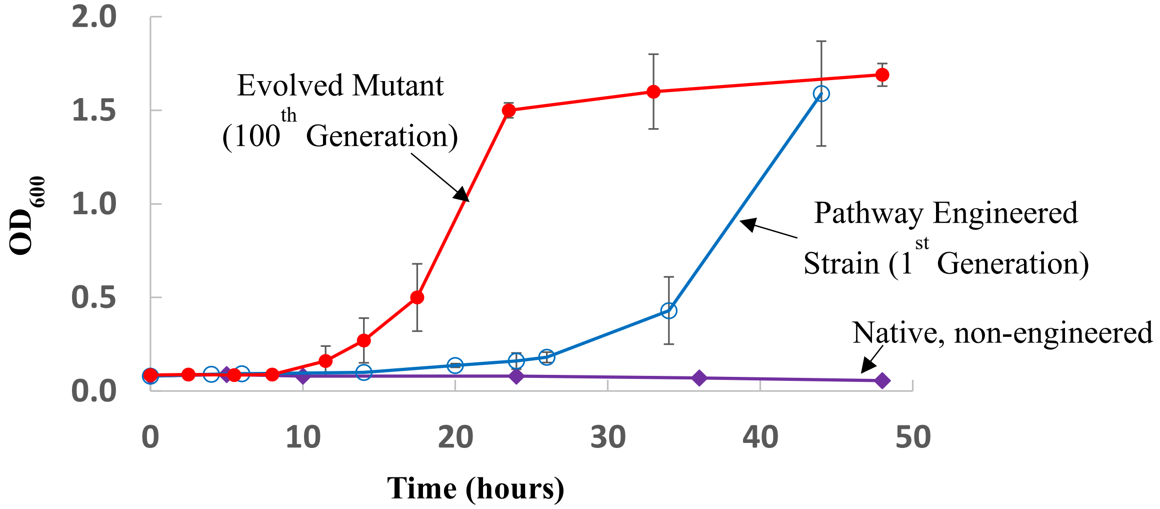

Supplemental Figure S2. Growth profiles of a strain bearing the *xylAB* genes before evolution and an evolved mutant isolated from a mixture of evolved mutant. All growth curves were measured when xylose (5 g/L) was supplemented as the main carbon source. The evolved mutant grows significantly faster than engineered but unevolved strain. The Gly315Ser point mutation is one of the six point mutations identified only in the evolved mutant but not in the unevolved strain shown above (n = 3, data reported as average ± Stdev).

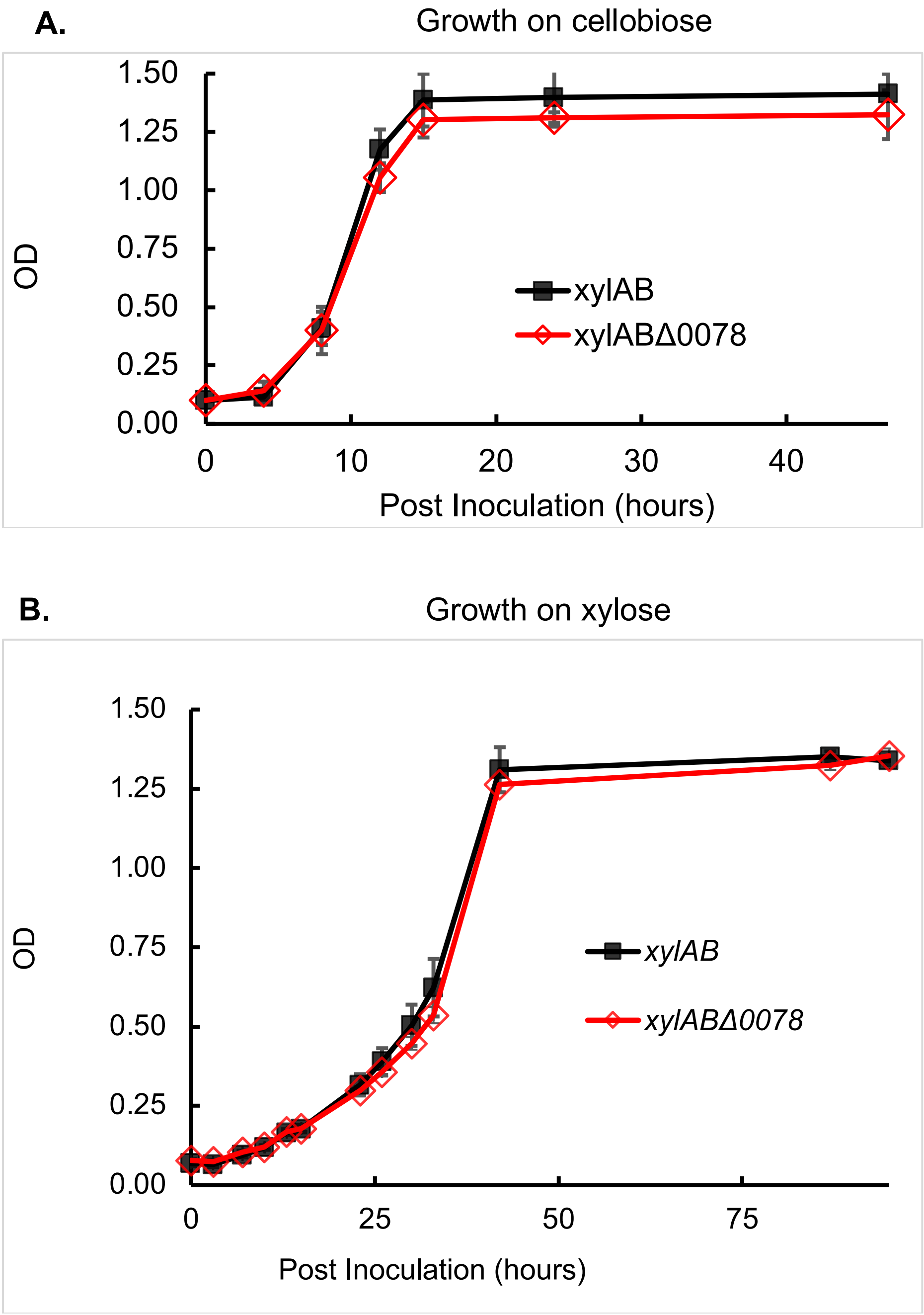

Supplemental Figure S3. Deletion of *clo1313_RS00405* (*clo1313_0078*) encoding for ATPase subunit of the CbpD transporter does not affect KJC315's growth on 5 g/L of cellobiose (A) or 5 g/L of xylose (B) as the primary carbon source ($n \geq 4$, data reported as average ± stdev).

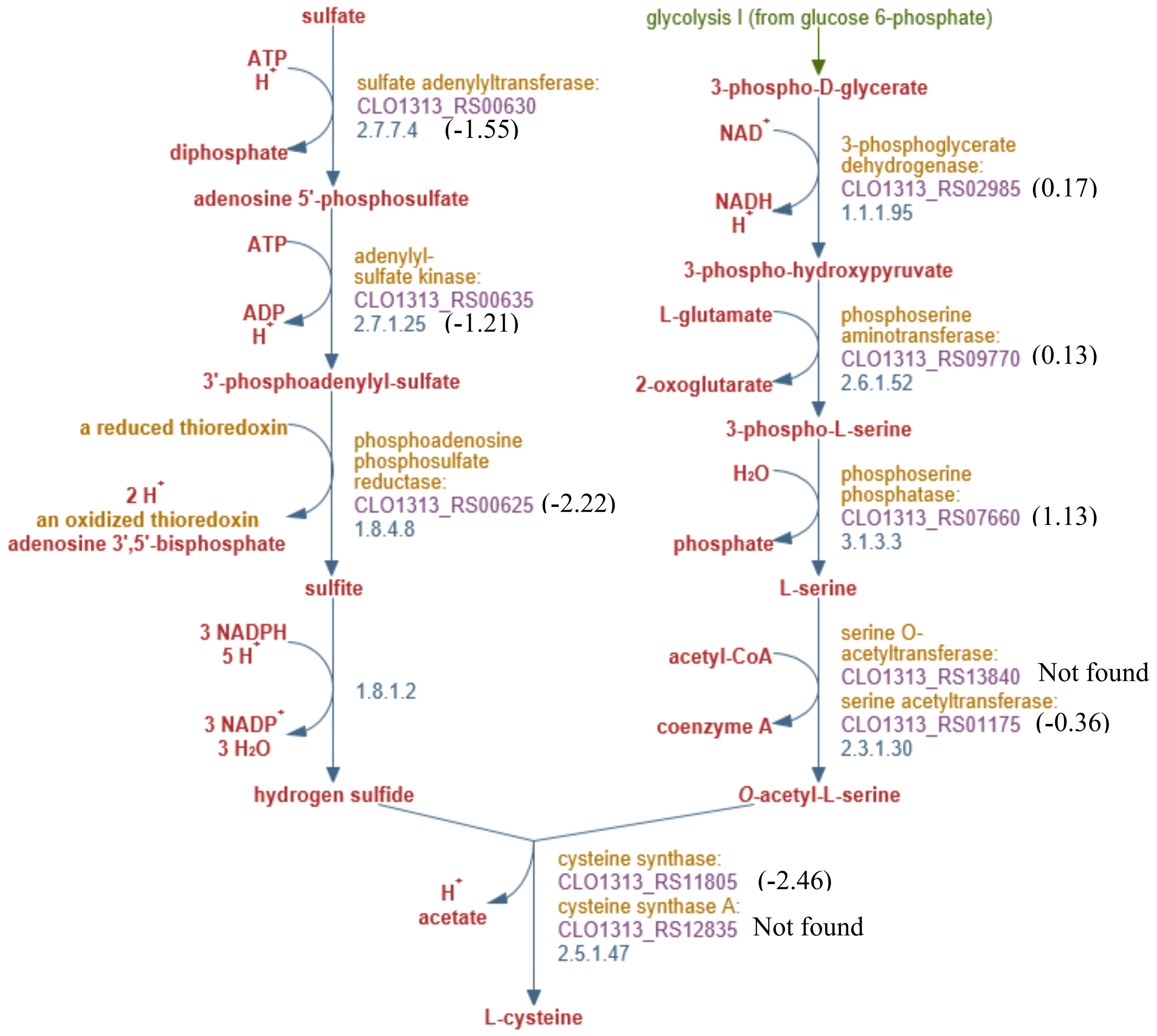

Supplemental Figure S4. Superpathway of sulfate assimilation and cysteine biosynthesis. The pathway is extracted from Biocyc with modifications. The numbers in parenthesis represent log2 fold changes. The gene locus for each enzyme annotated in the genome is represented with CLO1313 followed by a RS number.

Supplemental Table S1. Log2 Fold Change obtained for KJC335 growing on xylose.

Id	old locus tag	Fold Change	log2 Fold Change	pvalue	padj	product
acpP		0.703	-0.509	1.55E-12	8.14E-12	
addA		1.033	0.046	0.542539484	0.621970915	
addB		0.881	-0.183	0.010757456	0.019963868	
ahpC		0.974	-0.038	0.726224939	0.780067547	
alr		1.046	0.065	0.39239196	0.478756504	
amrS		1.018	0.026	0.782711691	0.827658809	
argB		0.287	-1.799	8.14E-28	1.00E-26	
argF		0.341	-1.552	4.71E-35	7.85E-34	
argH		0.62	-0.689	3.76E-10	1.63E-09	
aroA		1.087	0.12	0.120507245	0.173417097	
aroF		0.896	-0.158	0.069684569	0.107890563	
aroH		0.899	-0.153	0.054603503	0.087196968	
aroQ		1.14	0.189	0.059310353	0.093764759	
arsB		1.024	0.034	0.804090567	0.844055867	
asnB		2.109	1.077	6.25E-13	3.40E-12	
atpC		1.669	0.739	1.02E-08	3.87E-08	
atpD		1.734	0.794	9.09E-09	3.47E-08	
atpE		1.287	0.364	0.005932908	0.011523829	
atpF		1.555	0.637	1.36E-07	4.64E-07	
bioA		1.193	0.254	0.059945819	0.094619743	
bioC		1.277	0.353	0.03805906	0.062990684	
bioD		1.179	0.238	0.273098594	0.354401854	
bioF		1.23	0.299	0.11350819	0.164291053	
cadA		2.902	1.537	1.55E-05	4.23E-05	
cas2		1.98	0.985	3.12E-09	1.25E-08	
cas4		2.39	1.257	2.18E-19	1.76E-18	

cas6		0.405	-1.305	7.30E-10	3.10E-09	
cas7i		0.507	-0.98	4.46E-14	2.68E-13	
cbiQ		1.261	0.335	0.000573013	0.001265439	
cdd		1.048	0.068	0.307779052	0.390617595	
CLO1313_RS00010	Clo1313_0001	0.887	-0.173	0.084299727	0.127170463	chromosomal replication initiator protein DnaA
CLO1313_RS00015	Clo1313_0002	0.818	-0.29	0.002768922	0.005648977	DNA polymerase III subunit beta
CLO1313_RS00020	Clo1313_0003	0.819	-0.289	0.008131518	0.015454007	RNA-binding protein S4
CLO1313_RS00025	Clo1313_0004	0.823	-0.281	0.004908362	0.009670442	DNA recombination protein RecF
CLO1313_RS00030	Clo1313_0005	0.618	-0.694	7.97E-11	3.72E-10	hypothetical protein
CLO1313_RS00040	Clo1313_0007	1.046	0.065	0.450356035	0.534833467	sporulation initiation inhibitor Soj
CLO1313_RS00045	Clo1313_0008	1.01	0.015	0.827201558	0.860503822	chromosome partitioning protein ParB
CLO1313_RS00050	Clo1313_0009	0.96	-0.059	0.471656956	0.55318702	hypothetical protein
CLO1313_RS00055	Clo1313_0010	0.956	-0.064	0.476198834	0.55746437	hypothetical protein
CLO1313_RS00060	Clo1313_0011	1.105	0.143	0.020619776	0.035973653	serine--tRNA ligase
CLO1313_RS00065	Clo1313_R0001	0.954	-0.068	0.416298352	0.501397091	
CLO1313_RS00070	Clo1313_R0002	0.671	-0.576	6.00E-06	1.74E-05	
CLO1313_RS00075	Clo1313_0012	0.722	-0.47	0.000554636	0.001231202	MFS transporter
CLO1313_RS00080	Clo1313_0013	0.472	-1.083	1.58E-37	2.96E-36	copper amine oxidase
CLO1313_RS00085	Clo1313_0014	0.692	-0.531	2.63E-07	8.77E-07	hypothetical protein
CLO1313_RS00090	Clo1313_0015	0.581	-0.784	0.300580983	0.383103429	membrane protein
CLO1313_RS00095	Clo1313_0016	0.994	-0.009	0.922074622	0.938026387	vanomycin resistance protein VanB
CLO1313_RS00100	Clo1313_0017	1.234	0.304	0.087821606	0.131425647	peptidase M14
CLO1313_RS00105	Clo1313_0018	0.237	-2.08	0.081114497	0.123170823	hypothetical protein
CLO1313_RS00110	Clo1313_0019	1.724	0.786	0.085596503	0.12873817	pro-sigmaK processing inhibitor BofA
CLO1313_RS00115	Clo1313_0020	0.215	-2.218	2.71E-166	5.81E-164	pyruvate synthase
CLO1313_RS00120	Clo1313_0021	0.207	-2.27	1.37E-184	1.03E-181	ferredoxin
CLO1313_RS00130	Clo1313_0023	0.221	-2.18	5.60E-140	9.33E-138	thiamine pyrophosphate protein TPP-binding
CLO1313_RS00135	Clo1313_0025	0.44	-1.184	7.04E-07	2.27E-06	hypothetical protein
CLO1313_RS00140	Clo1313_0026	0.892	-0.166	0.827522992	0.860520615	XRE family transcriptional regulator
CLO1313_RS00145	Clo1313_0027	0.792	-0.337	0.45354576	0.537834612	hypothetical protein

CLO1313_RS00150	Clo1313_0028	1.666	0.736	0.055060169	0.087879429	glycosyl transferase family 39
CLO1313_RS00155	Clo1313_0029	0.91	-0.136	0.432670314	0.517788616	spore coat protein
CLO1313_RS00160	Clo1313_0030	0.969	-0.046	0.522936102	0.602260126	formate--tetrahydrofolate ligase
CLO1313_RS00170	Clo1313_0032	1.795	0.844	4.27E-24	4.35E-23	DUF1021 domain-containing protein
CLO1313_RS00175	Clo1313_0033	1.201	0.265	0.043880514	0.071629081	peptidoglycan-binding protein LysM
CLO1313_RS00180	Clo1313_0034	0.641	-0.643	6.78E-12	3.43E-11	4-diphosphocytidyl-2C-methyl-D-erythritol kinase
CLO1313_RS00185	Clo1313_0035	0.615	-0.701	5.98E-17	4.21E-16	GntR family transcriptional regulator
CLO1313_RS00190	Clo1313_0036	0.938	-0.093	0.297848871	0.380591719	heavy metal transporter
CLO1313_RS00195	Clo1313_0037	0.539	-0.893	6.99E-19	5.52E-18	hypothetical protein
CLO1313_RS00200	Clo1313_0038	0.712	-0.49	9.04E-14	5.28E-13	ATPase AAA
CLO1313_RS00205	Clo1313_0039	0.868	-0.205	0.006847818	0.01316449	phage-shock protein
CLO1313_RS00210	Clo1313_0040	0.658	-0.604	2.43E-13	1.37E-12	hypothetical protein
CLO1313_RS00215	Clo1313_0041	0.944	-0.083	0.262412439	0.343357289	hypothetical protein
CLO1313_RS00220	Clo1313_0042	1.075	0.104	0.183638806	0.250105712	metallophosphoesterase
CLO1313_RS00225	Clo1313_0043	0.934	-0.099	0.251718076	0.330228569	chromosome segregation protein SMC
CLO1313_RS00230	Clo1313_0044	0.964	-0.053	0.644733982	0.712963574	peptidase P60
CLO1313_RS00255	Clo1313_0048	1.198	0.26	0.000476904	0.001070536	abortive infection protein
CLO1313_RS00260	Clo1313_0049	0.914	-0.13	0.090880077	0.135260223	DNA replication protein DnaC
CLO1313_RS00270	Clo1313_R0003	0.516	-0.955	0.000170929	0.000411739	
CLO1313_RS00275	Clo1313_0051	0.857	-0.223	0.088025748	0.131471144	phosphohydrolase
CLO1313_RS00280	Clo1313_0052	0.85	-0.235	0.063543911	0.099357763	hypothetical protein
CLO1313_RS00285	Clo1313_0053	0.803	-0.317	2.48E-05	6.60E-05	hypothetical protein
CLO1313_RS00290	Clo1313_0054	0.845	-0.242	0.00269874	0.005528362	type 3a cellulose-binding domain protein
CLO1313_RS00295	Clo1313_0055	0.891	-0.166	0.06105142	0.0961624	copper amine oxidase
CLO1313_RS00300	Clo1313_0056	10.476	3.389	8.84E-133	1.33E-130	flagellar motor protein MotP
CLO1313_RS00305	Clo1313_0057	11.656	3.543	8.57E-125	1.17E-122	membrane protein
CLO1313_RS00310	Clo1313_0058	1.123	0.167	0.01724566	0.03073068	membrane protein
CLO1313_RS00325	Clo1313_0061	0.83	-0.269	0.047841031	0.077321162	electron transporter RnfC
CLO1313_RS00330	Clo1313_0062	0.877	-0.189	0.076621203	0.117238259	NADH:ubiquinone oxidoreductase
CLO1313_RS00335	Clo1313_0063	0.879	-0.186	0.055838326	0.088979351	electron transporter RnfG

CLO1313_RS00340	Clo1313_0064	0.94	-0.09	0.304701572	0.387367535	electron transporter RsxE
CLO1313_RS00345	Clo1313_0065	0.943	-0.084	0.343008773	0.428261162	electron transporter RsxE
CLO1313_RS00350	Clo1313_0066	0.945	-0.082	0.45219305	0.536442625	ferredoxin
CLO1313_RS00355	Clo1313_0067	0.941	-0.087	0.662485286	0.72776314	hypothetical protein
CLO1313_RS00360	Clo1313_0068	0.699	-0.517	0.109764141	0.159487722	shikimate kinase
CLO1313_RS00365	Clo1313_0070	0.84	-0.251	0.028419186	0.048288464	GCN5 family N-acetyltransferase
CLO1313_RS00370	Clo1313_0071	0.726	-0.461	4.75E-07	1.55E-06	ATP synthase F1 subunit delta
CLO1313_RS00375	Clo1313_0072	1.028	0.04	0.615157217	0.686841584	alkaline phosphatase
CLO1313_RS00380	Clo1313_0073	4.671	2.224	6.16E-46	1.63E-44	carbohydrate kinase
CLO1313_RS00385	Clo1313_0074	5.476	2.453	1.15E-49	3.44E-48	transketolase
CLO1313_RS00390	Clo1313_0075	5.533	2.468	2.47E-39	5.14E-38	transketolase
CLO1313_RS00395	Clo1313_0076	5.74	2.521	5.32E-55	1.90E-53	sorbitol dehydrogenase
CLO1313_RS00400	Clo1313_0077	4.387	2.133	7.98E-46	2.10E-44	sugar ABC transporter substrate-binding protein
CLO1313_RS00405	Clo1313_0078	3.818	1.933	2.38E-46	6.36E-45	ABC transporter
CLO1313_RS00410	Clo1313_0079	3.942	1.979	3.49E-55	1.26E-53	ABC transporter permease
CLO1313_RS00415	Clo1313_0080	4.293	2.102	1.99E-49	5.92E-48	phosphoglycerate mutase
CLO1313_RS00420		1.483	0.568	0.025387333	0.0436386	hypothetical protein
CLO1313_RS00425	Clo1313_0081	0.93	-0.105	0.199787795	0.268683227	helicase
CLO1313_RS00435	Clo1313_0083	0.96	-0.059	0.614203398	0.686032026	nucleotide pyrophosphohydrolase
CLO1313_RS00440	Clo1313_0084	13.235	3.726	2.25E-120	2.81E-118	ferrous iron transporter A
CLO1313_RS00445	Clo1313_R0004	0.307	-1.704	5.87E-18	4.41E-17	
CLO1313_RS00450	Clo1313_0085	0.635	-0.655	3.49E-15	2.28E-14	adenosine deaminase
CLO1313_RS00455	Clo1313_R0005	0.368	-1.441	9.06E-28	1.11E-26	
CLO1313_RS00460		0.592	-0.757	0.026663224	0.04561495	methionyl-tRNA synthetase
CLO1313_RS00465	Clo1313_0087	1.164	0.219	0.249986483	0.328292297	cupin
CLO1313_RS00470	Clo1313_0088	0.988	-0.017	0.865812033	0.893520402	hypothetical protein
CLO1313_RS00475	Clo1313_0089	1.063	0.088	0.267489998	0.34848067	LacI family transcriptional regulator
CLO1313_RS00490	Clo1313_0091	2.757	1.463	0.021755593	0.037867107	peptidase U57
CLO1313_RS00495	Clo1313_0092	0.639	-0.646	8.13E-15	5.12E-14	DNA topoisomerase III
CLO1313_RS00500	Clo1313_0093	0.574	-0.8	1.19E-23	1.19E-22	hypothetical protein

CLO1313_RS00505	Clo1313_0094	0.945	-0.081	0.476770133	0.557657421	mechanosensitive ion channel protein
CLO1313_RS00510	Clo1313_0095	1.287	0.364	0.037230957	0.061790615	hypothetical protein
CLO1313_RS00515	Clo1313_0096	1.615	0.692	1.79E-06	5.50E-06	peptidase S1
CLO1313_RS00520	Clo1313_0097	1.953	0.966	2.16E-08	7.88E-08	hypothetical protein
CLO1313_RS00525	Clo1313_0098	0.88	-0.185	0.008095625	0.015395549	cytosine deaminase
CLO1313_RS00535	Clo1313_0100	0.894	-0.161	0.115807662	0.167376954	acetolactate synthase
CLO1313_RS00540	Clo1313_0101	0.938	-0.093	0.34192893	0.427446795	ketol-acid reductoisomerase
CLO1313_RS00545	Clo1313_0102	0.911	-0.135	0.253268049	0.332116693	citramalate synthase
CLO1313_RS00550	Clo1313_0103	0.975	-0.036	0.733727217	0.787280116	hypothetical protein
CLO1313_RS00560	Clo1313_0105	1.109	0.149	0.297989891	0.380609746	membrane protein
CLO1313_RS00565	Clo1313_0106	0.892	-0.166	0.594732416	0.669699706	hypothetical protein
CLO1313_RS00570	Clo1313_0107	0.346	-1.53	1.48E-12	7.79E-12	Rrf2 family transcriptional regulator
CLO1313_RS00580	Clo1313_0109	0.475	-1.074	0.12819085	0.182894557	precorrin-2 oxidase
CLO1313_RS00585	Clo1313_0110	0.66	-0.599	0.122262538	0.175270245	porphobilinogen deaminase
CLO1313_RS00595	Clo1313_0112	1.059	0.083	0.717356965	0.771924485	delta-aminolevulinic acid dehydratase
CLO1313_RS00605	Clo1313_0114	0.145	-2.79	1.72E-40	3.73E-39	sulfate transporter subunit
CLO1313_RS00620	Clo1313_0117	0.213	-2.228	9.68E-24	9.64E-23	sulfate ABC transporter ATP-binding protein
CLO1313_RS00625	Clo1313_0118	0.261	-1.94	3.13E-10	1.37E-09	phosphoadenosine phosphosulfate reductase
CLO1313_RS00630	Clo1313_0119	0.341	-1.551	1.03E-09	4.30E-09	sulfate adenylyltransferase
CLO1313_RS00635	Clo1313_0120	0.43	-1.217	6.62E-13	3.59E-12	adenylylsulfate kinase
CLO1313_RS00645	Clo1313_0122	0.439	-1.189	1.68E-08	6.25E-08	adenylyltransferase
CLO1313_RS00650	Clo1313_0123	0.29	-1.787	7.72E-09	2.97E-08	Mov34/MPN/PAD-1 family protein
CLO1313_RS00655	Clo1313_0124	0.596	-0.747	2.34E-05	6.24E-05	nitrite and sulphite reductase 4Fe-4S region
CLO1313_RS00660	Clo1313_0125	1.251	0.323	0.070363244	0.108828968	transcriptional regulator
CLO1313_RS00665	Clo1313_0126	3.171	1.665	3.00E-85	2.10E-83	sporulation protein
CLO1313_RS00670	Clo1313_0127	2.801	1.486	2.04E-35	3.48E-34	RNA polymerase subunit sigma-24
CLO1313_RS00675	Clo1313_0128	3.429	1.778	0.253490165	0.332262677	histidine kinase
CLO1313_RS00680	Clo1313_0129	1.577	0.657	0.365067688	0.451852248	histidine kinase
CLO1313_RS00685		0.274	-1.87	0.219618274	0.292466787	transcriptional regulator
CLO1313_RS00690	Clo1313_0131	0.757	-0.402	0.000104613	0.000258004	hypothetical protein

CLO1313_RS00695	Clo1313_0132	0.683	-0.549	8.75E-09	3.34E-08	hypothetical protein
CLO1313_RS00700	Clo1313_0133	0.908	-0.139	0.081382041	0.123452069	Intracellular exo-alpha-(1->5)-L-arabinofuranosidase
CLO1313_RS00705	Clo1313_0135	1.097	0.133	0.386605572	0.472658015	dockerin
CLO1313_RS00710	Clo1313_0136	1.401	0.487	0.127823605	0.182544282	glycosyl transferase
CLO1313_RS00715	Clo1313_0137	1.039	0.055	0.827219913	0.860503822	radical SAM protein
CLO1313_RS00720	Clo1313_0138	1.404	0.49	0.027547553	0.04696709	radical SAM protein
CLO1313_RS00725	Clo1313_0139	1.72	0.782	2.61E-06	7.86E-06	radical SAM protein
CLO1313_RS00730	Clo1313_0140	1.213	0.278	0.118522216	0.171052996	glycosyl transferase family 1
CLO1313_RS00735	Clo1313_0141	1.358	0.441	0.043230827	0.070769241	FkbM family methyltransferase
CLO1313_RS00740	Clo1313_0142	1.568	0.648	4.90E-06	1.44E-05	radical SAM protein
CLO1313_RS00745	Clo1313_0143	1.794	0.843	7.58E-06	2.16E-05	glycosyl transferase
CLO1313_RS00750	Clo1313_0144	8.11	3.02	5.49E-25	5.86E-24	NAD-dependent dehydratase
CLO1313_RS00755	Clo1313_0145	1.058	0.081	0.399498686	0.485846131	LPS biosynthesis protein
CLO1313_RS00775	Clo1313_0149	1.078	0.108	0.378653546	0.465021288	hypothetical protein
CLO1313_RS00780	Clo1313_0150	1.658	0.729	2.58E-10	1.15E-09	transposase
CLO1313_RS00785	Clo1313_0151	1.01	0.014	0.950352704	0.960278895	hypothetical protein
CLO1313_RS00790	Clo1313_0152	1.002	0.003	0.985501948	0.988468342	radical SAM protein
CLO1313_RS00795	Clo1313_0153	0.823	-0.28	0.630763733	0.700736928	cupin
CLO1313_RS00800	Clo1313_0154	1.683	0.751	0.02767416	0.047156139	hypothetical protein
CLO1313_RS00805	Clo1313_0155	1.688	0.755	0.156687376	0.217548815	hypothetical protein
CLO1313_RS00810	Clo1313_0156	0.411	-1.283	0.102467464	0.150268912	hypothetical protein
CLO1313_RS00815	Clo1313_0157	1.062	0.087	0.382959567	0.468773772	hypothetical protein
CLO1313_RS00820		0.717	-0.479	0.021794045	0.037912031	hypothetical protein
CLO1313_RS00825	Clo1313_0159	0.645	-0.633	5.60E-08	1.98E-07	hypothetical protein
CLO1313_RS00830	Clo1313_0160	1.682	0.75	0.266222952	0.347736338	spermidine/putrescine ABC transporter ATP-binding protein
CLO1313_RS00835	Clo1313_0161	0.758	-0.4	0.713403592	0.76849762	sulfonate ABC transporter permease
CLO1313_RS00840	Clo1313_0162	1.765	0.82	0.004142434	0.00824911	hypothetical protein
CLO1313_RS00845	Clo1313_0163	0.854	-0.228	0.003018768	0.00612951	hypothetical protein
CLO1313_RS00850	Clo1313_0164	1.138	0.187	0.056890117	0.090415189	adenylate cyclase
CLO1313_RS00855	Clo1313_0165	1.37	0.454	1.46E-08	5.47E-08	exopolyphosphatase

CLO1313_RS00860	Clo1313_0166	0.865	-0.21	0.004559536	0.009031736	alcohol dehydrogenase
CLO1313_RS00865	Clo1313_0167	0.828	-0.273	0.000730062	0.001596978	phosphodiesterase
CLO1313_RS00885	Clo1313_0171	0.977	-0.034	0.657388908	0.724021056	hypothetical protein
CLO1313_RS00890	Clo1313_0172	1.183	0.242	0.001514916	0.003190474	bifunctional biotin--[acetyl-CoA-carboxylase] synthetase/biotin operon repressor
CLO1313_RS00895	Clo1313_0173	1.381	0.466	3.95E-10	1.71E-09	amidohydrolase
CLO1313_RS00900	Clo1313_0174	1.407	0.492	1.65E-12	8.62E-12	hypothetical protein
CLO1313_RS00905	Clo1313_0175	1.319	0.399	1.64E-08	6.09E-08	type III pantothenate kinase
CLO1313_RS00910	Clo1313_0176	0.716	-0.483	2.08E-08	7.60E-08	hypothetical protein
CLO1313_RS00915	Clo1313_0177	0.935	-0.097	0.50654364	0.585404384	glycoside hydrolase
CLO1313_RS00920	Clo1313_0178	1.047	0.067	0.367259868	0.454190658	hypothetical protein
CLO1313_RS00925	Clo1313_0179	1.016	0.022	0.815301845	0.853434636	hypothetical protein
CLO1313_RS00930	Clo1313_0180	0.75	-0.415	2.59E-11	1.25E-10	peptide chain release factor 1
CLO1313_RS00935	Clo1313_0181	0.799	-0.323	1.71E-05	4.66E-05	zinc permease
CLO1313_RS00940	Clo1313_0182	0.695	-0.524	1.46E-16	1.01E-15	tRNA threonylcarbamoyladenosine biosynthesis protein
CLO1313_RS00945	Clo1313_0183	0.913	-0.131	0.061240809	0.096359488	protein-tyrosine phosphatase
CLO1313_RS00955	Clo1313_0185	1.229	0.297	1.61E-06	4.95E-06	uracil phosphoribosyltransferase
CLO1313_RS00960	Clo1313_0186	1.215	0.281	2.78E-05	7.35E-05	cytidine deaminase
CLO1313_RS00965	Clo1313_0187	1.251	0.323	1.53E-06	4.74E-06	glycosyl transferase
CLO1313_RS00970	Clo1313_0188	1.192	0.253	0.001841782	0.003841101	UDP-N-acetylglucosamine 2-epimerase
CLO1313_RS00975	Clo1313_0189	1.46	0.546	7.69E-10	3.27E-09	F0F1 ATP synthase subunit A
CLO1313_RS00990	Clo1313_0192	1.441	0.527	4.08E-06	1.21E-05	ATP synthase F1 subunit delta
CLO1313_RS00995	Clo1313_0193	1.443	0.529	0.000159462	0.000385045	F0F1 ATP synthase subunit alpha
CLO1313_RS01000	Clo1313_0194	1.602	0.68	6.20E-07	2.01E-06	ATP synthase subunit gamma
CLO1313_RS01015	Clo1313_0197	1.038	0.054	0.732407741	0.786145604	hypothetical protein
CLO1313_RS01020	Clo1313_0198	2.45	1.293	3.10E-24	3.17E-23	ferrous iron transporter A
CLO1313_RS01025	Clo1313_0199	3.67	1.876	1.59E-36	2.86E-35	ferrous iron transporter A
CLO1313_RS01030	Clo1313_0200	1.494	0.579	1.02E-10	4.71E-10	hypothetical protein
CLO1313_RS01035	Clo1313_0201	0.956	-0.065	0.471200808	0.553084627	hypothetical protein
CLO1313_RS01050	Clo1313_0204	2.008	1.006	6.71E-13	3.63E-12	peptidase M23

CLO1313_RS01060	Clo1313_0206	0.841	-0.25	8.33E-05	0.000208894	rod shape-determining protein Mbl
CLO1313_RS01065	Clo1313_0207	5.403	2.434	5.61E-62	2.55E-60	flagellar basal body rod protein FlgG
CLO1313_RS01075	Clo1313_0209	1.751	0.808	1.49E-19	1.21E-18	flagellar protein FlgJ
CLO1313_RS01080	Clo1313_0210	1.406	0.491	1.10E-05	3.05E-05	exopolysaccharide biosynthesis protein
CLO1313_RS01085	Clo1313_0211	1.461	0.547	0.009306045	0.017453926	ABC transporter ATP-binding protein
CLO1313_RS01095	Clo1313_0214	0.806	-0.311	3.57E-06	1.06E-05	UDP-N-acetylmuramate--L-alanine ligase
CLO1313_RS01100	Clo1313_0215	1.07	0.098	0.19009285	0.258075355	LacI family transcriptional regulator
CLO1313_RS01105	Clo1313_0216	1.06	0.085	0.277528722	0.359063261	hypothetical protein
CLO1313_RS01115	Clo1313_0218	0.679	-0.559	6.30E-12	3.20E-11	ribose-phosphate pyrophosphokinase
CLO1313_RS01120	Clo1313_0219	0.85	-0.235	0.031349279	0.052937211	peptidyl-tRNA hydrolase
CLO1313_RS01130	Clo1313_0221	0.73	-0.454	8.62E-09	3.30E-08	peptidyl-prolyl cis-trans isomerase
CLO1313_RS01135	Clo1313_0223	0.972	-0.041	0.743700342	0.794286797	AraC family transcriptional regulator
CLO1313_RS01140	Clo1313_0224	1.043	0.06	0.824239449	0.859490301	pyridoxamine 5'-phosphate oxidase
CLO1313_RS01150	Clo1313_0226	1.558	0.64	0.193529575	0.261439277	glycosyl transferase family 1
CLO1313_RS01155	Clo1313_0227	1.011	0.016	0.956595485	0.964636806	polymerase
CLO1313_RS01160	Clo1313_0228	1.035	0.05	0.928195221	0.94266109	hypothetical protein
CLO1313_RS01175	Clo1313_0231	0.809	-0.306	0.752918242	0.802132081	serine acetyltransferase
CLO1313_RS01180	Clo1313_0232	1.776	0.828	0.28826742	0.371036049	nucleotidyltransferase
CLO1313_RS01185	Clo1313_0233	1.562	0.644	0.340032139	0.425430282	aminotransferase DegT
CLO1313_RS01190	Clo1313_0234	1.535	0.618	0.294721806	0.378116113	NAD-dependent dehydratase
CLO1313_RS01195	Clo1313_0235	2.042	1.03	0.05182739	0.083296004	capsular polysaccharide biosynthesis protein
CLO1313_RS01200	Clo1313_0236	1.002	0.003	0.99600069	0.996998021	carboxylate-amine ligase
CLO1313_RS01205	Clo1313_0237	1.771	0.825	0.308568766	0.3914542	sugar transferase
CLO1313_RS01210	Clo1313_0238	0.855	-0.226	0.700471391	0.759202639	aldolase
CLO1313_RS01215	Clo1313_0239	0.952	-0.071	0.82699567	0.860503822	nucleoside-diphosphate sugar epimerase
CLO1313_RS01220	Clo1313_0240	0.719	-0.476	0.359427169	0.44579077	hypothetical protein
CLO1313_RS01225	Clo1313_0241	0.621	-0.687	0.131025265	0.186406437	LPS biosynthesis protein
CLO1313_RS01230	Clo1313_0242	1.046	0.065	0.850223943	0.880159339	capsular polysaccharide biosynthesis protein
CLO1313_RS01235	Clo1313_0243	0.878	-0.188	0.596027457	0.670223601	phosphoesterase
CLO1313_RS01245	Clo1313_0245	0.74	-0.435	2.80E-08	1.01E-07	nucleoside triphosphate pyrophosphohydrolase

CLO1313_RS01250	Clo1313_0246	0.772	-0.372	0.000196058	0.00046702	transcriptional regulator
CLO1313_RS01255	Clo1313_0247	0.804	-0.315	0.004880199	0.00962876	hypothetical protein
CLO1313_RS01270	Clo1313_0250	0.979	-0.03	0.722145256	0.776798287	septation inhibitor protein
CLO1313_RS01275	Clo1313_0251	0.803	-0.316	8.53E-06	2.42E-05	RNA-binding protein S1
CLO1313_RS01280	Clo1313_R0006	0.718	-0.479	6.42E-07	2.08E-06	
CLO1313_RS01285	Clo1313_R0007	0.625	-0.679	1.99E-09	8.09E-09	
CLO1313_RS01290	Clo1313_0252	0.791	-0.338	0.012878712	0.023479185	chemotaxis protein
CLO1313_RS01295	Clo1313_0253	1.186	0.246	0.004781616	0.009444991	2-octaprenylphenol hydroxylase
CLO1313_RS01300	Clo1313_0254	1.024	0.034	0.8021429	0.842601246	Zn-finger containing protein
CLO1313_RS01305	Clo1313_0255	0.665	-0.588	9.58E-05	0.000238937	ATPase
CLO1313_RS01310	Clo1313_0256	0.931	-0.103	0.568045878	0.644315276	TetR family transcriptional regulator
CLO1313_RS01315		1.08	0.111	0.620843389	0.692224975	transglutaminase
CLO1313_RS01320		0.987	-0.019	0.920663422	0.93701771	transglutaminase
CLO1313_RS01325		1.202	0.266	0.129057994	0.183956712	hypothetical protein
CLO1313_RS01330	Clo1313_0258	1.222	0.289	0.103264863	0.15129034	hypothetical protein
CLO1313_RS01335		1.413	0.499	0.002761304	0.00563727	
CLO1313_RS01340	Clo1313_0260	1.29	0.367	0.073509548	0.112880253	hydrolase
CLO1313_RS01345	Clo1313_0262	1.325	0.406	0.048122482	0.077591035	50S ribosomal protein L7/L12
CLO1313_RS01350		1.389	0.474	0.072561116	0.111709849	hypothetical protein
CLO1313_RS01355	Clo1313_0264	1.218	0.284	0.16438781	0.227607823	hypothetical protein
CLO1313_RS01360	Clo1313_0265	1.033	0.046	0.629039217	0.699476683	hypothetical protein
CLO1313_RS01365	Clo1313_0266	1.43	0.516	7.42E-11	3.47E-10	GumN family protein
CLO1313_RS01375	Clo1313_0268	1.502	0.587	1.47E-06	4.56E-06	hypothetical protein
CLO1313_RS01380	Clo1313_0269	1.47	0.556	4.75E-06	1.39E-05	hypothetical protein
CLO1313_RS01385	Clo1313_0270	0.829	-0.271	0.00772203	0.014731786	peptidase S41
CLO1313_RS01390	Clo1313_R0008	0.454	-1.14	1.10E-16	7.67E-16	
CLO1313_RS01395	Clo1313_R0009	0.419	-1.255	4.38E-21	3.86E-20	
CLO1313_RS01400	Clo1313_R0010	0.32	-1.643	1.17E-27	1.42E-26	
CLO1313_RS01410	Clo1313_0272	1.783	0.834	1.33E-10	6.11E-10	hypothetical protein
CLO1313_RS01415	Clo1313_0273	1.523	0.607	1.01E-12	5.38E-12	hypothetical protein

CLO1313_RS01420	Clo1313_R0011	1.155	0.208	0.059671983	0.094286763	
CLO1313_RS01425	Clo1313_0274	1.433	0.519	7.40E-07	2.37E-06	pilus assembly protein PilZ
CLO1313_RS01430	Clo1313_0275	0.999	-0.002	0.980663308	0.984932773	hypothetical protein
CLO1313_RS01435	Clo1313_0276	1.028	0.04	0.572643499	0.647815108	pilus assembly protein PilM
CLO1313_RS01440	Clo1313_0277	0.724	-0.466	0.000452616	0.001023677	thioesterase
CLO1313_RS01450	Clo1313_0280	1.169	0.225	0.008240331	0.015650888	GntR family transcriptional regulator
CLO1313_RS01455	Clo1313_0281	0.716	-0.481	1.88E-05	5.08E-05	decaprenyl-phosphate phosphoribosyltransferase
CLO1313_RS01460	Clo1313_0282	0.73	-0.454	2.68E-06	8.03E-06	acetyltransferase
CLO1313_RS01465	Clo1313_0283	0.799	-0.324	2.79E-05	7.36E-05	glycosyl transferase family 1
CLO1313_RS01470	Clo1313_0284	0.879	-0.187	0.011202951	0.020726497	hypothetical protein
CLO1313_RS01475	Clo1313_0285	0.851	-0.233	0.002961387	0.006021152	polymerase
CLO1313_RS01480	Clo1313_0286	0.96	-0.059	0.459110275	0.542075478	hypothetical protein
CLO1313_RS01485	Clo1313_0287	1.051	0.071	0.346256871	0.431777279	carbohydrate kinase
CLO1313_RS01495	Clo1313_0289	1.339	0.421	1.57E-05	4.28E-05	CopG family transcriptional regulator
CLO1313_RS01500	Clo1313_0290	1.54	0.623	1.45E-10	6.64E-10	mRNA interferase PemK
CLO1313_RS01505	Clo1313_0291	1.869	0.902	9.56E-23	9.10E-22	hypothetical protein
CLO1313_RS01510	Clo1313_0292	0.733	-0.448	6.86E-06	1.97E-05	N-acetylmannosaminyltransferase
CLO1313_RS01520	Clo1313_0294	1.169	0.226	0.104060999	0.152188419	hypothetical protein
CLO1313_RS01525	Clo1313_0295	0.908	-0.14	0.122162725	0.175253402	transketolase
CLO1313_RS01530	Clo1313_0296	0.97	-0.044	0.549776761	0.628585782	transketolase
CLO1313_RS01535	Clo1313_0297	0.714	-0.485	6.40E-08	2.24E-07	ABC transporter
CLO1313_RS01540	Clo1313_0298	0.675	-0.568	4.78E-10	2.05E-09	ABC transporter permease
CLO1313_RS01545	Clo1313_0299	0.709	-0.496	3.04E-10	1.34E-09	ABC transporter
CLO1313_RS01550	Clo1313_0300	0.742	-0.431	6.73E-09	2.60E-08	hypothetical protein
CLO1313_RS01555	Clo1313_0301	1.239	0.309	0.08211326	0.124309776	membrane protein
CLO1313_RS01560	Clo1313_0302	0.887	-0.173	0.03305656	0.055538724	hypothetical protein
CLO1313_RS01565	Clo1313_0303	0.702	-0.511	6.96E-12	3.51E-11	colicin V production protein
CLO1313_RS01590	Clo1313_0308	0.522	-0.937	3.68E-54	1.28E-52	transcription termination/antitermination factor NusG
CLO1313_RS01600	Clo1313_0310	0.486	-1.042	7.65E-33	1.15E-31	50S ribosomal protein L1
CLO1313_RS01605	Clo1313_0311	0.416	-1.267	6.33E-62	2.83E-60	50S ribosomal protein L10

CLO1313_RS01610	Clo1313_0312	0.433	-1.208	1.08E-66	5.47E-65	50S ribosomal protein L7/L12
CLO1313_RS01625	Clo1313_0315	0.585	-0.774	8.17E-21	7.04E-20	50S ribosomal protein L7ae
CLO1313_RS01630	Clo1313_0316	0.495	-1.015	3.23E-45	8.00E-44	30S ribosomal protein S12
CLO1313_RS01635	Clo1313_0317	0.47	-1.089	4.93E-50	1.49E-48	30S ribosomal protein S7
CLO1313_RS01650	Clo1313_0320	0.981	-0.028	0.797392381	0.839964788	RNA polymerase subunit sigma-24
CLO1313_RS01655	Clo1313_0321	1.064	0.09	0.353310224	0.4394763	hypothetical protein
CLO1313_RS01660	Clo1313_0322	0.997	-0.005	0.984768352	0.988393671	hypothetical protein
CLO1313_RS01665	Clo1313_0323	1.15	0.202	0.196841177	0.265196177	vanomycin resistance protein VanB
CLO1313_RS01670	Clo1313_0324	0.936	-0.095	0.301260006	0.383805759	HPr kinase
CLO1313_RS01680	Clo1313_0326	1.221	0.288	0.001345891	0.002858589	UvrABC system protein C
CLO1313_RS01685	Clo1313_0327	1.352	0.435	5.63E-06	1.64E-05	ser/threonine protein phosphatase
CLO1313_RS01690	Clo1313_0328	0.606	-0.723	2.41E-26	2.74E-25	trigger factor
CLO1313_RS01700	Clo1313_0330	0.681	-0.555	9.49E-18	7.05E-17	ATP-dependent Clp protease ATP-binding subunit ClpX
CLO1313_RS01715	Clo1313_0333	3.162	1.661	1.79E-84	1.20E-82	lytic transglycosylase
CLO1313_RS01720	Clo1313_0334	0.691	-0.534	0.001793731	0.003748711	hypothetical protein
CLO1313_RS01725	Clo1313_0335	0.684	-0.548	7.95E-10	3.36E-09	hypothetical protein
CLO1313_RS01730	Clo1313_0336	0.684	-0.547	1.06E-08	4.00E-08	phosphoesterase
CLO1313_RS01735	Clo1313_0337	0.616	-0.7	4.06E-13	2.25E-12	SsrA-binding protein
CLO1313_RS01740	Clo1313_0338	0.376	-1.412	2.46E-26	2.79E-25	hypothetical protein
CLO1313_RS01745	Clo1313_0339	0.969	-0.046	0.768975932	0.816050538	hypothetical protein
CLO1313_RS01750	Clo1313_0341	1.092	0.127	0.305523984	0.388248487	MBL fold protein
CLO1313_RS01755	Clo1313_0342	0.969	-0.045	0.798971667	0.840447573	hypothetical protein
CLO1313_RS01760	Clo1313_0343	0.883	-0.18	0.111733623	0.162035365	MBL fold metallo-hydrolase
CLO1313_RS01765	Clo1313_0344	1.057	0.081	0.462388174	0.545087317	hypothetical protein
CLO1313_RS01770	Clo1313_0346	0.718	-0.477	7.27E-07	2.33E-06	potassium transporter CPA
CLO1313_RS01775	Clo1313_0348	1.026	0.037	0.751738526	0.801444663	AraC family transcriptional regulator
CLO1313_RS01780	Clo1313_0349	0.75	-0.414	0.000277473	0.000649096	glycoside hydrolase
CLO1313_RS01785	Clo1313_0350	0.697	-0.521	5.89E-07	1.92E-06	glycoside hydrolase
CLO1313_RS01790	Clo1313_0351	0.991	-0.013	0.893935731	0.915925267	transcriptional regulator
CLO1313_RS01795	Clo1313_0352	1.068	0.095	0.405901248	0.491440389	transcriptional regulator

CLO1313_RS01800	Clo1313_0353	1.555	0.637	7.93E-10	3.35E-09	RNA-binding protein
CLO1313_RS01805	Clo1313_0354	1.113	0.154	0.120032778	0.172900241	hypothetical protein
CLO1313_RS01810	Clo1313_0355	1.01	0.015	0.944643748	0.955476087	hypothetical protein
CLO1313_RS01815	Clo1313_0356	1.07	0.098	0.295709426	0.378826386	type 12 methyltransferase
CLO1313_RS01820	Clo1313_0357	1.187	0.248	0.008950388	0.016871284	polynucleotide kinase-phosphatase
CLO1313_RS01830	Clo1313_0359	1.82	0.864	1.50E-09	6.16E-09	flavoprotein
CLO1313_RS01835	Clo1313_0360	1.243	0.314	0.003597825	0.007226977	Crp/Fnr family transcriptional regulator
CLO1313_RS01840	Clo1313_0361	0.565	-0.823	0.003955984	0.007904061	hypothetical protein
CLO1313_RS01850	Clo1313_0364	0.872	-0.198	0.114755608	0.165936388	nicotinamide mononucleotide transporter
CLO1313_RS01855	Clo1313_0365	0.782	-0.355	0.001270669	0.002708412	cytidyltransferase
CLO1313_RS01860	Clo1313_0366	0.824	-0.279	0.094898632	0.140632784	hypothetical protein
CLO1313_RS01865	Clo1313_0367	0.758	-0.399	0.036885864	0.061319681	hypothetical protein
CLO1313_RS01870	Clo1313_0368	1.102	0.14	0.134458532	0.190387695	hypothetical protein
CLO1313_RS01875	Clo1313_0369	1.067	0.094	0.466137944	0.548775827	hypothetical protein
CLO1313_RS01880	Clo1313_0370	2.25	1.17	9.94E-05	0.00024677	N-acetylmuramoyl-L-alanine amidase
CLO1313_RS01885	Clo1313_0372	0.789	-0.343	0.682300629	0.744621393	cobalamin-binding protein
CLO1313_RS01890	Clo1313_0373	0.988	-0.018	0.973735236	0.979286376	methyltransferase MtaA/CmuA family
CLO1313_RS01895	Clo1313_0374	1.117	0.16	0.779212323	0.825453111	methylcobamide--CoM methyltransferase
CLO1313_RS01900	Clo1313_0375	1.361	0.445	0.284670298	0.367668485	ferredoxin
CLO1313_RS01905	Clo1313_0376	1.515	0.6	0.45047994	0.534833467	hypothetical protein
CLO1313_RS01910	Clo1313_0377	0.664	-0.59	0.20753806	0.278108419	metal ABC transporter substrate-binding protein
CLO1313_RS01915	Clo1313_0378	1.391	0.477	0.276953252	0.358644188	ABC transporter
CLO1313_RS01920	Clo1313_0379	1.349	0.432	0.29098531	0.374101684	ABC transporter permease
CLO1313_RS01925	Clo1313_0380	1.131	0.178	0.622006593	0.693198726	coenzyme F390 synthetase
CLO1313_RS01930	Clo1313_0381	2.81	1.491	1.04E-11	5.20E-11	aminotransferase
CLO1313_RS01935	Clo1313_0382	1.316	0.396	0.448254785	0.532613352	pyruvate/ketoisovalerate oxidoreductase gamma subunit
CLO1313_RS01940	Clo1313_0383	1.616	0.693	0.332169844	0.417159699	pyruvate ferredoxin/flavodoxin oxidoreductase delta subunit
CLO1313_RS01950	Clo1313_0385	0.328	-1.609	3.91E-07	1.29E-06	2-ketoisovalerate ferredoxin oxidoreductase subunit beta
CLO1313_RS01955	Clo1313_0386	0.317	-1.66	5.98E-17	4.21E-16	hypothetical protein
CLO1313_RS01960	Clo1313_0387	0.21	-2.249	1.54E-41	3.44E-40	reactive intermediate/imine deaminase

CLO1313_RS01965	Clo1313_0388	0.347	-1.528	1.94E-29	2.57E-28	cystathionine beta-lyase
CLO1313_RS01970	Clo1313_0389	0.413	-1.276	7.88E-25	8.29E-24	cystathionine beta-lyase
CLO1313_RS01980	Clo1313_0391	0.196	-2.351	3.01E-28	3.84E-27	metal ABC transporter substrate-binding protein
CLO1313_RS01985	Clo1313_0392	0.413	-1.277	6.99E-15	4.42E-14	sulfonate ABC transporter permease
CLO1313_RS01990	Clo1313_0393	0.424	-1.238	1.82E-14	1.12E-13	nitrate ABC transporter ATP-binding protein
CLO1313_RS01995	Clo1313_0394	0.328	-1.609	9.26E-45	2.24E-43	carbohydrate-binding protein
CLO1313_RS02000	Clo1313_0395	0.683	-0.551	6.90E-11	3.23E-10	endoglucanase
CLO1313_RS02005	Clo1313_0396	0.96	-0.059	0.435084861	0.5200556	LacI family transcriptional regulator
CLO1313_RS02010	Clo1313_0397	0.977	-0.034	0.612875351	0.685570003	glycoside hydrolase
CLO1313_RS02015	Clo1313_0398	0.867	-0.206	0.007475732	0.014307416	Na/Pi cotransporter
CLO1313_RS02020	Clo1313_0399	1.432	0.518	0.000464078	0.001044873	glycoside hydrolase
CLO1313_RS02025	Clo1313_0400	2.022	1.016	3.04E-08	1.10E-07	glycoside hydrolase
CLO1313_RS02030	Clo1313_0401	1.108	0.149	0.415256539	0.500947048	two-component system response regulator
CLO1313_RS02035	Clo1313_0402	1.02	0.029	0.782514306	0.827658809	two-component system sensor histidine kinase
CLO1313_RS02040	Clo1313_0403	0.573	-0.803	6.37E-37	1.18E-35	lysyl-tRNA synthetase
CLO1313_RS02045	Clo1313_0405	2.623	1.391	2.34E-22	2.20E-21	chemotaxis protein CheW
CLO1313_RS02050	Clo1313_0406	2.446	1.291	3.31E-25	3.58E-24	chemotaxis protein CheA
CLO1313_RS02055	Clo1313_0407	2.562	1.357	8.32E-44	1.95E-42	chemotaxis protein
CLO1313_RS02060	Clo1313_0408	2.079	1.056	1.27E-10	5.83E-10	chemotaxis protein CheR
CLO1313_RS02065	Clo1313_0409	1.978	0.984	3.51E-15	2.28E-14	two-component system protein-glutamate methylesterase response regulator
CLO1313_RS02070	Clo1313_0410	0.81	-0.304	0.000287523	0.000671037	oxidoreductase
CLO1313_RS02075	Clo1313_0411	0.685	-0.547	7.85E-13	4.22E-12	hypothetical protein
CLO1313_RS02080	Clo1313_0412	0.786	-0.348	0.033650988	0.056348025	TipAS antibiotic-recognition domain protein
CLO1313_RS02085	Clo1313_0413	0.743	-0.429	2.71E-07	9.01E-07	endoglucanase
CLO1313_RS02090	Clo1313_0414	0.861	-0.215	0.010350227	0.019255789	hypothetical protein
CLO1313_RS02095	Clo1313_0415	1.715	0.778	6.39E-14	3.77E-13	phosphoenolpyruvate carboxykinase
CLO1313_RS02110	Clo1313_0418	1.016	0.023	0.800418738	0.841084722	hypothetical protein
CLO1313_RS02115	Clo1313_0419	0.789	-0.343	0.000130254	0.00031707	peptidase C39
CLO1313_RS02120	Clo1313_0420	1.009	0.013	0.896649391	0.918391914	dockerin

CLO1313_RS02130	Clo1313_0422	1.194	0.256	0.07161667	0.110539575	ATP phosphoribosyltransferase
CLO1313_RS02145		0.789	-0.342	0.000298205	0.000693269	imidazoleglycerol-phosphate dehydratase
CLO1313_RS02150	Clo1313_0426	0.873	-0.195	0.014877928	0.026701919	phosphoribosylaminoimidazole-succinocarboxamide synthase
CLO1313_RS02165	Clo1313_0429	0.767	-0.383	5.45E-06	1.59E-05	imidazole glycerol phosphate synthase subunit HisH
CLO1313_RS02170	Clo1313_0430	0.831	-0.267	0.005046907	0.009925031	bifunctional phosphoribosyl-AMP cyclohydrolase/phosphoribosyl-ATP pyrophosphatase
CLO1313_RS02175	Clo1313_0431	0.882	-0.182	0.094717399	0.140552934	transcriptional regulator
CLO1313_RS02180	Clo1313_0432	0.81	-0.305	6.23E-05	0.000158522	molecular chaperone GroES
CLO1313_RS02190	Clo1313_0434	0.837	-0.256	0.018866416	0.033380756	histidine kinase
CLO1313_RS02195	Clo1313_0435	0.838	-0.256	0.003582685	0.007201388	hypothetical protein
CLO1313_RS02200	Clo1313_0436	1.042	0.059	0.431708132	0.516843388	glycoside hydrolase
CLO1313_RS02205	Clo1313_0437	1.239	0.309	0.000490257	0.001095589	hypothetical protein
CLO1313_RS02210	Clo1313_0438	0.89	-0.169	0.044389499	0.072232288	transcription elongation factor GreA
CLO1313_RS02215	Clo1313_0439	1.082	0.114	0.234044483	0.309070632	anti-sigma factor antagonist
CLO1313_RS02220	Clo1313_0440	0.983	-0.024	0.776509214	0.823753496	anti-sigma regulatory factor
CLO1313_RS02225	Clo1313_0441	0.967	-0.049	0.594375877	0.669699706	DNA-directed RNA polymerase sigma-70 factor
CLO1313_RS02230	Clo1313_0442	0.903	-0.147	0.322345861	0.40652449	anti-sigma regulatory factor
CLO1313_RS02235	Clo1313_R0013	0.841	-0.25	0.444825201	0.529167306	
CLO1313_RS02240	Clo1313_R0014	0.93	-0.105	0.533950052	0.612826715	
CLO1313_RS02245	Clo1313_R0015	0.817	-0.291	0.380245374	0.466022017	
CLO1313_RS02255	Clo1313_0444	0.513	-0.964	3.86E-38	7.53E-37	30S ribosomal protein S10
CLO1313_RS02260	Clo1313_0445	0.518	-0.949	3.79E-32	5.38E-31	50S ribosomal protein L3
CLO1313_RS02265	Clo1313_0446	0.563	-0.83	1.60E-21	1.45E-20	50S ribosomal protein L4
CLO1313_RS02270	Clo1313_0447	0.595	-0.75	5.84E-13	3.19E-12	50S ribosomal protein L23
CLO1313_RS02275	Clo1313_0448	0.584	-0.776	7.64E-14	4.50E-13	50S ribosomal protein L2
CLO1313_RS02280	Clo1313_0449	0.627	-0.673	7.44E-08	2.60E-07	30S ribosomal protein S19
CLO1313_RS02285	Clo1313_0450	0.623	-0.683	1.69E-09	6.90E-09	50S ribosomal protein L22
CLO1313_RS02290	Clo1313_0451	0.623	-0.682	2.48E-08	9.04E-08	30S ribosomal protein S3
CLO1313_RS02295	Clo1313_0452	0.632	-0.663	1.32E-07	4.53E-07	50S ribosomal protein L16

CLO1313_RS02300	Clo1313_0453	0.649	-0.623	5.77E-06	1.68E-05	50S ribosomal protein L29
CLO1313_RS02305	Clo1313_0454	0.683	-0.549	0.000189835	0.000453277	30S ribosomal protein S17
CLO1313_RS02310	Clo1313_0455	0.666	-0.585	8.84E-06	2.50E-05	50S ribosomal protein L14
CLO1313_RS02315	Clo1313_0456	0.671	-0.575	2.99E-05	7.87E-05	50S ribosomal protein L24
CLO1313_RS02320	Clo1313_0457	0.673	-0.571	1.29E-05	3.56E-05	50S ribosomal protein L5
CLO1313_RS02325		0.73	-0.454	0.000331523	0.000763624	30S ribosomal protein S14 type Z
CLO1313_RS02330	Clo1313_0459	0.712	-0.489	8.13E-06	2.31E-05	30S ribosomal protein S8
CLO1313_RS02335	Clo1313_0460	0.688	-0.539	2.40E-06	7.25E-06	50S ribosomal protein L6
CLO1313_RS02340	Clo1313_0461	0.689	-0.538	7.84E-07	2.50E-06	50S ribosomal protein L18
CLO1313_RS02345	Clo1313_0462	0.692	-0.531	5.83E-07	1.90E-06	30S ribosomal protein S5
CLO1313_RS02350	Clo1313_0463	0.673	-0.572	5.28E-08	1.87E-07	50S ribosomal protein L30
CLO1313_RS02355	Clo1313_0464	0.676	-0.564	4.04E-07	1.33E-06	50S ribosomal protein L15
CLO1313_RS02360	Clo1313_0465	0.697	-0.52	1.83E-06	5.59E-06	preprotein translocase subunit SecY
CLO1313_RS02365	Clo1313_0466	0.706	-0.502	6.63E-06	1.91E-05	adenylate kinase
CLO1313_RS02375	Clo1313_0468	0.677	-0.562	2.72E-12	1.40E-11	hypothetical protein
CLO1313_RS02380	Clo1313_0469	0.673	-0.572	4.45E-11	2.11E-10	translation initiation factor IF-1
CLO1313_RS02385	Clo1313_0470	0.711	-0.492	1.51E-08	5.65E-08	50S ribosomal protein L36
CLO1313_RS02390	Clo1313_0471	0.586	-0.771	4.94E-31	6.77E-30	30S ribosomal protein S13
CLO1313_RS02395	Clo1313_0472	0.585	-0.773	5.64E-28	7.05E-27	30S ribosomal protein S11
CLO1313_RS02400	Clo1313_0473	0.63	-0.667	2.17E-24	2.24E-23	30S ribosomal protein S4
CLO1313_RS02405	Clo1313_0474	0.645	-0.632	2.13E-19	1.73E-18	DNA-directed RNA polymerase subunit alpha
CLO1313_RS02410	Clo1313_0475	0.815	-0.294	1.21E-05	3.34E-05	50S ribosomal protein L17
CLO1313_RS02415	Clo1313_0476	0.789	-0.342	2.09E-05	5.60E-05	energy-coupling factor transporter ATPase
CLO1313_RS02420	Clo1313_0477	0.738	-0.439	0.000155296	0.000375288	16S rRNA methyltransferase
CLO1313_RS02425	Clo1313_0478	0.981	-0.028	0.696364514	0.755570614	type III restriction endonuclease subunit R
CLO1313_RS02430	Clo1313_0479	1.012	0.017	0.827817371	0.860528352	hypothetical protein
CLO1313_RS02435	Clo1313_0480	1.107	0.146	0.042408949	0.069499693	DNA methylase
CLO1313_RS02440	Clo1313_0481	0.96	-0.059	0.493179575	0.573051354	hypothetical protein
CLO1313_RS02445	Clo1313_0482	0.997	-0.004	0.955026428	0.964280079	hypothetical protein
CLO1313_RS02450	Clo1313_0483	1.261	0.334	0.840320767	0.872316366	LuxR family transcriptional regulator

CLO1313_RS02455	Clo1313_0484	1.47	0.556	0.500270684	0.579270958	hypothetical protein
CLO1313_RS02460	Clo1313_0485	1.116	0.159	0.085241718	0.128269043	copper amine oxidase
CLO1313_RS02465	Clo1313_0486	1.188	0.249	0.003459068	0.006971603	resolvase
CLO1313_RS02470	Clo1313_0487	0.833	-0.263	0.001447666	0.003066067	cobalt ABC transporter ATP-binding protein
CLO1313_RS02475	Clo1313_0488	0.744	-0.426	9.37E-06	2.64E-05	transporter
CLO1313_RS02480	Clo1313_0489	0.688	-0.539	8.04E-16	5.42E-15	glucokinase
CLO1313_RS02485	Clo1313_0490	1.042	0.059	0.48593679	0.566611366	tRNA pseudouridine synthase A
CLO1313_RS02490	Clo1313_0491	1.467	0.553	4.14E-10	1.79E-09	CarD family transcriptional regulator
CLO1313_RS02500	Clo1313_0493	0.61	-0.714	3.43E-12	1.77E-11	ABC transporter
CLO1313_RS02505	Clo1313_0494	0.632	-0.662	1.86E-14	1.14E-13	ABC transporter
CLO1313_RS02510	Clo1313_0495	1.647	0.72	8.02E-10	3.38E-09	two-component system sensor histidine kinase
CLO1313_RS02515	Clo1313_0496	1.175	0.233	0.040057688	0.066007146	histidine kinase
CLO1313_RS02520	Clo1313_0497	1.085	0.118	0.228099749	0.301618672	2-C-methyl-D-erythritol 2,4-cyclodiphosphate synthase
CLO1313_RS02525	Clo1313_0498	0.677	-0.562	1.29E-13	7.40E-13	prolyl-tRNA synthetase
CLO1313_RS02530	Clo1313_0499	1.113	0.155	0.295196542	0.378330952	hypothetical protein
CLO1313_RS02535	Clo1313_0500	2.04	1.029	7.74E-05	0.000195085	pectinesterase
CLO1313_RS02540	Clo1313_0501	1.052	0.074	0.655832145	0.722837413	pectate lyase
CLO1313_RS02550	Clo1313_0503	1.051	0.072	0.6950155	0.754379835	ATP-binding protein
CLO1313_RS02565		1.489	0.574	0.000118362	0.000289532	hypothetical protein
CLO1313_RS02570	Clo1313_0505	1.152	0.204	0.094765682	0.140555034	hypothetical protein
CLO1313_RS02575		1.177	0.235	0.02687751	0.045929148	transposase
CLO1313_RS02590		4.473	2.161	6.17E-69	3.30E-67	hypothetical protein
CLO1313_RS02605	Clo1313_0509	0.682	-0.552	0.001164633	0.002493029	hypothetical protein
CLO1313_RS02610	Clo1313_0510	2.911	1.542	8.24E-60	3.48E-58	peptide ABC transporter substrate-binding protein
CLO1313_RS02615	Clo1313_0511	2.542	1.346	2.68E-45	6.70E-44	peptide ABC transporter ATP-binding protein
CLO1313_RS02620	Clo1313_0512	2.29	1.195	8.52E-35	1.39E-33	peptide ABC transporter ATP-binding protein
CLO1313_RS02625	Clo1313_0513	2.191	1.131	9.13E-32	1.29E-30	peptide ABC transporter permease
CLO1313_RS02630	Clo1313_0514	1.953	0.966	1.11E-28	1.44E-27	peptide ABC transporter permease
CLO1313_RS02635	Clo1313_0515	0.571	-0.808	0.005261997	0.010307465	copper amine oxidase
CLO1313_RS02640	Clo1313_0516	0.942	-0.086	0.374330573	0.460089093	MFS transporter

CLO1313_RS02645	Clo1313_0517	1.012	0.018	0.848701548	0.879446008	4Fe-4S ferredoxin
CLO1313_RS02650	Clo1313_0518	1.444	0.53	3.22E-07	1.06E-06	GntR family transcriptional regulator
CLO1313_RS02655	Clo1313_0519	1.197	0.259	0.005323055	0.010420262	ABC transporter
CLO1313_RS02660	Clo1313_0520	1.061	0.085	0.466865686	0.548855426	hypothetical protein
CLO1313_RS02665	Clo1313_0521	0.856	-0.224	0.081233608	0.123289267	glycoside hydrolase
CLO1313_RS02670	Clo1313_0522	0.875	-0.193	0.158274953	0.219651357	endo-1,4-beta-xylanase
CLO1313_RS02680	Clo1313_0524	1.342	0.425	0.000213257	0.000505181	hypothetical protein
CLO1313_RS02690		0.338	-1.563	0.435458566	0.520091958	hypothetical protein
CLO1313_RS02695	Clo1313_0526	1.146	0.197	0.743297238	0.794139087	hypothetical protein
CLO1313_RS02700	Clo1313_0527	1.333	0.415	6.66E-06	1.92E-05	Crp/Fnr family transcriptional regulator
CLO1313_RS02705	Clo1313_0528	190.971	7.577	2.04E-113	2.19E-111	4Fe-4S ferredoxin
CLO1313_RS02710	Clo1313_0529	70.323	6.136	1.57E-182	9.41E-180	membrane protein
CLO1313_RS02715	Clo1313_0530	18.829	4.235	3.02E-85	2.10E-83	HymD protein
CLO1313_RS02720	Clo1313_0531	21.195	4.406	2.69E-39	5.57E-38	ABC transporter substrate-binding protein
CLO1313_RS02725	Clo1313_0532	4.552	2.186	1.87E-32	2.70E-31	chemotaxis protein
CLO1313_RS02730		1.647	0.72	9.83E-06	2.76E-05	multidrug ABC transporter ATP-binding protein
CLO1313_RS02735		1.076	0.105	0.596330994	0.670313587	hypothetical protein
CLO1313_RS02740	Clo1313_0534	0.96	-0.058	0.90286679	0.923498467	hypothetical protein
CLO1313_RS02745	Clo1313_0535	0.837	-0.257	0.267073436	0.348089193	hypothetical protein
CLO1313_RS02750	Clo1313_0536	1.262	0.336	0.702613595	0.7608995	hypothetical protein
CLO1313_RS02755	Clo1313_0537	0.905	-0.145	0.865025818	0.893016327	hypothetical protein
CLO1313_RS02760	Clo1313_0538	2.017	1.012	0.135431585	0.191584587	hypothetical protein
CLO1313_RS02765	Clo1313_0539	0.705	-0.504	6.46E-15	4.10E-14	glycosyl transferase
CLO1313_RS02770	Clo1313_0540	0.417	-1.261	2.70E-05	7.15E-05	hypothetical protein
CLO1313_RS02780	Clo1313_0542	0.923	-0.116	0.457442157	0.540744592	NADH:flavin oxidoreductase
CLO1313_RS02785	Clo1313_0543	1.133	0.18	0.19338741	0.261417886	RNA polymerase subunit sigma-24
CLO1313_RS02790	Clo1313_0544	1.543	0.626	9.20E-07	2.92E-06	hypothetical protein
CLO1313_RS02795	Clo1313_0545	1.051	0.072	0.416068239	0.501321273	hypothetical protein
CLO1313_RS02800	Clo1313_0546	1.507	0.592	0.000251203	0.000588561	hypothetical protein
CLO1313_RS02805	Clo1313_0547	0.919	-0.123	0.549330937	0.628315592	glutamine amidotransferase

CLO1313_RS02810	Clo1313_0548	0.785	-0.35	0.03797113	0.062879855	hypothetical protein
CLO1313_RS02815	Clo1313_0549	1.376	0.46	9.34E-07	2.96E-06	ABC transporter permease
CLO1313_RS02820	Clo1313_0550	0.731	-0.452	2.11E-06	6.40E-06	hypothetical protein
CLO1313_RS02825	Clo1313_0551	0.823	-0.282	0.000795884	0.001727764	inorganic phosphate transporter PiT
CLO1313_RS02830	Clo1313_0552	0.839	-0.254	0.004341299	0.008627937	hypothetical protein
CLO1313_RS02835	Clo1313_0553	0.829	-0.271	0.001351925	0.002867343	hypothetical protein
CLO1313_RS02840	Clo1313_0554	1.111	0.152	0.08728136	0.130812992	hydrogenase
CLO1313_RS02845	Clo1313_0555	1.717	0.78	1.74E-08	6.43E-08	peptidoglycan-binding protein LysM
CLO1313_RS02855	Clo1313_0557	0.485	-1.045	0.005103395	0.010022974	hypothetical protein
CLO1313_RS02860	Clo1313_0558	1.347	0.43	0.322031369	0.406298727	superoxide dismutase
CLO1313_RS02865	Clo1313_0559	1.151	0.202	0.807090236	0.846611968	hypothetical protein
CLO1313_RS02870		0.66	-0.598	0.048095211	0.077588777	hypothetical protein
CLO1313_RS02875	Clo1313_0562	0.71	-0.494	0.187986642	0.255911003	hypothetical protein
CLO1313_RS02880	Clo1313_0563	1.631	0.706	3.64E-18	2.75E-17	carbohydrate-binding protein
CLO1313_RS02890	Clo1313_0565	0.97	-0.044	0.64541016	0.713448238	hydrogenase assembly protein HupF
CLO1313_RS02915	Clo1313_0571	0.67	-0.579	4.96E-09	1.93E-08	NADH dehydrogenase
CLO1313_RS02920	Clo1313_0572	0.619	-0.693	1.14E-11	5.68E-11	ech hydrogenase subunit EchD
CLO1313_RS02925	Clo1313_0573	0.352	-1.508	4.71E-35	7.85E-34	NADH ubiquinone oxidoreductase
CLO1313_RS02930	Clo1313_0574	0.359	-1.48	6.78E-53	2.26E-51	NADH dehydrogenase subunit 1
CLO1313_RS02935	Clo1313_0575	0.406	-1.3	3.69E-41	8.15E-40	NADH/Ubiquinone/plastoquinone (complex I)
CLO1313_RS02940	Clo1313_0577	1.192	0.254	0.028898121	0.04904667	elongation factor GreAB
CLO1313_RS02945	Clo1313_0578	0.752	-0.411	2.31E-09	9.36E-09	citrate synthase
CLO1313_RS02950	Clo1313_0579	0.575	-0.8	1.41E-12	7.39E-12	histidine decarboxylase
CLO1313_RS02955	Clo1313_0580	2.014	1.01	0.026731877	0.045706328	chemotaxis protein CheW
CLO1313_RS02960	Clo1313_0581	1.694	0.761	0.000671222	0.001471488	chemotaxis protein
CLO1313_RS02970	Clo1313_0583	0.738	-0.437	2.54E-06	7.64E-06	hypothetical protein
CLO1313_RS02975	Clo1313_0584	0.775	-0.368	8.47E-07	2.69E-06	hypothetical protein
CLO1313_RS02980	Clo1313_0585	0.987	-0.019	0.858184779	0.887175509	hypothetical protein
CLO1313_RS02985	Clo1313_0586	1.126	0.171	0.124795135	0.178593532	3-phosphoglycerate dehydrogenase
CLO1313_RS02990	Clo1313_0587	3.338	1.739	2.81E-55	1.03E-53	chemotaxis protein

CLO1313_RS02995	Clo1313_0588	0.655	-0.611	1.60E-09	6.54E-09	nitroreductase
CLO1313_RS03000	Clo1313_0589	2.115	1.081	8.24E-16	5.53E-15	copper amine oxidase
CLO1313_RS03005	Clo1313_0590	0.961	-0.058	0.528051137	0.607451231	cell division protein FtsK
CLO1313_RS03010	Clo1313_0591	0.94	-0.089	0.368448006	0.455284536	hypothetical protein
CLO1313_RS03015	Clo1313_0592	0.992	-0.011	0.915376347	0.933746144	1,4-dihydroxy-2-naphthoate octaprenyltransferase
CLO1313_RS03020	Clo1313_0593	1.287	0.364	0.000964163	0.002081732	hypothetical protein
CLO1313_RS03025	Clo1313_0594	1.229	0.298	0.138441343	0.194648658	hypothetical protein
CLO1313_RS03030	Clo1313_0595	0.842	-0.248	0.00862165	0.016313141	hypothetical protein
CLO1313_RS03035	Clo1313_0596	0.741	-0.433	0.001191615	0.002548969	hypothetical protein
CLO1313_RS03040	Clo1313_0597	0.83	-0.268	0.003417344	0.006901423	cell cycle protein
CLO1313_RS03045	Clo1313_0598	0.893	-0.163	0.087961573	0.131471144	peptidoglycan glycosyltransferase
CLO1313_RS03050	Clo1313_0599	0.946	-0.079	0.42822244	0.513285011	hypothetical protein
CLO1313_RS03055	Clo1313_0600	0.543	-0.88	9.75E-20	8.01E-19	hypothetical protein
CLO1313_RS03060	Clo1313_0601	0.81	-0.303	0.137342053	0.193375032	fibronectin type III
CLO1313_RS03065	Clo1313_0602	1.561	0.643	9.17E-10	3.86E-09	transposase
CLO1313_RS03070	Clo1313_0603	1.382	0.467	3.33E-08	1.20E-07	hypothetical protein
CLO1313_RS03075	Clo1313_0604	0.91	-0.137	0.223006593	0.296321122	hypothetical protein
CLO1313_RS03080	Clo1313_0605	0.622	-0.685	0.000400458	0.000911901	hypothetical protein
CLO1313_RS03090		1.193	0.254	0.068527921	0.106374346	transposase
CLO1313_RS03105	Clo1313_0609	0.92	-0.12	0.371308269	0.458063964	polysaccharide biosynthesis protein GtrA
CLO1313_RS03110	Clo1313_0610	1.433	0.519	2.52E-06	7.60E-06	RNA 2'-phosphotransferase
CLO1313_RS03115	Clo1313_0611	1.094	0.13	0.501713501	0.580717402	hypothetical protein
CLO1313_RS03120		1.385	0.47	0.019282137	0.033956036	hypothetical protein
CLO1313_RS03125	Clo1313_0612	0.564	-0.826	4.92E-09	1.92E-08	signal transduction histidine kinase
CLO1313_RS03130	Clo1313_0613	0.543	-0.881	5.18E-15	3.32E-14	cephalosporin deacetylase
CLO1313_RS03135	Clo1313_R0017	0.837	-0.257	0.435037588	0.5200556	
CLO1313_RS03140	Clo1313_R0018	0.82	-0.286	0.380744321	0.4664429	
CLO1313_RS03150	Clo1313_0614	0.992	-0.011	0.898831028	0.920312139	sporulation protein SpoVB
CLO1313_RS03155	Clo1313_0615	0.249	-2.004	6.92E-74	4.07E-72	hypothetical protein
CLO1313_RS03160	Clo1313_0616	0.356	-1.491	2.33E-52	7.44E-51	ABC transporter

CLO1313_RS03165	Clo1313_0617	0.368	-1.444	9.16E-47	2.47E-45	membrane protein
CLO1313_RS03170	Clo1313_0618	0.425	-1.234	6.26E-35	1.03E-33	two-component system sensor histidine kinase
CLO1313_RS03175	Clo1313_0619	0.617	-0.696	9.43E-09	3.59E-08	two-component system response regulator
CLO1313_RS03185	Clo1313_0622	1.262	0.336	0.00038569	0.000880948	acyl-ACP thioesterase
CLO1313_RS03190	Clo1313_0623	0.943	-0.084	0.339451539	0.4249385	haloacid dehalogenase
CLO1313_RS03195	Clo1313_0624	0.987	-0.018	0.841128707	0.872852939	hypothetical protein
CLO1313_RS03200	Clo1313_0625	0.844	-0.245	0.005228032	0.010254328	VWA domain-containing protein
CLO1313_RS03205	Clo1313_0626	1.264	0.338	4.23E-05	0.000109628	radical SAM protein
CLO1313_RS03210	Clo1313_0627	1.651	0.724	6.51E-05	0.000164459	cellulosome anchoring protein cohesin subunit
CLO1313_RS03220	Clo1313_0629	2.022	1.016	1.61E-11	7.85E-11	Cell surface glycoprotein 2
CLO1313_RS03225	Clo1313_0630	2.056	1.04	1.26E-10	5.79E-10	cellulosome-anchoring protein
CLO1313_RS03230	Clo1313_0631	3.663	1.873	9.36E-141	1.65E-138	transglutaminase
CLO1313_RS03235	Clo1313_0632	0.699	-0.516	0.097052506	0.143308945	hypothetical protein
CLO1313_RS03240	Clo1313_0633	0.646	-0.631	0.000183122	0.000438646	hypothetical protein
CLO1313_RS03245	Clo1313_0634	0.627	-0.674	2.83E-12	1.46E-11	RNA methyltransferase
CLO1313_RS03250	Clo1313_0635	0.854	-0.228	0.004158749	0.008276104	chemotaxis protein CheY
CLO1313_RS03265	Clo1313_0638	0.721	-0.471	1.27E-09	5.25E-09	transcriptional regulator
CLO1313_RS03270	Clo1313_0639	1.169	0.225	0.019882521	0.034849609	universal stress protein UspA
CLO1313_RS03275	Clo1313_0640	0.705	-0.505	1.22E-06	3.81E-06	fumarate hydratase
CLO1313_RS03280	Clo1313_0641	1.107	0.146	0.355792255	0.442196838	fumarate hydratase
CLO1313_RS03285	Clo1313_0642	1.288	0.365	0.000111768	0.000275199	disulfide oxidoreductase
CLO1313_RS03290	Clo1313_0643	1.282	0.358	0.000297378	0.000691881	adenylosuccinate synthetase
CLO1313_RS03295	Clo1313_R0020	0.556	-0.846	6.70E-11	3.15E-10	
CLO1313_RS03300	Clo1313_R0021	0.465	-1.106	9.90E-16	6.61E-15	
CLO1313_RS03305	Clo1313_R0022	0.579	-0.789	1.50E-09	6.14E-09	
CLO1313_RS03310	Clo1313_R0023	0.558	-0.842	3.08E-09	1.23E-08	
CLO1313_RS03315	Clo1313_R0024	0.523	-0.934	1.44E-09	5.94E-09	
CLO1313_RS03320	Clo1313_0644	0.836	-0.258	0.013709833	0.024798426	transposase
CLO1313_RS03325	Clo1313_0645	0.823	-0.281	0.00061493	0.001351044	glycosyltransferase
CLO1313_RS03330	Clo1313_0646	0.808	-0.308	0.000169459	0.000408855	glycosyl transferase

CLO1313_RS03335	Clo1313_0647	1	0	0.998809974	0.998809974	hypothetical protein
CLO1313_RS03340	Clo1313_0648	1.244	0.315	1.01E-05	2.82E-05	glycosyl transferase
CLO1313_RS03345	Clo1313_0649	0.32	-1.642	0.096569304	0.142735999	hypothetical protein
CLO1313_RS03350	Clo1313_0650	1.518	0.602	0.260022975	0.340527904	glycosyl transferase family 2
CLO1313_RS03355	Clo1313_0651	9.872	3.303	3.90E-114	4.34E-112	diaminopimelate epimerase
CLO1313_RS03360	Clo1313_0652	8.323	3.057	9.45E-115	1.09E-112	LL-diaminopimelate aminotransferase
CLO1313_RS03365	Clo1313_0653	7.21	2.85	2.17E-97	1.97E-95	transcriptional regulator
CLO1313_RS03370	Clo1313_0654	1.356	0.439	5.09E-08	1.80E-07	2-hydroxyglutaryl-CoA dehydratase
CLO1313_RS03375	Clo1313_0655	1.162	0.216	0.05133548	0.082593941	2-hydroxyglutaryl-CoA dehydratase
CLO1313_RS03390	Clo1313_0658	1.255	0.328	2.06E-05	5.55E-05	radical SAM protein
CLO1313_RS03395	Clo1313_0659	1.119	0.162	0.041875836	0.068701112	DNA polymerase
CLO1313_RS03400	Clo1313_0660	1.152	0.204	0.009205116	0.01728625	uracil-DNA glycosylase
CLO1313_RS03405	Clo1313_R0025	0.85	-0.234	0.473861374	0.555241172	
CLO1313_RS03410	Clo1313_R0026	0.875	-0.192	0.237282486	0.313070909	
CLO1313_RS03415	Clo1313_R0027	0.83	-0.268	0.41304684	0.498682557	
CLO1313_RS03425	Clo1313_R0029	1.067	0.094	0.934041221	0.947255217	
CLO1313_RS03430	Clo1313_0661	1.035	0.05	0.589459591	0.665006518	thiamine biosynthesis protein ThiF
CLO1313_RS03435	Clo1313_0662	1.195	0.256	0.042731022	0.06998926	transposase
CLO1313_RS03440	Clo1313_0663	0.88	-0.184	0.041641946	0.06839222	hypothetical protein
CLO1313_RS03445	Clo1313_0665	1.833	0.875	6.10E-23	5.86E-22	glycosidase
CLO1313_RS03450	Clo1313_0666	1.748	0.806	1.86E-13	1.06E-12	nucleotidyltransferase
CLO1313_RS03455	Clo1313_0667	1.397	0.483	7.13E-10	3.04E-09	glycosyl transferase family 1
CLO1313_RS03460	Clo1313_0668	1.25	0.322	0.000454417	0.001026976	glycosyltransferase
CLO1313_RS03465	Clo1313_0669	0.832	-0.265	0.286586502	0.369189398	mannose-6-phosphate isomerase
CLO1313_RS03470	Clo1313_0670	0.988	-0.018	0.812943194	0.851857666	zinc permease
CLO1313_RS03475	Clo1313_0671	0.891	-0.166	0.0232571	0.040212913	hemerythrin
CLO1313_RS03480	Clo1313_0672	0.728	-0.458	1.68E-09	6.84E-09	flavin reductase
CLO1313_RS03490		1.494	0.58	0.27285632	0.354240738	hypothetical protein
CLO1313_RS03495	Clo1313_0675	0.599	-0.739	1.43E-11	7.03E-11	hypothetical protein
CLO1313_RS03500	Clo1313_0676	0.724	-0.465	0.000152923	0.000369851	hypothetical protein

CLO1313_RS03505	Clo1313_0677	0.932	-0.102	0.108902127	0.158311914	AMP-dependent synthetase
CLO1313_RS03510	Clo1313_0678	1.723	0.785	2.46E-09	9.91E-09	heat-shock protein Hsp20
CLO1313_RS03515	Clo1313_0679	2.049	1.035	2.39E-20	2.02E-19	ADP-ribose pyrophosphatase
CLO1313_RS03520	Clo1313_0680	1.567	0.648	3.44E-11	1.64E-10	peptidase M56
CLO1313_RS03525	Clo1313_0681	0.72	-0.474	1.18E-06	3.70E-06	iron-sulfur protein
CLO1313_RS03530	Clo1313_0682	0.937	-0.095	0.298130267	0.380626935	citrate transporter
CLO1313_RS03535	Clo1313_0683	0.883	-0.18	0.080411387	0.12228892	hypothetical protein
CLO1313_RS03540	Clo1313_0684	0.745	-0.425	4.61E-09	1.80E-08	VWA domain-containing protein
CLO1313_RS03545	Clo1313_0685	1.06	0.083	0.438541823	0.523121354	dockerin
CLO1313_RS03550	Clo1313_0686	1.449	0.535	5.11E-05	0.000131615	alpha/beta hydrolase
CLO1313_RS03555		1.064	0.089	0.758662596	0.807056927	hypothetical protein
CLO1313_RS03560	Clo1313_0688	0.92	-0.12	0.8152633	0.853434636	hypothetical protein
CLO1313_RS03565	Clo1313_0689	0.44	-1.184	6.30E-65	3.05E-63	peptidase S8
CLO1313_RS03570		0.707	-0.501	0.418950966	0.503782657	hypothetical protein
CLO1313_RS03575	Clo1313_0691	1.046	0.065	0.683974059	0.745606087	membrane protein
CLO1313_RS03580	Clo1313_0692	0.997	-0.005	0.969838982	0.976350153	TetR family transcriptional regulator
CLO1313_RS03585	Clo1313_0693	1.337	0.419	0.000760936	0.00165726	lipase
CLO1313_RS03590	Clo1313_0694	0.982	-0.027	0.75779439	0.806467486	hypothetical protein
CLO1313_RS03595		0.992	-0.012	0.926574639	0.941645999	hypothetical protein
CLO1313_RS03600		1.168	0.224	0.088189447	0.131647662	AraC family transcriptional regulator
CLO1313_RS03605	Clo1313_0696	1.156	0.209	0.056363958	0.089626463	transcriptional regulator
CLO1313_RS03610	Clo1313_0697	0.865	-0.21	0.045161892	0.073290322	peptidase M56
CLO1313_RS03615	Clo1313_0698	0.528	-0.922	5.72E-24	5.80E-23	ABC transporter
CLO1313_RS03620	Clo1313_0699	0.669	-0.58	1.11E-09	4.65E-09	ABC transporter
CLO1313_RS03625	Clo1313_0700	0.688	-0.539	1.38E-06	4.28E-06	aminoacyl-histidine dipeptidase
CLO1313_RS03635	Clo1313_0702	0.717	-0.479	0.002376974	0.004885912	adenosylcobinamide kinase
CLO1313_RS03640	Clo1313_0703	0.669	-0.581	1.05E-05	2.93E-05	adenosylcobinamide-GDP ribazoletransferase
CLO1313_RS03650	Clo1313_0705	0.071	-3.82	2.25E-170	6.76E-168	hypothetical protein
CLO1313_RS03655	Clo1313_0706	0.143	-2.808	1.82E-100	1.82E-98	MBL fold metallo-hydrolase
CLO1313_RS03660	Clo1313_0707	1.637	0.711	4.49E-09	1.76E-08	chemotaxis protein

CLO1313_RS03665	Clo1313_0708	1.875	0.907	2.50E-26	2.83E-25	2-isopropylmalate synthase
CLO1313_RS03670	Clo1313_0709	1.617	0.693	1.16E-20	9.94E-20	aconitate hydratase
CLO1313_RS03675	Clo1313_0710	1.599	0.677	1.07E-19	8.79E-19	GntR family transcriptional regulator
CLO1313_RS03680	Clo1313_0711	2.209	1.143	2.40E-32	3.47E-31	SAM-dependent methyltransferase
CLO1313_RS03685	Clo1313_0712	1.779	0.831	1.05E-11	5.24E-11	RNA pseudouridine synthase
CLO1313_RS03690	Clo1313_0713	1.704	0.769	8.40E-11	3.91E-10	SAM-dependent methyltransferase
CLO1313_RS03695	Clo1313_0714	1.241	0.312	0.00044858	0.001015313	carbohydrate-binding protein
CLO1313_RS03700	Clo1313_0715	1.307	0.386	6.77E-06	1.95E-05	two-component system response regulator
CLO1313_RS03705	Clo1313_0716	1.177	0.235	0.001219833	0.002605613	peptidyl-prolyl cis-trans isomerase
CLO1313_RS03710	Clo1313_0717	1.424	0.51	3.13E-10	1.37E-09	glucose-1-phosphate adenylyltransferase
CLO1313_RS03720	Clo1313_0719	1.455	0.541	1.49E-09	6.11E-09	lipid kinase
CLO1313_RS03725	Clo1313_0720	0.672	-0.573	9.45E-16	6.32E-15	enoyl-ACP reductase
CLO1313_RS03730	Clo1313_0721	0.326	-1.617	2.30E-12	1.20E-11	macrolide ABC transporter ATP-binding protein
CLO1313_RS03735	Clo1313_0722	0.341	-1.552	4.95E-25	5.32E-24	hypothetical protein
CLO1313_RS03740	Clo1313_0723	0.609	-0.715	2.61E-09	1.05E-08	hypothetical protein
CLO1313_RS03745	Clo1313_R0030	0.184	-2.439	0.082658449	0.124945912	
CLO1313_RS03750	Clo1313_0724	0.843	-0.247	0.600003408	0.673431969	membrane protein
CLO1313_RS03755	Clo1313_0725	0.764	-0.388	0.297686251	0.380591719	hypothetical protein
CLO1313_RS03760	Clo1313_0726	0.641	-0.642	0.003129312	0.006345373	membrane protein
CLO1313_RS03765	Clo1313_0727	0.783	-0.353	3.73E-06	1.10E-05	spore protein
CLO1313_RS03770	Clo1313_0728	0.644	-0.635	2.27E-17	1.64E-16	galactosyldiacylglycerol synthase
CLO1313_RS03775	Clo1313_0729	1.639	0.713	1.42E-05	3.91E-05	hypothetical protein
CLO1313_RS03780	Clo1313_0730	1.046	0.064	0.813227333	0.851857761	peptidase S11
CLO1313_RS03785	Clo1313_0731	0.375	-1.415	9.05E-21	7.78E-20	rRNA methyltransferase
CLO1313_RS03790	Clo1313_0732	0.709	-0.496	4.94E-09	1.93E-08	potassium transporter TrkA
CLO1313_RS03795	Clo1313_0733	0.665	-0.59	1.52E-10	6.91E-10	potassium transporter KefA
CLO1313_RS03800	Clo1313_0734	0.598	-0.743	3.73E-17	2.67E-16	tRNA-guanine transglycosylase
CLO1313_RS03805	Clo1313_0735	0.785	-0.349	6.91E-06	1.98E-05	radical SAM protein
CLO1313_RS03810	Clo1313_0736	1.412	0.497	1.26E-05	3.49E-05	anti-sigma regulatory factor
CLO1313_RS03815	Clo1313_0737	0.909	-0.137	0.102087536	0.14978499	copper amine oxidase

CLO1313_RS03820	Clo1313_0738	1.103	0.141	0.131228574	0.186607157	hypothetical protein
CLO1313_RS03830	Clo1313_0740	1.039	0.055	0.496602281	0.575467636	chromosome partitioning ATPase
CLO1313_RS03835	Clo1313_0741	0.705	-0.504	0.000114262	0.000280879	hypothetical protein
CLO1313_RS03840	Clo1313_0742	1.298	0.376	0.517260259	0.596181213	phospholipase C
CLO1313_RS03845	Clo1313_0743	0.945	-0.081	0.780462459	0.82620793	sporulation protein
CLO1313_RS03850	Clo1313_0744	0.916	-0.126	0.536847476	0.615680911	hypothetical protein
CLO1313_RS03855	Clo1313_0745	0.706	-0.502	1.69E-11	8.23E-11	hypothetical protein
CLO1313_RS03860	Clo1313_0746	1.942	0.958	0.503109396	0.582108441	photosystem reaction center subunit H
CLO1313_RS03865	Clo1313_0747	0.835	-0.259	0.818496195	0.855583858	hypothetical protein
CLO1313_RS03870	Clo1313_0748	1.779	0.831	0.104515911	0.152675702	hypothetical protein
CLO1313_RS03875	Clo1313_0749	0.835	-0.259	0.005968468	0.011577901	alanyl-tRNA synthetase
CLO1313_RS03880	Clo1313_0750	1.104	0.142	0.297777041	0.380591719	CRISPR-associated protein
CLO1313_RS03885	Clo1313_0751	1.137	0.185	0.223147122	0.296353006	DNA repair protein
CLO1313_RS03890	Clo1313_0752	1.087	0.121	0.713100168	0.768446785	hypothetical protein
CLO1313_RS03895	Clo1313_0753	1.083	0.114	0.607099322	0.680367172	hypothetical protein
CLO1313_RS03900	Clo1313_0754	1.127	0.172	0.402667863	0.488314161	hypothetical protein
CLO1313_RS03905	Clo1313_0755	1.067	0.094	0.507530982	0.586093729	hypothetical protein
CLO1313_RS03910	Clo1313_0756	1.127	0.173	0.353009873	0.4392849	hypothetical protein
CLO1313_RS03915	Clo1313_0757	1.232	0.301	0.062923553	0.098547164	hypothetical protein
CLO1313_RS03920	Clo1313_0758	1.153	0.205	0.376809896	0.462946693	hypothetical protein
CLO1313_RS03925	Clo1313_0759	1.086	0.119	0.639476195	0.709241535	hypothetical protein
CLO1313_RS03930	Clo1313_0760	0.945	-0.082	0.505380142	0.584284906	hypothetical protein
CLO1313_RS03935	Clo1313_0761	0.832	-0.266	0.278096927	0.359643244	hypothetical protein
CLO1313_RS03940	Clo1313_0762	1.196	0.258	0.023098883	0.040019382	CRISPR-associated protein
CLO1313_RS03960	Clo1313_0766	1.615	0.691	1.61E-11	7.85E-11	transposase
CLO1313_RS03965	Clo1313_0767	1.554	0.636	1.22E-05	3.37E-05	signal peptidase I
CLO1313_RS03970	Clo1313_0769	0.427	-1.228	0.019194435	0.033841335	hypothetical protein
CLO1313_RS03975	Clo1313_0770	0.976	-0.036	0.891021639	0.913563724	hypothetical protein
CLO1313_RS03980	Clo1313_0771	0.679	-0.558	0.019314343	0.03399279	hypothetical protein
CLO1313_RS03985	Clo1313_0772	0.871	-0.199	0.159661606	0.221370854	hypothetical protein

CLO1313_RS03990	Clo1313_0773	1.262	0.336	0.000618006	0.001356809	transposase
CLO1313_RS03995	Clo1313_0777	0.952	-0.071	0.702798138	0.7608995	hypothetical protein
CLO1313_RS04000	Clo1313_0778	0.81	-0.304	0.172551488	0.23770414	copper amine oxidase
CLO1313_RS04005	Clo1313_0779	1.006	0.008	0.933708228	0.947255217	copper amine oxidase
CLO1313_RS04010	Clo1313_0780	1.012	0.018	0.839263148	0.871520146	copper amine oxidase
CLO1313_RS04015	Clo1313_0781	0.922	-0.118	0.072557824	0.111709849	hypothetical protein
CLO1313_RS04020	Clo1313_0782	0.864	-0.212	0.487910257	0.568029061	hypothetical protein
CLO1313_RS04025	Clo1313_0783	0.929	-0.107	0.2273856	0.301072589	hypothetical protein
CLO1313_RS04030	Clo1313_0784	1.201	0.264	0.086813566	0.13030725	hypothetical protein
CLO1313_RS04035	Clo1313_0785	1.584	0.664	0.002739014	0.00559939	transposase
CLO1313_RS04045	Clo1313_0787	1.484	0.57	0.124818954	0.178593532	hypothetical protein
CLO1313_RS04050	Clo1313_0788	1.26	0.333	0.031436596	0.053054784	hypothetical protein
CLO1313_RS04060	Clo1313_0790	1.571	0.652	4.10E-09	1.61E-08	transposase
CLO1313_RS04065	Clo1313_0791	2.161	1.112	0.007579764	0.014478798	(2Fe-2S)-binding protein
CLO1313_RS04070	Clo1313_0792	1.102	0.14	0.30203851	0.384307803	hypothetical protein
CLO1313_RS04075	Clo1313_0793	1.022	0.031	0.665408539	0.730172048	amino acid ABC transporter substrate-binding protein
CLO1313_RS04080	Clo1313_0794	0.925	-0.113	0.120835982	0.173806767	polar amino acid ABC transporter permease
CLO1313_RS04085	Clo1313_0795	0.936	-0.095	0.180670533	0.246847803	polar amino acid ABC transporter ATP-binding protein
CLO1313_RS04090	Clo1313_0796	0.952	-0.07	0.442350087	0.526640695	hypothetical protein
CLO1313_RS04095	Clo1313_0797	1.329	0.411	5.64E-06	1.64E-05	hypothetical protein
CLO1313_RS04105	Clo1313_0799	2.489	1.315	2.54E-70	1.44E-68	membrane protein
CLO1313_RS04110	Clo1313_0800	0.875	-0.193	0.329619989	0.414478133	GCN5 family N-acetyltransferase
CLO1313_RS04115	Clo1313_0801	1	-0.001	0.996912808	0.997578082	hypothetical protein
CLO1313_RS04120	Clo1313_0802	1.045	0.063	0.617259953	0.688932862	MarR family transcriptional regulator
CLO1313_RS04125	Clo1313_0803	1.002	0.003	0.978968677	0.983560155	MATE family efflux transporter
CLO1313_RS04130	Clo1313_0804	0.905	-0.145	0.199353179	0.268339401	hypothetical protein
CLO1313_RS04135	Clo1313_0805	0.753	-0.409	1.43E-05	3.92E-05	peptidase S1
CLO1313_RS04140	Clo1313_0807	2.724	1.446	4.29E-26	4.80E-25	AraC family transcriptional regulator
CLO1313_RS04145		3.596	1.847	3.36E-34	5.33E-33	GCN5 family acetyltransferase
CLO1313_RS04150	Clo1313_0809	2.626	1.393	0.099878501	0.146831188	hypothetical protein

CLO1313_RS04155		1.662	0.733	1.85E-06	5.65E-06	transcriptional regulator
CLO1313_RS04160		1.641	0.715	0.011804128	0.021664982	transcriptional regulator
CLO1313_RS04165		1.547	0.63	0.174537598	0.2399992	hypothetical protein
CLO1313_RS04170		0.828	-0.273	0.65418973	0.721557558	hypothetical protein
CLO1313_RS04175	Clo1313_0811	1.915	0.937	8.57E-11	3.98E-10	DNA-directed RNA polymerase sigma-70 factor
CLO1313_RS04180	Clo1313_0812	2.299	1.201	6.14E-21	5.32E-20	hypothetical protein
CLO1313_RS04190	Clo1313_0813	1.174	0.232	0.028822166	0.048945456	phosphoglycerate mutase
CLO1313_RS04195	Clo1313_0814	0.989	-0.016	0.876460277	0.902955813	hypothetical protein
CLO1313_RS04200	Clo1313_0815	1.654	0.726	7.31E-06	2.09E-05	short-chain dehydrogenase
CLO1313_RS04205	Clo1313_0816	1.263	0.337	0.021604942	0.037648589	transposase
CLO1313_RS04210	Clo1313_0817	0.813	-0.298	0.00020225	0.000480245	hypothetical protein
CLO1313_RS04215	Clo1313_0818	0.655	-0.611	0.407183986	0.492198619	hypothetical protein
CLO1313_RS04220	Clo1313_0819	1.229	0.298	0.630873526	0.700736928	serine/threonine protein phosphatase
CLO1313_RS04225	Clo1313_0820	0.604	-0.728	2.32E-08	8.47E-08	glycoside hydrolase
CLO1313_RS04230	Clo1313_0821	0.665	-0.589	1.67E-07	5.67E-07	branched-chain amino acid ABC transporter
CLO1313_RS04235	Clo1313_0822	0.808	-0.307	0.007305393	0.013999281	branched-chain amino acid ABC transporter permease
CLO1313_RS04240	Clo1313_0823	1.486	0.571	3.77E-08	1.35E-07	potassium transporter
CLO1313_RS04245	Clo1313_0824	1.039	0.055	0.587071174	0.662636979	lantibiotic ABC transporter permease
CLO1313_RS04250	Clo1313_0825	1.266	0.34	1.73E-05	4.67E-05	hypothetical protein
CLO1313_RS04255	Clo1313_0827	1.019	0.027	0.826609031	0.860503822	transposase
CLO1313_RS04260	Clo1313_0828	0.882	-0.181	0.009068999	0.017062691	hypothetical protein
CLO1313_RS04270	Clo1313_0830	0.787	-0.345	0.000914764	0.001979348	membrane protein
CLO1313_RS04275	Clo1313_0831	0.794	-0.333	0.011585043	0.021325028	hypothetical protein
CLO1313_RS04280	Clo1313_0832	0.86	-0.217	0.08409448	0.126924683	hypothetical protein
CLO1313_RS04285	Clo1313_0833	0.865	-0.209	0.189451848	0.257438194	ABC transporter ATP-binding protein
CLO1313_RS04290	Clo1313_0834	0.736	-0.442	0.025425673	0.043651287	hypothetical protein
CLO1313_RS04295	Clo1313_0835	1.07	0.097	0.48452503	0.565404889	hypothetical protein
CLO1313_RS04305	Clo1313_0837	0.668	-0.582	2.35E-12	1.22E-11	tryptophan synthase subunit alpha
CLO1313_RS04310	Clo1313_0838	0.72	-0.474	3.62E-10	1.58E-09	cation diffusion facilitator transporter
CLO1313_RS04320	Clo1313_0841	1.355	0.438	0.000317547	0.000734818	two-component system response regulator

CLO1313_RS04325	Clo1313_0842	0.765	-0.386	0.193426572	0.261417886	molecular chaperone
CLO1313_RS04330	Clo1313_0843	0.851	-0.233	0.379298457	0.465432108	membrane protein
CLO1313_RS04335	Clo1313_0844	1.169	0.225	0.143032924	0.200259449	spore coat protein
CLO1313_RS04345	Clo1313_0846	2.206	1.141	0.206172326	0.276525406	FeoA family protein
CLO1313_RS04350	Clo1313_0847	0.747	-0.421	0.00425857	0.008469132	DtxR family transcriptional regulator
CLO1313_RS04355	Clo1313_0848	0.902	-0.149	0.061793896	0.097076948	hypothetical protein
CLO1313_RS04360	Clo1313_0849	1.085	0.117	0.191160885	0.258882332	glycosyl hydrolase
CLO1313_RS04365	Clo1313_0850	1.385	0.47	3.93E-07	1.29E-06	hypothetical protein
CLO1313_RS04370	Clo1313_0851	1.227	0.296	0.027854158	0.047382087	xyloglucanase Xgh74A
CLO1313_RS04375	Clo1313_0852	1.213	0.279	0.006861405	0.013182162	glycosyl hydrolase
CLO1313_RS04385	Clo1313_0855	1.316	0.396	2.38E-05	6.35E-05	two-component system response regulator
CLO1313_RS04390	Clo1313_0856	1.328	0.409	3.96E-06	1.17E-05	sensor histidine kinase
CLO1313_RS04395	Clo1313_0857	4.135	2.048	9.02E-43	2.07E-41	2-isopropylmalate synthase
CLO1313_RS04400	Clo1313_0858	1.631	0.706	1.02E-11	5.11E-11	alpha/beta hydrolase
CLO1313_RS04405	Clo1313_0859	1.124	0.169	0.04424906	0.072081983	polynucleotide adenylyltransferase
CLO1313_RS04410		3.069	1.618	3.69E-31	5.08E-30	hypothetical protein
CLO1313_RS04415	Clo1313_0860	0.989	-0.016	0.842721747	0.87420357	hypothetical protein
CLO1313_RS04420	Clo1313_0861	0.939	-0.09	0.223327041	0.296353006	hypothetical protein
CLO1313_RS04425	Clo1313_0862	0.885	-0.176	0.020594122	0.03594981	hypothetical protein
CLO1313_RS04430	Clo1313_0863	0.96	-0.059	0.485046156	0.565792852	protein translocase subunit SecA
CLO1313_RS04435	Clo1313_0864	0.892	-0.164	0.052902477	0.084841993	bifunctional folylpolyglutamate synthase/dihydrofolate synthase
CLO1313_RS04440	Clo1313_0865	1.002	0.004	0.967238376	0.974386258	hypothetical protein
CLO1313_RS04445	Clo1313_0866	1.005	0.007	0.919491244	0.936351186	patatin
CLO1313_RS04450	Clo1313_0867	1.083	0.115	0.089942709	0.133998104	threonine synthase
CLO1313_RS04455	Clo1313_0868	0.737	-0.44	0.002000527	0.00415196	chemotaxis protein CheY
CLO1313_RS04460	Clo1313_0869	1.067	0.093	0.571090817	0.64654638	oxidoreductase
CLO1313_RS04465	Clo1313_0870	15.622	3.966	1.31E-167	3.03E-165	phosphodiesterase
CLO1313_RS04470	Clo1313_0871	0.922	-0.117	0.19026512	0.258192351	hypothetical protein
CLO1313_RS04475	Clo1313_0872	0.895	-0.159	0.062601178	0.098190865	homoserine dehydrogenase

CLO1313_RS04480	Clo1313_0873	1.005	0.008	0.910780828	0.93032415	aspartate kinase
CLO1313_RS04485	Clo1313_0874	0.681	-0.554	0.010887548	0.020168097	copper amine oxidase
CLO1313_RS04490	Clo1313_0875	0.633	-0.66	2.52E-16	1.73E-15	hypothetical protein
CLO1313_RS04495	Clo1313_0876	0.709	-0.497	2.36E-07	7.90E-07	hypothetical protein
CLO1313_RS04500	Clo1313_0877	0.906	-0.142	0.17979151	0.245983002	hypothetical protein
CLO1313_RS04505	Clo1313_0879	0.998	-0.002	0.98540523	0.988468342	hypothetical protein
CLO1313_RS04510	Clo1313_0880	1.434	0.52	6.96E-12	3.51E-11	hypothetical protein
CLO1313_RS04515	Clo1313_0881	0.994	-0.008	0.943524878	0.955310975	hypothetical protein
CLO1313_RS04520	Clo1313_0882	1.091	0.126	0.442737152	0.526892349	hypothetical protein
CLO1313_RS04525	Clo1313_0883	1.092	0.127	0.093567259	0.139052631	hydrolase
CLO1313_RS04530	Clo1313_0884	0.808	-0.308	6.34E-05	0.000160855	hypothetical protein
CLO1313_RS04535	Clo1313_0885	0.994	-0.009	0.940009516	0.95207313	transposase
CLO1313_RS04540	Clo1313_0886	0.566	-0.821	2.84E-13	1.59E-12	phosphoesterase
CLO1313_RS04550	Clo1313_0888	0.902	-0.148	0.127884782	0.182544723	hypothetical protein
CLO1313_RS04555	Clo1313_0889	0.336	-1.575	1.41E-56	5.30E-55	hypothetical protein
CLO1313_RS04560	Clo1313_0890	0.609	-0.717	1.67E-16	1.15E-15	LPS biosynthesis protein
CLO1313_RS04565	Clo1313_0891	0.615	-0.702	2.35E-15	1.55E-14	antitermination factor NusG
CLO1313_RS04570	Clo1313_0892	0.609	-0.715	4.75E-15	3.05E-14	NAD-dependent epimerase
CLO1313_RS04575	Clo1313_0893	0.616	-0.7	4.04E-17	2.88E-16	beta-1,4-galactosyltransferase enhancer
CLO1313_RS04580	Clo1313_0894	0.606	-0.722	8.08E-14	4.75E-13	glycosyl transferase
CLO1313_RS04585	Clo1313_0895	0.651	-0.62	1.48E-09	6.10E-09	glycosyl transferase
CLO1313_RS04590	Clo1313_0896	0.576	-0.795	1.19E-16	8.30E-16	glycosyl transferase family 1
CLO1313_RS04595	Clo1313_0897	0.69	-0.536	1.95E-09	7.94E-09	glycosyl transferase
CLO1313_RS04600	Clo1313_0898	0.63	-0.666	1.44E-10	6.59E-10	glycosyl transferase
CLO1313_RS04605	Clo1313_0899	0.601	-0.735	1.78E-14	1.09E-13	polymerase
CLO1313_RS04610	Clo1313_0900	0.772	-0.373	0.000140958	0.000341464	polysaccharide biosynthesis protein
CLO1313_RS04615	Clo1313_0901	1.16	0.214	0.004903318	0.009668014	UDP-glucose 6-dehydrogenase
CLO1313_RS04620	Clo1313_0902	1.139	0.188	0.02435689	0.04198064	acetobutylicum phosphotransbutyrylase
CLO1313_RS04625	Clo1313_0903	1.111	0.152	0.060218461	0.094950138	single-stranded DNA-binding protein
CLO1313_RS04630	Clo1313_0904	1.019	0.027	0.725595493	0.779950137	UDP-phosphate galactose phosphotransferase

CLO1313_RS04635	Clo1313_0905	0.56	-0.837	5.37E-30	7.19E-29	30S ribosomal protein S21
CLO1313_RS04640	Clo1313_0906	0.558	-0.841	1.31E-23	1.29E-22	aspartyl-tRNA amidotransferase subunit B
CLO1313_RS04650	Clo1313_0908	0.986	-0.021	0.845551462	0.876532608	adenine phosphoribosyltransferase
CLO1313_RS04655	Clo1313_0909	1.147	0.197	0.091813028	0.136580988	(p)ppGpp synthetase
CLO1313_RS04660	Clo1313_0910	1.108	0.148	0.2194357	0.292466787	D-aminoacyl-tRNA deacylase
CLO1313_RS04665	Clo1313_0911	1.123	0.167	0.119705828	0.172512147	MBL fold metallo-hydrolase
CLO1313_RS04675	Clo1313_0913	0.516	-0.953	2.09E-33	3.21E-32	hypothetical protein
CLO1313_RS04680	Clo1313_0914	0.396	-1.336	3.04E-63	1.40E-61	pilus biosynthesis protein
CLO1313_RS04685	Clo1313_0915	0.411	-1.282	1.54E-42	3.49E-41	pilus assembly protein TadB
CLO1313_RS04690	Clo1313_0916	0.421	-1.246	3.41E-31	4.73E-30	type II secretion system protein
CLO1313_RS04695	Clo1313_0917	0.485	-1.044	3.33E-40	7.09E-39	hypothetical protein
CLO1313_RS04700	Clo1313_0918	0.521	-0.942	1.47E-23	1.45E-22	hypothetical protein
CLO1313_RS04705	Clo1313_0919	0.659	-0.602	4.85E-12	2.48E-11	peptidase A24
CLO1313_RS04710	Clo1313_0920	0.497	-1.009	5.38E-26	5.99E-25	hypothetical protein
CLO1313_RS04715	Clo1313_0921	0.908	-0.139	0.120148611	0.172984006	hypothetical protein
CLO1313_RS04720	Clo1313_0922	0.717	-0.48	7.66E-07	2.45E-06	histidine--tRNA ligase
CLO1313_RS04725	Clo1313_0923	0.933	-0.099	0.37301753	0.459228068	aspartyl-tRNA synthetase
CLO1313_RS04735	Clo1313_0925	1.102	0.14	0.136728718	0.192931692	2-hydroxyglutaryl-CoA dehydratase
CLO1313_RS04740	Clo1313_0926	0.828	-0.272	0.14651799	0.20485196	hypothetical protein
CLO1313_RS04745	Clo1313_0927	1.478	0.564	0.071038069	0.10975949	stage II sporulation protein P
CLO1313_RS04750	Clo1313_0928	1.305	0.384	0.299976792	0.382495919	hypothetical protein
CLO1313_RS04755	Clo1313_0929	0.581	-0.784	3.27E-30	4.40E-29	elongation factor 4
CLO1313_RS04760	Clo1313_0930	0.619	-0.691	2.81E-15	1.85E-14	coproporphyrinogen III oxidase
CLO1313_RS04770	Clo1313_0932	1.563	0.645	9.01E-18	6.70E-17	co-chaperone GrpE
CLO1313_RS04775	Clo1313_0933	1.289	0.366	0.000427114	0.000969655	molecular chaperone DnaK
CLO1313_RS04785	Clo1313_0935	1.052	0.074	0.323729854	0.407755494	ribosomal protein L11 methyltransferase
CLO1313_RS04790	Clo1313_0936	1.206	0.27	0.000483534	0.001082986	16S rRNA methyltransferase
CLO1313_RS04795	Clo1313_0937	1.143	0.193	0.028223877	0.047983791	chemotaxis protein CheY
CLO1313_RS04800	Clo1313_0938	1.312	0.392	0.006574918	0.012672351	hypothetical protein
CLO1313_RS04805	Clo1313_0939	1.065	0.091	0.153944567	0.214137179	hypothetical protein

CLO1313_RS04810	Clo1313_0940	1.228	0.297	9.41E-05	0.000234698	hypothetical protein
CLO1313_RS04815	Clo1313_0941	1.086	0.119	0.071801398	0.110767691	guanylate kinase
CLO1313_RS04820	Clo1313_0942	1.031	0.044	0.570973026	0.64654638	DNA-directed RNA polymerase subunit omega
CLO1313_RS04830	Clo1313_0944	0.978	-0.033	0.660390531	0.726260067	glycine--tRNA ligase
CLO1313_RS04835	Clo1313_0945	1.379	0.463	1.96E-08	7.22E-08	signal transduction histidine kinase
CLO1313_RS04840	Clo1313_0947	1.505	0.59	1.04E-07	3.59E-07	accessory gene regulator B
CLO1313_RS04845	Clo1313_0948	3.504	1.809	1.02E-38	2.05E-37	radical SAM protein
CLO1313_RS04850	Clo1313_0949	0.67	-0.577	3.83E-09	1.51E-08	pyruvate phosphate dikinase
CLO1313_RS04855	Clo1313_0950	1.376	0.46	6.57E-12	3.33E-11	cellulosome anchoring protein cohesin region
CLO1313_RS04860	Clo1313_R0031	0.782	-0.355	0.119055544	0.171740056	
CLO1313_RS04865	Clo1313_0951	0.895	-0.16	0.09412956	0.139818995	hypothetical protein
CLO1313_RS04870	Clo1313_0952	0.947	-0.079	0.320914115	0.405353207	cupin
CLO1313_RS04875	Clo1313_0953	0.975	-0.037	0.66472966	0.729846188	phosphate starvation protein PhoH
CLO1313_RS04880	Clo1313_0954	0.856	-0.224	0.008976706	0.016910265	glycosyl transferase family 1
CLO1313_RS04885	Clo1313_0955	0.844	-0.244	0.008583058	0.016250374	ribonuclease J
CLO1313_RS04890	Clo1313_0956	1.141	0.19	0.032277352	0.054259966	hypothetical protein
CLO1313_RS04895	Clo1313_0957	2.69	1.428	0.016779339	0.029970958	hypothetical protein
CLO1313_RS04900	Clo1313_0958	1.59	0.669	0.005491777	0.010722552	hypothetical protein
CLO1313_RS04905	Clo1313_0959	1.891	0.919	0.000182641	0.000437843	50S ribosomal protein L25
CLO1313_RS04915	Clo1313_0961	1.518	0.602	2.23E-10	1.00E-09	hypothetical protein
CLO1313_RS04920	Clo1313_0962	1.495	0.58	2.33E-08	8.49E-08	5-formyltetrahydrofolate cyclo-ligase
CLO1313_RS04925	Clo1313_0964	1.409	0.495	0.000583104	0.00128583	recombinase
CLO1313_RS04930	Clo1313_0965	1.204	0.268	0.038845412	0.06418589	phosphohydrolase
CLO1313_RS04935	Clo1313_0966	0.985	-0.022	0.715334069	0.770300493	phosphoglycerate mutase
CLO1313_RS04940	Clo1313_0967	1.042	0.059	0.379742046	0.46559542	amidohydrolase
CLO1313_RS04945	Clo1313_0968	1.288	0.365	7.30E-06	2.09E-05	hypothetical protein
CLO1313_RS04950	Clo1313_0969	1.038	0.054	0.560594171	0.637550975	hypothetical protein
CLO1313_RS04955	Clo1313_0970	1.083	0.114	0.139699397	0.196141616	two-component system response regulator
CLO1313_RS04960	Clo1313_0971	1.13	0.177	0.022034919	0.03826446	two-component system sensor histidine kinase
CLO1313_RS04965	Clo1313_0972	0.849	-0.236	0.047851976	0.077321162	peptidase S1

CLO1313_RS04970	Clo1313_0973	1.62	0.696	4.74E-07	1.55E-06	phosphohydrolase
CLO1313_RS04975	Clo1313_0974	0.657	-0.605	1.72E-05	4.66E-05	glycogen synthase
CLO1313_RS04985	Clo1313_0976	0.865	-0.21	0.01358664	0.024620141	diguanylate cyclase
CLO1313_RS04990	Clo1313_0977	0.925	-0.113	0.674402759	0.738420545	hypothetical protein
CLO1313_RS04995	Clo1313_0978	1.342	0.424	0.137234208	0.193313945	transglycosylase
CLO1313_RS05005	Clo1313_0980	1.016	0.023	0.737738877	0.789607028	ATP-dependent DNA helicase RecG
CLO1313_RS05015	Clo1313_0982	1.028	0.04	0.607316746	0.680367172	phosphopantetheine adenylyltransferase
CLO1313_RS05020	Clo1313_0983	1.082	0.114	0.131473202	0.186727383	ATPase
CLO1313_RS05030	Clo1313_0985	1.909	0.933	9.34E-14	5.43E-13	alpha-L-arabinofuranosidase
CLO1313_RS05035	Clo1313_0986	2.055	1.039	1.21E-09	5.02E-09	RNA polymerase sigma factor SigI
CLO1313_RS05040	Clo1313_0987	1.914	0.937	2.32E-26	2.65E-25	carbohydrate-binding protein
CLO1313_RS05045	Clo1313_0988	1.045	0.064	0.486993025	0.567622263	proteinase IV
CLO1313_RS05050	Clo1313_0989	1.078	0.109	0.212430705	0.284156861	iron-sulfur cluster-binding protein
CLO1313_RS05055	Clo1313_0990	8.913	3.156	9.38E-48	2.63E-46	histidine kinase
CLO1313_RS05060	Clo1313_0991	11.169	3.481	3.51E-48	1.02E-46	chemotaxis protein CheY
CLO1313_RS05065	Clo1313_0992	1.764	0.818	2.54E-10	1.14E-09	chemotaxis protein
CLO1313_RS05070	Clo1313_0993	0.818	-0.29	9.56E-06	2.69E-05	phosphoglucomutase
CLO1313_RS05075	Clo1313_0994	1.068	0.095	0.341301969	0.426840953	DNA polymerase III subunit alpha
CLO1313_RS05080	Clo1313_0995	1.231	0.3	5.53E-05	0.000141999	tryptophan RNA-binding attenuator protein
CLO1313_RS05085	Clo1313_0996	1.248	0.32	6.86E-06	1.97E-05	hypothetical protein
CLO1313_RS05095	Clo1313_0998	1.123	0.167	0.031685405	0.053414575	4-hydroxybenzoyl-CoA thioesterase
CLO1313_RS05100	Clo1313_0999	1.096	0.132	0.095807092	0.141819087	CDP-alcohol phosphatidyltransferase
CLO1313_RS05105	Clo1313_1000	2.044	1.032	3.73E-35	6.28E-34	copper amine oxidase
CLO1313_RS05110	Clo1313_1001	3.918	1.97	7.83E-93	6.52E-91	carbohydrate-binding protein
CLO1313_RS05115	Clo1313_1002	3.84	1.941	6.86E-91	5.56E-89	thermostable beta-glucosidase B
CLO1313_RS05120	Clo1313_1003	1.299	0.378	0.000460548	0.001039785	hypothetical protein
CLO1313_RS05125	Clo1313_1005	0.887	-0.172	0.016387147	0.029305339	phosphoenolpyruvate synthase
CLO1313_RS05130	Clo1313_1006	0.447	-1.16	6.73E-24	6.75E-23	transporter
CLO1313_RS05135	Clo1313_1007	2.277	1.187	6.80E-24	6.80E-23	guanine permease
CLO1313_RS05145	Clo1313_1009	1.648	0.721	7.28E-07	2.33E-06	amidophosphoribosyltransferase

CLO1313_RS05150	Clo1313_1010	1.514	0.598	3.96E-05	0.000102931	phosphoribosylformylglycinamidine cyclo-ligase
CLO1313_RS05155	Clo1313_1011	1.492	0.577	2.97E-05	7.81E-05	phosphoribosylglycinamide formyltransferase
CLO1313_RS05165	Clo1313_1013	1.411	0.497	4.67E-06	1.38E-05	phosphoribosylamine--glycine ligase
CLO1313_RS05170	Clo1313_1014	2.199	1.137	2.06E-21	1.86E-20	glycosyl transferase
CLO1313_RS05175	Clo1313_1015	0.471	-1.088	2.67E-06	8.03E-06	GCN5 family N-acetyltransferase
CLO1313_RS05180	Clo1313_1016	0.765	-0.386	6.85E-07	2.21E-06	nicotinate-nucleotide adenylyltransferase
CLO1313_RS05185	Clo1313_1017	0.873	-0.195	0.007529092	0.014400348	phosphohydrolase
CLO1313_RS05190	Clo1313_1018	0.71	-0.493	2.64E-09	1.06E-08	hypothetical protein
CLO1313_RS05200	Clo1313_1020	0.703	-0.508	2.18E-13	1.23E-12	leucine--tRNA ligase
CLO1313_RS05205	Clo1313_1021	0.535	-0.901	5.26E-25	5.63E-24	cellulose 1,4-beta-cellobiosidase
CLO1313_RS05210	Clo1313_1022	0.974	-0.037	0.917042136	0.9344918	chemotaxis protein
CLO1313_RS05215	Clo1313_1023	9.333	3.222	8.29E-09	3.18E-08	hypothetical protein
CLO1313_RS05220	Clo1313_1024	0.668	-0.581	5.67E-17	4.02E-16	AMP-dependent synthetase
CLO1313_RS05225	Clo1313_1025	0.744	-0.428	4.19E-08	1.49E-07	serine-type D-Ala-D-Ala carboxypeptidase
CLO1313_RS05230	Clo1313_1026	0.549	-0.865	0.00182713	0.003815851	competence protein ComEA
CLO1313_RS05235	Clo1313_1027	1.077	0.107	0.171978423	0.237023571	sporulation protein
CLO1313_RS05240	Clo1313_1029	0.666	-0.586	1.88E-10	8.49E-10	threonine--tRNA ligase
CLO1313_RS05245		0.824	-0.279	0.030680495	0.051924833	hypothetical protein
CLO1313_RS05255	Clo1313_1031	0.872	-0.198	0.014443034	0.026014809	dihydrofolate reductase
CLO1313_RS05260	Clo1313_1032	0.463	-1.111	3.52E-33	5.39E-32	translation initiation factor IF-3
CLO1313_RS05265	Clo1313_1033	0.521	-0.939	9.33E-28	1.14E-26	50S ribosomal protein L35
CLO1313_RS05270	Clo1313_1034	0.549	-0.864	6.47E-27	7.57E-26	50S ribosomal protein L20
CLO1313_RS05275	Clo1313_1035	1.353	0.437	6.40E-05	0.000162067	RNA methyltransferase
CLO1313_RS05280	Clo1313_1036	1.27	0.345	2.90E-05	7.65E-05	glycosyl transferase
CLO1313_RS05285	Clo1313_1037	0.975	-0.037	0.649571165	0.717254758	hypothetical protein
CLO1313_RS05290	Clo1313_1038	0.877	-0.189	0.053110177	0.085129568	hypothetical protein
CLO1313_RS05295	Clo1313_1039	0.758	-0.4	2.74E-05	7.25E-05	leucyl/phenylalanyl-tRNA--protein transferase
CLO1313_RS05305	Clo1313_1041	0.988	-0.018	0.830823221	0.863353721	ATP-dependent Clp protease adapter protein ClpS
CLO1313_RS05310	Clo1313_1042	0.505	-0.987	0.086670812	0.13015812	permease
CLO1313_RS05315	Clo1313_1043	0.574	-0.802	0.047678636	0.07712418	permease

CLO1313_RS05320	Clo1313_1044	0.702	-0.51	5.95E-05	0.00015216	hypothetical protein
CLO1313_RS05325	Clo1313_1045	1.442	0.529	1.90E-07	6.41E-07	hypothetical protein
CLO1313_RS05330	Clo1313_1046	1.095	0.131	0.201165998	0.270052295	tryptophan synthase subunit beta
CLO1313_RS05335	Clo1313_1047	0.955	-0.066	0.68372842	0.745606087	hypothetical protein
CLO1313_RS05340	Clo1313_1048	1.287	0.364	0.00012732	0.00031018	hypothetical protein
CLO1313_RS05345	Clo1313_1049	0.824	-0.279	0.098517866	0.145115462	GCN5 family N-acetyltransferase
CLO1313_RS05350	Clo1313_1051	1.156	0.21	0.009742966	0.018216431	membrane protein
CLO1313_RS05355	Clo1313_1052	1.082	0.114	0.547223689	0.62614416	serine protease
CLO1313_RS05360	Clo1313_1053	1.572	0.652	1.72E-05	4.67E-05	hypothetical protein
CLO1313_RS05365	Clo1313_1054	1.752	0.809	3.44E-09	1.37E-08	stage V sporulation protein R
CLO1313_RS05370	Clo1313_1055	0.609	-0.716	6.81E-11	3.20E-10	MFS transporter
CLO1313_RS05375	Clo1313_1056	0.755	-0.406	1.82E-08	6.71E-08	purine nucleoside phosphorylase
CLO1313_RS05380	Clo1313_1057	0.923	-0.115	0.142298574	0.199417488	S-adenosyl-L-homocysteine hydrolase
CLO1313_RS05385	Clo1313_1058	0.941	-0.088	0.227062637	0.30077776	5-methylthioadenosine/S-adenosylhomocysteine deaminase
CLO1313_RS05390	Clo1313_R0032	0.974	-0.038	0.72615964	0.780067547	
CLO1313_RS05405		1.293	0.371	0.010472865	0.019459803	hypothetical protein
CLO1313_RS05415		1.291	0.369	0.020292776	0.035485735	hypothetical protein
CLO1313_RS05420	Clo1313_1065	1.447	0.533	3.50E-06	1.04E-05	hypothetical protein
CLO1313_RS05425	Clo1313_1066	1.584	0.663	1.46E-09	6.04E-09	transposase
CLO1313_RS05430	Clo1313_1067	0.779	-0.36	0.000303929	0.000704936	hypothetical protein
CLO1313_RS05435	Clo1313_1068	1.372	0.456	0.001883994	0.003918238	haloacid dehalogenase
CLO1313_RS05440	Clo1313_1069	1.301	0.38	0.004497288	0.008920217	hypothetical protein
CLO1313_RS05445	Clo1313_1070	0.92	-0.121	0.196435461	0.264887567	ABC transporter
CLO1313_RS05450	Clo1313_1071	0.958	-0.062	0.458255384	0.541279204	multidrug ABC transporter permease
CLO1313_RS05455	Clo1313_1072	0.934	-0.098	0.224656098	0.297984802	hypothetical protein
CLO1313_RS05460	Clo1313_1073	2.011	1.008	1.10E-36	2.00E-35	alpha/beta hydrolase
CLO1313_RS05465	Clo1313_1074	1.188	0.249	0.000355711	0.000815579	hypothetical protein
CLO1313_RS05470	Clo1313_1075	1.153	0.205	0.005827527	0.011326477	hypothetical protein
CLO1313_RS05475	Clo1313_1076	0.897	-0.158	0.040546487	0.0667759	magnesium chelatase
CLO1313_RS05480	Clo1313_1077	0.908	-0.139	0.072607761	0.111724307	hypothetical protein

CLO1313_RS05485	Clo1313_1078	1.134	0.182	0.013326034	0.024191753	transglutaminase
CLO1313_RS05490	Clo1313_1079	1.139	0.188	0.129129055	0.183970563	hypothetical protein
CLO1313_RS05495	Clo1313_1080	1.45	0.536	0.004469297	0.008870563	small, acid-soluble spore protein H
CLO1313_RS05500	Clo1313_1081	1.13	0.176	0.085109977	0.128199307	isochorismatase
CLO1313_RS05505	Clo1313_1082	1.064	0.089	0.25002321	0.328292297	nicotinate phosphoribosyltransferase
CLO1313_RS05510	Clo1313_1083	4.514	2.174	1.26E-168	3.43E-166	chemotaxis protein CheC
CLO1313_RS05515	Clo1313_1084	0.66	-0.6	3.67E-05	9.57E-05	hypothetical protein
CLO1313_RS05520	Clo1313_1085	0.547	-0.869	1.45E-10	6.64E-10	hypothetical protein
CLO1313_RS05525	Clo1313_1086	1.356	0.439	1.53E-05	4.18E-05	pilus assembly protein PilZ
CLO1313_RS05530	Clo1313_1087	0.969	-0.046	0.625012169	0.69551447	hypothetical protein
CLO1313_RS05535	Clo1313_1089	1.155	0.208	0.017142103	0.030564309	D-alanyl-D-alanine carboxypeptidase
CLO1313_RS05545	Clo1313_1091	0.842	-0.249	0.030548583	0.051730774	serine hydrolase
CLO1313_RS05550	Clo1313_1093	0.703	-0.508	2.62E-06	7.86E-06	hypothetical protein
CLO1313_RS05560	Clo1313_1095	0.801	-0.32	0.000115795	0.000283947	membrane protein
CLO1313_RS05565	Clo1313_1096	0.81	-0.304	4.29E-05	0.000111136	hypothetical protein
CLO1313_RS05570	Clo1313_1097	0.958	-0.062	0.478119807	0.558671822	hypothetical protein
CLO1313_RS05575	Clo1313_1098	0.966	-0.05	0.569313427	0.645508872	phosphoglucosamine mutase
CLO1313_RS05585	Clo1313_1100	1.309	0.388	0.005825885	0.011326477	ArsR family transcriptional regulator
CLO1313_RS05595	Clo1313_1102	0.928	-0.108	0.495269111	0.574733082	protein-tyrosine phosphatase
CLO1313_RS05600	Clo1313_1103	0.924	-0.113	0.418949846	0.503782657	permease
CLO1313_RS05610		1.247	0.318	0.000229363	0.000540771	integrase
CLO1313_RS05615	Clo1313_1105	1.35	0.433	0.035024157	0.058386573	hypothetical protein
CLO1313_RS05620	Clo1313_1106	0.98	-0.029	0.684532943	0.745700797	type II secretion system protein E
CLO1313_RS05625	Clo1313_1107	0.901	-0.15	0.045732765	0.074176615	twitching motility protein PilT
CLO1313_RS05630	Clo1313_1108	0.731	-0.452	4.57E-08	1.63E-07	secretion system protein
CLO1313_RS05635	Clo1313_1109	1.163	0.218	0.00258958	0.00531564	prepilin cleavage protein
CLO1313_RS05640	Clo1313_1110	0.74	-0.435	1.95E-08	7.18E-08	prepilin cleavage protein
CLO1313_RS05645	Clo1313_1111	0.774	-0.371	1.38E-05	3.80E-05	fimbrial protein
CLO1313_RS05650	Clo1313_1112	0.769	-0.378	4.09E-07	1.34E-06	hypothetical protein
CLO1313_RS05655	Clo1313_1113	0.792	-0.336	2.39E-05	6.37E-05	prepilin cleavage protein

CLO1313_RS05660	Clo1313_1114	0.723	-0.468	1.51E-10	6.87E-10	hypothetical protein
CLO1313_RS05665	Clo1313_1115	0.832	-0.265	0.001259504	0.002686524	hypothetical protein
CLO1313_RS05670	Clo1313_1116	0.912	-0.133	0.309264785	0.392171286	translocation-enhancing protein TepA
CLO1313_RS05675	Clo1313_1117	0.996	-0.006	0.978133728	0.983380171	hypothetical protein
CLO1313_RS05680	Clo1313_1118	1.101	0.139	0.275839133	0.35764875	cell division protein FtsK
CLO1313_RS05685	Clo1313_1119	1.248	0.32	0.002127879	0.004394979	radical SAM protein
CLO1313_RS05690	Clo1313_1120	1.004	0.006	0.925363062	0.940733499	bifunctional protein FolD
CLO1313_RS05695	Clo1313_1121	0.954	-0.068	0.32536751	0.40947426	ATPase
CLO1313_RS05705	Clo1313_1123	0.967	-0.048	0.614047575	0.686032026	metallophosphoesterase
CLO1313_RS05710	Clo1313_1124	1.759	0.815	3.32E-19	2.66E-18	stage V sporulation protein S
CLO1313_RS05715	Clo1313_1125	1.198	0.26	0.027820505	0.047378589	hypothetical protein
CLO1313_RS05720	Clo1313_1126	1.489	0.574	0.010432074	0.019396027	hypothetical protein
CLO1313_RS05725	Clo1313_1127	1.677	0.746	0.001426469	0.003023307	spore coat protein
CLO1313_RS05730	Clo1313_1128	1.442	0.528	0.008566083	0.016228479	glycosyl transferase family 1
CLO1313_RS05735	Clo1313_1129	1.33	0.411	0.061220152	0.096359488	spore coat protein
CLO1313_RS05740	Clo1313_1130	1.121	0.165	0.42584177	0.510635533	homoserine kinase
CLO1313_RS05745	Clo1313_1131	1.423	0.509	0.053635551	0.085834054	transcription initiation factor IIE
CLO1313_RS05750	Clo1313_1132	1.791	0.84	0.024317207	0.041936345	hypothetical protein
CLO1313_RS05755	Clo1313_1133	0.651	-0.62	0.31197137	0.395389551	carbohydrate-binding protein
CLO1313_RS05760	Clo1313_1134	1.233	0.302	0.099029477	0.145725909	nucleotidyltransferase
CLO1313_RS05765	Clo1313_1135	1.295	0.373	0.012487881	0.022822155	hypothetical protein
CLO1313_RS05770	Clo1313_1136	0.581	-0.784	0.136542873	0.192800867	hypothetical protein
CLO1313_RS05775	Clo1313_1137	0.92	-0.121	0.200807021	0.269811943	hypothetical protein
CLO1313_RS05780	Clo1313_1138	0.963	-0.054	0.476515569	0.557577132	hypothetical protein
CLO1313_RS05800	Clo1313_1142	1.092	0.127	0.07780908	0.11893447	phosphate starvation-inducible protein PhoH
CLO1313_RS05805	Clo1313_1143	1.015	0.021	0.742334752	0.793676264	phosphohydrolase
CLO1313_RS05810	Clo1313_1144	1.015	0.021	0.780475847	0.82620793	endoribonuclease YbeY
CLO1313_RS05820	Clo1313_1146	1.014	0.02	0.759939841	0.807890671	GTPase Era
CLO1313_RS05830	Clo1313_1149	1.554	0.636	5.38E-13	2.95E-12	pilus assembly protein PilZ
CLO1313_RS05835	Clo1313_1150	0.905	-0.144	0.033437744	0.056059744	cysteine desulfurase

CLO1313_RS05840	Clo1313_1151	0.845	-0.243	0.000214541	0.000507821	thiamine biosynthesis protein ThiI
CLO1313_RS05845	Clo1313_1152	0.734	-0.446	3.21E-06	9.56E-06	vanomycin resistance protein VanB
CLO1313_RS05850	Clo1313_1153	2.096	1.068	1.12E-14	6.97E-14	hypothetical protein
CLO1313_RS05855	Clo1313_1154	0.932	-0.102	0.373659939	0.459830183	AIR synthase
CLO1313_RS05865	Clo1313_1155	0.431	-1.213	9.04E-25	9.42E-24	serine hydroxymethyltransferase
CLO1313_RS05870	Clo1313_1156	0.646	-0.631	2.29E-10	1.03E-09	phage-shock protein
CLO1313_RS05875	Clo1313_1157	0.649	-0.623	1.87E-17	1.36E-16	transglutaminase
CLO1313_RS05880	Clo1313_1158	0.67	-0.578	4.00E-13	2.22E-12	hypothetical protein
CLO1313_RS05885	Clo1313_1159	0.793	-0.334	2.48E-05	6.60E-05	magnesium chelatase
CLO1313_RS05890	Clo1313_1160	0.722	-0.47	3.53E-09	1.40E-08	L-lactate dehydrogenase
CLO1313_RS05895	Clo1313_1161	1.02	0.029	0.778802216	0.825310193	damage-inducible protein CinA
CLO1313_RS05910	Clo1313_1164	1.091	0.125	0.136881881	0.192998948	recombinase RecX
CLO1313_RS05915	Clo1313_1165	0.987	-0.019	0.789505041	0.832533621	peptidase S54
CLO1313_RS05920	Clo1313_1166	1.206	0.27	4.70E-05	0.000121182	hypothetical protein
CLO1313_RS05925	Clo1313_1167	2.206	1.141	4.32E-21	3.81E-20	ABC transporter substrate-binding protein
CLO1313_RS05930	Clo1313_1168	1.38	0.465	0.00073186	0.001599745	hypothetical protein
CLO1313_RS05935	Clo1313_1169	1.239	0.31	0.013221655	0.02403136	hypothetical protein
CLO1313_RS05940	Clo1313_1170	0.705	-0.503	0.000283113	0.000661257	hypothetical protein
CLO1313_RS05945	Clo1313_R0033	0.312	-1.68	9.94E-05	0.00024677	
CLO1313_RS05950	Clo1313_1171	1.491	0.576	1.71E-07	5.78E-07	nucleotidyltransferase
CLO1313_RS05955	Clo1313_1172	0.735	-0.443	1.00E-06	3.16E-06	UDP-N-acetylmuramoylalanine--D-glutamate ligase
CLO1313_RS05960	Clo1313_R0034	0.328	-1.607	5.62E-16	3.82E-15	
CLO1313_RS05965	Clo1313_R0035	0.46	-1.119	2.72E-11	1.31E-10	
CLO1313_RS05970	Clo1313_R0036	0.532	-0.91	6.71E-09	2.60E-08	
CLO1313_RS05975	Clo1313_1173	1.069	0.096	0.294969396	0.378201462	DNA polymerase III subunit delta
CLO1313_RS05985	Clo1313_1175	1.813	0.858	0.03176189	0.053513431	GPR endopeptidase
CLO1313_RS05990	Clo1313_1176	1.428	0.514	6.24E-10	2.67E-09	cell wall hydrolase
CLO1313_RS05995	Clo1313_1177	1.072	0.1	0.362578198	0.44932728	hypothetical protein
CLO1313_RS06000	Clo1313_1178	1.022	0.032	0.689436635	0.749829802	DNA ligase
CLO1313_RS06005		1.842	0.882	1.31E-17	9.67E-17	hypothetical protein

CLO1313_RS06010		0.813	-0.299	0.006633568	0.012776049	aspartyl/glutamyl-tRNA(Asn/Gln) amidotransferase subunit C
CLO1313_RS06015		0.722	-0.469	2.92E-06	8.73E-06	glutamyl-tRNA(Gln) amidotransferase
CLO1313_RS06020	Clo1313_1183	0.627	-0.673	1.12E-12	5.96E-12	aspartyl/glutamyl-tRNA amidotransferase subunit B
CLO1313_RS06025	Clo1313_1184	0.802	-0.318	0.000606353	0.001334155	hypothetical protein
CLO1313_RS06035	Clo1313_1186	0.315	-1.665	5.28E-48	1.51E-46	acetate kinase
CLO1313_RS06040	Clo1313_1187	0.589	-0.763	1.48E-18	1.13E-17	hypothetical protein
CLO1313_RS06045	Clo1313_1188	0.8	-0.322	2.25E-07	7.53E-07	50S ribosomal protein L32
CLO1313_RS06050	Clo1313_1189	0.888	-0.172	0.038889961	0.064224115	radical SAM protein
CLO1313_RS06055	Clo1313_1190	0.968	-0.046	0.583404406	0.65899428	GTPase Der
CLO1313_RS06065	Clo1313_1192	0.97	-0.044	0.570391907	0.646487275	glycerol-3-phosphate dehydrogenase
CLO1313_RS06075	Clo1313_1194	0.644	-0.636	8.05E-23	7.69E-22	ABC transporter substrate-binding protein
CLO1313_RS06080	Clo1313_1195	0.637	-0.652	3.27E-15	2.13E-14	ABC transporter permease
CLO1313_RS06085	Clo1313_1196	0.662	-0.596	5.15E-14	3.06E-13	sugar ABC transporter permease
CLO1313_RS06090	Clo1313_1197	1.324	0.404	0.000218688	0.000517228	hypothetical protein
CLO1313_RS06095	Clo1313_1198	2.278	1.188	3.03E-52	9.47E-51	N-acetylmuramoyl-L-alanine amidase
CLO1313_RS06100	Clo1313_1199	1.377	0.462	1.80E-07	6.07E-07	hypothetical protein
CLO1313_RS06105	Clo1313_1200	1.563	0.644	2.53E-11	1.22E-10	hypothetical protein
CLO1313_RS06110	Clo1313_1201	1.035	0.05	0.44782716	0.532316153	DNA mismatch repair protein MutS
CLO1313_RS06125	Clo1313_1204	1.027	0.038	0.869504141	0.896713521	peptidoglycan glycosyltransferase
CLO1313_RS06130	Clo1313_1205	0.917	-0.125	0.163778992	0.226869837	peptidase U32
CLO1313_RS06135	Clo1313_1206	0.95	-0.074	0.305945617	0.388619612	O-methyltransferase
CLO1313_RS06140	Clo1313_1207	0.875	-0.193	0.00329148	0.006665192	aminodeoxychorismate lyase
CLO1313_RS06160	Clo1313_1211	0.478	-1.065	3.37E-25	3.63E-24	uridylate kinase
CLO1313_RS06165	Clo1313_1212	0.562	-0.832	1.35E-18	1.04E-17	ribosome recycling factor
CLO1313_RS06170	Clo1313_1213	0.533	-0.908	5.43E-22	5.03E-21	hypothetical protein
CLO1313_RS06175	Clo1313_1214	0.669	-0.581	2.81E-16	1.92E-15	UDP pyrophosphate synthase
CLO1313_RS06180	Clo1313_1215	0.675	-0.567	3.36E-13	1.88E-12	phosphatidate cytidylyltransferase
CLO1313_RS06185	Clo1313_1216	0.714	-0.486	2.70E-12	1.40E-11	1-deoxy-D-xylulose 5-phosphate reductoisomerase
CLO1313_RS06195	Clo1313_1218	1.08	0.11	0.210246304	0.281486012	4-hydroxy-3-methylbut-2-en-1-yl diphosphate synthase

CLO1313_RS06205	Clo1313_1221	0.494	-1.017	9.98E-39	2.02E-37	ribosome maturation factor RimP
CLO1313_RS06215	Clo1313_1223	0.858	-0.22	0.057066561	0.090647573	hypothetical protein
CLO1313_RS06220	Clo1313_1224	0.9	-0.151	0.278496728	0.360005038	50S ribosomal protein L7ae
CLO1313_RS06225	Clo1313_1225	0.915	-0.127	0.248982229	0.327211965	translation initiation factor IF-2
CLO1313_RS06230	Clo1313_1226	0.953	-0.07	0.545742706	0.625046653	ribosome-binding factor A
CLO1313_RS06235	Clo1313_1227	0.903	-0.147	0.138368378	0.19463732	phosphoesterase
CLO1313_RS06240	Clo1313_1228	0.752	-0.41	2.08E-07	6.99E-07	tRNA pseudouridine synthase B
CLO1313_RS06245	Clo1313_1229	0.849	-0.236	0.006029701	0.011689123	bifunctional riboflavin kinase/FMN adenylyltransferase
CLO1313_RS06250	Clo1313_1231	0.77	-0.378	6.02E-07	1.95E-06	peptidase M16
CLO1313_RS06255	Clo1313_1232	0.764	-0.387	2.92E-06	8.74E-06	zinc protease
CLO1313_RS06260	Clo1313_1233	0.888	-0.172	0.069176572	0.107214749	prolipoprotein diacylglyceryl transferase
CLO1313_RS06265	Clo1313_1235	1.176	0.234	0.011837941	0.021713752	rod shape-determining protein RodA
CLO1313_RS06270	Clo1313_1236	1.21	0.274	3.43E-05	8.98E-05	transcriptional regulator MraZ
CLO1313_RS06275	Clo1313_1237	1.143	0.193	0.005145886	0.010099812	16S rRNA (cytosine(1402)-N(4))-methyltransferase
CLO1313_RS06280	Clo1313_1238	1.132	0.179	0.020557497	0.035906775	cell division protein FtsL
CLO1313_RS06285	Clo1313_1239	0.989	-0.016	0.861301763	0.89005488	peptidoglycan glycosyltransferase
CLO1313_RS06290	Clo1313_1240	0.967	-0.049	0.551092615	0.629474098	UDP-N-acetylmuramyl peptide synthase
CLO1313_RS06295	Clo1313_1241	0.947	-0.078	0.418402656	0.503527113	UDP-N-acetylmuramoyl-tripeptide--D-alanyl-D- alanine ligase
CLO1313_RS06300	Clo1313_1242	0.901	-0.15	0.069130042	0.107198034	phospho-N-acetylmuramoyl-pentapeptide transferase
CLO1313_RS06305	Clo1313_1243	0.95	-0.073	0.291023417	0.374101684	stage V sporulation protein E
CLO1313_RS06315	Clo1313_1245	1.46	0.546	0.087122247	0.130639809	UDP-N-acetylglucosamine 1-carboxyvinyltransferase
CLO1313_RS06320	Clo1313_1246	1.363	0.447	0.094911889	0.140632784	hypothetical protein
CLO1313_RS06325		1.689	0.756	2.85E-10	1.26E-09	hypothetical protein
CLO1313_RS06330	Clo1313_1248	1.51	0.594	1.30E-07	4.44E-07	transposase
CLO1313_RS06335		1.169	0.225	0.34208187	0.427459803	cyclase
CLO1313_RS06340	Clo1313_1249	0.569	-0.814	1.32E-14	8.17E-14	helicase UvrD
CLO1313_RS06345	Clo1313_R0037	0.292	-1.776	1.87E-27	2.27E-26	
CLO1313_RS06350	Clo1313_1250	0.925	-0.113	0.870551546	0.897176662	copper amine oxidase

CLO1313_RS06355	Clo1313_1251	0.851	-0.234	0.016903493	0.030174747	cob(I)alamin adenolsyltransferase/cobinamide ATP-dependent adenolsyltransferase
CLO1313_RS06360	Clo1313_1252	1.415	0.501	4.35E-05	0.000112416	amino acid-binding protein
CLO1313_RS06365	Clo1313_1253	1.375	0.46	0.000461125	0.001039785	4-hydroxy-tetrahydrodipicolinate reductase
CLO1313_RS06370	Clo1313_1254	1.27	0.345	0.008841155	0.016686359	4-hydroxy-tetrahydrodipicolinate synthase
CLO1313_RS06375	Clo1313_1255	1.26	0.334	0.009921878	0.018527841	semialdehyde dehydrogenase
CLO1313_RS06380	Clo1313_1256	0.713	-0.488	8.62E-07	2.74E-06	stage II sporulation protein SpoIID
CLO1313_RS06385	Clo1313_1257	0.93	-0.104	0.271724053	0.353076445	S-adenosylmethionine:tRNA ribosyltransferase-isomerase
CLO1313_RS06390	Clo1313_1258	1.093	0.128	0.285319612	0.36803162	queuine tRNA-ribosyltransferase
CLO1313_RS06400	Clo1313_1260	1.164	0.22	0.064106017	0.100080138	hypothetical protein
CLO1313_RS06405	Clo1313_1261	1.573	0.654	0.027126652	0.046302123	hypothetical protein
CLO1313_RS06410	Clo1313_1262	0.913	-0.131	0.292009037	0.375207842	bifunctional protein PyrR
CLO1313_RS06415	Clo1313_1263	0.927	-0.109	0.492823022	0.572859009	helicase
CLO1313_RS06420	Clo1313_1264	1.3	0.379	0.019326759	0.033994693	aspartate carbamoyltransferase
CLO1313_RS06425	Clo1313_1265	1.246	0.318	0.061961296	0.097288967	dihydroorotase
CLO1313_RS06435	Clo1313_1267	1.331	0.412	0.012639906	0.023085919	carbamoyl phosphate synthase small subunit
CLO1313_RS06440	Clo1313_1268	1.147	0.198	0.284942378	0.367703181	carbamoyl phosphate synthase large subunit
CLO1313_RS06445	Clo1313_1269	1.189	0.25	0.179507214	0.245706132	diguanylate cyclase
CLO1313_RS06450	Clo1313_1270	1.394	0.48	0.003022526	0.006132988	diguanylate cyclase
CLO1313_RS06455	Clo1313_1271	1.261	0.335	0.00147348	0.003112614	phosphoglycerate mutase
CLO1313_RS06460	Clo1313_1272	1.27	0.345	0.002697676	0.005528362	metallophosphoesterase
CLO1313_RS06465	Clo1313_1273	1.116	0.159	0.215803132	0.288410692	chromosome segregation protein SMC
CLO1313_RS06470	Clo1313_1274	1.07	0.097	0.439220982	0.523538842	viral A-type inclusion protein
CLO1313_RS06485	Clo1313_1277	1.095	0.131	0.331846218	0.416927863	hypothetical protein
CLO1313_RS06490	Clo1313_1278	1.056	0.079	0.598435812	0.671923998	hypothetical protein
CLO1313_RS06495	Clo1313_1279	0.419	-1.255	2.76E-21	2.47E-20	fatty acid biosynthesis transcriptional regulator
CLO1313_RS06500	Clo1313_1280	0.503	-0.991	4.34E-10	1.87E-09	phosphate acyltransferase
CLO1313_RS06505	Clo1313_1281	0.589	-0.764	7.14E-05	0.000180285	3-oxoacyl-ACP synthase
CLO1313_RS06530	Clo1313_1286	0.751	-0.412	6.04E-05	0.000153915	ribonuclease III
CLO1313_RS06535	Clo1313_1287	0.69	-0.535	3.13E-07	1.04E-06	radical SAM protein

CLO1313_RS06540	Clo1313_1288	1.269	0.344	0.000466392	0.001049295	stage V sporulation protein S
CLO1313_RS06545	Clo1313_1289	1.146	0.196	0.051976674	0.083446491	4-hydroxythreonine-4-phosphate dehydrogenase
CLO1313_RS06555	Clo1313_1291	0.966	-0.05	0.530221106	0.609013059	signal recognition particle-docking protein FtsY
CLO1313_RS06560	Clo1313_1292	0.85	-0.234	0.014784897	0.026550843	hypothetical protein
CLO1313_RS06565	Clo1313_1293	0.813	-0.298	0.011593383	0.021325028	hypothetical protein
CLO1313_RS06570	Clo1313_1294	0.947	-0.079	0.662344444	0.72776314	hypothetical protein
CLO1313_RS06575	Clo1313_1295	0.513	-0.964	3.42E-45	8.41E-44	diaminopimelate dehydrogenase
CLO1313_RS06580	Clo1313_1296	0.525	-0.929	5.61E-19	4.45E-18	hypothetical protein
CLO1313_RS06585	Clo1313_1297	0.647	-0.628	5.05E-08	1.79E-07	hypothetical protein
CLO1313_RS06590		1.268	0.342	0.000225276	0.000531972	integrase
CLO1313_RS06600	Clo1313_1299	0.594	-0.752	9.23E-05	0.000230829	fibronectin type III domain-containing protein
CLO1313_RS06605	Clo1313_1300	1.186	0.246	0.077913993	0.119034165	dockerin
CLO1313_RS06610	Clo1313_1301	0.79	-0.34	2.93E-05	7.70E-05	glutamine--tRNA ligase
CLO1313_RS06615	Clo1313_1302	0.866	-0.207	0.008361726	0.015861364	hypothetical protein
CLO1313_RS06620	Clo1313_1303	0.99	-0.015	0.870464931	0.897176662	hypothetical protein
CLO1313_RS06625	Clo1313_1304	0.966	-0.05	0.504834885	0.583879606	hypothetical protein
CLO1313_RS06630	Clo1313_1305	1.154	0.206	0.001751399	0.00366635	glycoside hydrolase
CLO1313_RS06635	Clo1313_1306	1.134	0.181	0.019011699	0.033558614	polyphenol oxidase
CLO1313_RS06640	Clo1313_1308	1.105	0.144	0.05837194	0.092525078	ABC transporter substrate-binding protein
CLO1313_RS06650	Clo1313_1310	0.859	-0.219	0.00334564	0.006765728	RNA pseudouridine synthase
CLO1313_RS06660	Clo1313_1313	0.787	-0.345	6.35E-07	2.06E-06	phosphohydrolase
CLO1313_RS06680	Clo1313_1317	1.31	0.389	4.85E-05	0.000125008	pantothenate synthetase
CLO1313_RS06685	Clo1313_1318	1.297	0.375	0.000325991	0.000752615	aspartate 1-decarboxylase
CLO1313_RS06690	Clo1313_1319	0.965	-0.051	0.63471976	0.704487254	hypothetical protein
CLO1313_RS06695	Clo1313_1320	3.689	1.883	6.40E-48	1.81E-46	phosphohydrolase
CLO1313_RS06700	Clo1313_1321	0.919	-0.121	0.293129964	0.376325668	deoxyguanosinetriphosphate triphosphohydrolase
CLO1313_RS06705	Clo1313_1323	1	0	0.997804383	0.998137207	DNA primase
CLO1313_RS06710	Clo1313_1324	1.151	0.203	0.005456676	0.010660959	RNA polymerase sigma factor RpoD
CLO1313_RS06715	Clo1313_1325	1.259	0.332	0.036311949	0.060432595	rubrerythrin
CLO1313_RS06720	Clo1313_R0038	0.578	-0.791	0.006059756	0.011739798	

CLO1313_RS06725	Clo1313_R0039	0.508	-0.978	1.94E-07	6.51E-07	
CLO1313_RS06730	Clo1313_1327	0.875	-0.192	0.010887679	0.020168097	hypothetical protein
CLO1313_RS06735	Clo1313_1328	0.987	-0.019	0.796147451	0.839242955	DUF34 domain-containing protein
CLO1313_RS06740	Clo1313_1329	1.574	0.654	1.17E-09	4.85E-09	chemotaxis protein CheY
CLO1313_RS06745	Clo1313_1330	0.806	-0.311	0.00106909	0.002300001	RNA polymerase subunit sigma-24
CLO1313_RS06750	Clo1313_1331	0.85	-0.235	0.006835207	0.013148676	anti-sigma factor
CLO1313_RS06755	Clo1313_1332	0.777	-0.363	1.99E-05	5.36E-05	pilus assembly protein PilZ
CLO1313_RS06760	Clo1313_1333	0.824	-0.28	0.014276243	0.025760801	hypothetical protein
CLO1313_RS06765	Clo1313_1334	0.816	-0.293	5.80E-05	0.00014877	DNA polymerase I
CLO1313_RS06770	Clo1313_1335	0.797	-0.327	0.000386813	0.00088284	dephospho-CoA kinase
CLO1313_RS06775	Clo1313_1336	0.893	-0.163	0.062561972	0.098180718	lytic transglycosylase
CLO1313_RS06780	Clo1313_1337	1.389	0.475	4.40E-10	1.89E-09	flagellar protein
CLO1313_RS06785	Clo1313_1338	0.986	-0.02	0.843127228	0.87432177	hypothetical protein
CLO1313_RS06790	Clo1313_1339	0.935	-0.098	0.276965635	0.358644188	diguanylate cyclase
CLO1313_RS06800	Clo1313_1341	1.19	0.251	0.003246261	0.006578065	chemotaxis protein CheW
CLO1313_RS06805	Clo1313_1342	0.739	-0.437	9.36E-11	4.34E-10	GTP-binding protein
CLO1313_RS06810	Clo1313_1343	1.086	0.119	0.101330746	0.148747384	glycosyl transferase
CLO1313_RS06820	Clo1313_1345	1.295	0.373	0.011904618	0.021822708	anthranilate synthase subunit II
CLO1313_RS06830	Clo1313_1347	1.392	0.477	0.173197771	0.238375454	indole-3-glycerol phosphate synthase
CLO1313_RS06835	Clo1313_1348	1.514	0.598	0.098979258	0.145723512	N-(5'-phosphoribosyl)anthranilate isomerase
CLO1313_RS06840	Clo1313_1349	1.305	0.384	1.11E-06	3.48E-06	FMN reductase
CLO1313_RS06845	Clo1313_1351	1.275	0.35	1.15E-05	3.19E-05	ATP synthase
CLO1313_RS06850	Clo1313_1352	1.312	0.392	1.02E-05	2.86E-05	pilus assembly protein PilZ
CLO1313_RS06855	Clo1313_1353	1.189	0.249	0.018043236	0.032056673	2-oxoacid:acceptor oxidoreductase subunit delta
CLO1313_RS06860	Clo1313_1354	1.174	0.231	0.019979285	0.034998759	2-ketoisovalerate ferredoxin reductase
CLO1313_RS06865	Clo1313_1355	1.225	0.293	0.005657477	0.011031713	MFS transporter
CLO1313_RS06870	Clo1313_1356	1.297	0.375	0.000310241	0.000718466	2-oxoglutarate ferredoxin oxidoreductase subunit gamma
CLO1313_RS06875	Clo1313_1357	1.023	0.033	0.816936872	0.854847759	glutamine synthetase
CLO1313_RS06880	Clo1313_1358	0.958	-0.062	0.506946438	0.585644209	hypothetical protein
CLO1313_RS06885	Clo1313_1359	0.969	-0.045	0.585670329	0.661304713	transcriptional regulator

CLO1313_RS06895	Clo1313_1361	1.439	0.525	9.13E-09	3.48E-08	hypothetical protein
CLO1313_RS06900	Clo1313_1362	1.421	0.507	3.50E-09	1.39E-08	hypothetical protein
CLO1313_RS06905	Clo1313_1363	1.267	0.341	2.19E-06	6.66E-06	hypothetical protein
CLO1313_RS06910	Clo1313_1364	0.755	-0.405	1.07E-07	3.69E-07	branched-chain amino acid aminotransferase
CLO1313_RS06915	Clo1313_1365	0.502	-0.995	9.37E-29	1.22E-27	haloacid dehalogenase
CLO1313_RS06920	Clo1313_1366	0.568	-0.816	3.10E-27	3.69E-26	quinate dehydrogenase
CLO1313_RS06925	Clo1313_1367	0.551	-0.86	1.99E-17	1.45E-16	type II secretion system protein E
CLO1313_RS06930	Clo1313_1368	1.094	0.13	0.152205882	0.212210804	peptidase A24
CLO1313_RS06935	Clo1313_1369	1.233	0.303	5.80E-05	0.00014877	hypothetical protein
CLO1313_RS06940	Clo1313_1370	1.043	0.061	0.496817504	0.575494667	endonuclease IV
CLO1313_RS06950	Clo1313_1372	1.09	0.124	0.131499911	0.186727383	peptidase M24
CLO1313_RS06960	Clo1313_1374	0.702	-0.51	4.13E-15	2.68E-14	hypothetical protein
CLO1313_RS06965	Clo1313_1375	0.89	-0.168	0.129701727	0.184611049	transposase
CLO1313_RS07005	Clo1313_1383	1.048	0.068	0.785778991	0.829479477	hypothetical protein
CLO1313_RS07010	Clo1313_1384	1.173	0.23	0.020256222	0.035462369	alkaline-shock protein
CLO1313_RS07015	Clo1313_1385	1.349	0.431	1.90E-10	8.57E-10	hypothetical protein
CLO1313_RS07020	Clo1313_1386	1.355	0.438	1.90E-07	6.41E-07	hypothetical protein
CLO1313_RS07030	Clo1313_1388	1.131	0.178	0.033441441	0.056059744	exodeoxyribonuclease VII large subunit
CLO1313_RS07035	Clo1313_1389	1.251	0.323	0.007182204	0.013772013	exodeoxyribonuclease VII small subunit
CLO1313_RS07040	Clo1313_1390	1.257	0.33	0.000658983	0.001445714	farnesyl-diphosphate synthase
CLO1313_RS07045	Clo1313_1391	1.238	0.308	0.003438003	0.006938473	acid phosphatase
CLO1313_RS07050	Clo1313_1392	0.865	-0.209	0.004105571	0.008186531	Mg2+ and Co2+ transporter CorB
CLO1313_RS07060	Clo1313_1394	0.901	-0.151	0.043899383	0.071629081	RNA methyltransferase
CLO1313_RS07065	Clo1313_1395	0.924	-0.114	0.149594038	0.208666288	hypothetical protein
CLO1313_RS07070	Clo1313_1396	1.455	0.541	1.40E-06	4.34E-06	endoglucanase
CLO1313_RS07075	Clo1313_1397	0.995	-0.007	0.938702119	0.95139157	copper amine oxidase
CLO1313_RS07080	Clo1313_1398	1.315	0.396	0.037787577	0.062610466	coagulation factor 5/8 type domain protein
CLO1313_RS07085	Clo1313_1399	1.052	0.073	0.778095368	0.824852601	hypothetical protein
CLO1313_RS07090	Clo1313_1400	0.891	-0.166	0.408929771	0.494109744	hypothetical protein
CLO1313_RS07095	Clo1313_1401	1.28	0.356	0.037404307	0.06204398	ABC transporter

CLO1313_RS07100	Clo1313_1402	1.272	0.347	0.022560371	0.039154255	hypothetical protein
CLO1313_RS07105	Clo1313_1404	4.716	2.238	3.92E-66	1.93E-64	diguanylate cyclase
CLO1313_RS07110	Clo1313_1405	0.976	-0.035	0.690238933	0.750112359	NAD kinase
CLO1313_RS07115	Clo1313_1406	0.783	-0.353	1.57E-06	4.84E-06	arginine repressor
CLO1313_RS07135	Clo1313_1410	8.478	3.084	2.67E-28	3.42E-27	chemotaxis protein CheY
CLO1313_RS07140	Clo1313_1411	10.749	3.426	3.72E-88	2.86E-86	chemotaxis protein CheA
CLO1313_RS07145	Clo1313_1412	9.222	3.205	3.94E-33	5.96E-32	chemotaxis protein CheW
CLO1313_RS07150	Clo1313_1413	8.386	3.068	2.05E-51	6.33E-50	chemotaxis protein CheR
CLO1313_RS07155	Clo1313_1414	7.893	2.981	1.12E-68	5.89E-67	two-component system protein-glutamate methylesterase response regulator
CLO1313_RS07160	Clo1313_1415	5.002	2.323	2.38E-59	9.91E-58	PAS domain-containing two-component system sensor histidine kinase/response regulator
CLO1313_RS07165	Clo1313_1416	5.212	2.382	6.98E-28	8.65E-27	hisitidine kinase
CLO1313_RS07170	Clo1313_1417	1.597	0.675	7.95E-05	0.000200155	MerR family transcriptional regulator
CLO1313_RS07175	Clo1313_1418	1.094	0.129	0.189359691	0.257429607	pseudouridine synthase
CLO1313_RS07180	Clo1313_1419	1.776	0.829	7.68E-09	2.96E-08	hypothetical protein
CLO1313_RS07185	Clo1313_1421	1.614	0.691	2.94E-11	1.41E-10	transposase
CLO1313_RS07190	Clo1313_1422	1.73	0.791	7.95E-22	7.31E-21	two-component system sensor histidine kinase
CLO1313_RS07195	Clo1313_1423	1.775	0.828	8.35E-20	6.91E-19	two-component system response regulator
CLO1313_RS07200	Clo1313_1424	0.934	-0.098	0.394267674	0.480653965	lipase
CLO1313_RS07205	Clo1313_1425	1.081	0.113	0.416683428	0.501659414	glycoside hydrolase
CLO1313_RS07210		1.711	0.775	0.01617349	0.028957789	hypothetical protein
CLO1313_RS07215	Clo1313_1426	1.756	0.812	2.63E-19	2.12E-18	alpha-amylase
CLO1313_RS07220	Clo1313_1427	1.062	0.086	0.321532693	0.405840297	hypothetical protein
CLO1313_RS07225	Clo1313_1428	1.057	0.08	0.314920909	0.398501184	hypothetical protein
CLO1313_RS07230	Clo1313_1429	1.145	0.195	0.011987276	0.0219474	YggS family pyridoxal phosphate enzyme
CLO1313_RS07235	Clo1313_1430	1.093	0.129	0.062797871	0.098447891	cell division protein SepF
CLO1313_RS07240	Clo1313_1431	0.877	-0.189	0.01010658	0.018854294	hypothetical protein
CLO1313_RS07245	Clo1313_1432	0.917	-0.124	0.081789082	0.123944143	RNA-binding protein S4
CLO1313_RS07250	Clo1313_1433	0.898	-0.155	0.048066012	0.077583407	septum formation initiator

CLO1313_RS07255	Clo1313_1434	0.809	-0.306	0.00185121	0.003855401	isoleucyl-tRNA synthetase
CLO1313_RS07260	Clo1313_1435	0.813	-0.299	0.00134711	0.002859153	3-dehydroquinate synthase
CLO1313_RS07265	Clo1313_1436	1.844	0.883	1.48E-25	1.63E-24	hypothetical protein
CLO1313_RS07270	Clo1313_1437	1.056	0.079	0.357453445	0.443893533	orotate phosphoribosyltransferase
CLO1313_RS07280	Clo1313_1439	18.683	4.224	1.36E-180	6.81E-178	hypothetical protein
CLO1313_RS07285	Clo1313_1440	3.47	1.795	8.29E-30	1.10E-28	methyl-accepting chemotaxis protein
CLO1313_RS07290	Clo1313_1441	1.417	0.502	4.83E-05	0.000124439	GCN5 family N-acetyltransferase
CLO1313_RS07295	Clo1313_1442	1.336	0.418	8.19E-05	0.000205747	HAD family hydrolase
CLO1313_RS07300	Clo1313_1443	1.621	0.697	7.27E-13	3.91E-12	copper amine oxidase
CLO1313_RS07305	Clo1313_1444	1.218	0.285	0.000247396	0.000581003	hypothetical protein
CLO1313_RS07310	Clo1313_1445	0.901	-0.15	0.061777641	0.097076948	DNA mismatch repair protein MutS
CLO1313_RS07315	Clo1313_1446	0.827	-0.273	0.000736633	0.001609005	mannonate oxidoreductase
CLO1313_RS07320	Clo1313_1447	1.211	0.277	9.39E-05	0.000234533	tRNA dimethylallyltransferase
CLO1313_RS07325	Clo1313_1448	1.196	0.259	0.000461661	0.001040212	RNA chaperone Hfq
CLO1313_RS07330	Clo1313_1449	1.059	0.082	0.49282256	0.572859009	LexA repressor
CLO1313_RS07335	Clo1313_1450	1.334	0.416	0.003721393	0.007450238	peptidoglycan-binding protein LysM
CLO1313_RS07340	Clo1313_1451	0.883	-0.179	0.036257927	0.060376193	hypothetical protein
CLO1313_RS07345	Clo1313_1452	0.906	-0.142	0.06165986	0.09696797	signal recognition particle protein
CLO1313_RS07350	Clo1313_1453	0.674	-0.569	1.84E-18	1.40E-17	30S ribosomal protein S16
CLO1313_RS07355	Clo1313_1454	0.693	-0.529	1.59E-12	8.34E-12	hypothetical protein
CLO1313_RS07360	Clo1313_1455	1.213	0.279	0.003313596	0.006705449	ribosome maturation factor RimM
CLO1313_RS07365	Clo1313_1456	1.19	0.251	0.001870745	0.003893383	tRNA (guanine(37)-N(1))-methyltransferase
CLO1313_RS07385	Clo1313_1460	1.027	0.039	0.613123091	0.685591405	hypothetical protein
CLO1313_RS07390	Clo1313_1461	1.029	0.042	0.596019369	0.670223601	ribonuclease HII
CLO1313_RS07395	Clo1313_1462	1.252	0.324	0.000135589	0.000329523	hypothetical protein
CLO1313_RS07400	Clo1313_1463	1.418	0.503	1.71E-05	4.66E-05	FlhB domain protein
CLO1313_RS07405	Clo1313_1464	1.279	0.355	0.019463729	0.034195503	hypothetical protein
CLO1313_RS07410	Clo1313_1465	1.077	0.107	0.430363857	0.515645708	DNA repair protein RadC
CLO1313_RS07415	Clo1313_1466	1.147	0.198	0.18123498	0.247506241	peptidase
CLO1313_RS07420	Clo1313_1467	3.179	1.669	8.40E-46	2.19E-44	aspartate aminotransferase

CLO1313_RS07425	Clo1313_1468	1.432	0.518	0.000117935	0.000288724	hypothetical protein
CLO1313_RS07430	Clo1313_1469	1.977	0.983	1.23E-08	4.62E-08	hypothetical protein
CLO1313_RS07435	Clo1313_1470	1.544	0.626	4.52E-06	1.33E-05	hypothetical protein
CLO1313_RS07440	Clo1313_1471	0.477	-1.069	9.35E-15	5.86E-14	Cro/Cl family transcriptional regulator
CLO1313_RS07445	Clo1313_1472	0.397	-1.331	1.28E-18	9.92E-18	spermidine/putrescine import ATP-binding protein PotA
CLO1313_RS07450	Clo1313_1473	0.366	-1.451	4.55E-23	4.42E-22	ABC transporter permease
CLO1313_RS07455	Clo1313_1474	0.387	-1.37	1.48E-25	1.63E-24	spermidine/putrescine ABC transporter permease
CLO1313_RS07460	Clo1313_1475	0.352	-1.508	1.07E-35	1.86E-34	spermidine/putrescine ABC transporter substrate-binding protein
CLO1313_RS07465	Clo1313_1476	0.476	-1.071	2.50E-17	1.80E-16	signal peptidase
CLO1313_RS07470	Clo1313_1477	1.825	0.868	1.17E-18	9.11E-18	endoglucanase
CLO1313_RS07475	Clo1313_1478	0.615	-0.702	3.45E-14	2.09E-13	copper amine oxidase
CLO1313_RS07480	Clo1313_1479	0.896	-0.159	0.044913851	0.072927255	MATE family efflux transporter
CLO1313_RS07485	Clo1313_1480	0.885	-0.176	0.064181179	0.100145346	hypothetical protein
CLO1313_RS07490	Clo1313_1481	0.721	-0.471	9.83E-15	6.14E-14	adenylosuccinate lyase
CLO1313_RS07495	Clo1313_1482	0.846	-0.242	0.001466669	0.003101933	GntR family transcriptional regulator
CLO1313_RS07500	Clo1313_1483	0.811	-0.302	1.41E-05	3.89E-05	hypothetical protein
CLO1313_RS07505	Clo1313_1484	1.062	0.087	0.611280658	0.684552163	copper chaperone CopZ
CLO1313_RS07510	Clo1313_1486	0.456	-1.132	8.87E-25	9.26E-24	phosphopantetheine-protein transferase
CLO1313_RS07515	Clo1313_1487	0.466	-1.103	2.49E-25	2.72E-24	cellulosome anchoring protein cohesin region
CLO1313_RS07520	Clo1313_1488	0.734	-0.445	1.03E-08	3.90E-08	cellulosome anchoring protein cohesin region
CLO1313_RS07525	Clo1313_1489	0.716	-0.483	9.22E-08	3.20E-07	peptidase M23
CLO1313_RS07530	Clo1313_1490	1.663	0.734	4.16E-11	1.98E-10	pilus assembly protein PilZ
CLO1313_RS07535	Clo1313_1491	1.033	0.047	0.648560247	0.716666242	chorismate synthase
CLO1313_RS07540	Clo1313_1492	0.921	-0.119	0.088959717	0.132599499	shikimate kinase
CLO1313_RS07545	Clo1313_1493	1.009	0.013	0.877966856	0.903886921	cell division protein FtsZ
CLO1313_RS07550	Clo1313_1494	0.795	-0.33	0.008656855	0.016369425	LuxR family transcriptional regulator
CLO1313_RS07555	Clo1313_1495	1.186	0.246	0.008692939	0.0164273	tyrosine recombinase XerC
CLO1313_RS07560	Clo1313_1496	0.699	-0.516	8.65E-06	2.45E-05	transcriptional regulator
CLO1313_RS07565	Clo1313_1497	1.144	0.194	0.013689986	0.02477747	aminopeptidase

CLO1313_RS07570	Clo1313_1498	2.115	1.081	1.76E-52	5.66E-51	phosphodiesterase
CLO1313_RS07575	Clo1313_1499	0.662	-0.595	1.21E-12	6.42E-12	histidinol phosphate phosphatase
CLO1313_RS07580	Clo1313_1500	0.794	-0.333	0.003451501	0.00696103	tyrosine--tRNA ligase
CLO1313_RS07585	Clo1313_1501	1.243	0.314	0.003489451	0.007023398	tRNA-specific 2-thiouridylase MnmA
CLO1313_RS07600	Clo1313_1504	2.061	1.044	5.66E-21	4.93E-20	AsnC family transcriptional regulator
CLO1313_RS07605	Clo1313_1505	0.66	-0.599	0.00150949	0.003181278	hypothetical protein
CLO1313_RS07610	Clo1313_1507	0.888	-0.171	0.02995198	0.050749146	chemotaxis protein CheR
CLO1313_RS07615	Clo1313_1508	0.907	-0.141	0.086658142	0.13015812	nucleoside diphosphate kinase
CLO1313_RS07620	Clo1313_1509	0.392	-1.349	1.44E-43	3.35E-42	S-adenosylmethionine decarboxylase proenzyme
CLO1313_RS07630	Clo1313_1511	0.788	-0.343	2.48E-06	7.50E-06	1-acyl-sn-glycerol-3-phosphate acyltransferase
CLO1313_RS07635	Clo1313_1512	0.926	-0.111	0.111628982	0.161961933	cytidylate kinase
CLO1313_RS07645	Clo1313_1514	0.915	-0.127	0.088027362	0.131471144	hypothetical protein
CLO1313_RS07650	Clo1313_1515	1.221	0.289	0.005661329	0.01103205	hypothetical protein
CLO1313_RS07655	Clo1313_1516	1.14	0.189	0.027844083	0.047382087	FAD-dependent oxidoreductase
CLO1313_RS07660	Clo1313_1517	1.134	0.181	0.011604683	0.021325028	phosphoglycerate mutase
CLO1313_RS07665	Clo1313_1518	1.165	0.22	0.002736335	0.005597727	N-acetylmannosamine kinase
CLO1313_RS07670	Clo1313_1520	0.524	-0.931	8.14E-16	5.47E-15	hypothetical protein
CLO1313_RS07675	Clo1313_1521	0.789	-0.342	0.000183341	0.000438818	hypothetical protein
CLO1313_RS07680	Clo1313_1522	0.644	-0.634	1.93E-06	5.89E-06	hypothetical protein
CLO1313_RS07685	Clo1313_1523	0.791	-0.339	0.002081236	0.004310516	oxaloacetate decarboxylase
CLO1313_RS07690	Clo1313_1524	0.656	-0.608	3.74E-05	9.72E-05	carboxylesterase
CLO1313_RS07695	Clo1313_1525	0.658	-0.603	5.62E-05	0.000144197	sodium pump decarboxylase subunit gamma
CLO1313_RS07700	Clo1313_1526	0.652	-0.617	4.66E-06	1.37E-05	methylmalonyl-CoA carboxyltransferase
CLO1313_RS07705	Clo1313_1527	0.84	-0.252	0.010109271	0.018854294	pilus assembly protein PilZ
CLO1313_RS07720	Clo1313_1530	0.473	-1.081	3.20E-28	4.07E-27	polyamine aminopropyltransferase
CLO1313_RS07725	Clo1313_1531	0.469	-1.092	4.82E-19	3.84E-18	ribosomal large subunit pseudouridine synthase B
CLO1313_RS07735	Clo1313_1533	1.188	0.249	0.334476595	0.419354226	hypothetical protein
CLO1313_RS07740	Clo1313_1534	0.975	-0.037	0.657335149	0.724021056	hypothetical protein
CLO1313_RS07750	Clo1313_1536	0.855	-0.225	0.010236723	0.019056446	condensin subunit ScpA
CLO1313_RS07760	Clo1313_1538	0.985	-0.021	0.800180608	0.841084722	peptidase M50

CLO1313_RS07765	Clo1313_1539	1.098	0.135	0.087909193	0.131471144	polynucleotide adenylyltransferase
CLO1313_RS07775	Clo1313_1541	0.942	-0.086	0.735648061	0.789058846	hypothetical protein
CLO1313_RS07780	Clo1313_1542	1.061	0.086	0.455184562	0.539138429	inosine-5-monophosphate dehydrogenase
CLO1313_RS07785	Clo1313_1543	0.968	-0.047	0.692119709	0.751508692	lysine transporter LysE
CLO1313_RS07790	Clo1313_1544	1.103	0.142	0.09763047	0.143949743	D-alanyl-D-alanine carboxypeptidase
CLO1313_RS07800	Clo1313_1546	1.045	0.063	0.487232881	0.567681201	phosphopentomutase
CLO1313_RS07805	Clo1313_1547	0.534	-0.906	5.73E-14	3.39E-13	tyrosine recombinase XerD
CLO1313_RS07815	Clo1313_1549	1.232	0.301	1.16E-05	3.23E-05	ADP-ribose pyrophosphatase
CLO1313_RS07820	Clo1313_1550	1.083	0.115	0.153074813	0.213224043	ribonuclease H
CLO1313_RS07825	Clo1313_1551	1.217	0.283	9.64E-05	0.000240144	pyrroline-5-carboxylate reductase
CLO1313_RS07830	Clo1313_R0041	0.966	-0.05	0.767674451	0.815434542	
CLO1313_RS07835	Clo1313_1552	1.089	0.124	0.797686932	0.839980025	hypothetical protein
CLO1313_RS07840	Clo1313_1553	1.232	0.301	0.393462579	0.479867537	germination protein
CLO1313_RS07845	Clo1313_1555	1.046	0.065	0.869398037	0.896713521	germination protein
CLO1313_RS07850	Clo1313_1556	1.581	0.661	0.097361239	0.143658131	germination protein
CLO1313_RS07855	Clo1313_1557	1.315	0.395	0.100448718	0.147597111	germination protein GerA
CLO1313_RS07860	Clo1313_1558	1.096	0.132	0.141461936	0.198380005	membrane protein
CLO1313_RS07870	Clo1313_1560	1.707	0.771	0.072316424	0.111447562	hypothetical protein
CLO1313_RS07875	Clo1313_R0042	0.254	-1.98	1.13E-25	1.25E-24	
CLO1313_RS07880	Clo1313_1561	0.554	-0.851	1.60E-07	5.44E-07	hypothetical protein
CLO1313_RS07885		1.224	0.292	0.128920051	0.183847472	hypothetical protein
CLO1313_RS07890	Clo1313_1563	1.363	0.446	3.64E-08	1.31E-07	hypothetical protein
CLO1313_RS07895	Clo1313_1564	1.177	0.236	0.006159204	0.011917067	glycoside hydrolase
CLO1313_RS07900	Clo1313_1565	1.693	0.76	0.025078541	0.043174825	hypothetical protein
CLO1313_RS07905	Clo1313_1566	1.525	0.609	0.005453143	0.010660959	hypothetical protein
CLO1313_RS07910	Clo1313_1567	0.873	-0.196	0.021868065	0.03801874	hypothetical protein
CLO1313_RS07915	Clo1313_1569	1.175	0.233	0.002580191	0.005299995	pilus assembly protein PilM
CLO1313_RS07920	Clo1313_1570	0.903	-0.147	0.112431114	0.162889329	cysteine desulfurase
CLO1313_RS07925	Clo1313_1571	0.762	-0.393	8.08E-05	0.000203131	[FeFe] hydrogenase H-cluster radical SAM maturase HydG
CLO1313_RS07930	Clo1313_1572	1.718	0.781	2.89E-10	1.28E-09	CopG family transcriptional regulator

CLO1313_RS07935	Clo1313_1573	0.989	-0.016	0.910152095	0.929998682	4-oxalocrotonate tautomerase
CLO1313_RS07940	Clo1313_1574	1.094	0.13	0.363615144	0.450240222	hypothetical protein
CLO1313_RS07945	Clo1313_1575	1.085	0.118	0.753340926	0.802297385	hypothetical protein
CLO1313_RS07950	Clo1313_1576	0.689	-0.537	0.223232734	0.296353006	hypothetical protein
CLO1313_RS07955	Clo1313_1577	0.939	-0.091	0.758886473	0.807056927	hypothetical protein
CLO1313_RS07960	Clo1313_R0043	0.582	-0.782	6.31E-05	0.000160279	
CLO1313_RS07965	Clo1313_1578	0.635	-0.655	3.35E-08	1.21E-07	glutamate--tRNA ligase
CLO1313_RS07970	Clo1313_1579	0.754	-0.407	0.000338826	0.00077865	ribonucleoside-triphosphate reductase activating protein
CLO1313_RS07975	Clo1313_1580	0.741	-0.433	2.60E-07	8.69E-07	ribonucleoside triphosphate reductase
CLO1313_RS07980	Clo1313_1581	0.873	-0.195	0.016308007	0.029181213	5-methyltetrahydrofolate--homocysteine methyltransferase
CLO1313_RS07985	Clo1313_1582	0.88	-0.185	0.018268784	0.032399813	methionine synthase
CLO1313_RS07990	Clo1313_1583	1.138	0.187	0.423937536	0.508555469	membrane protein
CLO1313_RS07995	Clo1313_1584	1.635	0.709	3.32E-06	9.88E-06	hypothetical protein
CLO1313_RS08000	Clo1313_1585	1.658	0.729	4.43E-09	1.74E-08	coat protein F
CLO1313_RS08005	Clo1313_1586	1.273	0.348	0.24184397	0.318388966	hypothetical protein
CLO1313_RS08010	Clo1313_1587	1.484	0.569	1.34E-12	7.09E-12	dockerin
CLO1313_RS08015	Clo1313_1588	0.87	-0.201	0.009867098	0.018437025	glycoside hydrolase
CLO1313_RS08025	Clo1313_1590	0.823	-0.281	0.00048511	0.001085705	hypothetical protein
CLO1313_RS08030	Clo1313_1591	0.524	-0.931	5.16E-13	2.84E-12	hypothetical protein
CLO1313_RS08035	Clo1313_1592	0.522	-0.937	4.17E-12	2.14E-11	serine/threonine phosphatase
CLO1313_RS08040	Clo1313_1593	0.92	-0.121	0.057175576	0.090772658	serine/threonine protein kinase
CLO1313_RS08045	Clo1313_1594	1.027	0.039	0.642119921	0.710859226	hypothetical protein
CLO1313_RS08050	Clo1313_1595	1.013	0.019	0.820773553	0.857147737	hypothetical protein
CLO1313_RS08055	Clo1313_1596	0.95	-0.074	0.412899738	0.498682557	hypothetical protein
CLO1313_RS08060	Clo1313_1597	0.779	-0.36	0.000481874	0.001080882	hypothetical protein
CLO1313_RS08065	Clo1313_1598	0.773	-0.372	6.43E-05	0.000162669	hypothetical protein
CLO1313_RS08070	Clo1313_1599	0.867	-0.206	0.025339327	0.043598762	type II secretion system protein E
CLO1313_RS08075	Clo1313_1600	0.863	-0.213	0.017379344	0.030932138	ATPase
CLO1313_RS08080	Clo1313_1601	0.781	-0.356	0.000360616	0.000826193	flagellar protein FlgA
CLO1313_RS08085	Clo1313_1602	1.128	0.174	0.080453568	0.122291055	hypothetical protein

CLO1313_RS08090	Clo1313_1603	1.313	0.392	0.00120294	0.002571359	endoglucanase
CLO1313_RS08095	Clo1313_1604	0.884	-0.177	0.190493937	0.258269131	glycoside hydrolase
CLO1313_RS08100	Clo1313_1605	1.215	0.28	0.122192352	0.175253402	conjugal transfer protein TraR
CLO1313_RS08105	Clo1313_1606	0.886	-0.175	0.04099768	0.067481911	5'-methylthioadenosine phosphorylase
CLO1313_RS08115	Clo1313_1608	0.935	-0.098	0.200493775	0.269511802	DtxR family transcriptional regulator
CLO1313_RS08120	Clo1313_1609	2.077	1.055	8.44E-20	6.97E-19	iron transporter FeoA
CLO1313_RS08130	Clo1313_1612	1.095	0.131	0.069530178	0.107707129	ATPase AAA
CLO1313_RS08135	Clo1313_1613	0.873	-0.196	0.08209966	0.124309776	amino acid-binding protein
CLO1313_RS08140	Clo1313_1614	0.882	-0.181	0.103894229	0.152063832	phenylacetate-CoA ligase
CLO1313_RS08155	Clo1313_1617	1.369	0.453	1.48E-10	6.75E-10	sodium:solute symporter
CLO1313_RS08160	Clo1313_1618	0.95	-0.074	0.321011626	0.405353207	MBL fold hydrolase
CLO1313_RS08165	Clo1313_1619	0.707	-0.501	1.01E-09	4.25E-09	histidinol-phosphate aminotransferase
CLO1313_RS08170	Clo1313_1620	1.074	0.103	0.415573242	0.501127524	aminopeptidase
CLO1313_RS08175	Clo1313_1621	1.107	0.146	0.181697241	0.247998341	aminopeptidase
CLO1313_RS08180	Clo1313_1622	0.971	-0.043	0.684197383	0.745606087	peptidase M42
CLO1313_RS08185	Clo1313_1623	0.761	-0.395	1.55E-05	4.23E-05	competence protein ComEC
CLO1313_RS08190	Clo1313_1624	0.935	-0.098	0.266737268	0.347802202	peptidase
CLO1313_RS08195	Clo1313_1625	1.241	0.311	0.041076904	0.067575225	hypothetical protein
CLO1313_RS08200	Clo1313_1626	1.443	0.529	0.000249597	0.000585712	GCN5 family N-acetyltransferase
CLO1313_RS08205	Clo1313_1627	1.789	0.839	3.57E-06	1.06E-05	phosphomethylpyrimidine synthase
CLO1313_RS08210	Clo1313_1628	1.932	0.95	1.24E-06	3.87E-06	thiamine-phosphate pyrophosphorylase
CLO1313_RS08220	Clo1313_1630	2.272	1.184	5.37E-11	2.53E-10	thiamine biosynthesis protein ThiH
CLO1313_RS08225	Clo1313_1631	2.197	1.135	2.70E-10	1.20E-09	thiazole synthase
CLO1313_RS08235	Clo1313_1633	1.115	0.157	0.112785517	0.163323885	GTP-binding protein
CLO1313_RS08240	Clo1313_1634	1.135	0.183	0.561019886	0.637665135	hypothetical protein
CLO1313_RS08245	Clo1313_1636	1.301	0.38	0.000487515	0.001090275	hypothetical protein
CLO1313_RS08250	Clo1313_1637	0.988	-0.018	0.8028624	0.843061743	copper amine oxidase
CLO1313_RS08255	Clo1313_1638	1.491	0.577	0.285631388	0.368275379	type 11 methyltransferase
CLO1313_RS08285	Clo1313_1642	1.587	0.666	8.72E-05	0.000218474	hypothetical protein
CLO1313_RS08290	Clo1313_1643	1.51	0.594	7.98E-10	3.36E-09	resolvase

CLO1313_RS08295	Clo1313_1644	1.686	0.754	1.45E-08	5.45E-08	hypothetical protein
CLO1313_RS08300	Clo1313_1645	1.467	0.553	8.62E-06	2.44E-05	DEAD/DEAH box helicase
CLO1313_RS08315	Clo1313_1647	1.536	0.619	7.83E-07	2.50E-06	membrane protein
CLO1313_RS08320	Clo1313_1648	1.678	0.747	2.85E-13	1.59E-12	resolvase
CLO1313_RS08325	Clo1313_1649	1.221	0.288	0.706010754	0.763275505	hypothetical protein
CLO1313_RS08335		1.248	0.319	0.000122878	0.000300089	integrase
CLO1313_RS08340	Clo1313_1652	1.052	0.073	0.529382961	0.608628188	hypothetical protein
CLO1313_RS08345	Clo1313_R0044	1.076	0.106	0.628190955	0.698792535	
CLO1313_RS08350	Clo1313_1653	1.416	0.502	1.10E-07	3.78E-07	hypothetical protein
CLO1313_RS08355	Clo1313_1654	1.03	0.042	0.578504815	0.653952484	chemotaxis protein CheY
CLO1313_RS08360	Clo1313_1655	0.897	-0.157	0.065327584	0.101828184	sensor histidine kinase
CLO1313_RS08365	Clo1313_1656	0.959	-0.06	0.386916865	0.472845835	hypothetical protein
CLO1313_RS08370	Clo1313_1657	0.855	-0.226	0.000546079	0.001213105	aspartate aminotransferase
CLO1313_RS08375	Clo1313_1658	1.162	0.217	0.010222464	0.019041719	nitroreductase
CLO1313_RS08380	Clo1313_1659	0.997	-0.004	0.96814521	0.974972292	endoglucanase
CLO1313_RS08385	Clo1313_1660	0.861	-0.217	0.00746548	0.014296918	thiamine pyrophosphokinase
CLO1313_RS08390	Clo1313_1661	0.885	-0.176	0.023264223	0.040212913	ribulose-phosphate 3-epimerase
CLO1313_RS08400	Clo1313_1663	0.93	-0.104	0.139927409	0.196369817	serine/threonine protein kinase
CLO1313_RS08405	Clo1313_1664	1.02	0.028	0.680297322	0.743517372	protein phosphatase
CLO1313_RS08410	Clo1313_1665	1.041	0.058	0.406212064	0.491618232	23S rRNA (adenine(2503)-C(2))-methyltransferase RlmN
CLO1313_RS08415	Clo1313_1666	1.153	0.206	0.014617945	0.02629827	16S rRNA methyltransferase
CLO1313_RS08420	Clo1313_1667	1.198	0.26	0.003676642	0.007365564	zinc metallopeptidase
CLO1313_RS08425	Clo1313_1668	1.021	0.03	0.75042773	0.800616422	hypothetical protein
CLO1313_RS08430	Clo1313_1669	1.026	0.036	0.69988898	0.758909797	methionyl-tRNA formyltransferase
CLO1313_RS08440	Clo1313_1671	1.216	0.282	0.000460936	0.001039785	primosomal protein N'
CLO1313_RS08445	Clo1313_1672	2.009	1.006	3.36E-21	2.98E-20	hypothetical protein
CLO1313_RS08450	Clo1313_1673	0.665	-0.588	3.62E-16	2.46E-15	trans-hexaprenyltranstransferase
CLO1313_RS08455	Clo1313_1674	0.633	-0.661	1.12E-10	5.17E-10	heptaprenyl diphosphate synthase subunit I
CLO1313_RS08460	Clo1313_1675	0.647	-0.627	3.48E-10	1.52E-09	hypothetical protein
CLO1313_RS08465	Clo1313_1676	0.632	-0.663	5.52E-13	3.02E-12	thiamine biosynthesis lipoprotein ApbE

CLO1313_RS08470	Clo1313_1677	1.031	0.044	0.703926638	0.76184626	pyridine nucleotide-disulfide oxidoreductase
CLO1313_RS08475	Clo1313_1678	1.075	0.104	0.458099843	0.541279204	single-stranded DNA-binding protein
CLO1313_RS08480	Clo1313_1679	4.787	2.259	3.65E-17	2.62E-16	glutamate ligase
CLO1313_RS08485	Clo1313_1680	10.6	3.406	3.40E-17	2.44E-16	polysaccharide deacetylase
CLO1313_RS08495	Clo1313_1682	1.94	0.956	6.81E-18	5.10E-17	peptidyl-prolyl cis-trans isomerase
CLO1313_RS08500	Clo1313_1683	3.397	1.764	1.32E-47	3.65E-46	phosphoribosylformylglycinamidine synthase
CLO1313_RS08505	Clo1313_1684	1.806	0.853	5.86E-17	4.14E-16	LysR family transcriptional regulator
CLO1313_RS08510	Clo1313_1685	1.175	0.233	0.002222964	0.004581903	transcriptional regulator
CLO1313_RS08515	Clo1313_1686	1.01	0.015	0.821421339	0.857147737	acetyl-CoA synthetase
CLO1313_RS08520		1.281	0.358	0.26890781	0.350023664	hypothetical protein
CLO1313_RS08525	Clo1313_1687	1.199	0.262	0.001276238	0.002718352	heat-shock protein Hsp90
CLO1313_RS08530	Clo1313_1688	1.924	0.944	1.18E-13	6.80E-13	metal ABC transporter permease
CLO1313_RS08535	Clo1313_1689	1.71	0.774	1.31E-07	4.50E-07	zinc ABC transporter ATP-binding protein
CLO1313_RS08540	Clo1313_1690	1.67	0.74	7.66E-08	2.67E-07	ABC transporter substrate-binding protein
CLO1313_RS08545	Clo1313_1691	1.473	0.559	5.09E-11	2.40E-10	Fur family transcriptional regulator
CLO1313_RS08550	Clo1313_1692	1.131	0.177	0.088513173	0.132065176	peptidase A24
CLO1313_RS08555	Clo1313_1693	1.136	0.184	0.023595633	0.040738804	serine/threonine phosphatase
CLO1313_RS08560	Clo1313_1694	0.957	-0.063	0.509765758	0.588221435	endoglucanase
CLO1313_RS08565	Clo1313_1695	0.831	-0.267	0.001835042	0.003829709	hypothetical protein
CLO1313_RS08570	Clo1313_1696	0.881	-0.182	0.03211351	0.054014816	peptidase M24
CLO1313_RS08575	Clo1313_1697	1.307	0.386	6.40E-05	0.000162026	hypothetical protein
CLO1313_RS08580	Clo1313_1698	1.341	0.423	0.000138519	0.000335827	peptide ABC transporter ATP-binding protein
CLO1313_RS08585	Clo1313_1699	1.46	0.546	5.79E-06	1.68E-05	two-component system sensor histidine kinase
CLO1313_RS08590	Clo1313_1700	1.264	0.338	0.00018241	0.000437638	two-component system response regulator
CLO1313_RS08595	Clo1313_1701	0.858	-0.22	0.000390745	0.000890711	endoglucanase
CLO1313_RS08600	Clo1313_1702	1.512	0.596	2.41E-10	1.08E-09	hypothetical protein
CLO1313_RS08605	Clo1313_1703	2.058	1.041	5.85E-11	2.75E-10	bacteriocin ABC transporter ATP-binding protein
CLO1313_RS08610	Clo1313_1704	1.645	0.718	7.05E-06	2.02E-05	radical SAM/SPASM domain-containing protein
CLO1313_RS08615	Clo1313_1705	0.968	-0.047	0.88071127	0.905779526	haloacid dehalogenase
CLO1313_RS08620	Clo1313_1706	1.108	0.148	0.438696301	0.523121354	hypothetical protein

CLO1313_RS08625	Clo1313_1707	1.065	0.091	0.454459309	0.538492086	hypothetical protein
CLO1313_RS08630		1.553	0.635	0.001127484	0.002418688	peptidoglycan-binding protein
CLO1313_RS08635	Clo1313_1709	0.696	-0.522	0.002298204	0.004733733	hypothetical protein
CLO1313_RS08640	Clo1313_1710	0.903	-0.148	0.152858533	0.213021719	transposase
CLO1313_RS08645	Clo1313_R0045	0.307	-1.704	1.29E-18	1.00E-17	
CLO1313_RS08650	Clo1313_R0046	0.079	-3.656	0.012967862	0.023613005	
CLO1313_RS08655	Clo1313_1711	0.22	-2.185	3.53E-28	4.45E-27	two-component system sensor histidine kinase
CLO1313_RS08660	Clo1313_1712	0.682	-0.553	1.10E-10	5.08E-10	glutaconyl-CoA decarboxylase subunit beta
CLO1313_RS08665	Clo1313_1713	0.653	-0.615	6.31E-10	2.69E-09	oxaloacetate decarboxylase
CLO1313_RS08670	Clo1313_1714	0.934	-0.099	0.674377728	0.738420545	hypothetical protein
CLO1313_RS08675	Clo1313_1715	1.157	0.211	0.014322024	0.025827872	DNA polymerase III
CLO1313_RS08685	Clo1313_1717	1.182	0.241	0.04369972	0.071419869	formate acetyltransferase
CLO1313_RS08690	Clo1313_1718	1.367	0.451	1.91E-08	7.05E-08	poly-gamma-glutamate biosynthesis protein
CLO1313_RS08695	Clo1313_1719	1.321	0.401	2.29E-06	6.94E-06	2'-5' RNA ligase
CLO1313_RS08700	Clo1313_1720	1.21	0.275	0.345521956	0.431040077	hypothetical protein
CLO1313_RS08705	Clo1313_1721	3.015	1.592	6.50E-59	2.60E-57	aminodeoxychorismate lyase
CLO1313_RS08710	Clo1313_1722	3.141	1.651	1.14E-48	3.34E-47	hypothetical protein
CLO1313_RS08715	Clo1313_1723	2.567	1.36	1.02E-18	7.94E-18	hypothetical protein
CLO1313_RS08720	Clo1313_1724	2.296	1.199	3.02E-40	6.46E-39	hypothetical protein
CLO1313_RS08725	Clo1313_1725	2.21	1.144	1.53E-28	1.97E-27	DNA-directed RNA polymerase sigma-70 factor
CLO1313_RS08730	Clo1313_1726	2.042	1.03	1.38E-09	5.74E-09	hypothetical protein
CLO1313_RS08735	Clo1313_1727	2.622	1.39	9.35E-32	1.31E-30	chemotaxis protein CheD
CLO1313_RS08740	Clo1313_1728	2.503	1.324	3.68E-33	5.60E-32	chemotaxis protein CheC
CLO1313_RS08745	Clo1313_1729	2.475	1.308	6.65E-36	1.17E-34	chemotaxis protein CheW
CLO1313_RS08750	Clo1313_1730	2.498	1.321	3.70E-61	1.61E-59	chemotaxis protein CheA
CLO1313_RS08755	Clo1313_1731	2.408	1.268	6.82E-39	1.39E-37	two-component system protein-glutamate methylesterase response regulator
CLO1313_RS08760	Clo1313_1732	2.63	1.395	2.09E-20	1.77E-19	pilus assembly protein PilZ
CLO1313_RS08765	Clo1313_1733	2.73	1.449	8.54E-47	2.33E-45	ATPase
CLO1313_RS08785	Clo1313_1737	1.87	0.903	1.96E-14	1.20E-13	flagellar biosynthesis protein FliR

CLO1313_RS08800	Clo1313_1740	2.024	1.017	4.16E-20	3.51E-19	hypothetical protein
CLO1313_RS08805	Clo1313_1741	2.017	1.012	8.03E-15	5.07E-14	chemotaxis protein CheY
CLO1313_RS08810	Clo1313_1742	2.378	1.25	7.82E-27	9.12E-26	flagellar motor switch protein FliN
CLO1313_RS08815	Clo1313_1743	2.411	1.269	2.72E-32	3.91E-31	flagellar motor switch protein FliM
CLO1313_RS08820	Clo1313_1744	2.057	1.04	1.82E-25	2.00E-24	flagellar basal body-associated protein FliL
CLO1313_RS08825	Clo1313_1745	1.842	0.882	2.45E-23	2.40E-22	flagellar protein
CLO1313_RS08830	Clo1313_1746	2.497	1.32	5.89E-60	2.52E-58	flagellar hook protein FlgE
CLO1313_RS08835	Clo1313_1747	2.703	1.435	7.55E-55	2.66E-53	flagellar biosynthesis protein
CLO1313_RS08840	Clo1313_1748	2.971	1.571	1.19E-71	6.88E-70	flagellar basal body rod modification protein FlgD
CLO1313_RS08845	Clo1313_1749	2.935	1.553	8.80E-86	6.59E-84	flagellar hook-length control protein
CLO1313_RS08850	Clo1313_1750	2.353	1.235	1.07E-20	9.19E-20	hypothetical protein
CLO1313_RS08860	Clo1313_1752	1.133	0.18	0.139533908	0.196001025	ATP synthase
CLO1313_RS08865	Clo1313_1753	0.974	-0.039	0.689573389	0.749829802	flagellar biosynthesis protein
CLO1313_RS08870	Clo1313_1754	0.931	-0.103	0.322652178	0.406739757	flagellar motor switch protein FliG
CLO1313_RS08875	Clo1313_1755	1.037	0.053	0.630757029	0.700736928	flagellar M-ring protein FliF
CLO1313_RS08880	Clo1313_1756	1.168	0.225	0.019565178	0.034353612	flagellar hook-basal body protein FliE
CLO1313_RS08895	Clo1313_1759	0.731	-0.453	1.26E-08	4.73E-08	methylenetetrahydrofolate--tRNA-(uracil-5-)-methyltransferase TrmFO
CLO1313_RS08900	Clo1313_1760	0.663	-0.593	2.33E-12	1.21E-11	DNA topoisomerase I
CLO1313_RS08915	Clo1313_1763	0.663	-0.593	2.35E-09	9.50E-09	membrane protein
CLO1313_RS08920	Clo1313_1764	0.631	-0.663	3.65E-10	1.59E-09	hypothetical protein
CLO1313_RS08925	Clo1313_1765	0.623	-0.682	6.87E-13	3.71E-12	Fis family transcriptional regulator
CLO1313_RS08930	Clo1313_1766	1.884	0.914	0.002067783	0.004285613	hypothetical protein
CLO1313_RS08935	Clo1313_1767	1.968	0.976	0.000183938	0.000439897	hypothetical protein
CLO1313_RS08940	Clo1313_1768	1.208	0.273	0.005957968	0.011565014	cellulosome anchoring protein cohesin region
CLO1313_RS08945	Clo1313_1769	0.921	-0.119	0.1180914	0.170513293	hypothetical protein
CLO1313_RS08950	Clo1313_1770	0.959	-0.06	0.49539347	0.574733082	NrdR family transcriptional regulator
CLO1313_RS08955	Clo1313_1771	4.328	2.114	0.242783221	0.319485247	sporulation protein, YlmC/YmxH family
CLO1313_RS08965	Clo1313_1773	1.163	0.218	0.19120519	0.258882332	DNA-directed RNA polymerase sigma-70 factor
CLO1313_RS08975	Clo1313_1775	0.858	-0.221	0.002985007	0.006065065	cell division protein FtsZ

CLO1313_RS08985	Clo1313_1777	1.032	0.045	0.564376769	0.640638126	hypothetical protein
CLO1313_RS08990	Clo1313_1778	0.985	-0.022	0.766641901	0.814726811	cell division protein FtsQ
CLO1313_RS09000	Clo1313_1780	0.98	-0.03	0.712379672	0.767946311	hypothetical protein
CLO1313_RS09005	Clo1313_1781	0.884	-0.177	0.011933092	0.021861541	glutamine amidotransferase
CLO1313_RS09010		0.789	-0.342	0.096648688	0.142782963	hypothetical protein
CLO1313_RS09015	Clo1313_1782	1.572	0.653	0.000748382	0.001633478	transposase
CLO1313_RS09020	Clo1313_1783	1.115	0.157	0.666796857	0.731427862	LuxR family transcriptional regulator
CLO1313_RS09025	Clo1313_1784	1.106	0.145	0.189002326	0.257060306	hypothetical protein
CLO1313_RS09030	Clo1313_1785	0.932	-0.102	0.13642641	0.192800867	hypothetical protein
CLO1313_RS09035	Clo1313_1786	1.154	0.206	0.006557458	0.012646828	dockerin
CLO1313_RS09040	Clo1313_1787	1.032	0.046	0.881628221	0.905894147	ATPase
CLO1313_RS09045	Clo1313_1788	0.841	-0.25	0.007556925	0.014444371	glycoside hydrolase
CLO1313_RS09050	Clo1313_1789	0.411	-1.283	2.78E-25	3.02E-24	hypothetical protein
CLO1313_RS09055	Clo1313_1790	0.536	-0.899	2.87E-22	2.69E-21	hypothetical protein
CLO1313_RS09060	Clo1313_1791	0.551	-0.86	1.56E-19	1.27E-18	ferredoxin
CLO1313_RS09065	Clo1313_1792	0.613	-0.705	2.06E-13	1.17E-12	NADH dehydrogenase
CLO1313_RS09070	Clo1313_1793	0.69	-0.536	8.99E-08	3.12E-07	NADH dehydrogenase
CLO1313_RS09075	Clo1313_1794	0.864	-0.211	0.020510527	0.035845613	serine/threonine phosphatase
CLO1313_RS09080	Clo1313_1795	0.816	-0.293	0.003507063	0.007054113	signal transduction histidine kinase
CLO1313_RS09085	Clo1313_1796	0.914	-0.13	0.183204133	0.249740544	hypothetical protein
CLO1313_RS09090	Clo1313_1797	0.886	-0.175	0.087672255	0.131267644	aminoglycoside phosphotransferase
CLO1313_RS09095	Clo1313_1798	0.8	-0.322	0.001121297	0.002407137	acetaldehyde dehydrogenase
CLO1313_RS09100	Clo1313_1799	0.785	-0.35	3.44E-05	8.99E-05	REX family transcriptional regulator
CLO1313_RS09105	Clo1313_1800	0.623	-0.684	0.000107337	0.000264506	dipicolinate synthase
CLO1313_RS09110	Clo1313_1801	0.678	-0.561	0.0027746	0.005656713	dihydrofolate reductase
CLO1313_RS09115	Clo1313_1802	0.751	-0.413	4.38E-11	2.08E-10	zinc protease
CLO1313_RS09120	Clo1313_1803	0.704	-0.507	8.38E-14	4.91E-13	polyribonucleotide nucleotidyltransferase
CLO1313_RS09125	Clo1313_1804	0.831	-0.267	0.000124803	0.000304297	30S ribosomal protein S15
CLO1313_RS09130	Clo1313_1805	4.68	2.226	0.403060772	0.48859307	hypothetical protein
CLO1313_RS09135	Clo1313_1806	1.048	0.067	0.944387243	0.955476087	spore coat protein

CLO1313_RS09140	Clo1313_1807	1.031	0.044	0.902833711	0.923498467	hypothetical protein
CLO1313_RS09145	Clo1313_1808	2.368	1.244	3.62E-31	5.01E-30	cellulose 1,4-beta-cellobiosidase
CLO1313_RS09150	Clo1313_1809	1.012	0.017	0.899696397	0.920883787	cellulose 1,4-beta-cellobiosidase
CLO1313_RS09155	Clo1313_1810	1.154	0.206	0.007798538	0.014858842	hypothetical protein
CLO1313_RS09160	Clo1313_1811	1.357	0.44	7.95E-06	2.26E-05	hypothetical protein
CLO1313_RS09165	Clo1313_1812	1.148	0.199	0.066927819	0.10421419	hypothetical protein
CLO1313_RS09170	Clo1313_1813	0.613	-0.707	1.21E-19	9.89E-19	diguanylate cyclase
CLO1313_RS09175	Clo1313_1814	1.319	0.399	0.000123607	0.000301626	biotin synthase
CLO1313_RS09180	Clo1313_1815	1.813	0.858	2.19E-18	1.67E-17	DNA metabolism protein
CLO1313_RS09185	Clo1313_1816	1.765	0.819	1.91E-27	2.30E-26	endoglucanase
CLO1313_RS09190	Clo1313_1817	1.342	0.424	5.28E-06	1.54E-05	type 3a, cellulose-binding protein
CLO1313_RS09200	Clo1313_1819	0.707	-0.5	7.90E-12	3.98E-11	copper amine oxidase
CLO1313_RS09205	Clo1313_1820	1.771	0.824	1.58E-26	1.83E-25	chemotaxis protein
CLO1313_RS09210	Clo1313_1821	1.121	0.164	0.054183897	0.08661914	hypothetical protein
CLO1313_RS09215	Clo1313_1822	1.008	0.012	0.880660165	0.905779526	hypothetical protein
CLO1313_RS09220	Clo1313_1823	0.634	-0.657	3.51E-13	1.95E-12	hypothetical protein
CLO1313_RS09225	Clo1313_1824	0.488	-1.036	2.11E-45	5.33E-44	sugar ABC transporter ATP-binding protein
CLO1313_RS09230	Clo1313_1825	0.796	-0.33	1.10E-05	3.07E-05	multidrug ABC transporter ATP-binding protein
CLO1313_RS09235	Clo1313_1826	1.515	0.599	9.28E-19	7.25E-18	RbsD or FucU transporter
CLO1313_RS09240	Clo1313_1827	1.106	0.146	0.057998619	0.091981945	alcohol dehydrogenase
CLO1313_RS09245	Clo1313_1828	1.12	0.164	0.037610735	0.062351904	sugar ABC transporter substrate-binding protein
CLO1313_RS09250	Clo1313_1829	0.874	-0.195	0.017574914	0.031243134	ABC transporter permease
CLO1313_RS09255	Clo1313_1830	0.977	-0.034	0.658840197	0.725086881	D-ribose transporter ATP-binding protein
CLO1313_RS09260	Clo1313_1831	1.218	0.285	0.000227514	0.000536832	ROK family transcriptional regulator
CLO1313_RS09265	Clo1313_1832	1.057	0.08	0.266541961	0.347759088	sugar kinase
CLO1313_RS09270	Clo1313_1833	1.328	0.409	1.27E-08	4.77E-08	alcohol dehydrogenase
CLO1313_RS09275	Clo1313_1834	0.879	-0.186	0.554633241	0.63173	hypothetical protein
CLO1313_RS09280	Clo1313_1838	1.753	0.81	0.10821119	0.157383782	peptide ABC transporter ATP-binding protein
CLO1313_RS09285		0.824	-0.279	0.640282433	0.709873204	hypothetical protein
CLO1313_RS09290		0.749	-0.417	0.460766014	0.543388626	hypothetical protein

CLO1313_RS09295		0.921	-0.118	0.799923498	0.841084722	hypothetical protein
CLO1313_RS09300	Clo1313_1840	1.069	0.097	0.33875331	0.424361394	hypothetical protein
CLO1313_RS09305	Clo1313_1841	0.972	-0.041	0.855103736	0.884295208	hypothetical protein
CLO1313_RS09310	Clo1313_1842	0.938	-0.093	0.551183388	0.629474098	hypothetical protein
CLO1313_RS09315	Clo1313_1843	0.939	-0.091	0.654754615	0.721915107	hypothetical protein
CLO1313_RS09320	Clo1313_1844	0.954	-0.068	0.668768143	0.73305397	peptidase M56
CLO1313_RS09325	Clo1313_1845	1.214	0.28	0.082574187	0.124881486	transcriptional regulator
CLO1313_RS09330	Clo1313_1846	0.818	-0.29	0.000568822	0.001258037	GMP synthase [glutamine-hydrolyzing]
CLO1313_RS09335	Clo1313_1847	3.517	1.815	2.58E-67	1.34E-65	glutamate dehydrogenase
CLO1313_RS09340	Clo1313_1848	1.08	0.111	0.240371288	0.316589149	ferredoxin-NADP+ reductase subunit alpha
CLO1313_RS09350	Clo1313_1850	1.584	0.664	8.69E-09	3.32E-08	transposase
CLO1313_RS09355	Clo1313_1851	1.171	0.228	0.000570348	0.001260482	radical SAM protein
CLO1313_RS09360	Clo1313_1852	1.162	0.217	0.000750576	0.001637073	hypothetical protein
CLO1313_RS09365	Clo1313_1853	1.349	0.431	5.71E-08	2.01E-07	1-(5-phosphoribosyl)-5-amino-4-imidazole- carboxylate carboxylase
CLO1313_RS09370	Clo1313_1854	1.228	0.296	2.57E-05	6.82E-05	NADP oxidoreductase
CLO1313_RS09375	Clo1313_1855	0.614	-0.703	1.02E-14	6.36E-14	copper amine oxidase
CLO1313_RS09380		0.86	-0.218	0.000755851	0.001647381	peptide chain release factor 2
CLO1313_RS09385	Clo1313_1857	1.788	0.839	1.31E-24	1.36E-23	XRE family transcriptional regulator
CLO1313_RS09390		0.827	-0.274	0.004010474	0.008002271	hypothetical protein
CLO1313_RS09395	Clo1313_1858	1.154	0.206	0.013423376	0.024353723	aminotransferase
CLO1313_RS09400	Clo1313_1859	1.068	0.095	0.28744015	0.370130102	AsnC family transcriptional regulator
CLO1313_RS09405	Clo1313_1861	1.299	0.378	1.40E-07	4.78E-07	membrane protein
CLO1313_RS09420	Clo1313_1864	1.604	0.682	0.000250592	0.000587589	transposase
CLO1313_RS09425		1.304	0.382	0.000102392	0.000253151	integrase
CLO1313_RS09435	Clo1313_1866	3.175	1.667	1.62E-06	4.98E-06	hypothetical protein
CLO1313_RS09440	Clo1313_1867	0.413	-1.275	4.65E-22	4.33E-21	alpha-glucan phosphorylase
CLO1313_RS09450	Clo1313_1869	1.061	0.085	0.272356214	0.353744602	hypothetical protein
CLO1313_RS09455	Clo1313_1870	1.477	0.563	0.005358412	0.010482634	2-hydroxyglutaryl-CoA dehydratase
CLO1313_RS09460	Clo1313_1871	1.303	0.381	0.110293713	0.160179586	hypothetical protein

CLO1313_RS09465	Clo1313_1872	1.332	0.414	0.078323677	0.119538274	hypothetical protein
CLO1313_RS09470	Clo1313_1873	1.221	0.288	1.00E-05	2.80E-05	peptidyl-prolyl cis-trans isomerase
CLO1313_RS09485	Clo1313_1876	0.682	-0.553	2.10E-16	1.45E-15	6-phosphofructokinase
CLO1313_RS09490	Clo1313_1877	1.533	0.616	7.20E-09	2.78E-08	hypothetical protein
CLO1313_RS09495	Clo1313_1878	0.764	-0.389	7.34E-08	2.57E-07	L-lactate dehydrogenase
CLO1313_RS09500	Clo1313_1879	0.821	-0.284	0.001482973	0.003129794	malate dehydrogenase
CLO1313_RS09505	Clo1313_1880	1.177	0.235	0.050658251	0.081591887	flavin reductase
CLO1313_RS09510	Clo1313_1881	0.457	-1.131	1.04E-30	1.40E-29	ferredoxin
CLO1313_RS09515	Clo1313_1882	0.443	-1.176	3.93E-32	5.56E-31	NADH dehydrogenase
CLO1313_RS09520	Clo1313_1883	0.431	-1.213	1.68E-41	3.74E-40	ferredoxin
CLO1313_RS09525	Clo1313_1884	0.471	-1.087	1.82E-35	3.11E-34	histidine kinase
CLO1313_RS09530	Clo1313_1885	0.513	-0.964	1.76E-32	2.57E-31	NADH dehydrogenase
CLO1313_RS09535	Clo1313_1886	1.065	0.091	0.38549138	0.471680395	phosphoesterase
CLO1313_RS09540	Clo1313_1887	1.208	0.272	0.008847591	0.016688003	AraC family transcriptional regulator
CLO1313_RS09545	Clo1313_1888	1.034	0.048	0.536605139	0.615638413	ferredoxin
CLO1313_RS09550	Clo1313_1889	0.805	-0.313	0.000196463	0.000467449	anti-sigma regulatory factor
CLO1313_RS09555	Clo1313_1890	0.909	-0.138	0.076317704	0.116848665	hypothetical protein
CLO1313_RS09560	Clo1313_1891	0.887	-0.174	0.018995682	0.033550088	ATPase AAA
CLO1313_RS09565	Clo1313_1892	1.045	0.063	0.481655445	0.562275079	hypothetical protein
CLO1313_RS09570		0.72	-0.474	9.84E-07	3.11E-06	hypothetical protein
CLO1313_RS09575	Clo1313_1894	1.242	0.313	0.000235983	0.00055594	hypothetical protein
CLO1313_RS09580	Clo1313_1895	1.124	0.169	0.619758834	0.691464562	hypothetical protein
CLO1313_RS09585	Clo1313_1896	0.47	-1.089	8.98E-37	1.63E-35	peptide chain release factor 3
CLO1313_RS09590	Clo1313_1897	0.483	-1.049	2.92E-18	2.21E-17	hypothetical protein
CLO1313_RS09595	Clo1313_1898	0.873	-0.196	0.094200786	0.139855523	transposase
CLO1313_RS09600	Clo1313_R0047	0.619	-0.691	1.61E-07	5.47E-07	
CLO1313_RS09605	Clo1313_R0048	0.635	-0.654	3.65E-07	1.20E-06	
CLO1313_RS09610	Clo1313_R0049	0.748	-0.42	0.000137336	0.000333437	
CLO1313_RS09615	Clo1313_1900	1.764	0.819	9.69E-20	7.99E-19	hypothetical protein
CLO1313_RS09620	Clo1313_1901	3.44	1.782	3.14E-96	2.77E-94	NAD synthetase

CLO1313_RS09625	Clo1313_1902	3.096	1.631	2.04E-66	1.02E-64	valine--tRNA ligase
CLO1313_RS09630	Clo1313_1903	1.362	0.446	7.48E-05	0.000188599	hypothetical protein
CLO1313_RS09635	Clo1313_1904	0.97	-0.044	0.649886432	0.71733876	glycoside hydrolase
CLO1313_RS09640	Clo1313_1905	1.203	0.266	0.000414358	0.000942123	hypothetical protein
CLO1313_RS09645	Clo1313_1906	1.052	0.073	0.279710623	0.361418423	hypothetical protein
CLO1313_RS09650	Clo1313_1907	1.008	0.012	0.89011719	0.912948513	aldolase
CLO1313_RS09655	Clo1313_1908	1.742	0.801	0.450945216	0.535174002	hypothetical protein
CLO1313_RS09660	Clo1313_1909	0.513	-0.964	5.99E-21	5.20E-20	hypothetical protein
CLO1313_RS09665	Clo1313_1910	1.04	0.056	0.711787762	0.767584142	hypothetical protein
CLO1313_RS09675	Clo1313_1912	0.66	-0.6	7.19E-10	3.06E-09	glycosyl transferase
CLO1313_RS09680	Clo1313_1913	0.609	-0.716	4.92E-14	2.94E-13	hypothetical protein
CLO1313_RS09685	Clo1313_1914	0.943	-0.085	0.222928537	0.296321122	ABC transporter ATPase
CLO1313_RS09690	Clo1313_1915	1.233	0.302	0.001751512	0.00366635	transposase
CLO1313_RS09695	Clo1313_1916	0.965	-0.051	0.473963788	0.555241172	excinuclease ABC subunit A
CLO1313_RS09700	Clo1313_1917	1.382	0.467	5.97E-08	2.10E-07	hypothetical protein
CLO1313_RS09705	Clo1313_1918	1.05	0.07	0.460644049	0.543388626	excinuclease ABC subunit B
CLO1313_RS09715	Clo1313_1920	0.729	-0.455	6.93E-13	3.74E-12	hypothetical protein
CLO1313_RS09720	Clo1313_1921	1.08	0.11	0.153911492	0.214137179	topoisomerase IV
CLO1313_RS09725	Clo1313_1922	1.054	0.076	0.248227129	0.32636263	DNA topoisomerase
CLO1313_RS09730	Clo1313_1923	1.195	0.257	0.023175023	0.040128114	hypothetical protein
CLO1313_RS09735	Clo1313_1924	1.667	0.737	0.004783908	0.009444991	hypothetical protein
CLO1313_RS09740	Clo1313_1925	2.151	1.105	0.000717448	0.001570531	hypothetical protein
CLO1313_RS09745	Clo1313_1926	2.858	1.515	5.65E-06	1.65E-05	hypothetical protein
CLO1313_RS09750	Clo1313_1927	1.791	0.841	1.46E-08	5.46E-08	hypothetical protein
CLO1313_RS09755	Clo1313_1928	1.331	0.412	3.81E-08	1.36E-07	MATE family efflux transporter
CLO1313_RS09760	Clo1313_1929	1.745	0.803	2.34E-19	1.89E-18	chemotaxis protein
CLO1313_RS09765	Clo1313_1930	1.025	0.036	0.664868218	0.729846188	hypothetical protein
CLO1313_RS09770	Clo1313_1931	0.909	-0.138	0.207712846	0.278218323	phosphoserine aminotransferase
CLO1313_RS09775		0.844	-0.244	0.23050636	0.304532411	methyltransferase type 11
CLO1313_RS09780		0.583	-0.778	0.00078594	0.001707995	hypothetical protein

CLO1313_RS09785	Clo1313_1933	1.121	0.165	0.333881195	0.418782812	hypothetical protein
CLO1313_RS09790		1.351	0.434	0.012666995	0.023121314	hypothetical protein
CLO1313_RS09795	Clo1313_1935	1.287	0.364	0.000119556	0.000292215	transposase
CLO1313_RS09800		2.009	1.007	4.89E-15	3.14E-14	hypothetical protein
CLO1313_RS09805	Clo1313_1936	0.868	-0.204	0.22764482	0.301282796	hypothetical protein
CLO1313_RS09810	Clo1313_1937	1.139	0.188	0.227761078	0.301303694	hypothetical protein
CLO1313_RS09815	Clo1313_1938	1.179	0.237	0.003632985	0.007287842	homoserine dehydrogenase
CLO1313_RS09820	Clo1313_1939	1.285	0.362	0.01736263	0.030920741	ATP-dependent helicase
CLO1313_RS09825	Clo1313_1940	1.049	0.069	0.544748185	0.624264351	chemotaxis protein CheR
CLO1313_RS09835	Clo1313_1942	1.11	0.151	0.166406825	0.230084863	two-component system sensor histidine kinase/response regulator
CLO1313_RS09840	Clo1313_1944	0.859	-0.22	0.004529372	0.008977915	isocitrate dehydrogenase
CLO1313_RS09845	Clo1313_1945	1.213	0.278	0.001848586	0.003852613	hypothetical protein
CLO1313_RS09850	Clo1313_1946	0.885	-0.177	0.074064959	0.11367493	voltage-gated potassium channel
CLO1313_RS09855	Clo1313_1947	1.056	0.078	0.491767177	0.572075161	glycogen synthase
CLO1313_RS09860	Clo1313_1948	0.871	-0.199	0.082446218	0.124750861	coat protein F
CLO1313_RS09865	Clo1313_1949	0.962	-0.057	0.61372917	0.686013336	hypothetical protein
CLO1313_RS09870	Clo1313_1950	0.91	-0.136	0.101262958	0.148720672	pseudouridine synthase
CLO1313_RS09875	Clo1313_1951	0.811	-0.302	0.000526012	0.00117026	SAM-dependent methyltransferase
CLO1313_RS09880	Clo1313_1953	0.836	-0.258	0.003798379	0.007599291	2-hydroxyacid dehydrogenase
CLO1313_RS09885	Clo1313_1954	0.591	-0.759	2.35E-27	2.81E-26	glycosyl transferase
CLO1313_RS09890	Clo1313_1955	1.851	0.889	4.88E-20	4.09E-19	glycoside hydrolase
CLO1313_RS09895	Clo1313_1956	1.118	0.162	0.033919424	0.056734162	phosphohydrolase
CLO1313_RS09900	Clo1313_1957	1.105	0.144	0.106504746	0.155278431	penicillin-binding protein 6
CLO1313_RS09905	Clo1313_1958	1.199	0.262	0.00092725	0.002003475	type 3a, cellulose-binding protein
CLO1313_RS09910	Clo1313_1959	1.863	0.898	1.30E-32	1.92E-31	glycoside hydrolase
CLO1313_RS09915	Clo1313_1960	1.144	0.195	0.007827811	0.014905146	endoglucanase
CLO1313_RS09925	Clo1313_1962	1.623	0.698	9.96E-15	6.21E-14	type 3a, cellulose-binding protein
CLO1313_RS09930	Clo1313_1963	0.07	-3.832	1.35E-15	8.95E-15	chemotaxis protein
CLO1313_RS09935	Clo1313_1964	0.008	-6.937	9.73E-46	2.52E-44	hypothetical protein

CLO1313_RS09940	Clo1313_1965	1.691	0.758	0.000445681	0.001009516	hypothetical protein
CLO1313_RS09945	Clo1313_1966	1.186	0.246	0.620902028	0.692224975	hypothetical protein
CLO1313_RS09950	Clo1313_1967	1.109	0.15	0.067272502	0.104642237	gamma-glutamyl phosphate reductase
CLO1313_RS09955	Clo1313_1968	0.912	-0.133	0.191768268	0.259527544	haloacid dehalogenase
CLO1313_RS09960	Clo1313_1969	8.967	3.165	2.10E-118	2.52E-116	peptidase S1
CLO1313_RS09965	Clo1313_1970	1.283	0.359	6.02E-05	0.000153601	peptidase S54
CLO1313_RS09970	Clo1313_1971	1.114	0.156	0.121651831	0.174728851	LytTR family transcriptional regulator
CLO1313_RS09975	Clo1313_1972	2.841	1.507	1.56E-11	7.64E-11	twitching motility protein PilT
CLO1313_RS09980	Clo1313_1973	1.135	0.182	0.175108543	0.240673933	histidine kinase
CLO1313_RS09985	Clo1313_1974	0.876	-0.192	0.104339443	0.152492198	hypothetical protein
CLO1313_RS09990	Clo1313_1975	1.185	0.245	0.001655803	0.003479855	hypothetical protein
CLO1313_RS09995	Clo1313_1976	1.073	0.101	0.194733069	0.262710065	ATPase
CLO1313_RS10000	Clo1313_1977	1.119	0.163	0.058956377	0.093254312	hypothetical protein
CLO1313_RS10005	Clo1313_1978	0.935	-0.097	0.175944484	0.24149085	transglutaminase
CLO1313_RS10010	Clo1313_1979	0.955	-0.066	0.437120536	0.521864844	hypothetical protein
CLO1313_RS10015	Clo1313_1980	0.897	-0.157	0.031834167	0.053575009	peptidase C11
CLO1313_RS10020	Clo1313_1981	1.241	0.312	0.000320197	0.000740379	transposase
CLO1313_RS10025	Clo1313_1982	0.977	-0.034	0.729962491	0.783801471	DNA mismatch repair protein MutS
CLO1313_RS10030	Clo1313_1983	1.19	0.251	0.080207363	0.122067779	carbohydrate-binding protein
CLO1313_RS10035	Clo1313_1984	2.711	1.439	0.000171824	0.000413231	ArsR family transcriptional regulator
CLO1313_RS10045	Clo1313_1986	0.947	-0.079	0.439627081	0.523814706	copper amine oxidase
CLO1313_RS10050	Clo1313_1987	0.639	-0.647	2.83E-10	1.26E-09	hypothetical protein
CLO1313_RS10055	Clo1313_1988	1.673	0.742	3.13E-07	1.04E-06	cell division protein FtsZ
CLO1313_RS10060	Clo1313_1989	2.48	1.31	4.49E-13	2.48E-12	VTC domain protein
CLO1313_RS10065	Clo1313_1990	2.543	1.347	2.58E-14	1.57E-13	dockerin
CLO1313_RS10070		1.822	0.865	0.000101855	0.00025224	hypothetical protein
CLO1313_RS10080	Clo1313_1992	1.641	0.714	1.20E-06	3.76E-06	aldo/keto reductase
CLO1313_RS10085	Clo1313_1993	2.265	1.18	3.75E-32	5.35E-31	glutaredoxin
CLO1313_RS10090	Clo1313_1994	2.543	1.347	3.08E-58	1.18E-56	AMP-dependent synthetase
CLO1313_RS10095	Clo1313_1995	1.076	0.106	0.286090669	0.368709031	GCN5 family N-acetyltransferase

CLO1313_RS10100	Clo1313_1996	1.014	0.02	0.823943208	0.859480237	hypothetical protein
CLO1313_RS10105	Clo1313_1997	1.325	0.406	4.60E-08	1.64E-07	radical SAM/SPASM domain-containing protein
CLO1313_RS10110	Clo1313_1998	0.982	-0.026	0.700915206	0.759409214	hypothetical protein
CLO1313_RS10115	Clo1313_1999	1.609	0.686	1.46E-15	9.70E-15	NAD-dependent epimerase
CLO1313_RS10120	Clo1313_2000	1.433	0.519	2.94E-09	1.18E-08	hypothetical protein
CLO1313_RS10125	Clo1313_2001	1.723	0.785	3.40E-13	1.89E-12	hypothetical protein
CLO1313_RS10130	Clo1313_2002	1.574	0.654	5.87E-12	2.99E-11	hypothetical protein
CLO1313_RS10135		1.735	0.795	6.25E-05	0.000158909	transposase
CLO1313_RS10140		1.532	0.616	5.99E-05	0.000152969	transposase
CLO1313_RS10145	Clo1313_2004	1.628	0.703	7.38E-09	2.85E-08	DNA helicase UvrD
CLO1313_RS10150	Clo1313_2005	1.55	0.632	2.70E-09	1.08E-08	chromosome segregation protein SMC
CLO1313_RS10155	Clo1313_2006	2.08	1.057	2.91E-15	1.91E-14	hypothetical protein
CLO1313_RS10165	Clo1313_2008	1.537	0.62	9.51E-07	3.01E-06	transposase
CLO1313_RS10175	Clo1313_2009	0.506	-0.982	0.046993786	0.076139581	hypothetical protein
CLO1313_RS10180	Clo1313_2010	0.839	-0.254	0.317016117	0.400814222	hypothetical protein
CLO1313_RS10185	Clo1313_2012	0.92	-0.121	0.453982459	0.538139681	MFS transporter
CLO1313_RS10190	Clo1313_R0050	0.628	-0.671	0.031806591	0.053558656	
CLO1313_RS10195	Clo1313_2014	0.936	-0.095	0.277096251	0.358658462	metallophosphoesterase
CLO1313_RS10200	Clo1313_2015	0.813	-0.298	0.000307059	0.000711647	glucose-6-phosphate isomerase
CLO1313_RS10205	Clo1313_2016	0.752	-0.41	0.000193141	0.000460436	spore protein
CLO1313_RS10210	Clo1313_2017	0.768	-0.381	0.001303091	0.002773577	phenylalanine--tRNA ligase subunit beta
CLO1313_RS10215	Clo1313_2018	0.768	-0.38	0.002093516	0.004332957	phenylalanine--tRNA ligase subunit alpha
CLO1313_RS10220	Clo1313_2019	0.836	-0.258	0.000188826	0.000451226	hypothetical protein
CLO1313_RS10225	Clo1313_2020	0.935	-0.096	0.213953828	0.286066665	beta-glucosidase
CLO1313_RS10230	Clo1313_2021	2.045	1.032	1.21E-22	1.14E-21	hypothetical protein
CLO1313_RS10235	Clo1313_2022	0.872	-0.198	0.10356235	0.151652093	beta-glucanase
CLO1313_RS10240	Clo1313_2023	1.456	0.542	7.95E-13	4.26E-12	LacI family transcriptional regulator
CLO1313_RS10245	Clo1313_2024	1.476	0.562	2.10E-05	5.63E-05	glycosyl transferase
CLO1313_RS10250		1.635	0.71	7.37E-19	5.80E-18	phosphoesterase
CLO1313_RS10255		1.465	0.551	4.00E-10	1.73E-09	phosphoesterase RecJ

CLO1313_RS10260	Clo1313_2026	1.577	0.657	8.52E-12	4.28E-11	hypothetical protein
CLO1313_RS10265	Clo1313_2027	1.398	0.483	2.58E-10	1.15E-09	helicase UvrD
CLO1313_RS10270	Clo1313_2028	1.343	0.425	5.23E-08	1.85E-07	hypothetical protein
CLO1313_RS10275	Clo1313_2029	1.564	0.645	2.77E-10	1.23E-09	hypothetical protein
CLO1313_RS10280	Clo1313_2030	2.235	1.16	1.30E-35	2.24E-34	transcriptional regulator
CLO1313_RS10290	Clo1313_2032	1.994	0.996	1.16E-10	5.34E-10	glutamate synthase
CLO1313_RS10295	Clo1313_2033	2.563	1.358	4.06E-05	0.00010551	pyridine nucleotide-disulfide oxidoreductase
CLO1313_RS10300	Clo1313_2034	19.285	4.269	2.70E-147	5.40E-145	4Fe-4S ferredoxin
CLO1313_RS10305	Clo1313_2035	17.748	4.15	3.46E-173	1.15E-170	glutamate synthase
CLO1313_RS10310	Clo1313_2036	19.104	4.256	1.49E-239	4.47E-236	hypothetical protein
CLO1313_RS10315	Clo1313_2038	3.137	1.649	1.15E-64	5.47E-63	glutamine synthetase
CLO1313_RS10320		0.941	-0.088	0.342668326	0.428014289	RNA polymerase subunit sigma-24
CLO1313_RS10325	Clo1313_2040	1.168	0.224	0.004910998	0.009670442	hypothetical protein
CLO1313_RS10330	Clo1313_2041	0.767	-0.383	0.09055691	0.134846164	potassium transporter
CLO1313_RS10335	Clo1313_R0051	0.869	-0.202	0.078999132	0.120446568	
CLO1313_RS10340	Clo1313_R0052	0.797	-0.327	0.009066629	0.017062691	
CLO1313_RS10345	Clo1313_2042	1.658	0.729	2.59E-05	6.85E-05	proteinase IV
CLO1313_RS10350	Clo1313_2043	0.836	-0.258	0.049662022	0.080030308	proteinase IV
CLO1313_RS10355	Clo1313_2044	0.759	-0.398	1.90E-05	5.14E-05	nucleoside-triphosphate diphosphatase
CLO1313_RS10360	Clo1313_2045	0.707	-0.501	9.86E-09	3.74E-08	ribonuclease PH
CLO1313_RS10365	Clo1313_2046	0.876	-0.191	0.016695606	0.029839166	sporulation protein
CLO1313_RS10375	Clo1313_2048	0.907	-0.141	0.110864653	0.16093083	cell wall hydrolase
CLO1313_RS10380	Clo1313_2049	1.156	0.209	0.004767145	0.009424304	pilus assembly protein PilM
CLO1313_RS10385	Clo1313_2050	0.904	-0.145	0.088712314	0.132296485	hypothetical protein
CLO1313_RS10390	Clo1313_2051	0.993	-0.01	0.891920216	0.914172497	Holliday junction DNA helicase RuvB
CLO1313_RS10395	Clo1313_2052	0.963	-0.054	0.524368886	0.603678422	Holliday junction DNA helicase RuvA
CLO1313_RS10400	Clo1313_2053	0.973	-0.04	0.678540702	0.741867869	crossover junction endodeoxyribonuclease RuvC
CLO1313_RS10405	Clo1313_2054	0.585	-0.775	1.10E-13	6.35E-13	argininosuccinate synthase
CLO1313_RS10415	Clo1313_2056	0.942	-0.087	0.640965494	0.710105473	metal-dependent phosphohydrolase
CLO1313_RS10420	Clo1313_2057	1.496	0.581	1.99E-11	9.66E-11	hypothetical protein

CLO1313_RS10425	Clo1313_2058	0.914	-0.129	0.037022049	0.061477921	polysaccharide deacetylase
CLO1313_RS10430	Clo1313_2059	0.861	-0.217	0.000423981	0.000963272	sulfatase
CLO1313_RS10435	Clo1313_2060	1.337	0.419	0.362898035	0.449537879	dehydrogenase
CLO1313_RS10440	Clo1313_2061	0.268	-1.897	0.357307449	0.443893533	hypothetical protein
CLO1313_RS10445	Clo1313_2062	6.623	2.727	0.198629129	0.267484849	hypothetical protein
CLO1313_RS10450	Clo1313_2063	1.024	0.035	0.961277002	0.969031842	hypothetical protein
CLO1313_RS10455	Clo1313_2064	0.95	-0.075	0.825429082	0.860196198	dehydrogenase
CLO1313_RS10460	Clo1313_2065	0.788	-0.343	0.099087379	0.145739603	hypothetical protein
CLO1313_RS10465	Clo1313_2066	0.912	-0.133	0.374292936	0.460089093	chemotaxis protein CheY
CLO1313_RS10470	Clo1313_2067	0.846	-0.242	0.021972782	0.038178664	4Fe-4S ferredoxin
CLO1313_RS10475	Clo1313_2068	1.41	0.495	1.00E-09	4.21E-09	hypothetical protein
CLO1313_RS10480	Clo1313_2069	1.313	0.393	0.000568302	0.001257815	RNA-binding protein
CLO1313_RS10485	Clo1313_2070	1.149	0.2	0.011296612	0.020874023	hypothetical protein
CLO1313_RS10490	Clo1313_2071	0.787	-0.346	0.000133682	0.000325151	GTPase Obg
CLO1313_RS10495	Clo1313_2072	0.632	-0.663	1.73E-23	1.70E-22	50S ribosomal protein L27
CLO1313_RS10500	Clo1313_2073	0.494	-1.018	1.37E-52	4.45E-51	hypothetical protein
CLO1313_RS10510	Clo1313_2075	1.157	0.21	0.011461099	0.021125898	ribonuclease G
CLO1313_RS10515	Clo1313_2076	1.044	0.062	0.402623448	0.488314161	hypothetical protein
CLO1313_RS10520	Clo1313_2077	1.153	0.205	0.003611462	0.007249515	B12-binding protein
CLO1313_RS10525	Clo1313_2078	0.949	-0.076	0.495838275	0.574804401	hypothetical protein
CLO1313_RS10530	Clo1313_2079	0.909	-0.137	0.202232512	0.271362552	hypothetical protein
CLO1313_RS10535	Clo1313_2080	0.956	-0.065	0.532033707	0.610861059	Holliday junction DNA helicase
CLO1313_RS10540	Clo1313_2081	0.976	-0.035	0.752299116	0.801757302	aldo/keto reductase
CLO1313_RS10545	Clo1313_2082	0.862	-0.215	0.029277152	0.049661866	hypothetical protein
CLO1313_RS10550	Clo1313_2083	0.761	-0.394	1.01E-06	3.18E-06	30S ribosomal protein S12 methylthiotransferase
CLO1313_RS10555	Clo1313_2084	1.145	0.196	0.025428076	0.043651287	HPr kinase
CLO1313_RS10560	Clo1313_2085	1.114	0.155	0.121502354	0.174597776	hypothetical protein
CLO1313_RS10570	Clo1313_2087	1.095	0.131	0.221124393	0.294341791	transposase
CLO1313_RS10575	Clo1313_2088	0.891	-0.167	0.019028332	0.033568216	phosphohydrolase
CLO1313_RS10580	Clo1313_2089	0.892	-0.165	0.011258359	0.020816164	preprotein translocase subunit SecG

CLO1313_RS10585	Clo1313_2090	0.915	-0.128	0.168829854	0.233005399	enolase
CLO1313_RS10590	Clo1313_2091	0.718	-0.478	1.47E-06	4.56E-06	phosphohydrolase
CLO1313_RS10595		2.406	1.266	0.019419638	0.034138038	hypothetical protein
CLO1313_RS10600	Clo1313_2092	1.223	0.291	0.000777326	0.001691728	2,3-bisphosphoglycerate-independent phosphoglycerate mutase
CLO1313_RS10605	Clo1313_2093	0.778	-0.362	5.27E-06	1.54E-05	triosephosphate isomerase
CLO1313_RS10620	Clo1313_2096	1.066	0.092	0.40460395	0.490265554	4'-phosphopantetheinyl transferase
CLO1313_RS10625	Clo1313_2097	0.499	-1.004	3.13E-38	6.14E-37	beta-ketoacyl synthase
CLO1313_RS10630	Clo1313_2098	0.438	-1.19	1.63E-34	2.64E-33	hypothetical protein
CLO1313_RS10635	Clo1313_2099	0.392	-1.351	2.51E-37	4.68E-36	AMP-dependent synthetase
CLO1313_RS10640	Clo1313_2100	0.455	-1.135	1.26E-29	1.68E-28	3-oxoacyl-ACP synthase
CLO1313_RS10645	Clo1313_2101	0.613	-0.707	7.88E-10	3.34E-09	acyl carrier protein
CLO1313_RS10650	Clo1313_2102	0.577	-0.793	4.83E-22	4.49E-21	3-oxoacyl-ACP synthase
CLO1313_RS10655	Clo1313_2103	0.625	-0.679	5.07E-20	4.23E-19	phosphohydrolase
CLO1313_RS10665	Clo1313_2105	0.707	-0.5	0.068077047	0.10572919	chemotaxis protein CheC
CLO1313_RS10670	Clo1313_2106	1.052	0.073	0.72486448	0.779443734	stage V sporulation protein AE
CLO1313_RS10690	Clo1313_2110	0.564	-0.827	0.466614991	0.548775827	hypothetical protein
CLO1313_RS10700	Clo1313_2112	1.139	0.188	0.355215527	0.441663087	ATPase
CLO1313_RS10710	Clo1313_2114	1.067	0.094	0.367702406	0.454550501	phosphate ABC transporter permease
CLO1313_RS10720	Clo1313_2116	0.916	-0.126	0.080505472	0.122307959	glycogen debranching protein
CLO1313_RS10725	Clo1313_2117	0.919	-0.122	0.133860079	0.189809162	hypothetical protein
CLO1313_RS10730	Clo1313_2118	0.99	-0.015	0.854437013	0.883910522	glmZ(sRNA)-inactivating NTPase
CLO1313_RS10735	Clo1313_2119	1.042	0.059	0.420451482	0.505181889	UDP-N-acetylenolpyruvoylglucosamine reductase
CLO1313_RS10740	Clo1313_2120	0.978	-0.033	0.643982374	0.712395108	phosphatase
CLO1313_RS10750	Clo1313_2122	1.246	0.318	0.337584486	0.423073913	dockerin
CLO1313_RS10755	Clo1313_R0053	0.449	-1.156	2.03E-11	9.81E-11	
CLO1313_RS10760	Clo1313_R0054	0.555	-0.85	8.11E-26	9.01E-25	
CLO1313_RS10765	Clo1313_2123	0.895	-0.16	0.112192799	0.162622622	pilus assembly protein PilZ
CLO1313_RS10770	Clo1313_2124	1.667	0.737	2.87E-16	1.96E-15	6,7-dimethyl-8-ribityllumazine synthase
CLO1313_RS10775	Clo1313_2125	1.544	0.627	1.74E-08	6.43E-08	3,4-dihydroxy-2-butanone 4-phosphate synthase

CLO1313_RS10780	Clo1313_2126	1.255	0.328	0.011630206	0.021358842	riboflavin synthase subunit alpha
CLO1313_RS10790	Clo1313_2128	1.35	0.433	1.50E-06	4.65E-06	hypothetical protein
CLO1313_RS10795	Clo1313_2129	0.701	-0.513	1.92E-06	5.87E-06	tRNA (guanine-N(7)-)-methyltransferase
CLO1313_RS10800	Clo1313_2130	1.148	0.199	0.029847511	0.050600726	NADH-dependent butanol dehydrogenase
CLO1313_RS10805	Clo1313_2131	1.021	0.029	0.706522964	0.763308974	hypothetical protein
CLO1313_RS10815	Clo1313_2133	1.048	0.067	0.780906343	0.826371955	peptidase M50
CLO1313_RS10820	Clo1313_2134	1.193	0.254	0.081480248	0.123538556	peptidase M23B
CLO1313_RS10825	Clo1313_2135	1.117	0.159	0.072688007	0.111790428	hypothetical protein
CLO1313_RS10830	Clo1313_2136	1.365	0.448	4.94E-08	1.75E-07	methylglyoxal synthase
CLO1313_RS10835	Clo1313_2137	1.335	0.417	2.89E-07	9.61E-07	cell division topological specificity factor
CLO1313_RS10845	Clo1313_2139	1.187	0.248	0.002865476	0.005834055	septum site-determining protein MinC
CLO1313_RS10865	Clo1313_2143	0.944	-0.083	0.314552612	0.398203159	rod shape-determining protein Mbl
CLO1313_RS10870	Clo1313_2144	0.917	-0.124	0.108082265	0.15734889	maf-like protein
CLO1313_RS10875	Clo1313_2145	0.594	-0.751	0.018896491	0.033414255	hypothetical protein
CLO1313_RS10880	Clo1313_2146	1.549	0.631	1.08E-10	4.99E-10	adapter protein MecA
CLO1313_RS10885	Clo1313_2147	1.245	0.316	0.00011514	0.000282573	hypothetical protein
CLO1313_RS10890	Clo1313_2148	0.858	-0.222	0.004572383	0.009051206	serine phosphatase
CLO1313_RS10900	Clo1313_2150	0.898	-0.155	0.058555248	0.092766608	ornithine acetyltransferase
CLO1313_RS10905	Clo1313_2151	1.042	0.059	0.57700759	0.652505944	chemotaxis protein CheW
CLO1313_RS10910	Clo1313_2152	1.524	0.608	3.10E-05	8.13E-05	amidase
CLO1313_RS10915	Clo1313_2153	0.821	-0.285	0.010869949	0.020160159	hypothetical protein
CLO1313_RS10920	Clo1313_2154	1.294	0.371	7.66E-06	2.18E-05	hypothetical protein
CLO1313_RS10925	Clo1313_2155	1.144	0.194	0.055731908	0.088856986	hypothetical protein
CLO1313_RS10930	Clo1313_2156	0.9	-0.152	0.433881295	0.519030716	hypothetical protein
CLO1313_RS10935		0.701	-0.512	0.136054826	0.192375494	RNA polymerase subunit sigma-24
CLO1313_RS10940	Clo1313_2159	0.871	-0.2	0.266588244	0.347759088	hypothetical protein
CLO1313_RS10945	Clo1313_2160	0.784	-0.351	0.062959775	0.098547164	phage-shock protein
CLO1313_RS10950	Clo1313_2161	1.107	0.147	0.105167745	0.1535531	cellulose 1,4-beta-cellobiosidase
CLO1313_RS10955	Clo1313_2162	0.747	-0.421	1.61E-09	6.57E-09	asparagine--tRNA ligase
CLO1313_RS10960	Clo1313_2163	0.636	-0.653	2.10E-22	1.98E-21	asparagine synthase

CLO1313_RS10965	Clo1313_2164	0.982	-0.027	0.686063134	0.74693395	peptidylprolyl isomerase
CLO1313_RS10970	Clo1313_2165	1.616	0.693	1.52E-14	9.35E-14	NAD-dependent deacetylase
CLO1313_RS10975	Clo1313_2166	1.368	0.452	0.033276083	0.055844977	hypothetical protein
CLO1313_RS10980	Clo1313_2167	1.005	0.007	0.970855885	0.977045905	hypothetical protein
CLO1313_RS10985	Clo1313_2168	0.93	-0.105	0.255149441	0.33429147	Na/Pi cotransporter
CLO1313_RS10990	Clo1313_2169	0.907	-0.142	0.145212253	0.203121057	rubredoxin
CLO1313_RS10995	Clo1313_2170	0.423	-1.24	5.52E-40	1.17E-38	sulfatase
CLO1313_RS11000	Clo1313_2171	1.037	0.053	0.507996351	0.586405334	biotin biosynthesis protein BioY
CLO1313_RS11005	Clo1313_2172	0.862	-0.215	0.002143182	0.00442354	preprotein translocase subunit SecA
CLO1313_RS11010	Clo1313_2173	1.021	0.03	0.743159588	0.794139087	type 3a, cellulose-binding protein
CLO1313_RS11020	Clo1313_2175	0.825	-0.278	0.579924697	0.655310537	hypothetical protein
CLO1313_RS11025	Clo1313_2176	0.781	-0.356	0.000520139	0.001158052	hypothetical protein
CLO1313_RS11030	Clo1313_2177	1.162	0.216	0.239346313	0.315516305	hypothetical protein
CLO1313_RS11035	Clo1313_2178	1.126	0.171	0.041320429	0.067938579	hypothetical protein
CLO1313_RS11040	Clo1313_2179	0.861	-0.215	0.051224766	0.08246005	ribonucleotide-diphosphate reductase subunit alpha
CLO1313_RS11045	Clo1313_2180	0.7	-0.515	1.04E-11	5.20E-11	hypothetical protein
CLO1313_RS11050	Clo1313_2181	0.861	-0.216	0.478196255	0.558671822	GTP-binding protein
CLO1313_RS11055	Clo1313_2182	0.574	-0.801	0.071914267	0.110884775	hypothetical protein
CLO1313_RS11060	Clo1313_2183	0.691	-0.534	0.000292414	0.00068178	DNA-binding protein
CLO1313_RS11065	Clo1313_2184	0.513	-0.963	2.41E-05	6.41E-05	copper amine oxidase
CLO1313_RS11070	Clo1313_2185	0.434	-1.203	0.000242561	0.000570096	copper amine oxidase
CLO1313_RS11075	Clo1313_2186	0.496	-1.013	7.97E-34	1.26E-32	hypothetical protein
CLO1313_RS11080	Clo1313_2187	0.548	-0.867	6.24E-15	3.97E-14	copper amine oxidase
CLO1313_RS11085	Clo1313_2188	1.157	0.21	0.188143913	0.256008891	dockerin
CLO1313_RS11090	Clo1313_2189	2.111	1.078	5.40E-27	6.35E-26	glycoside hydrolase
CLO1313_RS11100	Clo1313_2191	0.952	-0.071	0.597323056	0.671177162	hypothetical protein
CLO1313_RS11105	Clo1313_2192	0.955	-0.067	0.352298997	0.438764406	endoglucanase
CLO1313_RS11110	Clo1313_2193	2.575	1.364	9.65E-34	1.50E-32	chemotaxis protein
CLO1313_RS11115	Clo1313_2194	3.139	1.65	9.55E-07	3.02E-06	chemotaxis protein CheW
CLO1313_RS11120	Clo1313_2195	2.884	1.528	2.91E-80	1.86E-78	chemotaxis protein

CLO1313_RS11125	Clo1313_2196	2.332	1.221	2.24E-38	4.43E-37	chemotaxis protein CheW
CLO1313_RS11130	Clo1313_2197	0.749	-0.418	0.690695873	0.750234307	heat-shock protein Hsp20
CLO1313_RS11135	Clo1313_2198	1.162	0.216	0.234437227	0.309453012	hydroxylamine reductase
CLO1313_RS11140	Clo1313_2199	1.205	0.269	0.38923711	0.475294012	(Fe-S)-binding protein
CLO1313_RS11145	Clo1313_2200	1.101	0.139	0.139002638	0.195346256	Crp/Fnr family transcriptional regulator
CLO1313_RS11150	Clo1313_2201	0.863	-0.212	0.002066868	0.004285613	small GTP-binding protein
CLO1313_RS11155	Clo1313_2202	1.35	0.433	9.93E-05	0.00024677	glycoside hydrolase
CLO1313_RS11160	Clo1313_2203	0.377	-1.406	9.71E-36	1.70E-34	hypothetical protein
CLO1313_RS11165	Clo1313_2204	0.897	-0.157	0.176129267	0.241544198	nucleotidyltransferase
CLO1313_RS11170		1.277	0.353	0.085241815	0.128269043	hypothetical protein
CLO1313_RS11175		1.167	0.223	0.422478723	0.507008279	chloride transporter
CLO1313_RS11180		1.108	0.147	0.401558464	0.487757729	anthranilate synthase
CLO1313_RS11185		0.993	-0.011	0.956329368	0.964636806	hypothetical protein
CLO1313_RS11190		0.911	-0.135	0.29927606	0.38176474	para-aminobenzoate synthase
CLO1313_RS11205	Clo1313_2209	1.046	0.065	0.707303622	0.763572197	pimeloyl-CoA synthetase
CLO1313_RS11220	Clo1313_2212	1.166	0.221	0.347355474	0.432967192	biotin synthase
CLO1313_RS11225		1.287	0.364	2.23E-05	5.97E-05	nitrogen regulatory protein P-II
CLO1313_RS11230		1.141	0.19	0.026246897	0.044979682	hypothetical protein
CLO1313_RS11235	Clo1313_2214	0.94	-0.089	0.266467835	0.347759088	hypothetical protein
CLO1313_RS11240	Clo1313_2215	0.742	-0.43	0.000345749	0.000793951	ferritin
CLO1313_RS11245	Clo1313_2216	1.555	0.636	5.80E-06	1.68E-05	alpha-L-arabinofuranosidase
CLO1313_RS11250	Clo1313_2217	1.736	0.796	0.216289679	0.288803539	hypothetical protein
CLO1313_RS11255		3.522	1.816	0.074475835	0.114247074	sugar-binding protein
CLO1313_RS11260		1.371	0.455	0.323324557	0.407416111	hypothetical protein
CLO1313_RS11265	Clo1313_2219	1.288	0.365	0.216963511	0.289574353	hypothetical protein
CLO1313_RS11270		1.176	0.234	0.009563435	0.017903085	hypothetical protein
CLO1313_RS11280	Clo1313_2221	0.484	-1.048	0.00037167	0.000850869	hypothetical protein
CLO1313_RS11285	Clo1313_2222	0.72	-0.473	0.012980698	0.023622034	hypothetical protein
CLO1313_RS11290		1.45	0.536	0.395541335	0.481814973	hypothetical protein
CLO1313_RS11300	Clo1313_2224	1.591	0.67	4.02E-10	1.74E-09	transposase

CLO1313_RS11305	Clo1313_2225	1.204	0.268	0.041392536	0.068019845	XRE family transcriptional regulator
CLO1313_RS11310	Clo1313_2226	1.02	0.029	0.848948381	0.879446008	hypothetical protein
CLO1313_RS11315		0.99	-0.014	0.915942166	0.93382508	hypothetical protein
CLO1313_RS11320	Clo1313_2228	0.896	-0.159	0.331209524	0.416302331	peroxiredoxin
CLO1313_RS11325	Clo1313_2229	1.6	0.678	5.92E-06	1.72E-05	hypothetical protein
CLO1313_RS11330		2.831	1.501	0.170818416	0.235641412	hypothetical protein
CLO1313_RS11335		1.196	0.259	0.004963134	0.009766691	hypothetical protein
CLO1313_RS11340	Clo1313_2232	1.103	0.141	0.217442374	0.289955393	RNA polymerase subunit sigma-24
CLO1313_RS11345	Clo1313_2233	2.02	1.014	8.07E-21	6.98E-20	glycoside hydrolase
CLO1313_RS11350	Clo1313_2234	0.819	-0.288	0.000608667	0.001338265	endoglucanase
CLO1313_RS11355	Clo1313_2235	0.962	-0.056	0.659717451	0.725786	FMN reductase
CLO1313_RS11360		2.837	1.504	3.77E-09	1.49E-08	hypothetical protein
CLO1313_RS11365	Clo1313_2237	2.339	1.226	1.10E-33	1.71E-32	hypothetical protein
CLO1313_RS11370	Clo1313_2238	2.006	1.004	3.25E-27	3.85E-26	hypothetical protein
CLO1313_RS11375		1.996	0.997	2.57E-21	2.30E-20	hypothetical protein
CLO1313_RS11385	Clo1313_2240	1.557	0.639	1.11E-11	5.53E-11	TetR family transcriptional regulator
CLO1313_RS11390	Clo1313_2241	0.956	-0.065	0.88203098	0.905894147	hypothetical protein
CLO1313_RS11395	Clo1313_2242	1.342	0.424	0.097384789	0.143658131	hypothetical protein
CLO1313_RS11400	Clo1313_2243	1.2	0.263	0.036875764	0.061319681	membrane protein
CLO1313_RS11405	Clo1313_2244	1.216	0.282	0.001065268	0.002295072	hypothetical protein
CLO1313_RS11410	Clo1313_2245	1.114	0.156	0.076327621	0.116848665	hypothetical protein
CLO1313_RS11415	Clo1313_2246	1.016	0.023	0.82028021	0.857147737	transcriptional regulator
CLO1313_RS11420	Clo1313_2247	0.978	-0.032	0.77746935	0.824480404	FprA family A-type flavoprotein
CLO1313_RS11425	Clo1313_2248	1.077	0.107	0.298866966	0.381405119	NUDIX hydrolase
CLO1313_RS11430	Clo1313_2249	1.15	0.202	0.047953322	0.077443195	membrane protein
CLO1313_RS11435	Clo1313_2250	1.557	0.639	0.000860632	0.00186491	chemotaxis protein
CLO1313_RS11440	Clo1313_2251	1.093	0.128	0.597887759	0.671560071	hypothetical protein
CLO1313_RS11445	Clo1313_2252	0.791	-0.337	0.012958748	0.023610745	hypothetical protein
CLO1313_RS11450	Clo1313_2253	0.674	-0.568	0.000390855	0.000890711	PadR family transcriptional regulator
CLO1313_RS11455	Clo1313_2254	0.907	-0.142	0.055166675	0.088002585	NAD(P)H dehydrogenase

CLO1313_RS11460	Clo1313_2255	0.889	-0.169	0.079087747	0.120517049	PadR family transcriptional regulator
CLO1313_RS11465	Clo1313_2256	1.028	0.04	0.676498261	0.739904554	membrane protein
CLO1313_RS11470	Clo1313_2257	1.195	0.257	0.000280711	0.00065616	pyridoxamine 5'-phosphate oxidase-related FMN-binding
CLO1313_RS11475	Clo1313_2258	1.805	0.852	0.078960821	0.120446568	hypothetical protein
CLO1313_RS11480	Clo1313_2259	13.103	3.712	2.44E-55	9.03E-54	chemotaxis protein
CLO1313_RS11485	Clo1313_2260	4.406	2.14	8.27E-35	1.36E-33	adenylate cyclase
CLO1313_RS11490	Clo1313_2261	0.796	-0.329	0.000493295	0.001101558	multidrug ABC transporter ATP-binding protein
CLO1313_RS11495	Clo1313_2262	0.768	-0.38	0.000104042	0.00025702	ABC transporter
CLO1313_RS11500	Clo1313_2263	0.636	-0.653	6.64E-06	1.91E-05	MarR family transcriptional regulator
CLO1313_RS11505	Clo1313_2264	0.744	-0.427	3.53E-09	1.40E-08	hypothetical protein
CLO1313_RS11510	Clo1313_2265	0.738	-0.438	2.11E-10	9.51E-10	bacteriocin
CLO1313_RS11515	Clo1313_2266	0.923	-0.115	0.30369102	0.386246551	hypothetical protein
CLO1313_RS11520	Clo1313_2267	0.807	-0.31	0.011501106	0.021186619	phosphohydrolase
CLO1313_RS11525	Clo1313_2269	2.257	1.174	0.00053886	0.001197955	hypothetical protein
CLO1313_RS11530	Clo1313_2270	1.371	0.455	5.79E-07	1.88E-06	ArsC family transcriptional regulator
CLO1313_RS11535	Clo1313_2271	0.946	-0.081	0.460593189	0.543388626	hypothetical protein
CLO1313_RS11540	Clo1313_2272	0.999	-0.001	0.989724088	0.992039618	short-chain dehydrogenase
CLO1313_RS11545	Clo1313_2273	1.046	0.065	0.406999029	0.492173423	DNA methylase N-4
CLO1313_RS11550	Clo1313_2274	1.039	0.055	0.473173995	0.554749339	type II restriction endonuclease
CLO1313_RS11555	Clo1313_2275	1.32	0.401	1.45E-07	4.93E-07	restriction endonuclease subunit M
CLO1313_RS11560	Clo1313_2276	1.343	0.425	1.52E-06	4.69E-06	membrane protein
CLO1313_RS11565		1.069	0.097	0.476233317	0.55746437	GCN5 family acetyltransferase
CLO1313_RS11575	Clo1313_2280	1.197	0.259	0.031584979	0.053275225	phosphodiesterase
CLO1313_RS11585	Clo1313_2282	0.682	-0.553	3.19E-06	9.52E-06	hypothetical protein
CLO1313_RS11590	Clo1313_2283	0.886	-0.174	0.056194601	0.089404567	SAM-dependent methyltransferase
CLO1313_RS11595	Clo1313_2284	0.777	-0.364	0.001544904	0.003251346	hypothetical protein
CLO1313_RS11600	Clo1313_2285	0.752	-0.412	0.003968031	0.007922854	hypothetical protein
CLO1313_RS11605		0.724	-0.466	0.0114605	0.021125898	hypothetical protein
CLO1313_RS11610	Clo1313_2286	0.728	-0.459	1.02E-06	3.21E-06	copper amine oxidase
CLO1313_RS11615	Clo1313_2287	0.688	-0.539	9.97E-05	0.000247375	hypothetical protein

CLO1313_RS11620	Clo1313_2288	0.951	-0.073	0.547190829	0.62614416	FMN reductase
CLO1313_RS11625	Clo1313_2289	0.996	-0.006	0.932672374	0.946559882	glycosyl transferase family 1
CLO1313_RS11630	Clo1313_2290	10.885	3.444	2.90E-179	1.24E-176	hypothetical protein
CLO1313_RS11635	Clo1313_2291	1.074	0.103	0.430809203	0.515973163	hypothetical protein
CLO1313_RS11640	Clo1313_2292	1.079	0.109	0.592761746	0.668305442	bacitracin ABC transporter ATP-binding protein
CLO1313_RS11645	Clo1313_2293	1.479	0.565	8.29E-05	0.000208138	two-component system sensor histidine kinase
CLO1313_RS11650	Clo1313_2294	1.674	0.743	4.44E-07	1.45E-06	two-component system response regulator
CLO1313_RS11670		1.98	0.986	0.044769529	0.072732296	hypothetical protein
CLO1313_RS11675	Clo1313_2298	3.389	1.761	1.13E-15	7.55E-15	hypothetical protein
CLO1313_RS11680	Clo1313_2299	1.388	0.473	0.016931206	0.030206239	membrane protein
CLO1313_RS11685	Clo1313_2300	1.28	0.356	0.007796303	0.014858842	ABC transporter ATP-binding protein
CLO1313_RS11690	Clo1313_2301	1.057	0.08	0.68064991	0.743631723	hypothetical protein
CLO1313_RS11695	Clo1313_2302	1.196	0.259	0.033686618	0.05637621	DNA-binding protein
CLO1313_RS11700	Clo1313_2303	21.174	4.404	3.44E-125	4.92E-123	glutamine synthetase
CLO1313_RS11705	Clo1313_2304	1.358	0.442	0.009529924	0.017851494	aspartyl/glutamyl-tRNA amidotransferase subunit B
CLO1313_RS11710		1.296	0.375	0.007975575	0.015176872	glutamyl-tRNA(Gln) amidotransferase
CLO1313_RS11715	Clo1313_2306	1.267	0.342	0.027110081	0.046300189	aspartyl/glutamyl-tRNA amidotransferase subunit C
CLO1313_RS11720	Clo1313_2307	0.96	-0.06	0.667964777	0.732441084	aspartyl-tRNA synthetase
CLO1313_RS11725		1.566	0.647	3.30E-09	1.32E-08	hypothetical protein
CLO1313_RS11735	Clo1313_2310	1.197	0.26	0.000299922	0.00069672	4Fe-4S ferredoxin
CLO1313_RS11740	Clo1313_2311	1.016	0.023	0.911100768	0.930334083	membrane protein
CLO1313_RS11745	Clo1313_2312	0.863	-0.213	0.146926548	0.20523182	enoyl-CoA hydratase
CLO1313_RS11750	Clo1313_2314	0.983	-0.024	0.818085093	0.855583858	type 12 methyltransferase
CLO1313_RS11765	Clo1313_2317	1.153	0.206	0.068630196	0.106477992	MAEBL
CLO1313_RS11770	Clo1313_2318	0.919	-0.122	0.190010501	0.258075355	flavin reductase
CLO1313_RS11775	Clo1313_2319	0.658	-0.605	2.06E-17	1.49E-16	methylated-DNA--protein-cysteine methyltransferase
CLO1313_RS11780	Clo1313_2321	0.634	-0.656	7.05E-22	6.50E-21	metal ABC transporter substrate-binding protein
CLO1313_RS11785	Clo1313_2322	0.642	-0.64	1.21E-16	8.43E-16	methionine ABC transporter permease
CLO1313_RS11790	Clo1313_2323	0.56	-0.837	6.19E-28	7.70E-27	ABC transporter
CLO1313_RS11795	Clo1313_2324	0.816	-0.294	0.00019655	0.000467449	Rrf2 family transcriptional regulator

CLO1313_RS11800	Clo1313_2325	0.165	-2.595	8.51E-37	1.56E-35	cystathionine beta-lyase
CLO1313_RS11805	Clo1313_2326	0.181	-2.463	2.59E-13	1.45E-12	cysteine synthase
CLO1313_RS11810	Clo1313_2327	0.166	-2.591	2.26E-40	4.87E-39	hypothetical protein
CLO1313_RS11815	Clo1313_2328	0.217	-2.206	1.81E-32	2.63E-31	hypothetical protein
CLO1313_RS11820	Clo1313_2329	0.18	-2.471	3.90E-36	6.92E-35	ABC transporter ATP-binding protein
CLO1313_RS11825	Clo1313_2330	0.145	-2.788	8.28E-14	4.86E-13	GCN5 family N-acetyltransferase
CLO1313_RS11830	Clo1313_2331	0.131	-2.937	1.50E-20	1.28E-19	oxidoreductase/nitrogenase component 1
CLO1313_RS11835	Clo1313_2332	0.127	-2.974	6.45E-19	5.11E-18	hydrogenase
CLO1313_RS11840	Clo1313_2333	0.105	-3.254	2.44E-44	5.80E-43	diguanylate cyclase
CLO1313_RS11845	Clo1313_2334	0.123	-3.027	1.90E-47	5.22E-46	Radical SAM domain protein
CLO1313_RS11850	Clo1313_2335	0.122	-3.034	1.71E-22	1.61E-21	O-acetylhomoserine aminocarboxypropyltransferase
CLO1313_RS11855	Clo1313_2336	0.125	-2.996	2.11E-21	1.90E-20	nitrate ABC transporter substrate-binding protein
CLO1313_RS11860	Clo1313_2337	0.187	-2.42	8.75E-34	1.37E-32	ABC transporter permease
CLO1313_RS11865	Clo1313_2338	0.183	-2.446	1.30E-13	7.45E-13	ABC transporter ATP-binding protein
CLO1313_RS11875	Clo1313_2340	1.116	0.158	0.096242267	0.14232276	hypothetical protein
CLO1313_RS11885	Clo1313_2342	2.003	1.002	8.05E-20	6.69E-19	membrane protein
CLO1313_RS11890	Clo1313_2343	2.166	1.115	1.33E-21	1.22E-20	hypothetical protein
CLO1313_RS11900	Clo1313_2345	2.972	1.572	1.52E-24	1.57E-23	heme ABC transporter ATP-binding protein
CLO1313_RS11905	Clo1313_2346	2.843	1.508	5.77E-15	3.69E-14	ABC transporter permease
CLO1313_RS11910	Clo1313_2347	2.707	1.436	2.78E-13	1.56E-12	branched-chain amino acid ABC transporter permease
CLO1313_RS11915	Clo1313_2348	1.883	0.913	0.006637241	0.012776049	cytidine deaminase
CLO1313_RS11925	Clo1313_2350	1.477	0.563	0.000559106	0.001239289	two-component system response regulator
CLO1313_RS11930	Clo1313_2351	1.323	0.404	0.067240495	0.104642237	sensor histidine kinase
CLO1313_RS11935	Clo1313_2352	2.617	1.388	3.20E-22	2.99E-21	iron ABC transporter permease
CLO1313_RS11940	Clo1313_2353	2.858	1.515	3.80E-21	3.36E-20	peptide ABC transporter substrate-binding protein
CLO1313_RS11945	Clo1313_2354	3.368	1.752	4.55E-24	4.63E-23	iron ABC transporter substrate-binding protein
CLO1313_RS11950	Clo1313_2355	0.792	-0.336	0.010987362	0.020340183	transcriptional regulator
CLO1313_RS11955	Clo1313_2356	0.707	-0.5	1.92E-11	9.32E-11	peptidase M56
CLO1313_RS11960	Clo1313_2358	0.948	-0.077	0.64278681	0.711334924	hypothetical protein
CLO1313_RS11965	Clo1313_2359	1.043	0.06	0.495733823	0.574804401	glycerate kinase

CLO1313_RS11970	Clo1313_2361	1.309	0.388	0.014585923	0.026256413	hypothetical protein
CLO1313_RS11975		1.606	0.683	1.51E-05	4.14E-05	hypothetical protein
CLO1313_RS11980		1.272	0.347	0.007012186	0.013454604	hypothetical protein
CLO1313_RS11985	Clo1313_2365	1.184	0.244	0.312066378	0.395389551	alkaline phosphatase
CLO1313_RS11990	Clo1313_2366	1.389	0.474	0.013644206	0.024709525	helicase
CLO1313_RS11995	Clo1313_2367	2.28	1.189	1.06E-07	3.65E-07	DNA methylase
CLO1313_RS12000		1.682	0.75	0.000112645	0.00027713	hypothetical protein
CLO1313_RS12005	Clo1313_2369	1.192	0.254	0.002681153	0.005499847	transposase
CLO1313_RS12010	Clo1313_2370	0.848	-0.239	0.043588123	0.071276326	hypothetical protein
CLO1313_RS12015	Clo1313_2371	0.772	-0.373	0.002176697	0.004489625	iron-sulfur cluster loop
CLO1313_RS12020	Clo1313_2372	0.722	-0.47	0.001149586	0.002462578	hypothetical protein
CLO1313_RS12025	Clo1313_2373	0.651	-0.619	2.14E-06	6.50E-06	restriction endonuclease
CLO1313_RS12030	Clo1313_2374	0.7	-0.515	0.001717264	0.00360145	NUDIX hydrolase
CLO1313_RS12035	Clo1313_2375	0.605	-0.725	6.06E-15	3.87E-14	integrase
CLO1313_RS12040	Clo1313_2376	0.986	-0.021	0.950281669	0.960278895	serine recombinase
CLO1313_RS12045	Clo1313_2377	1.976	0.983	0.563158653	0.639739697	recombinase
CLO1313_RS12050	Clo1313_2378	1.356	0.439	0.612491523	0.685396297	recombinase
CLO1313_RS12055	Clo1313_2379	0.554	-0.853	0.681391916	0.744171288	hypothetical protein
CLO1313_RS12060	Clo1313_2380	1.844	0.883	0.491265819	0.571713695	peptidoglycan-binding protein LysM
CLO1313_RS12065	Clo1313_2381	4.634	2.212	0.266024711	0.347628805	hypothetical protein
CLO1313_RS12070	Clo1313_2382	2.465	1.302	2.07E-13	1.17E-12	glycosyl hydrolase
CLO1313_RS12075	Clo1313_2383	2.199	1.137	0.073242762	0.112551585	hypothetical protein
CLO1313_RS12080	Clo1313_2384	1.068	0.095	0.928213609	0.94266109	hypothetical protein
CLO1313_RS12085	Clo1313_2385	1.408	0.494	0.126157752	0.180337034	hypothetical protein
CLO1313_RS12090	Clo1313_2386	0.891	-0.167	0.76890746	0.816050538	tail protein
CLO1313_RS12095	Clo1313_2387	1.739	0.798	0.141492107	0.198380005	hypothetical protein
CLO1313_RS12100		NA	NA	NA	NA	
CLO1313_RS12105	Clo1313_2389	16.735	4.065	0.015051272	0.026996869	hypothetical protein
CLO1313_RS12110	Clo1313_2390	2.744	1.456	0.154283628	0.214409916	phage tail protein
CLO1313_RS12115	Clo1313_2391	1.044	0.063	0.944212411	0.955476087	hypothetical protein

CLO1313_RS12120	Clo1313_2392	0.784	-0.351	0.706848802	0.763355981	hypothetical protein
CLO1313_RS12125	Clo1313_2393	0.895	-0.16	0.886913869	0.909974237	phage head-tail adapter protein
CLO1313_RS12130		0.486	-1.042	0.798363264	0.840130629	
CLO1313_RS12135	Clo1313_2395	1.504	0.589	0.55252615	0.630287533	phage capsid protein
CLO1313_RS12140	Clo1313_2396	3.348	1.744	0.398073628	0.484309457	peptidase
CLO1313_RS12145	Clo1313_2397	0.412	-1.278	0.405599792	0.491273739	portal protein
CLO1313_RS12150	Clo1313_2398	2.024	1.017	0.018540872	0.032843517	terminase
CLO1313_RS12155	Clo1313_2399	3.381	1.758	0.047503372	0.076882144	virulence protein
CLO1313_RS12160	Clo1313_2400	2.683	1.424	0.021723662	0.037833485	gamma-glutamyl cyclotransferase
CLO1313_RS12165	Clo1313_2401	1.87	0.903	0.161014706	0.223143764	hypothetical protein
CLO1313_RS12170	Clo1313_2402	3.348	1.744	0.398073628	0.484309457	methyltransferase
CLO1313_RS12175	Clo1313_2403	0.276	-1.858	0.595114944	0.669699706	hypothetical protein
CLO1313_RS12180	Clo1313_2404	1.281	0.357	0.686163065	0.74693395	virulence factor
CLO1313_RS12185	Clo1313_2405	1.468	0.554	0.471463504	0.553176467	chromosome partitioning protein ParB
CLO1313_RS12190	Clo1313_2406	1.512	0.597	0.225820838	0.2993973	DNA methylase N-4
CLO1313_RS12195	Clo1313_2407	1.686	0.754	0.552455172	0.630287533	terminase
CLO1313_RS12200	Clo1313_2408	3.014	1.592	0.374016736	0.460080473	HNH endonuclease
CLO1313_RS12205	Clo1313_2409	1.475	0.56	0.519326482	0.598332739	hypothetical protein
CLO1313_RS12210	Clo1313_2410	1.224	0.292	0.270279203	0.351351248	hypothetical protein
CLO1313_RS12215	Clo1313_2411	1.087	0.12	0.736723025	0.789280017	VRR-NUC domain protein
CLO1313_RS12220	Clo1313_2412	1.114	0.155	0.176890196	0.242455986	DNA-binding protein
CLO1313_RS12225	Clo1313_2414	0.712	-0.491	0.001705112	0.003578469	peptide ABC transporter ATP-binding protein
CLO1313_RS12230	Clo1313_2415	0.888	-0.172	0.104080842	0.152188419	hypothetical protein
CLO1313_RS12235	Clo1313_2416	1.953	0.966	2.83E-21	2.51E-20	histidine kinase
CLO1313_RS12240	Clo1313_2417	1.525	0.608	2.88E-08	1.04E-07	two-component system response regulator
CLO1313_RS12245	Clo1313_2418	1.69	0.757	4.69E-15	3.03E-14	MBL fold metallo-hydrolase
CLO1313_RS12250		1.704	0.769	0.002325272	0.004786199	homoserine kinase
CLO1313_RS12255		1.356	0.44	0.296226471	0.379326724	aminoglycoside phosphotransferase
CLO1313_RS12260		0.755	-0.405	0.108170209	0.157383782	hypothetical protein
CLO1313_RS12265	Clo1313_2421	0.747	-0.422	0.001487818	0.003137811	hypothetical protein

CLO1313_RS12270	Clo1313_2422	0.636	-0.653	6.63E-06	1.91E-05	hypothetical protein
CLO1313_RS12280	Clo1313_2425	0.721	-0.472	3.82E-05	9.94E-05	RNA methyltransferase
CLO1313_RS12285	Clo1313_2426	1.115	0.157	0.325038468	0.409231892	ABC transporter
CLO1313_RS12290	Clo1313_2427	1.19	0.25	0.148185385	0.206893841	corrinoid ABC transporter permease
CLO1313_RS12295	Clo1313_2428	0.815	-0.296	0.095611792	0.141599883	ABC transporter substrate-binding protein
CLO1313_RS12300	Clo1313_2429	0.979	-0.031	0.706550754	0.763308974	virion core protein (lumpy skin disease virus)
CLO1313_RS12305	Clo1313_2430	0.842	-0.248	0.009196501	0.017280894	peptidase M23
CLO1313_RS12310	Clo1313_2431	0.777	-0.363	0.000475507	0.001068199	hypothetical protein
CLO1313_RS12320	Clo1313_2433	1.061	0.086	0.222444529	0.295836427	penicillin-binding protein 1A
CLO1313_RS12325	Clo1313_2434	1.263	0.337	0.000114847	0.000282085	hypothetical protein
CLO1313_RS12330	Clo1313_2435	1.194	0.256	0.00470526	0.009308096	RND transporter MFP subunit
CLO1313_RS12335	Clo1313_2436	1.095	0.131	0.133014017	0.188787997	macrolide ABC transporter ATP-binding protein
CLO1313_RS12340	Clo1313_2437	1.164	0.219	0.00078345	0.00170382	hypothetical protein
CLO1313_RS12345	Clo1313_2438	1.09	0.124	0.052376762	0.084043825	hypothetical protein
CLO1313_RS12355	Clo1313_2440	0.582	-0.78	4.51E-29	5.94E-28	hypothetical protein
CLO1313_RS12360	Clo1313_2441	0.545	-0.876	1.05E-35	1.83E-34	nitrogen-fixing protein NifU
CLO1313_RS12365	Clo1313_2442	1.406	0.492	6.52E-06	1.88E-05	hypothetical protein
CLO1313_RS12370	Clo1313_2443	1.546	0.629	1.50E-11	7.35E-11	hypothetical protein
CLO1313_RS12375	Clo1313_2444	1.292	0.37	1.85E-06	5.65E-06	rubrerythrin
CLO1313_RS12380	Clo1313_R0055	0.761	-0.395	0.000375088	0.00085804	
CLO1313_RS12385	Clo1313_2445	0.898	-0.156	0.226461046	0.300113423	hypothetical protein
CLO1313_RS12390	Clo1313_2446	0.842	-0.249	0.000993351	0.002143208	ATP-dependent protease
CLO1313_RS12405	Clo1313_2449	0.78	-0.359	0.000470946	0.001058746	tRNA threonylcarbamoyladenosine biosynthesis protein TsaE
CLO1313_RS12410	Clo1313_2450	0.788	-0.344	1.35E-06	4.21E-06	amidohydrolase
CLO1313_RS12415	Clo1313_2451	0.495	-1.016	4.43E-27	5.23E-26	copper amine oxidase
CLO1313_RS12420	Clo1313_2452	1.084	0.116	0.359412894	0.44579077	hypothetical protein
CLO1313_RS12425	Clo1313_2453	0.839	-0.252	0.056034317	0.089244246	hypothetical protein
CLO1313_RS12430	Clo1313_2454	0.904	-0.146	0.167544993	0.231338597	hypothetical protein
CLO1313_RS12435	Clo1313_2455	0.623	-0.682	5.59E-28	7.02E-27	30S ribosomal protein S9

CLO1313_RS12440	Clo1313_2456	0.543	-0.88	6.88E-42	1.55E-40	50S ribosomal protein L13
CLO1313_RS12445	Clo1313_2457	1.626	0.701	4.91E-14	2.94E-13	hypothetical protein
CLO1313_RS12450	Clo1313_2458	1.83	0.872	1.37E-21	1.25E-20	DUF147 domain-containing protein
CLO1313_RS12455	Clo1313_2459	1.458	0.544	2.56E-08	9.32E-08	DNA repair protein RadA
CLO1313_RS12460	Clo1313_2460	1.287	0.364	2.49E-05	6.61E-05	glycoside hydrolase
CLO1313_RS12465	Clo1313_2461	1.154	0.206	0.276049732	0.357767133	glycosyl transferase family 2
CLO1313_RS12470	Clo1313_2462	1.186	0.246	0.022840435	0.039617389	ATP-dependent Clp protease ATP-binding protein
CLO1313_RS12475	Clo1313_2463	1.291	0.369	1.35E-05	3.71E-05	ATP--guanido phosphotransferase
CLO1313_RS12480	Clo1313_2464	1.43	0.516	1.24E-12	6.57E-12	hypothetical protein
CLO1313_RS12485	Clo1313_2465	1.192	0.253	0.003673817	0.007364823	hypothetical protein
CLO1313_RS12510	Clo1313_2469	1.047	0.066	0.421962114	0.506694804	prephenate dehydrogenase
CLO1313_RS12520	Clo1313_2471	1.02	0.028	0.736906985	0.789280017	REX family transcriptional regulator
CLO1313_RS12525	Clo1313_2472	0.829	-0.27	0.025770736	0.044214209	ABC transporter
CLO1313_RS12530	Clo1313_2473	1.168	0.224	0.040022162	0.065984862	glycosyl hydrolase
CLO1313_RS12535	Clo1313_2474	1.174	0.231	0.02326221	0.040212913	cobalt ABC transporter ATP-binding protein
CLO1313_RS12545	Clo1313_2476	1.126	0.171	0.034532367	0.057630811	cobalamin biosynthesis protein CbiM
CLO1313_RS12555	Clo1313_2478	0.79	-0.34	0.000329942	0.000760566	two-component system diguanylate cyclase response regulator
CLO1313_RS12560	Clo1313_2479	1.999	0.999	1.03E-12	5.49E-12	dockerin
CLO1313_RS12570	Clo1313_2481	1.852	0.889	1.48E-17	1.08E-16	hypothetical protein
CLO1313_RS12575	Clo1313_2482	2.605	1.382	5.76E-24	5.82E-23	hypothetical protein
CLO1313_RS12580	Clo1313_2483	4.437	2.15	2.66E-27	3.17E-26	RNA polymerase subunit sigma-24
CLO1313_RS12585	Clo1313_2484	1.027	0.039	0.784516563	0.829022259	peptidase M56
CLO1313_RS12590	Clo1313_2485	1.267	0.342	0.065919961	0.102698163	transcriptional regulator
CLO1313_RS12595	Clo1313_2486	1.935	0.953	0.009278868	0.017413845	urease accessory protein UreD
CLO1313_RS12605	Clo1313_2488	1.869	0.902	0.018251148	0.032387688	urease accessory protein UreF
CLO1313_RS12655	Clo1313_2498	3.102	1.633	9.65E-15	6.04E-14	two-component system response regulator
CLO1313_RS12660	Clo1313_2499	2.742	1.455	4.18E-17	2.97E-16	sensor histidine kinase
CLO1313_RS12665	Clo1313_2500	1.335	0.417	1.85E-05	5.00E-05	two-component system sensor histidine kinase/response regulator

CLO1313_RS12670	Clo1313_2502	0.94	-0.089	0.690333482	0.750112359	copper amine oxidase
CLO1313_RS12675	Clo1313_2503	0.996	-0.006	0.987949355	0.990591813	hypothetical protein
CLO1313_RS12680	Clo1313_2504	0.948	-0.077	0.849434297	0.879645531	hypothetical protein
CLO1313_RS12685		0.661	-0.597	0.121373522	0.174496257	ATPase
CLO1313_RS12690		0.487	-1.039	3.54E-05	9.24E-05	hypothetical protein
CLO1313_RS12695	Clo1313_2506	0.912	-0.133	0.29477675	0.378116113	hypothetical protein
CLO1313_RS12700	Clo1313_2507	0.48	-1.058	6.47E-31	8.82E-30	hypothetical protein
CLO1313_RS12705	Clo1313_2508	1.565	0.646	6.04E-09	2.34E-08	transposase
CLO1313_RS12715	Clo1313_2510	0.751	-0.413	0.000576986	0.001273276	copper amine oxidase
CLO1313_RS12720	Clo1313_2511	0.802	-0.318	0.069842532	0.108079336	hypothetical protein
CLO1313_RS12725		0.752	-0.412	0.10554529	0.154029356	hypothetical protein
CLO1313_RS12740	Clo1313_2515	1.17	0.227	0.14958682	0.208666288	hypothetical protein
CLO1313_RS12745	Clo1313_2516	0.466	-1.102	1.25E-32	1.86E-31	hypothetical protein
CLO1313_RS12755	Clo1313_2518	0.527	-0.924	6.34E-29	8.31E-28	hypothetical protein
CLO1313_RS12760	Clo1313_2519	0.56	-0.837	8.42E-19	6.59E-18	copper amine oxidase
CLO1313_RS12765	Clo1313_2520	0.589	-0.764	6.01E-17	4.22E-16	hypothetical protein
CLO1313_RS12770		0.395	-1.34	1.38E-17	1.01E-16	hypothetical protein
CLO1313_RS12775	Clo1313_2522	1.134	0.181	0.36069378	0.447176786	transposase
CLO1313_RS12780	Clo1313_2523	0.998	-0.003	0.978889423	0.983560155	hypothetical protein
CLO1313_RS12785	Clo1313_2524	0.885	-0.177	0.142756986	0.199966464	hypothetical protein
CLO1313_RS12790	Clo1313_2525	0.899	-0.153	0.238581455	0.314646343	copper amine oxidase
CLO1313_RS12795	Clo1313_2526	0.882	-0.182	0.129207813	0.183995361	hypothetical protein
CLO1313_RS12800		0.646	-0.63	0.000717274	0.001570531	hypothetical protein
CLO1313_RS12805		0.673	-0.571	0.000102149	0.000252759	hypothetical protein
CLO1313_RS12815	Clo1313_2528	1.414	0.5	0.063721784	0.099583966	hypothetical protein
CLO1313_RS12820	Clo1313_2529	1.114	0.156	0.0370129	0.061477921	hypothetical protein
CLO1313_RS12825	Clo1313_2530	0.893	-0.163	0.026479438	0.045352276	glycoside hydrolase
CLO1313_RS12840	Clo1313_2533	0.494	-1.019	1.49E-21	1.36E-20	potassium-transporting ATPase subunit A
CLO1313_RS12845	Clo1313_2534	0.52	-0.943	8.04E-19	6.31E-18	O-acetylhomoserine aminocarboxypropyltransferase
CLO1313_RS12850	Clo1313_2535	0.716	-0.482	1.71E-06	5.26E-06	Rrf2 family transcriptional regulator

CLO1313_RS12855	Clo1313_2536	0.95	-0.075	0.370156131	0.45683055	AsnC family transcriptional regulator
CLO1313_RS12860	Clo1313_2537	1.084	0.116	0.165180831	0.22849507	homoserine O-succinyltransferase
CLO1313_RS12865	Clo1313_2538	1.315	0.395	9.00E-05	0.000225423	two-component system sensor histidine kinase
CLO1313_RS12870	Clo1313_2539	1.147	0.198	0.134389925	0.190380436	two-component system response regulator
CLO1313_RS12875	Clo1313_2540	1.145	0.195	0.179987953	0.246139476	ATPase P
CLO1313_RS12880	Clo1313_2541	1.277	0.353	0.00037602	0.000859515	heavy metal transporter
CLO1313_RS12885	Clo1313_2542	0.945	-0.082	0.736646888	0.789280017	hypothetical protein
CLO1313_RS12890	Clo1313_2543	1.792	0.842	1.87E-17	1.36E-16	hypothetical protein
CLO1313_RS12895	Clo1313_R0056	0.217	-2.206	3.36E-26	3.77E-25	
CLO1313_RS12900	Clo1313_R0057	0.337	-1.569	3.91E-18	2.95E-17	
CLO1313_RS12905	Clo1313_R0058	0.386	-1.375	2.28E-18	1.74E-17	
CLO1313_RS12910	Clo1313_R0059	0.452	-1.145	1.37E-18	1.05E-17	
CLO1313_RS12915	Clo1313_R0060	0.513	-0.963	3.12E-14	1.89E-13	
CLO1313_RS12920	Clo1313_2544	0.633	-0.659	4.81E-14	2.88E-13	molecular chaperone Hsp33
CLO1313_RS12925	Clo1313_2545	0.613	-0.707	5.51E-14	3.27E-13	cold-shock protein
CLO1313_RS12930	Clo1313_2546	0.713	-0.488	3.53E-05	9.23E-05	methylated-DNA--protein-cysteine methyltransferase
CLO1313_RS12935	Clo1313_2547	0.724	-0.466	6.33E-06	1.83E-05	type 11 methyltransferase
CLO1313_RS12940	Clo1313_2548	0.888	-0.172	0.082700577	0.124946614	spore protein
CLO1313_RS12945	Clo1313_2549	0.978	-0.032	0.674993874	0.73879804	peptidase S41
CLO1313_RS12950	Clo1313_2550	0.851	-0.232	0.00132757	0.002823676	peptidase M23
CLO1313_RS12955	Clo1313_2551	0.815	-0.295	0.000137422	0.000333437	cell division protein FtsX
CLO1313_RS12965	Clo1313_2553	1.112	0.153	0.053586679	0.085801628	CdaR family transcriptional regulator
CLO1313_RS12970	Clo1313_2554	0.528	-0.921	5.00E-48	1.44E-46	sugar ABC transporter ATP-binding protein
CLO1313_RS12975	Clo1313_2555	0.304	-1.72	1.60E-26	1.84E-25	N-acetyl-gamma-glutamyl-phosphate reductase
CLO1313_RS12985	Clo1313_2557	0.559	-0.839	2.24E-14	1.37E-13	pilus assembly protein PilZ
CLO1313_RS12990	Clo1313_2558	0.339	-1.561	2.31E-34	3.70E-33	acetylornithine aminotransferase
CLO1313_RS12995	Clo1313_2559	0.29	-1.785	7.04E-43	1.62E-41	carbamoyl phosphate synthase small subunit
CLO1313_RS13000	Clo1313_2560	0.267	-1.903	2.24E-53	7.56E-52	carbamoyl phosphate synthase large subunit
CLO1313_RS13010	Clo1313_2562	0.323	-1.631	5.92E-35	9.81E-34	GCN5 family N-acetyltransferase
CLO1313_RS13015	Clo1313_2563	0.359	-1.478	4.29E-19	3.43E-18	transposase

CLO1313_RS13020	Clo1313_2564	2.379	1.25	1.26E-17	9.32E-17	dockerin
CLO1313_RS13025	Clo1313_2565	2.464	1.301	1.78E-17	1.30E-16	hypothetical protein
CLO1313_RS13030		1.479	0.564	1.75E-06	5.38E-06	hypothetical protein
CLO1313_RS13035	Clo1313_2567	1.513	0.597	8.69E-12	4.36E-11	cell division protein
CLO1313_RS13040	Clo1313_2568	1.394	0.479	4.57E-09	1.79E-08	PadR family transcriptional regulator
CLO1313_RS13045	Clo1313_2569	0.773	-0.372	0.000338812	0.00077865	metallophosphoesterase
CLO1313_RS13050	Clo1313_2570	0.569	-0.813	1.08E-05	3.01E-05	hypothetical protein
CLO1313_RS13055		0.669	-0.579	2.17E-05	5.80E-05	hypothetical protein
CLO1313_RS13060	Clo1313_2572	0.624	-0.68	1.83E-15	1.21E-14	D-Ala-D-Ala carboxypeptidase VanY
CLO1313_RS13070	Clo1313_2574	1.582	0.662	2.99E-10	1.32E-09	transposase
CLO1313_RS13075	Clo1313_2575	1.827	0.87	1.12E-09	4.68E-09	pyridoxal-5'-phosphate-dependent protein subunit beta
CLO1313_RS13080	Clo1313_2576	0.951	-0.073	0.854424055	0.883910522	polysaccharide deacetylase
CLO1313_RS13085	Clo1313_2577	0.952	-0.071	0.70871019	0.764540237	non-ribosomal peptide synthetase
CLO1313_RS13090	Clo1313_2578	0.903	-0.147	0.55443455	0.63173	glycosyl transferase
CLO1313_RS13095	Clo1313_2579	0.804	-0.315	0.119185448	0.171844787	glycosyl transferase
CLO1313_RS13100	Clo1313_2580	1.073	0.102	0.553520625	0.631181884	amino acid adenylation protein
CLO1313_RS13105	Clo1313_2581	0.692	-0.532	1.65E-06	5.07E-06	copper amine oxidase
CLO1313_RS13110	Clo1313_2582	0.82	-0.286	0.001233871	0.002633721	copper amine oxidase
CLO1313_RS13115	Clo1313_2583	0.981	-0.028	0.751155961	0.801108367	hypothetical protein
CLO1313_RS13120	Clo1313_2584	0.601	-0.734	2.13E-31	2.98E-30	carbohydrate-binding protein
CLO1313_RS13125	Clo1313_2585	0.906	-0.142	0.183430016	0.249934856	transposase
CLO1313_RS13130	Clo1313_2586	0.631	-0.664	2.30E-24	2.36E-23	copper amine oxidase
CLO1313_RS13135	Clo1313_2587	0.588	-0.766	1.40E-14	8.65E-14	membrane protein
CLO1313_RS13140	Clo1313_2588	0.771	-0.375	0.00014673	0.000355161	hypothetical protein
CLO1313_RS13145	Clo1313_2589	1.109	0.149	0.106868489	0.155733041	two-component system sensor histidine kinase
CLO1313_RS13150	Clo1313_2590	1.057	0.081	0.510219979	0.588519122	two-component system response regulator
CLO1313_RS13155	Clo1313_2591	0.726	-0.463	1.15E-07	3.94E-07	ATPase
CLO1313_RS13160	Clo1313_2592	0.559	-0.838	9.38E-34	1.46E-32	arginine decarboxylase
CLO1313_RS13165	Clo1313_2593	1.327	0.408	9.30E-06	2.62E-05	methyltransferase
CLO1313_RS13175	Clo1313_2595	0.829	-0.271	0.124499601	0.178306736	PadR family transcriptional regulator

CLO1313_RS13180	Clo1313_2596	0.894	-0.162	0.071095639	0.109791875	hypothetical protein
CLO1313_RS13185	Clo1313_2597	0.76	-0.396	1.61E-05	4.39E-05	CTP synthase
CLO1313_RS13200	Clo1313_2599	1.032	0.046	0.826858055	0.860503822	hypothetical protein
CLO1313_RS13205	Clo1313_2600	1.072	0.101	0.602330709	0.67579117	
CLO1313_RS13210	Clo1313_2601	1.314	0.394	4.38E-09	1.72E-08	peptidase S41
CLO1313_RS13215	Clo1313_2602	1.346	0.428	3.70E-09	1.46E-08	hypothetical protein
CLO1313_RS13220	Clo1313_2603	5.557	2.474	8.06E-59	3.18E-57	hypothetical protein
CLO1313_RS13225	Clo1313_2604	0.565	-0.823	1.90E-20	1.61E-19	MBL fold metallo-hydrolase
CLO1313_RS13230	Clo1313_2605	1.044	0.062	0.556116903	0.63317942	abortive infection protein
CLO1313_RS13235	Clo1313_2606	0.961	-0.058	0.456241766	0.539751107	arginine--tRNA ligase
CLO1313_RS13240	Clo1313_2607	0.841	-0.25	0.00943405	0.017682947	hypothetical protein
CLO1313_RS13245	Clo1313_2608	0.831	-0.267	0.003474865	0.006998738	glutamate racemase
CLO1313_RS13250	Clo1313_2609	0.739	-0.437	7.68E-07	2.46E-06	D-alanine--D-alanine ligase
CLO1313_RS13260	Clo1313_2612	0.71	-0.495	1.31E-11	6.48E-11	hypothetical protein
CLO1313_RS13265	Clo1313_2613	0.921	-0.119	0.228705753	0.302286713	FmdB family transcriptional regulator
CLO1313_RS13270	Clo1313_2614	1.038	0.054	0.661907486	0.727661492	hypothetical protein
CLO1313_RS13280	Clo1313_2616	1.696	0.762	3.85E-14	2.32E-13	hypothetical protein
CLO1313_RS13285	Clo1313_2617	1.349	0.432	9.40E-09	3.58E-08	thioredoxin-disulfide reductase
CLO1313_RS13295	Clo1313_2619	1.173	0.23	0.08483714	0.127852554	redoxin
CLO1313_RS13300	Clo1313_2620	0.933	-0.101	0.554340754	0.63173	cytochrome C biogenesis protein ResB
CLO1313_RS13310	Clo1313_2621	0.966	-0.051	0.949927089	0.960278895	hypothetical protein
CLO1313_RS13315	Clo1313_2622	1.358	0.441	0.439918305	0.523953533	hypothetical protein
CLO1313_RS13320	Clo1313_2623	1.234	0.303	0.545847677	0.625046653	hypothetical protein
CLO1313_RS13330	Clo1313_2625	0.752	-0.412	1.53E-05	4.18E-05	hypothetical protein
CLO1313_RS13335	Clo1313_2626	0.775	-0.369	9.74E-06	2.73E-05	hypothetical protein
CLO1313_RS13340	Clo1313_2627	1.003	0.004	0.951795451	0.961412784	tagatose-6-phosphate kinase
CLO1313_RS13345	Clo1313_2628	1.49	0.575	1.79E-08	6.62E-08	sugar ABC transporter substrate-binding protein
CLO1313_RS13350	Clo1313_2629	1.004	0.006	0.983160434	0.987110191	hypothetical protein
CLO1313_RS13355	Clo1313_2630	0.962	-0.056	0.705432004	0.762924839	UV damage repair endonuclease
CLO1313_RS13360	Clo1313_2631	0.811	-0.301	0.018947619	0.033484921	type 11 methyltransferase

CLO1313_RS13365	Clo1313_2632	0.96	-0.058	0.406835174	0.492173423	peptidoglycan-binding protein
CLO1313_RS13370	Clo1313_2633	1.061	0.086	0.289330309	0.372244357	nucleotidyltransferase
CLO1313_RS13375	Clo1313_2634	1.378	0.462	5.92E-08	2.08E-07	pseudouridine synthase
CLO1313_RS13380	Clo1313_2635	1.326	0.407	0.001473795	0.003112614	endo-1,4-beta-xylanase Z
CLO1313_RS13385	Clo1313_2637	0.941	-0.088	0.419569589	0.504324328	NADH dehydrogenase
CLO1313_RS13395	Clo1313_2640	0.853	-0.23	0.167348595	0.231173854	hypothetical protein
CLO1313_RS13400	Clo1313_2641	0.809	-0.306	0.012123647	0.022183537	cell division protein FtsK
CLO1313_RS13405	Clo1313_2642	0.719	-0.476	6.42E-07	2.08E-06	cell division protein FtsK
CLO1313_RS13410	Clo1313_2643	0.774	-0.37	0.000324482	0.000749708	hypothetical protein
CLO1313_RS13415	Clo1313_2644	0.84	-0.251	0.018108101	0.032152869	hypothetical protein
CLO1313_RS13420	Clo1313_2645	0.8	-0.322	0.018837617	0.033349477	hypothetical protein
CLO1313_RS13425	Clo1313_2646	0.71	-0.495	0.000592713	0.0013051	hypothetical protein
CLO1313_RS13430	Clo1313_2647	0.727	-0.46	1.36E-06	4.24E-06	hypothetical protein
CLO1313_RS13435	Clo1313_2648	0.817	-0.292	0.033537628	0.056189578	hypothetical protein
CLO1313_RS13440	Clo1313_2649	2.023	1.017	5.06E-11	2.39E-10	hypothetical protein
CLO1313_RS13445	Clo1313_2650	1.829	0.871	1.35E-06	4.22E-06	hypothetical protein
CLO1313_RS13460	Clo1313_2654	1.474	0.559	1.09E-05	3.05E-05	hypothetical protein
CLO1313_RS13465	Clo1313_2655	2.212	1.146	1.62E-13	9.21E-13	hypothetical protein
CLO1313_RS13480	Clo1313_2657	1.148	0.2	0.262237489	0.343278144	hypothetical protein
CLO1313_RS13485	Clo1313_2658	1.045	0.064	0.750197013	0.800616422	hypothetical protein
CLO1313_RS13490	Clo1313_2659	1.011	0.016	0.955277131	0.964280079	transposase
CLO1313_RS13495	Clo1313_2660	0.679	-0.558	0.054424328	0.086957145	resolvase
CLO1313_RS13500	Clo1313_2661	0.5	-1	0.034514358	0.057630811	hypothetical protein
CLO1313_RS13505		0.306	-1.711	0.039517714	0.065189013	hypothetical protein
CLO1313_RS13515		1.269	0.343	1.87E-05	5.05E-05	integrase
CLO1313_RS13520	Clo1313_2664	1.272	0.347	7.98E-05	0.000200738	transposase
CLO1313_RS13525	Clo1313_2665	1.467	0.553	0.005694552	0.011089585	1,4-dihydroxy-6-naphthoate synthase
CLO1313_RS13530		1.407	0.493	8.84E-06	2.50E-05	
CLO1313_RS13535	Clo1313_2667	1.513	0.597	0.063277674	0.098993085	DNA primase
CLO1313_RS13540	Clo1313_2669	1.338	0.42	4.66E-05	0.000120308	hypothetical protein

CLO1313_RS13545	Clo1313_2670	3.379	1.757	9.43E-38	1.78E-36	hypothetical protein
CLO1313_RS13550	Clo1313_2671	2.002	1.001	1.76E-32	2.57E-31	Hedgehog/intein hint domain-containing protein
CLO1313_RS13555	Clo1313_2672	1.808	0.854	1.20E-06	3.75E-06	hypothetical protein
CLO1313_RS13565	Clo1313_2675	1.426	0.512	0.000505979	0.001129041	hypothetical protein
CLO1313_RS13570		0.994	-0.009	0.971214758	0.977079187	hypothetical protein
CLO1313_RS13575	Clo1313_2677	2.449	1.292	2.68E-26	3.02E-25	hypothetical protein
CLO1313_RS13580	Clo1313_2678	2.062	1.044	2.42E-21	2.18E-20	Hedgehog/intein hint domain-containing protein
CLO1313_RS13600	Clo1313_2682	1.73	0.791	3.17E-07	1.05E-06	hypothetical protein
CLO1313_RS13605	Clo1313_2683	1.902	0.928	3.28E-10	1.44E-09	hypothetical protein
CLO1313_RS13610	Clo1313_2684	1.792	0.841	2.01E-08	7.40E-08	hypothetical protein
CLO1313_RS13620	Clo1313_2686	0.852	-0.231	0.012676201	0.023124042	transposase
CLO1313_RS13625	Clo1313_2687	1.094	0.129	0.114354656	0.165436379	hypothetical protein
CLO1313_RS13630	Clo1313_2688	1.129	0.175	0.320726874	0.405334975	hypothetical protein
CLO1313_RS13635	Clo1313_2689	1.073	0.101	0.481608686	0.562275079	serine/threonine protein kinase
CLO1313_RS13640		0.897	-0.157	0.495273207	0.574733082	hypothetical protein
CLO1313_RS13645		0.748	-0.418	0.01960528	0.034403882	hypothetical protein
CLO1313_RS13650	Clo1313_2691	0.755	-0.406	0.005765618	0.011220694	amidohydrolase
CLO1313_RS13665	Clo1313_2693	1.225	0.293	0.000212447	0.00050366	dockerin
CLO1313_RS13680	Clo1313_2696	0.975	-0.037	0.934305079	0.947255217	hypothetical protein
CLO1313_RS13695	Clo1313_2698	1.232	0.301	0.437413781	0.522007135	hypothetical protein
CLO1313_RS13705	Clo1313_2700	1.233	0.302	0.000565394	0.001252302	transposase
CLO1313_RS13710		1.201	0.265	0.005619109	0.010964026	hypothetical protein
CLO1313_RS13715		1.109	0.15	0.240168812	0.316461453	hypothetical protein
CLO1313_RS13720	Clo1313_2702	1.04	0.057	0.699948149	0.758909797	hypothetical protein
CLO1313_RS13725	Clo1313_2703	1.098	0.135	0.589613982	0.665006518	hypothetical protein
CLO1313_RS13735	Clo1313_2705	0.882	-0.18	0.571984954	0.647314293	hypothetical protein
CLO1313_RS13740	Clo1313_2706	0.885	-0.175	0.358609718	0.445145092	hypothetical protein
CLO1313_RS13745	Clo1313_2707	0.89	-0.168	0.297366988	0.380498536	CRISPR-associated protein Csm3
CLO1313_RS13750	Clo1313_2708	0.697	-0.521	0.005251596	0.010293814	CRISPR-associated protein Csm3
CLO1313_RS13755	Clo1313_2709	0.713	-0.488	0.177406774	0.243052953	hypothetical protein

CLO1313_RS13760	Clo1313_2710	0.576	-0.796	0.000482429	0.001081319	hypothetical protein
CLO1313_RS13765	Clo1313_2711	0.426	-1.232	3.85E-07	1.27E-06	hypothetical protein
CLO1313_RS13770	Clo1313_2712	0.516	-0.954	6.75E-16	4.57E-15	hypothetical protein
CLO1313_RS13775	Clo1313_2713	0.287	-1.803	6.08E-25	6.45E-24	hypothetical protein
CLO1313_RS13780	Clo1313_2714	0.662	-0.596	0.000180454	0.000433291	CRISPR-associated protein
CLO1313_RS13785	Clo1313_2715	1.099	0.136	0.400712247	0.487020394	hypothetical protein
CLO1313_RS13795	Clo1313_R0061	0.497	-1.009	1.88E-14	1.15E-13	
CLO1313_RS13800	Clo1313_R0062	0.868	-0.205	0.215925881	0.288446199	
CLO1313_RS13805	Clo1313_2716	1.451	0.537	9.85E-10	4.14E-09	DNA-directed RNA polymerase sigma-70 factor
CLO1313_RS13810	Clo1313_2718	0.54	-0.89	8.00E-31	1.09E-29	RNA methyltransferase
CLO1313_RS13815	Clo1313_2719	0.667	-0.584	1.55E-13	8.85E-13	ribonuclease III
CLO1313_RS13820	Clo1313_2720	1.051	0.072	0.767851666	0.815434542	germination protein GerA
CLO1313_RS13825	Clo1313_2721	1.329	0.41	0.306922373	0.389695257	germination protein
CLO1313_RS13830	Clo1313_2722	1.219	0.286	0.154260506	0.214409916	germination protein
CLO1313_RS13835	Clo1313_2723	1.227	0.296	0.004108279	0.008186531	cysteine--tRNA ligase
CLO1313_RS13845	Clo1313_2725	1.048	0.068	0.682239777	0.744621393	membrane protein
CLO1313_RS13850	Clo1313_2726	0.85	-0.234	0.172703197	0.237803897	8-oxoguanine DNA glycosylase
CLO1313_RS13855	Clo1313_2727	1.286	0.363	2.60E-06	7.84E-06	hypothetical protein
CLO1313_RS13860	Clo1313_2728	0.946	-0.079	0.316710658	0.400596906	hypothetical protein
CLO1313_RS13870	Clo1313_2730	1.209	0.273	0.009176058	0.01725329	hypothetical protein
CLO1313_RS13875	Clo1313_2731	1.245	0.316	0.000436826	0.000990205	amidohydrolase
CLO1313_RS13880	Clo1313_2732	1.486	0.571	3.73E-05	9.72E-05	membrane protein
CLO1313_RS13885	Clo1313_2734	0.821	-0.284	0.000507907	0.001132501	transcriptional regulator
CLO1313_RS13890	Clo1313_2735	0.97	-0.044	0.704836406	0.762555694	two-component system sensor histidine kinase
CLO1313_RS13895	Clo1313_2736	0.93	-0.105	0.414183877	0.499854104	CoA-binding protein
CLO1313_RS13900	Clo1313_2737	0.805	-0.313	0.043511346	0.071189595	hypothetical protein
CLO1313_RS13910	Clo1313_2739	0.856	-0.224	0.060132275	0.094864121	NUDIX hydrolase
CLO1313_RS13920	Clo1313_2741	1.683	0.751	0.058768538	0.093006251	hypothetical protein
CLO1313_RS13925	Clo1313_2742	1.702	0.767	3.16E-10	1.39E-09	DNA polymerase IV
CLO1313_RS13930		1.033	0.047	0.825490049	0.860196198	hypothetical protein

CLO1313_RS13935	Clo1313_2743	1.059	0.082	0.371847672	0.458352309	aminotransferase
CLO1313_RS13940	Clo1313_2744	1.321	0.402	7.50E-08	2.62E-07	peptidase U32
CLO1313_RS13945	Clo1313_2745	1.453	0.539	7.82E-10	3.32E-09	peptidase
CLO1313_RS13950	Clo1313_2746	1.415	0.501	2.38E-07	7.97E-07	hypothetical protein
CLO1313_RS13955	Clo1313_2747	2.996	1.583	2.89E-05	7.63E-05	endoglucanase
CLO1313_RS13960		1.133	0.18	0.116352163	0.168082918	hypothetical protein
CLO1313_RS13965	Clo1313_2748	2.991	1.581	1.45E-32	2.14E-31	hypothetical protein
CLO1313_RS13970	Clo1313_2749	1.07	0.098	0.805044996	0.844762052	hypothetical protein
CLO1313_RS13975	Clo1313_2750	1.181	0.24	0.074808831	0.114699225	dimethyladenosine transferase
CLO1313_RS13980	Clo1313_2751	1.596	0.674	4.18E-08	1.49E-07	hypothetical protein
CLO1313_RS13985	Clo1313_2752	0.882	-0.181	0.051777999	0.083261243	hypothetical protein
CLO1313_RS13990	Clo1313_2753	0.908	-0.139	0.087381557	0.130897748	hydrolase TatD
CLO1313_RS13995	Clo1313_2754	0.877	-0.189	0.008776871	0.016575463	methionyl-tRNA synthetase
CLO1313_RS14000	Clo1313_2755	0.742	-0.431	0.042226864	0.069239128	hypothetical protein
CLO1313_RS14005	Clo1313_2756	0.783	-0.353	0.181843398	0.247998341	spore maturation protein
CLO1313_RS14010	Clo1313_2757	0.688	-0.539	0.199564778	0.26850371	spore maturation protein
CLO1313_RS14015	Clo1313_2758	1.017	0.025	0.784958417	0.829197003	AbrB family transcriptional regulator
CLO1313_RS14025	Clo1313_2760	0.652	-0.618	1.42E-11	7.01E-11	methyltransferase type 11
CLO1313_RS14030	Clo1313_2761	0.918	-0.123	0.067492222	0.104929587	ferredoxin
CLO1313_RS14035	Clo1313_2762	0.831	-0.267	9.89E-05	0.000246022	stage 0 sporulation protein
CLO1313_RS14045	Clo1313_2764	0.865	-0.21	0.018482633	0.032759702	hypothetical protein
CLO1313_RS14050	Clo1313_2765	0.664	-0.59	5.09E-13	2.80E-12	thymidylate kinase
CLO1313_RS14055	Clo1313_2766	0.639	-0.645	1.27E-13	7.28E-13	arginine decarboxylase
CLO1313_RS14060	Clo1313_2767	0.891	-0.167	0.076188248	0.1167545	hypothetical protein
CLO1313_RS14065	Clo1313_2768	0.926	-0.111	0.083299453	0.125788047	copper amine oxidase
CLO1313_RS14070	Clo1313_2769	1.001	0.001	0.992115275	0.993772114	ABC transporter
CLO1313_RS14075	Clo1313_2770	0.954	-0.068	0.527565797	0.607125796	ABC transporter permease
CLO1313_RS14080	Clo1313_2771	1.281	0.358	0.000176087	0.000423144	chemotaxis protein CheY
CLO1313_RS14085	Clo1313_2772	1.03	0.042	0.683152793	0.745280184	two-component system sensor histidine kinase
CLO1313_RS14090	Clo1313_2773	1.599	0.677	1.00E-07	3.46E-07	hypothetical protein

CLO1313_RS14095	Clo1313_2774	1.838	0.878	4.05E-14	2.44E-13	ABC transporter permease
CLO1313_RS14100	Clo1313_2775	1.803	0.851	6.27E-12	3.19E-11	nitrate ABC transporter substrate-binding protein
CLO1313_RS14105	Clo1313_2776	1.886	0.915	8.11E-13	4.34E-12	ABC transporter
CLO1313_RS14110	Clo1313_2777	1.225	0.293	5.88E-05	0.000150613	glycoside hydrolase
CLO1313_RS14130	Clo1313_2781	1.048	0.068	0.797383097	0.839964788	Trypanosome RHS
CLO1313_RS14135	Clo1313_2782	0.955	-0.066	0.861563627	0.89005488	multidrug transporter
CLO1313_RS14140	Clo1313_2783	0.582	-0.782	0.038504567	0.063692883	sugar ABC transporter permease
CLO1313_RS14145	Clo1313_2784	0.46	-1.121	6.37E-05	0.000161442	sugar ABC transporter permease
CLO1313_RS14150	Clo1313_2785	0.385	-1.376	4.69E-15	3.03E-14	ATPase
CLO1313_RS14155	Clo1313_2786	2.345	1.23	7.33E-11	3.43E-10	ABC transporter substrate-binding protein
CLO1313_RS14160		3.748	1.906	1.73E-06	5.30E-06	ABC transporter substrate-binding protein
CLO1313_RS14165	Clo1313_2788	3.686	1.882	1.23E-50	3.75E-49	hypothetical protein
CLO1313_RS14170	Clo1313_2789	3.452	1.787	9.32E-41	2.04E-39	hypothetical protein
CLO1313_RS14175	Clo1313_2790	0.777	-0.364	1.87E-05	5.05E-05	abortive infection protein
CLO1313_RS14180	Clo1313_2791	0.771	-0.375	0.000296278	0.000689859	hypothetical protein
CLO1313_RS14190	Clo1313_2793	2.556	1.354	6.73E-38	1.29E-36	dockerin
CLO1313_RS14195	Clo1313_2794	0.931	-0.103	0.529683085	0.608628188	glycoside hydrolase
CLO1313_RS14200	Clo1313_2795	1.266	0.34	0.002847699	0.005801799	alpha-L-arabinofuranosidase
CLO1313_RS14205	Clo1313_2796	0.781	-0.357	0.001595308	0.00335507	hypothetical protein
CLO1313_RS14210	Clo1313_2797	0.776	-0.365	0.053957552	0.086303306	hypothetical protein
CLO1313_RS14215	Clo1313_2798	0.723	-0.467	7.81E-06	2.22E-05	phosphohydrolase
CLO1313_RS14220	Clo1313_2800	0.84	-0.251	0.002939431	0.005980565	recombination protein RecR
CLO1313_RS14225	Clo1313_2801	0.777	-0.365	1.80E-07	6.07E-07	nucleoid-associated protein
CLO1313_RS14230	Clo1313_2802	0.707	-0.501	2.23E-11	1.08E-10	DNA polymerase III subunit gamma/tau
CLO1313_RS14235		0.722	-0.47	0.16716446	0.231025905	hypothetical protein
CLO1313_RS14240		1.115	0.157	0.466382923	0.548775827	hypothetical protein
CLO1313_RS14245	Clo1313_2804	1.257	0.329	0.270136095	0.351317497	copper amine oxidase
CLO1313_RS14250	Clo1313_2805	1.524	0.607	1.07E-12	5.65E-12	glycoside hydrolase
CLO1313_RS14255	Clo1313_2806	0.595	-0.749	5.63E-16	3.82E-15	carbohydrate-binding protein
CLO1313_RS14260	Clo1313_2807	0.601	-0.735	1.20E-16	8.34E-16	amine oxidase

CLO1313_RS14265	Clo1313_2808	0.669	-0.58	1.61E-10	7.29E-10	membrane protein
CLO1313_RS14270	Clo1313_2809	0.724	-0.467	1.40E-09	5.77E-09	hypothetical protein
CLO1313_RS14275	Clo1313_2810	1.166	0.222	0.044058437	0.071810463	polysaccharide biosynthesis protein GtrA
CLO1313_RS14280	Clo1313_2811	1.195	0.257	0.044329688	0.072174123	type 11 methyltransferase
CLO1313_RS14285	Clo1313_2812	1.108	0.147	0.46472642	0.547413406	hypothetical protein
CLO1313_RS14290	Clo1313_2813	1.098	0.134	0.81844088	0.855583858	hypothetical protein
CLO1313_RS14295	Clo1313_2814	0.855	-0.226	0.716078791	0.770825661	hypothetical protein
CLO1313_RS14300	Clo1313_2815	0.839	-0.254	0.067703429	0.105203411	hypothetical protein
CLO1313_RS14305	Clo1313_2816	0.783	-0.354	4.27E-06	1.26E-05	Spore coat protein CotH
CLO1313_RS14310	Clo1313_2818	5.015	2.326	6.52E-85	4.44E-83	autoinducer
CLO1313_RS14315	Clo1313_2819	1.507	0.592	3.59E-10	1.57E-09	hypothetical protein
CLO1313_RS14320	Clo1313_2820	1.381	0.466	8.61E-06	2.44E-05	peptidase C26
CLO1313_RS14325	Clo1313_2821	0.915	-0.128	0.371823994	0.458352309	anti-sigma factor antagonist
CLO1313_RS14330	Clo1313_2823	0.899	-0.154	0.176144435	0.241544198	rubredoxin
CLO1313_RS14340	Clo1313_2825	0.736	-0.442	1.33E-07	4.54E-07	signal transduction histidine kinase
CLO1313_RS14345	Clo1313_2826	0.719	-0.476	5.03E-06	1.47E-05	glycoside hydrolase
CLO1313_RS14350	Clo1313_2827	0.657	-0.606	8.60E-18	6.43E-17	peptidase M4
CLO1313_RS14355	Clo1313_2828	0.87	-0.2	0.251277853	0.329795309	metallophosphoesterase
CLO1313_RS14360	Clo1313_2829	0.668	-0.582	2.90E-11	1.39E-10	hypothetical protein
CLO1313_RS14365	Clo1313_2830	0.646	-0.63	2.05E-15	1.36E-14	type III restriction endonuclease subunit R
CLO1313_RS14370	Clo1313_2831	0.485	-1.043	9.72E-28	1.19E-26	50S ribosomal protein L31
CLO1313_RS14375	Clo1313_2832	0.792	-0.337	0.000170341	0.000410652	transcription termination factor Rho
CLO1313_RS14380	Clo1313_2834	1.579	0.659	1.00E-12	5.34E-12	hypothetical protein
CLO1313_RS14385	Clo1313_2835	0.798	-0.326	0.000254705	0.000596301	abortive infection protein
CLO1313_RS14390	Clo1313_2836	0.638	-0.648	2.76E-06	8.28E-06	hypothetical protein
CLO1313_RS14395	Clo1313_2837	0.768	-0.382	0.001963182	0.004077273	hypothetical protein
CLO1313_RS14400		1.307	0.387	0.059937539	0.094619743	hypothetical protein
CLO1313_RS14405	Clo1313_2839	1.493	0.579	0.00020368	0.000483258	hypothetical protein
CLO1313_RS14410	Clo1313_2840	0.975	-0.037	0.737333036	0.7894544	acyltransferase
CLO1313_RS14415	Clo1313_2841	0.679	-0.558	6.23E-05	0.000158522	hypothetical protein

CLO1313_RS14420	Clo1313_R0065	0.552	-0.858	3.45E-05	9.03E-05	
CLO1313_RS14425	Clo1313_2842	2.096	1.068	1.06E-07	3.66E-07	pectate lyase
CLO1313_RS14430	Clo1313_2843	1.066	0.092	0.469522137	0.551330028	pectate lyase
CLO1313_RS14435	Clo1313_R0066	0.553	-0.855	2.14E-05	5.73E-05	
CLO1313_RS14440	Clo1313_2844	0.525	-0.931	4.84E-21	4.25E-20	voltage-gated chloride channel protein
CLO1313_RS14445	Clo1313_R0067	0.213	-2.233	1.19E-38	2.38E-37	
CLO1313_RS14450	Clo1313_2845	0.703	-0.508	3.86E-08	1.38E-07	ATPase
CLO1313_RS14455	Clo1313_2846	0.886	-0.175	0.025923164	0.044450297	hypothetical protein
CLO1313_RS14465	Clo1313_2848	0.636	-0.653	0.002364146	0.004862876	hypothetical protein
CLO1313_RS14475	Clo1313_2850	0.441	-1.183	3.08E-69	1.68E-67	single-stranded DNA-binding protein
CLO1313_RS14480	Clo1313_2851	0.487	-1.038	1.46E-45	3.72E-44	30S ribosomal protein S6
CLO1313_RS14485	Clo1313_2852	1.237	0.307	0.000334201	0.0007692	diguanylate cyclase
CLO1313_RS14495	Clo1313_2854	0.69	-0.535	1.02E-13	5.92E-13	glycogen branching protein
CLO1313_RS14500	Clo1313_2855	0.658	-0.604	3.12E-05	8.18E-05	hypothetical protein
CLO1313_RS14505		0.896	-0.159	0.382680249	0.468623139	hypothetical protein
CLO1313_RS14510	Clo1313_2856	1.302	0.381	6.21E-06	1.80E-05	carbohydrate-binding protein
CLO1313_RS14515	Clo1313_2857	1.595	0.673	6.86E-08	2.40E-07	carbohydrate-binding protein
CLO1313_RS14520	Clo1313_2858	1.42	0.505	4.34E-05	0.000112354	carbohydrate-binding protein
CLO1313_RS14525	Clo1313_2859	2.17	1.117	1.95E-07	6.54E-07	carbohydrate-binding protein
CLO1313_RS14530	Clo1313_2860	2.072	1.051	0.024886036	0.04286802	carbohydrate-binding protein
CLO1313_RS14535	Clo1313_2861	3.831	1.938	2.51E-09	1.01E-08	sugar-binding protein
CLO1313_RS14540	Clo1313_2862	0.698	-0.518	0.003829929	0.007657304	HNH endonuclease
CLO1313_RS14545	Clo1313_2863	0.636	-0.653	0.000238986	0.000562573	hypothetical protein
CLO1313_RS14550	Clo1313_2864	0.937	-0.094	0.550403481	0.629062515	MerR family transcriptional regulator
CLO1313_RS14555	Clo1313_2865	1.056	0.079	0.565532365	0.641706986	hypothetical protein
CLO1313_RS14560	Clo1313_2866	1.144	0.194	0.292843684	0.376119146	ABC transporter ATP-binding protein
CLO1313_RS14565	Clo1313_2867	1.123	0.168	0.193994027	0.261888362	membrane protein
CLO1313_RS14570	Clo1313_2868	0.862	-0.214	0.079125732	0.120517049	two-component system response regulator
CLO1313_RS14575	Clo1313_2869	1.087	0.121	0.564260702	0.640638126	hypothetical protein
CLO1313_RS14580	Clo1313_2870	0.909	-0.137	0.372284036	0.458701654	hypothetical protein

CLO1313_RS14585	Clo1313_2871	0.823	-0.281	0.044047199	0.071810463	hypothetical protein
CLO1313_RS14590	Clo1313_2872	0.567	-0.819	3.02E-07	1.00E-06	hypothetical protein
CLO1313_RS14595		0.852	-0.23	0.351378981	0.437800401	hypothetical protein
CLO1313_RS14605	Clo1313_2874	0.697	-0.521	0.001026693	0.002213553	hypothetical protein
CLO1313_RS14610	Clo1313_2875	1.292	0.369	0.000327241	0.00075492	hypothetical protein
CLO1313_RS14625	Clo1313_2878	0.708	-0.499	0.333308861	0.418239864	cyanophycinase
CLO1313_RS14630	Clo1313_2879	0.925	-0.112	0.863143626	0.891380074	hypothetical protein
CLO1313_RS14635	Clo1313_2880	1.021	0.029	0.906550232	0.92694993	peptidase
CLO1313_RS14640	Clo1313_2881	1.108	0.148	0.051936764	0.083427078	LexA family transcriptional regulator
CLO1313_RS14660	Clo1313_2885	1.123	0.168	0.268145253	0.349182637	AraC family transcriptional regulator
CLO1313_RS14665	Clo1313_2886	1.077	0.107	0.435462123	0.520091958	hypothetical protein
CLO1313_RS14670	Clo1313_2887	1.336	0.418	1.46E-05	4.02E-05	dipeptidyl aminopeptidase
CLO1313_RS14675	Clo1313_2888	1.251	0.323	2.51E-05	6.66E-05	magnesium transporter
CLO1313_RS14680		1.198	0.261	0.002751129	0.005620323	hypothetical protein
CLO1313_RS14685	Clo1313_2889	3.174	1.666	3.66E-81	2.39E-79	FlgN family protein
CLO1313_RS14695	Clo1313_2891	1.708	0.772	8.55E-08	2.98E-07	flagellar hook protein FliD
CLO1313_RS14700	Clo1313_2892	2.273	1.184	7.75E-23	7.42E-22	flagellar protein FlaG
CLO1313_RS14705	Clo1313_2893	1.542	0.625	6.46E-15	4.10E-14	NAD-dependent dehydratase
CLO1313_RS14720	Clo1313_2896	1.191	0.252	0.023762762	0.041003753	GlcNAc-PI de-N-acetylase
CLO1313_RS14725	Clo1313_2897	1.188	0.249	0.007677574	0.014656298	methionyl-tRNA formyltransferase
CLO1313_RS14730	Clo1313_2898	1.186	0.246	0.038601087	0.063817343	lactoylglutathione lyase
CLO1313_RS14735	Clo1313_2899	1.164	0.22	0.044714353	0.072682029	hypothetical protein
CLO1313_RS14740	Clo1313_2900	1.059	0.083	0.63544888	0.705035587	phosphopantetheine-binding protein
CLO1313_RS14755	Clo1313_2903	2.833	1.503	6.07E-23	5.86E-22	acylneuraminate cytidylyltransferase
CLO1313_RS14760	Clo1313_2904	5.057	2.338	1.21E-45	3.11E-44	type 11 methyltransferase
CLO1313_RS14765	Clo1313_2905	13.265	3.73	1.53E-123	2.00E-121	membrane protein
CLO1313_RS14770	Clo1313_2906	10.194	3.35	8.55E-134	1.35E-131	hypothetical protein
CLO1313_RS14775	Clo1313_2907	13.563	3.762	2.09E-61	9.23E-60	hypothetical protein
CLO1313_RS14780	Clo1313_2908	14.558	3.864	1.64E-141	3.08E-139	hypothetical protein
CLO1313_RS14785	Clo1313_2909	36.793	5.201	1.13E-190	1.13E-187	flagellin

CLO1313_RS14790	Clo1313_2910	28.2	4.818	2.24E-201	3.36E-198	flagellin
CLO1313_RS14795	Clo1313_2911	0.993	-0.01	0.928516459	0.94266109	aldehyde dehydrogenase
CLO1313_RS14805	Clo1313_2913	1.219	0.285	0.080225272	0.122067779	flagellar assembly factor FliW
CLO1313_RS14810	Clo1313_2914	1.225	0.293	0.143223893	0.20043325	hypothetical protein
CLO1313_RS14820	Clo1313_2916	4.448	2.153	1.35E-94	1.16E-92	flagellar hook protein FlgK
CLO1313_RS14825	Clo1313_2917	4.017	2.006	1.55E-98	1.45E-96	flagellar hook protein FlgK
CLO1313_RS14830	Clo1313_2918	2.941	1.556	2.98E-34	4.75E-33	FlgN family protein
CLO1313_RS14840	Clo1313_2920	1.587	0.666	1.06E-08	4.01E-08	MerR family transcriptional regulator
CLO1313_RS14845	Clo1313_2921	0.788	-0.344	0.006271251	0.012121338	phosphoribosyltransferase
CLO1313_RS14850	Clo1313_2922	0.75	-0.416	3.27E-06	9.73E-06	ATPase AAA
CLO1313_RS14855	Clo1313_2923	0.484	-1.048	2.12E-05	5.69E-05	hypothetical protein
CLO1313_RS14860	Clo1313_2924	0.35	-1.515	1.54E-39	3.24E-38	S-adenosylmethionine synthase
CLO1313_RS14865	Clo1313_2925	0.788	-0.343	2.03E-06	6.17E-06	thioesterase
CLO1313_RS14870	Clo1313_2926	1.408	0.494	7.10E-09	2.74E-08	cell division protein FtsH
CLO1313_RS14885	Clo1313_2929	1	0	0.995365289	0.996694659	replicative DNA helicase
CLO1313_RS14890	Clo1313_2930	1.029	0.042	0.561119804	0.637665135	50S ribosomal protein L9
CLO1313_RS14895	Clo1313_2931	0.768	-0.381	9.40E-06	2.65E-05	phosphoesterase
CLO1313_RS14900	Clo1313_2932	0.897	-0.157	0.102590616	0.150375981	hypothetical protein
CLO1313_RS14905	Clo1313_2933	0.993	-0.01	0.883327752	0.90660504	prephenate dehydratase
CLO1313_RS14910	Clo1313_2934	0.975	-0.036	0.672641315	0.73703007	hypothetical protein
CLO1313_RS14915	Clo1313_2935	0.934	-0.098	0.17361126	0.23883494	ATPase
CLO1313_RS14920	Clo1313_2936	0.944	-0.084	0.26384399	0.345079863	V-type ATP synthase subunit K
CLO1313_RS14925	Clo1313_2937	0.935	-0.097	0.19049062	0.258269131	V-type ATP synthase subunit E
CLO1313_RS14930	Clo1313_2938	0.974	-0.037	0.624880632	0.69551447	ATP synthase subunit C
CLO1313_RS14935	Clo1313_2939	0.975	-0.037	0.624926415	0.69551447	V-type ATP synthase subunit F
CLO1313_RS14940	Clo1313_2940	0.928	-0.108	0.127686923	0.18243596	V-type ATP synthase alpha chain
CLO1313_RS14945	Clo1313_2941	0.972	-0.041	0.558199823	0.635068766	ATP synthase subunit beta
CLO1313_RS14950	Clo1313_2942	0.948	-0.078	0.301394189	0.383813661	V-type ATP synthase subunit D
CLO1313_RS14955	Clo1313_2943	1.111	0.152	0.175294813	0.240801817	ABC-F type ribosomal protection protein
CLO1313_RS14960	Clo1313_2944	2.329	1.22	0.014714342	0.026439971	dockerin

CLO1313_RS14965	Clo1313_2945	1.315	0.395	0.217301406	0.289896315	hypothetical protein
CLO1313_RS14970	Clo1313_2946	0.942	-0.087	0.648847507	0.716719585	hypothetical protein
CLO1313_RS14975	Clo1313_2947	1.114	0.156	0.378954086	0.465199878	hypothetical protein
CLO1313_RS14980		1.18	0.239	0.093457675	0.138958635	ATPase
CLO1313_RS14985	Clo1313_2949	0.975	-0.036	0.939662666	0.952043356	hypothetical protein
CLO1313_RS14990	Clo1313_2950	0.734	-0.446	4.04E-08	1.45E-07	ATPase AAA
CLO1313_RS14995	Clo1313_2951	0.481	-1.055	9.64E-18	7.14E-17	hypothetical protein
CLO1313_RS15000	Clo1313_2952	0.772	-0.373	5.23E-09	2.03E-08	amino acid ABC transporter substrate-binding protein
CLO1313_RS15005	Clo1313_2953	0.716	-0.482	3.10E-10	1.37E-09	polar amino acid ABC transporter permease
CLO1313_RS15010	Clo1313_2954	0.702	-0.51	1.14E-09	4.75E-09	ABC transporter
CLO1313_RS15015	Clo1313_2955	1.04	0.057	0.529659322	0.608628188	two-component system protein-glutamate methylesterase response regulator
CLO1313_RS15020	Clo1313_2956	2.646	1.404	1.33E-34	2.15E-33	chemotaxis protein CheR
CLO1313_RS15025	Clo1313_2957	3.127	1.645	1.61E-88	1.27E-86	chemotaxis protein
CLO1313_RS15030	Clo1313_2958	3.365	1.75	1.42E-74	8.51E-73	chemotaxis protein CheA
CLO1313_RS15035	Clo1313_2959	2.61	1.384	2.06E-36	3.68E-35	chemotaxis protein CheW
CLO1313_RS15040	Clo1313_2961	1.628	0.704	5.63E-08	1.98E-07	recombinase XerD
CLO1313_RS15045	Clo1313_2962	0.679	-0.558	0.000117089	0.000286887	AraC family transcriptional regulator
CLO1313_RS15050	Clo1313_2963	1.067	0.093	0.51602501	0.594986161	phosphatidylethanolamine-binding protein
CLO1313_RS15055	Clo1313_2964	1.39	0.475	0.243074327	0.319728029	hypothetical protein
CLO1313_RS15060	Clo1313_2965	1.703	0.768	0.379575231	0.465581235	multidrug ABC transporter ATP-binding protein
CLO1313_RS15075	Clo1313_2967	0.753	-0.408	0.594896097	0.669699706	hypothetical protein
CLO1313_RS15085	Clo1313_2970	1.013	0.018	0.95564647	0.964328319	subtype I-B CRISPR-associated endonuclease Cas1
CLO1313_RS15095	Clo1313_2972	0.622	-0.684	0.000589137	0.001298179	CRISPR-associated protein Cas3
CLO1313_RS15100	Clo1313_2973	0.526	-0.926	9.58E-12	4.79E-11	CRISPR-associated protein Cas5
CLO1313_RS15110	Clo1313_2975	0.364	-1.458	1.73E-21	1.56E-20	hypothetical protein
CLO1313_RS15120	Clo1313_2977	0.83	-0.268	0.000406277	0.00092445	5,10-methylenetetrahydrofolate reductase
CLO1313_RS15125	Clo1313_2978	0.826	-0.275	0.000558498	0.001238857	UDP pyrophosphate phosphatase
CLO1313_RS15130	Clo1313_2979	0.903	-0.147	0.03321588	0.055775153	MATE family efflux transporter
CLO1313_RS15135	Clo1313_2980	14.917	3.899	2.23E-102	2.30E-100	hemerythrin

CLO1313_RS15140	Clo1313_2981	4.542	2.183	5.08E-59	2.06E-57	chemotaxis protein CheX
CLO1313_RS15145	Clo1313_2982	1.151	0.203	0.044645015	0.072608677	apolipoprotein acyltransferase
CLO1313_RS15150		1.27	0.345	0.557103181	0.634061647	hypothetical protein
CLO1313_RS15155	Clo1313_2983	0.783	-0.354	0.084363703	0.12720299	copper amine oxidase
CLO1313_RS15160	Clo1313_R0068	0.614	-0.704	2.39E-10	1.07E-09	
CLO1313_RS15165	Clo1313_2984	1.267	0.342	2.99E-06	8.93E-06	hypothetical protein
CLO1313_RS15170	Clo1313_2985	1.288	0.365	0.020267589	0.035462369	FMN-binding protein
CLO1313_RS15175	Clo1313_2986	0.915	-0.128	0.121884922	0.174979838	hypothetical protein
CLO1313_RS15180	Clo1313_2987	0.895	-0.16	0.053430651	0.085597501	ribosomal RNA large subunit methyltransferase H
CLO1313_RS15185	Clo1313_2988	0.831	-0.267	0.000240875	0.000566575	MBL fold metallo-hydrolase
CLO1313_RS15190	Clo1313_2989	1.012	0.017	0.811882181	0.851043223	UDP-N-acetylglucosamine 1-carboxyvinyltransferase
CLO1313_RS15195	Clo1313_2990	0.608	-0.717	4.90E-12	2.50E-11	hypothetical protein
CLO1313_RS15200	Clo1313_2991	0.553	-0.855	6.02E-25	6.41E-24	hypothetical protein
CLO1313_RS15205	Clo1313_2992	0.605	-0.725	4.59E-13	2.53E-12	hypothetical protein
CLO1313_RS15210	Clo1313_2993	0.862	-0.215	0.001687698	0.003544403	sensor histidine kinase
CLO1313_RS15215	Clo1313_2994	0.845	-0.243	0.00088471	0.001915701	two-component system response regulator
CLO1313_RS15220	Clo1313_2995	0.889	-0.17	0.269063454	0.350074316	hypothetical protein
CLO1313_RS15225	Clo1313_2996	0.885	-0.177	0.098106174	0.144580057	TetR family transcriptional regulator
CLO1313_RS15230	Clo1313_2997	0.732	-0.451	1.49E-11	7.29E-11	nucleoside-diphosphate sugar epimerase
CLO1313_RS15235	Clo1313_2998	0.588	-0.767	7.13E-16	4.82E-15	polysaccharide biosynthesis protein
CLO1313_RS15240	Clo1313_2999	0.725	-0.465	3.36E-07	1.11E-06	glycosyl transferase family 1
CLO1313_RS15245	Clo1313_3000	0.873	-0.197	0.131402372	0.186727383	hypothetical protein
CLO1313_RS15250	Clo1313_3001	0.83	-0.269	0.001753512	0.003667212	glycosyl transferase family 1
CLO1313_RS15255	Clo1313_3002	1.126	0.171	0.064005086	0.099974611	glycosyl transferase family 1
CLO1313_RS15260	Clo1313_3003	0.733	-0.448	5.28E-08	1.87E-07	UDP-N-acetyl-D-galactosamine dehydrogenase
CLO1313_RS15265	Clo1313_3004	0.716	-0.482	9.97E-07	3.14E-06	glycosyl transferase
CLO1313_RS15270	Clo1313_3005	0.714	-0.487	7.11E-07	2.29E-06	hypothetical protein
CLO1313_RS15275	Clo1313_3006	0.63	-0.667	3.05E-08	1.10E-07	acetyltransferase
CLO1313_RS15280	Clo1313_3007	0.702	-0.511	2.11E-09	8.56E-09	sugar transferase
CLO1313_RS15285	Clo1313_3008	0.875	-0.193	0.016072354	0.028793901	aminotransferase DegT

CLO1313_RS15290	Clo1313_3009	0.653	-0.614	1.69E-12	8.83E-12	polymerase
CLO1313_RS15295	Clo1313_3010	0.601	-0.735	1.47E-14	9.10E-14	LPS biosynthesis protein
CLO1313_RS15300	Clo1313_3011	0.62	-0.689	5.08E-19	4.04E-18	hypothetical protein
CLO1313_RS15305	Clo1313_3012	2.457	1.297	8.79E-18	6.56E-17	spore protein
CLO1313_RS15310	Clo1313_3013	1.263	0.337	0.056082605	0.089273744	cell wall hydrolase
CLO1313_RS15315	Clo1313_3014	1.25	0.322	0.034743141	0.057950322	hypothetical protein
CLO1313_RS15320	Clo1313_3015	1.192	0.253	0.0234071	0.040436574	hypothetical protein
CLO1313_RS15325	Clo1313_3016	1.099	0.136	0.13654853	0.192800867	hypothetical protein
CLO1313_RS15340	Clo1313_3019	1.015	0.022	0.75513731	0.803925024	quinolinate synthetase
CLO1313_RS15345	Clo1313_3020	1.055	0.077	0.339497381	0.4249385	hypothetical protein
CLO1313_RS15350	Clo1313_3021	1.349	0.431	4.09E-07	1.34E-06	accessory gene regulator B
CLO1313_RS15360	Clo1313_R0070	0.84	-0.252	0.456716566	0.540099756	
CLO1313_RS15365	Clo1313_R0071	0.772	-0.374	0.017479686	0.031092276	
CLO1313_RS15370	Clo1313_R0072	0.872	-0.198	0.551993911	0.630159779	
CLO1313_RS15375	Clo1313_3022	1.269	0.344	4.34E-05	0.000112395	5'-nucleotidase
CLO1313_RS15380	Clo1313_3023	1.236	0.305	0.106345122	0.15512112	glycosyl hydrolase
CLO1313_RS15385	Clo1313_3024	0.939	-0.091	0.17108576	0.235901698	DNA gyrase subunit A
CLO1313_RS15395	Clo1313_3026	0.871	-0.199	0.041729259	0.06849811	ribosomal RNA small subunit methyltransferase G
CLO1313_RS15400	Clo1313_3027	0.822	-0.283	1.01E-05	2.84E-05	tRNA uridine 5-carboxymethylaminomethyl modification enzyme MnmG
CLO1313_RS15405	Clo1313_3028	0.758	-0.4	7.25E-06	2.08E-05	tRNA modification GTPase MnmE
CLO1313_RS15410	Clo1313_3029	0.69	-0.536	2.85E-12	1.47E-11	DNA-binding protein
CLO1313_RS15415	Clo1313_3030	0.672	-0.573	5.91E-13	3.22E-12	protein translocase component YidC
CLO1313_RS15420	Clo1313_3031	0.688	-0.541	2.97E-08	1.07E-07	membrane protein insertion efficiency factor YidD
CLO1313_RS15425	Clo1313_3032	0.719	-0.476	8.01E-09	3.08E-08	ribonuclease P protein component
CLO1313_RS15430	Clo1313_3033	0.665	-0.589	5.33E-10	2.29E-09	50S ribosomal protein L34
CLO1313_RS15440	Clo1313_0628	2.399	1.262	5.64E-14	3.34E-13	cellulosome anchoring protein cohesin subunit
CLO1313_RS15445		1.671	0.741	0.001887076	0.003921927	hypothetical protein
CLO1313_RS15480		0.423	-1.241	9.97E-09	3.78E-08	
CLO1313_RS15490		4.458	2.156	9.97E-174	3.74E-171	

CLO1313_RS15495		6.976	2.802	0.181782792	0.247998341	
CLO1313_RS15500		1.585	0.664	0.000926766	0.002003475	
CLO1313_RS15505		1.082	0.114	0.466468403	0.548775827	
CLO1313_RS15525		0.78	-0.359	0.657882245	0.724298404	
CLO1313_RS15530		0.956	-0.064	0.838656141	0.871191467	
CLO1313_RS15535		0.712	-0.49	1.18E-07	4.05E-07	
CLO1313_RS15540		0.977	-0.034	0.913549495	0.932516996	
CLO1313_RS15550		0.693	-0.529	0.387113266	0.472893151	
CLO1313_RS15555		1.034	0.048	0.821014396	0.857147737	
CLO1313_RS15560		1.215	0.281	0.44677288	0.53127354	
CLO1313_RS15570		1.007	0.01	0.964033825	0.971484356	
CLO1313_RS15575		1.57	0.651	9.36E-08	3.25E-07	
CLO1313_RS15580		1.258	0.331	0.455482043	0.539277792	
CLO1313_RS15585		0.969	-0.045	0.650593193	0.717854667	
CLO1313_RS15590		0.551	-0.859	1.50E-10	6.81E-10	
CLO1313_RS15600		0.69	-0.535	8.46E-13	4.52E-12	
CLO1313_RS15605		1.234	0.303	0.006357188	0.012276374	
CLO1313_RS15610		1.92	0.941	8.88E-14	5.19E-13	
CLO1313_RS15620		1.144	0.194	0.879636874	0.905295465	
CLO1313_RS15630		1.278	0.354	0.312620804	0.395924743	
CLO1313_RS15635		0.967	-0.049	0.60351502	0.676866695	
CLO1313_RS15640		2.826	1.499	9.26E-58	3.52E-56	
CLO1313_RS15645		1.402	0.488	0.011397529	0.021034577	
CLO1313_RS15655		0.919	-0.121	0.396265265	0.482500824	
CLO1313_RS15665		2.19	1.131	0.001335599	0.002838739	
CLO1313_RS15670		1.261	0.334	0.200999404	0.269949491	
CLO1313_RS15685		3.071	1.619	0.010518043	0.019531648	
CLO1313_RS15695		1.098	0.135	0.36906386	0.455857709	
CLO1313_RS15760		1.046	0.065	0.604906977	0.678174214	
CLO1313_RS15785		1.321	0.402	0.74226123	0.793676264	

CLO1313_RS15790		0.937	-0.094	0.881983812	0.905894147	
CLO1313_RS15800		1.264	0.338	0.057676323	0.091519202	
CLO1313_RS15830		0.434	-1.206	2.98E-18	2.26E-17	
clpA		0.897	-0.156	0.047388249	0.076737235	
clpP		0.723	-0.467	4.03E-09	1.59E-08	
cls		1.112	0.153	0.137700366	0.193788549	
coaBC		1.005	0.007	0.916076488	0.93382508	
cobA		0.833	-0.264	0.210446201	0.281627915	
cobC		0.738	-0.439	0.000219545	0.000518847	
cobD		0.827	-0.274	0.00010427	0.00025737	
cobT		0.725	-0.464	6.08E-05	0.000154989	
cooS		0.209	-2.26	4.84E-38	9.36E-37	
cphA		0.959	-0.06	0.689008153	0.749758872	
csaB		0.712	-0.491	1.05E-07	3.62E-07	
csrA		1.205	0.268	0.011599813	0.021325028	
cwlD		0.739	-0.437	9.24E-06	2.61E-05	
cysE		1.063	0.088	0.467176165	0.549005219	
cysK		0.396	-1.336	3.43E-64	1.61E-62	
cysT		0.198	-2.333	6.18E-25	6.53E-24	
cysW		0.176	-2.51	6.64E-24	6.69E-23	
dapB		1.727	0.788	1.16E-05	3.21E-05	
dcd		1.771	0.824	4.84E-20	4.07E-19	
def		1.053	0.075	0.386010744	0.472123255	
deoA		1.05	0.07	0.343750839	0.429009058	
deoC		0.954	-0.068	0.623005261	0.694053781	
deoD		1.778	0.83	1.19E-13	6.83E-13	
dnaJ		0.986	-0.02	0.786403941	0.829847086	
dprA		0.754	-0.408	7.79E-07	2.49E-06	
dxs		0.913	-0.132	0.060861448	0.095913549	
efp		0.817	-0.291	4.06E-06	1.20E-05	

essC		0.704	-0.506	2.27E-09	9.19E-09	
fabD		0.575	-0.799	0.011314548	0.020894291	
fabF		0.671	-0.576	3.43E-05	8.98E-05	
fabG		0.63	-0.666	0.035348834	0.058895085	
fabZ		0.54	-0.89	7.89E-20	6.57E-19	
fba		0.824	-0.279	5.18E-06	1.52E-05	
feoB		2.036	1.026	6.26E-13	3.40E-12	
ffs		0.849	-0.237	0.014389303	0.025933606	
flgB		0.984	-0.023	0.798390228	0.840130629	
flgC		1.161	0.215	0.012436152	0.022741476	
flgG		8.527	3.092	1.23E-98	1.19E-96	
flgL		4.161	2.057	9.62E-75	5.89E-73	
flgM		4.457	2.156	9.19E-86	6.72E-84	
flhA		2.248	1.169	3.37E-28	4.26E-27	
flhB		2.25	1.17	7.56E-33	1.14E-31	
flhF		2.348	1.232	8.77E-38	1.67E-36	
fliJ		1.612	0.689	2.47E-06	7.47E-06	
fliP		1.977	0.983	1.48E-11	7.27E-11	
fliQ		2.12	1.084	9.25E-15	5.82E-14	
fliS		2.203	1.139	1.28E-26	1.49E-25	
folB		0.843	-0.247	0.003391839	0.006854531	
folE		1.071	0.099	0.284925363	0.367703181	
folK		0.977	-0.033	0.675975876	0.739602938	
folP		0.898	-0.156	0.025391583	0.0436386	
ftsA		1.04	0.056	0.415765122	0.501157396	
ftsE		0.868	-0.205	0.013772437	0.024896647	
fusA		0.527	-0.925	8.07E-38	1.54E-36	
galE		0.882	-0.181	0.009659398	0.018071451	
galU		1.095	0.131	0.064626602	0.100787924	
gap		0.721	-0.473	2.07E-13	1.17E-12	

glgD		1.456	0.542	3.16E-11	1.51E-10	
glmS		1.005	0.007	0.918218376	0.935372591	
glmU		0.622	-0.685	9.23E-14	5.38E-13	
glnA		2.084	1.059	2.32E-26	2.65E-25	
gltA		1.09	0.125	0.219559918	0.292466787	
groL		0.804	-0.315	2.02E-05	5.43E-05	
gyrB		0.822	-0.282	5.14E-05	0.000132082	
hemA		0.277	-1.854	1.73E-08	6.40E-08	
hemL		1.104	0.143	0.570909115	0.64654638	
hemZ		0.911	-0.135	0.12414379	0.177882096	
hflK		1.111	0.152	0.107313796	0.156306009	
hflX		0.889	-0.17	0.043815895	0.071570735	
hisA		0.71	-0.495	1.63E-08	6.08E-08	
hisC		1.212	0.277	0.030973238	0.052361184	
hisD		1.22	0.287	0.026512172	0.045382422	
hisH		0.781	-0.356	0.00019988	0.000474991	
hisZ		1.014	0.02	0.881550632	0.905894147	
holB		0.955	-0.066	0.468054215	0.549821618	
hprK		1.005	0.007	0.915341284	0.933746144	
hpt		1.174	0.231	0.086942558	0.130435584	
hrcA		2.137	1.095	8.52E-53	2.81E-51	
hydE		1.219	0.285	0.010119982	0.018862539	
hydF		3.08	1.623	1.87E-69	1.04E-67	
hypA		0.691	-0.533	1.46E-09	6.00E-09	
hypB		0.735	-0.443	1.18E-08	4.43E-08	
hypC		0.928	-0.108	0.372548786	0.458839347	
hypE		1.001	0.001	0.991477281	0.993464873	
hypF		1.014	0.02	0.809480298	0.848822172	
ilvB		0.767	-0.383	2.00E-05	5.38E-05	
ilvD		0.756	-0.404	8.09E-07	2.57E-06	

iorA		0.826	-0.275	0.004347094	0.008633733	
iorB		0.867	-0.207	0.096112621	0.142201159	
ispD		0.605	-0.725	4.27E-15	2.76E-14	
lepB		0.89	-0.168	0.008340037	0.015830234	
leuB		1.294	0.372	5.97E-05	0.000152583	
leuC		1.358	0.441	2.31E-06	6.99E-06	
leuD		1.422	0.508	1.67E-08	6.21E-08	
lon		1.213	0.278	0.013877042	0.025070631	
lonB		1.116	0.158	0.390506883	0.476650444	
lspA		0.98	-0.029	0.749066784	0.799733459	
lysA		3.243	1.697	1.46E-53	4.97E-52	
map		0.689	-0.537	5.04E-09	1.96E-08	
metF		0.866	-0.208	0.022991618	0.039856568	
mfd		0.91	-0.136	0.136769849	0.192931692	
mgtE		0.76	-0.395	2.06E-08	7.55E-08	
miaB		1.068	0.095	0.196686468	0.26510684	
minD		1.274	0.349	4.11E-07	1.35E-06	
mraW		0.389	-1.364	9.75E-54	3.36E-52	
mrdA		1.167	0.222	0.002117756	0.004377085	
mreC		0.943	-0.085	0.297395321	0.380498536	
mreD		0.971	-0.042	0.631847839	0.7015593	
msrA		0.817	-0.292	0.077958028	0.119040797	
mtnA		0.886	-0.175	0.034422794	0.057511954	
murA		0.928	-0.107	0.133307974	0.189115712	
murG		0.887	-0.172	0.034150756	0.057089252	
mviN		1.059	0.083	0.394874477	0.481198114	
nadB		0.863	-0.213	0.005058547	0.009941404	
nadC		1.095	0.13	0.221905831	0.295250926	
nagA		1.07	0.098	0.327308409	0.411744093	
neuB		1.984	0.988	0.178154864	0.243966409	

neuC		1.433	0.519	0.494993604	0.574733082	
nifH		0.212	-2.238	1.96E-28	2.53E-27	
nifJ		0.811	-0.303	9.29E-05	0.000232069	
nifS		1.173	0.23	0.085844719	0.129046774	
nifU		1.307	0.386	0.001107319	0.002380538	
noc		0.944	-0.083	0.402588672	0.488314161	
nth		0.675	-0.567	1.68E-08	6.25E-08	
nusA		0.709	-0.495	4.52E-12	2.31E-11	
nusB		1.195	0.257	0.001463636	0.003097702	
panB		2.019	1.014	5.00E-14	2.98E-13	
pcrA		0.67	-0.579	2.29E-11	1.11E-10	
pdaA		1.387	0.472	0.01467234	0.026380305	
pepF		0.61	-0.714	5.11E-21	4.47E-20	
pfkA		1.17	0.227	0.006145538	0.011898301	
pflA		1.334	0.416	1.60E-05	4.37E-05	
pgk		0.745	-0.424	2.18E-08	7.95E-08	
pgsA		1.157	0.211	0.006978562	0.01339866	
plsY		1.008	0.012	0.877637818	0.903858453	
polC		0.945	-0.081	0.194036659	0.261888362	
porA		0.215	-2.22	1.22E-167	3.03E-165	
prmC		1.03	0.043	0.64073691	0.710105473	
proB		1.116	0.158	0.019645235	0.034453836	
pseC		1.248	0.319	0.006272863	0.012121338	
pseG		1.274	0.35	0.001068489	0.002300001	
pseH		0.964	-0.052	0.785510373	0.829479477	
pseI		0.849	-0.236	0.175362177	0.240801817	
pta		0.295	-1.759	8.41E-37	1.55E-35	
ptsP		1.084	0.116	0.301595272	0.383906715	
purE		1.662	0.733	1.71E-07	5.78E-07	
purH		1.496	0.581	1.66E-05	4.52E-05	

pyrF		1.172	0.229	0.159283154	0.220948279	
queC		1.283	0.36	7.21E-07	2.32E-06	
queD		1.348	0.431	3.03E-07	1.00E-06	
raiA		0.922	-0.117	0.180573661	0.246827899	
recA		0.944	-0.083	0.402280543	0.488314161	
recJ		0.706	-0.502	1.41E-05	3.89E-05	
recN		0.73	-0.454	2.88E-09	1.16E-08	
recO		1.016	0.022	0.7879428	0.831178493	
rfbC		1.005	0.008	0.922878432	0.938525744	
rfbF		6.085	2.605	4.85E-76	3.03E-74	
rfbG		4.929	2.301	1.32E-58	5.16E-57	
ribD		1.34	0.422	0.001109741	0.002384035	
ric		1.643	0.716	1.03E-06	3.24E-06	
rimI		0.785	-0.349	0.000796189	0.001727764	
rimO		1.117	0.159	0.013578192	0.024619708	
rnpB		1.441	0.527	4.94E-11	2.34E-10	
rnr		0.763	-0.39	0.000292581	0.00068178	
rny		0.855	-0.226	0.000429333	0.000973957	
rpiB		1.074	0.102	0.134649461	0.190568066	
rplK		0.481	-1.055	1.81E-38	3.59E-37	
rplS		0.832	-0.266	0.00272353	0.005575335	
rplU		0.518	-0.949	2.63E-52	8.31E-51	
rpmB		0.96	-0.06	0.46409718	0.546887011	
rpmG		0.53	-0.915	6.30E-45	1.54E-43	
rpoB		0.534	-0.905	2.65E-44	6.25E-43	
rpoC		0.6	-0.738	2.69E-23	2.63E-22	
rpsB		0.655	-0.61	1.28E-16	8.86E-16	
rpsR		0.492	-1.024	4.05E-59	1.66E-57	
rpsT		0.616	-0.698	8.31E-25	8.72E-24	
rrf		0.416	-1.265	0.13407079	0.1900181	

rseP		0.763	-0.39	2.66E-08	9.67E-08	
rsfS		0.613	-0.706	2.43E-16	1.67E-15	
rsgA		1.041	0.059	0.352509524	0.43884436	
rsmD		1.239	0.309	0.000302546	0.000702271	
rsmI		0.652	-0.616	4.45E-10	1.91E-09	
scfA		0.931	-0.104	0.164463539	0.227607823	
scfB		0.797	-0.327	1.46E-07	4.96E-07	
scpB		0.87	-0.2	0.013285243	0.024132309	
secD		0.781	-0.356	7.27E-07	2.33E-06	
secE		0.539	-0.892	3.43E-39	7.04E-38	
secF		0.802	-0.319	0.000100591	0.000249316	
sfsA		1.351	0.434	0.000171341	0.0004124	
sigF		1.013	0.019	0.909957067	0.929998682	
sigG		2.332	1.222	0.137122853	0.193247855	
sigI		1.024	0.034	0.708656622	0.764540237	
sigK		0.574	-0.8	4.68E-06	1.38E-05	
sleB		0.779	-0.36	0.000814939	0.001767173	
smc		0.912	-0.134	0.058692683	0.092935246	
speB		0.426	-1.23	2.73E-35	4.63E-34	
spo0A		1.067	0.094	0.190788357	0.258551415	
spoIIAA		0.674	-0.568	0.1465871	0.204853082	
spoIID		1.206	0.271	0.332346562	0.417206923	
spoIIE		1.2	0.263	0.027459053	0.046842833	
spoIIGA		1.095	0.13	0.594746055	0.669699706	
spoIIIAA		0.671	-0.576	0.006499948	0.012543979	
spoIIIAB		0.746	-0.422	0.125345455	0.179261335	
spoIIIAC		0.874	-0.194	0.612142853	0.68526182	
spoIIIAD		0.827	-0.275	0.400789041	0.487020394	
spoIIIAE		0.738	-0.438	0.062935251	0.098547164	
spoIIIAF		0.684	-0.547	0.155097642	0.215441328	

spoIIIAG		0.932	-0.102	0.78295033	0.827658809	
spoIIID		0.251	-1.993	0.48766707	0.567966425	
spoIIM		2.287	1.193	0.021431032	0.037367248	
spoIIR		0.936	-0.095	0.821376989	0.857147737	
spoIVA		1.414	0.499	0.001751877	0.00366635	
spoIVB		1.533	0.617	3.84E-10	1.67E-09	
spoVAC		0.538	-0.895	0.369699362	0.456454668	
spoVAD		0.977	-0.034	0.920770654	0.93701771	
spoVAE		1.185	0.245	0.641699228	0.710655829	
spoVB		1.036	0.051	0.736346634	0.789280017	
spoVT		1.074	0.103	0.739598538	0.791315025	
sppA		0.734	-0.446	3.29E-09	1.31E-08	
ssrA		0.894	-0.162	0.265203467	0.346706712	
ssrS		1.818	0.862	1.43E-11	7.05E-11	
thiF		2.282	1.19	2.55E-10	1.14E-09	
thiS		2.22	1.15	3.89E-11	1.85E-10	
thyA		0.882	-0.181	0.013195352	0.023998096	
tilS		1.08	0.111	0.153943238	0.214137179	
trmL		0.862	-0.214	0.015459682	0.027712842	
trpB		0.626	-0.676	2.45E-15	1.61E-14	
trpD		1.375	0.459	0.019255123	0.033928386	
trpE		1.063	0.088	0.456011456	0.539691537	
trpS		0.897	-0.157	0.031120222	0.052580027	
trxA		1.354	0.437	0.001130853	0.00242418	
trxB		1.277	0.353	0.000355237	0.000815116	
tsaB		0.888	-0.171	0.077416891	0.118395337	
tsaD		1.037	0.052	0.47802881	0.558671822	
tsf		0.633	-0.659	5.26E-23	5.09E-22	
tuf		0.478	-1.065	1.54E-44	3.69E-43	
typA		0.669	-0.581	2.47E-13	1.39E-12	

ureA		2.455	1.296	0.030715485	0.051954732	
ureB		3.015	1.592	0.002111254	0.004366655	
ureC		2.199	1.137	2.94E-08	1.06E-07	
ureG		1.769	0.823	0.014222493	0.025679263	
urtA		9.262	3.211	4.17E-23	4.06E-22	
urtB		5.615	2.489	3.79E-12	1.95E-11	
urtC		7.152	2.838	3.75E-17	2.68E-16	
urtD		5.704	2.512	6.74E-08	2.36E-07	
urtE		4.574	2.193	1.14E-05	3.17E-05	
whiA		0.888	-0.172	0.039336598	0.064925953	
xth		0.738	-0.438	9.41E-06	2.65E-05	
yabG		1.071	0.099	0.882724869	0.906296433	
yabP		2.176	1.122	0.192102801	0.259863014	
yabQ		1.402	0.488	0.274938151	0.356634738	
yajC		1.17	0.226	0.073257984	0.112551585	
yjjW		2.565	1.359	1.08E-13	6.26E-13	
ylbJ		1.291	0.368	8.08E-07	2.57E-06	
ylqF		0.939	-0.091	0.206444041	0.276766061	
ypeB		1.532	0.615	0.046833846	0.075921462	
yqfC		1.208	0.272	0.422048556	0.506694804	
yqfD		1.145	0.196	0.008536283	0.016182246	
ytfJ		1.132	0.179	0.36651205	0.453452821	
ytvI		0.831	-0.267	0.000516556	0.001150929	
ytxC		1.166	0.222	0.070842502	0.109513744	
yunB		0.505	-0.986	4.78E-07	1.56E-06	
yyaC		1.21	0.275	0.000190508	0.000454523	

Supplemental Table S2. DEGs not mapped in pathview.

porA
cysK
rpsR
mraW
tuf
rplK
cooS
pta
speB
argF
nifH
argB
cysT
cysW
CLO1313_RS15830
CLO1313_RS12770
cas6
CLO1313_RS15480
hemA
CLO1313_RS12690
CLO1313_RS15490
flgG
flgM
rfbF
flgL
hydF
CLO1313_RS02590
rfbG
CLO1313_RS15640
lysA
hrcA
flhF
CLO1313_RS04145
flhB
CLO1313_RS04410
flhA
fliS

glnA
urtA
cas4
urtC
CLO1313_RS09800
fliQ
panB
yjjW
asnB
feoB
urtB
thiS
thiF
CLO1313_RS11360
ureC
urtD
CLO1313_RS14160
urtE
cadA
CLO1313_RS15665
ureB
CLO1313_RS15685
CLO1313_RS10595
spoIIM

Supplemental Table S3. Pathview results.

KEGG pathway	Stat mean	Set size	p.up	p.dn	p.val	q.val
ctx02020 Two-component system	3.674	32	0.000	1.000	0.001	0.006
ctx02030 Bacterial chemotaxis	3.234	32	0.001	0.999	0.002	0.013
ctx00970 Aminoacyl-tRNA biosynthesis	-2.930	20	0.997	0.003	0.006	0.023
ctx02040 Flagellar assembly	2.881	13	0.004	0.996	0.009	0.026
ctx00270 Cysteine and methionine metabolism	-2.546	10	0.989	0.011	0.022	0.052
ctx01100 Metabolic pathways	-1.550	50	0.938	0.062	0.124	0.249
ctx01110 Biosynthesis of secondary metabolites	-1.376	23	0.912	0.088	0.176	0.270
ctx01120 Microbial metabolism in diverse environments	-1.367	20	0.910	0.090	0.180	0.270
ctx01130 Biosynthesis of antibiotics	-1.230	18	0.886	0.114	0.227	0.303
ctx01200 Carbon metabolism	-0.987	10	0.831	0.169	0.337	0.405
ctx02010 ABC transporters	-0.333	20	0.630	0.370	0.741	0.808
ctx01230 Biosynthesis of amino acids	-0.158	17	0.562	0.438	0.876	0.876
ctx00920 Sulfur metabolism	NA	8	NA	NA	NA	NA
ctx03010 Ribosome	NA	7	NA	NA	NA	NA
ctx00680 Methane metabolism	NA	7	NA	NA	NA	NA
ctx00250 Alanine, aspartate and glutamate metabolism	NA	6	NA	NA	NA	NA
ctx00620 Pyruvate metabolism	NA	6	NA	NA	NA	NA
ctx00450 Selenocompound metabolism	NA	5	NA	NA	NA	NA
ctx00633 Nitrotoluene degradation	NA	5	NA	NA	NA	NA
ctx00640 Propanoate metabolism	NA	5	NA	NA	NA	NA
ctx00910 Nitrogen metabolism	NA	5	NA	NA	NA	NA
ctx00010 Glycolysis / Gluconeogenesis	NA	4	NA	NA	NA	NA
ctx00020 Citrate cycle (TCA cycle)	NA	4	NA	NA	NA	NA
ctx00240 Pyrimidine metabolism	NA	4	NA	NA	NA	NA
ctx00650 Butanoate metabolism	NA	4	NA	NA	NA	NA
ctx00730 Thiamine metabolism	NA	4	NA	NA	NA	NA
ctx01210 2-Oxocarboxylic acid metabolism	NA	4	NA	NA	NA	NA
ctx00230 Purine metabolism	NA	3	NA	NA	NA	NA
ctx00300 Lysine biosynthesis	NA	3	NA	NA	NA	NA
ctx00330 Arginine and proline metabolism	NA	3	NA	NA	NA	NA
ctx00500 Starch and sucrose metabolism	NA	3	NA	NA	NA	NA
ctx00630 Glyoxylate and dicarboxylate metabolism	NA	3	NA	NA	NA	NA
ctx00030 Pentose phosphate pathway	NA	2	NA	NA	NA	NA
ctx00400 Phenylalanine, tyrosine and tryptophan	NA	2	NA	NA	NA	NA
ctx00460 Cyanoamino acid metabolism	NA	2	NA	NA	NA	NA

Supplemental Table S4. Protein sequences for each down-DEGs classified as biological process.

GO	Number of Seqs	Proteins for Gene (nucleotide) Sequences involved in GO
oxidation-reduction process	17	lcl|NC_017304.1_prot_WP_003517362.1_2335[CLO1313_RS11835],<br>lcl|NC_017304.1_prot_WP_003515288.1_387[CLO1313_RS01975],<br>lcl|NC_017304.1_prot_WP_003514857.1_572[CLO1313_RS02930],<br>lcl|NC_017304.1_prot_WP_003512582.1_1871[CLO1313_RS09520],<br>lcl|NC_017304.1_prot_WP_003513114.1_108[CLO1313_RS00575],<br>lcl|NC_017304.1_prot_WP_003517348.1_2342[CLO1313_RS11870],<br>lcl|NC_017304.1_prot_WP_003518903.1_2557[CLO1313_RS12975],<br>lcl|NC_017304.1_prot_WP_003516455.1_21[CLO1313_RS00120],<br>lcl|NC_017304.1_prot_WP_003512583.1_1870[CLO1313_RS09515],<br>lcl|NC_017304.1_prot_WP_003513282.1_20[CLO1313_RS00115],<br>lcl|NC_017304.1_prot_WP_003516452.1_22[CLO1313_RS00125],<br>lcl|NC_017304.1_prot_WP_003514859.1_571[CLO1313_RS02925],<br>lcl|NC_017304.1_prot_WP_003511903.1_2219[CLO1313_RS11280],<br>lcl|NC_017304.1_prot_WP_003513279.1_23[CLO1313_RS00130],<br>lcl|NC_017304.1_prot_WP_003515293.1_382[CLO1313_RS01950],<br>lcl|NC_017304.1_prot_WP_003514855.1_573[CLO1313_RS02935],<br>lcl|NC_017304.1_prot_WP_003517364.1_2334[CLO1313_RS11830]
transmembrane transport	8	lcl|NC_017304.1_prot_WP_003518681.1_1811[CLO1313_RS09225],<br>lcl|NC_017304.1_prot_WP_003517461.1_1004[CLO1313_RS05130],<br>lcl|NC_017304.1_prot_WP_003511526.1_615[CLO1313_RS03165],<br>lcl|NC_017304.1_prot_WP_003517352.1_2340[CLO1313_RS11860],<br>lcl|NC_017304.1_prot_WP_003516704.1_2782[CLO1313_RS14145],<br>lcl|NC_017304.1_prot_WP_003516282.1_1461[CLO1313_RS07450],<br>lcl|NC_017304.1_prot_WP_003514465.1_389[CLO1313_RS01985],<br>lcl|NC_017304.1_prot_WP_003516281.1_1462[CLO1313_RS07455]
translation	8	lcl|NC_017304.1_prot_WP_003514304.1_316[CLO1313_RS01630],<br>lcl|NC_017304.1_prot_WP_003515019.1_311[CLO1313_RS01605],<br>lcl|NC_017304.1_prot_WP_003515021.1_309[CLO1313_RS01595],<br>lcl|NC_017304.1_prot_WP_003513685.1_2844[CLO1313_RS14470],<br>lcl|NC_017304.1_prot_WP_003513680.1_2846[CLO1313_RS14480],<br>lcl|NC_017304.1_prot_WP_003514305.1_317[CLO1313_RS01635],<br>lcl|NC_017304.1_prot_WP_003515018.1_312[CLO1313_RS01610],<br>lcl|NC_017304.1_prot_WP_003516863.1_2827[CLO1313_RS14370]
acetyl-CoA biosynthetic process from pyruvate	5	lcl|NC_017304.1_prot_WP_003513282.1_20[CLO1313_RS00115],<br>lcl|NC_017304.1_prot_WP_003516452.1_22[CLO1313_RS00125],<br>lcl|NC_017304.1_prot_WP_003513279.1_23[CLO1313_RS00130],<br>lcl|NC_017304.1_prot_WP_003515293.1_382[CLO1313_RS01950],<br>lcl|NC_017304.1_prot_WP_003516455.1_21[CLO1313_RS00120]
phosphorylation	4	lcl|NC_017304.1_prot_WP_003515609.1_1180[CLO1313_RS06035],<br>lcl|NC_017304.1_prot_WP_037295095.1_2558[CLO1313_RS12980],<br>lcl|NC_017304.1_prot_WP_003518357.1_120[CLO1313_RS00635],<br>lcl|NC_017304.1_prot_WP_003515563.1_1205[CLO1313_RS06160]
arginine biosynthetic process	4	lcl|NC_017304.1_prot_WP_003513825.1_2560[CLO1313_RS12990],<br>lcl|NC_017304.1_prot_WP_003513826.1_2561[CLO1313_RS12995],

		lcl|NC_017304.1_prot_WP_003513827.1_2562[CLO1313_RS13000], lcl|NC_017304.1_prot_WP_003518903.1_2557[CLO1313_RS12975]
anion transmembrane transport	4	lcl|NC_017304.1_prot_WP_003513100.1_115[CLO1313_RS00610], lcl|NC_017304.1_prot_WP_003513101.1_114[CLO1313_RS00605], lcl|NC_017304.1_prot_WP_003518358.1_116[CLO1313_RS00615], lcl|NC_017304.1_prot_WP_003513098.1_117[CLO1313_RS00620]
sulfate transmembrane transport	4	lcl|NC_017304.1_prot_WP_003513100.1_115[CLO1313_RS00610], lcl|NC_017304.1_prot_WP_003513101.1_114[CLO1313_RS00605], lcl|NC_017304.1_prot_WP_003518358.1_116[CLO1313_RS00615], lcl|NC_017304.1_prot_WP_003513098.1_117[CLO1313_RS00620]
translational elongation	2	lcl|NC_017304.1_prot_WP_003518357.1_120[CLO1313_RS00635], lcl|NC_017304.1_prot_WP_003514309.1_319[CLO1313_RS01645]
peptidyl-histidine phosphorylation	2	lcl|NC_017304.1_prot_WP_003519346.1_616[CLO1313_RS03170], lcl|NC_017304.1_prot_WP_003512581.1_1872[CLO1313_RS09525]
proteolysis	2	lcl|NC_017304.1_prot_WP_003511670.1_687[CLO1313_RS03565], lcl|NC_017304.1_prot_WP_003513087.1_123[CLO1313_RS00650]
hydrogen sulfide biosynthetic process	2	lcl|NC_017304.1_prot_WP_003513096.1_118[CLO1313_RS00625], lcl|NC_017304.1_prot_WP_003518357.1_120[CLO1313_RS00635]
acetyl-CoA biosynthetic process	2	lcl|NC_017304.1_prot_WP_003515609.1_1180[CLO1313_RS06035], lcl|NC_017304.1_prot_WP_003521237.1_1179[CLO1313_RS06030]
cysteine biosynthetic process from serine	2	lcl|NC_017304.1_prot_WP_003521330.1_2534[CLO1313_RS12835], lcl|NC_017304.1_prot_WP_003517374.1_2329[CLO1313_RS11805]
methylation	2	lcl|NC_017304.1_prot_WP_003516178.1_1514[CLO1313_RS07710], lcl|NC_017304.1_prot_WP_003515671.1_1150[CLO1313_RS05865]
polysaccharide catabolic process	2	lcl|NC_017304.1_prot_WP_003516263.1_1474[CLO1313_RS07515], lcl|NC_017304.1_prot_WP_003511670.1_687[CLO1313_RS03565]
signal transduction by protein phosphorylation	2	lcl|NC_017304.1_prot_WP_003519346.1_616[CLO1313_RS03170], lcl|NC_017304.1_prot_WP_003518008.1_1697[CLO1313_RS08655]
fatty acid biosynthetic process	2	lcl|NC_017304.1_prot_WP_003512155.1_2090[CLO1313_RS10640], lcl|NC_017304.1_prot_WP_003516264.1_1473[CLO1313_RS07510]
'de novo' UMP biosynthetic process	2	lcl|NC_017304.1_prot_WP_003513826.1_2561[CLO1313_RS12995], lcl|NC_017304.1_prot_WP_003513827.1_2562[CLO1313_RS13000]
spermidine biosynthetic process	2	lcl|NC_017304.1_prot_WP_003516175.1_1516[CLO1313_RS07720], lcl|NC_017304.1_prot_WP_003516223.1_1495[CLO1313_RS07620]
cysteine biosynthetic process	2	lcl|NC_017304.1_prot_WP_003513096.1_118[CLO1313_RS00625], lcl|NC_017304.1_prot_WP_003517355.1_2338[CLO1313_RS11850]
phosphorelay signal transduction system	2	lcl|NC_017304.1_prot_WP_003519346.1_616[CLO1313_RS03170], lcl|NC_017304.1_prot_WP_003518008.1_1697[CLO1313_RS08655]
nitrogen fixation	2	lcl|NC_017304.1_prot_WP_003517348.1_2342[CLO1313_RS11870], lcl|NC_017304.1_prot_WP_003517364.1_2334[CLO1313_RS11830]
long-chain fatty acid metabolic process	1	lcl|NC_017304.1_prot_WP_003512157.1_2089[CLO1313_RS10635]
glutamine metabolic process	1	lcl|NC_017304.1_prot_WP_003513826.1_2561[CLO1313_RS12995]
thiosulfate transport	1	lcl|NC_017304.1_prot_WP_003513098.1_117[CLO1313_RS00620]
sulfate reduction	1	lcl|NC_017304.1_prot_WP_003513095.1_119[CLO1313_RS00630]

nucleoside monophosphate phosphorylation	1	lcl|NC_017304.1_prot_WP_003515563.1_1205[CLO1313_RS06160]
one-carbon metabolic process	1	lcl|NC_017304.1_prot_WP_003518486.1_2922[CLO1313_RS14860]
ATP synthesis coupled electron transport	1	lcl|NC_017304.1_prot_WP_003512584.1_1869[CLO1313_RS09510]
ornithine metabolic process	1	lcl|NC_017304.1_prot_WP_003513828.1_2563[CLO1313_RS13005]
chemotaxis	1	lcl|NC_017304.1_prot_WP_003512414.1_1950[CLO1313_RS09930]
DNA replication	1	lcl|NC_017304.1_prot_WP_003513683.1_2845[CLO1313_RS14475]
negative regulation of fatty acid biosynthetic process	1	lcl|NC_017304.1_prot_WP_011837932.1_1272[CLO1313_RS06495]
translational initiation	1	lcl|NC_017304.1_prot_WP_003520198.1_1030[CLO1313_RS05260]
ribosomal small subunit biogenesis	1	lcl|NC_017304.1_prot_WP_003515546.1_1214[CLO1313_RS06205]
signal transduction	1	lcl|NC_017304.1_prot_WP_003512414.1_1950[CLO1313_RS09930]
glycine biosynthetic process from serine	1	lcl|NC_017304.1_prot_WP_003515671.1_1150[CLO1313_RS05865]
regulation of translational termination	1	lcl|NC_017304.1_prot_WP_003512565.1_1884[CLO1313_RS09585]
regulation of translation	1	lcl|NC_017304.1_prot_WP_003515020.1_310[CLO1313_RS01600]
generation of precursor metabolites and energy	1	lcl|NC_017304.1_prot_WP_003515288.1_387[CLO1313_RS01975]
peptidyl-tyrosine dephosphorylation	1	lcl|NC_017304.1_prot_WP_003511903.1_2219[CLO1313_RS11280]
organic acid metabolic process	1	lcl|NC_017304.1_prot_WP_003515609.1_1180[CLO1313_RS06035]
protoporphyrinogen IX biosynthetic process	1	lcl|NC_017304.1_prot_WP_003513114.1_108[CLO1313_RS00575]
ribosome biogenesis	1	lcl|NC_017304.1_prot_WP_003515019.1_311[CLO1313_RS01605]
carbohydrate metabolic process	1	lcl|NC_017304.1_prot_WP_003512623.1_1855[CLO1313_RS09440]
tetrahydrofolate interconversion	1	lcl|NC_017304.1_prot_WP_003515671.1_1150[CLO1313_RS05865]
sulfate assimilation, phosphoadenylyl sulfate reduction by phosphoadenylyl-sulfate reductase (thioredoxin)	1	lcl|NC_017304.1_prot_WP_003513096.1_118[CLO1313_RS00625]
polyamine transport	1	lcl|NC_017304.1_prot_WP_003516280.1_1463[CLO1313_RS07460]
DNA repair	1	lcl|NC_017304.1_prot_WP_003513683.1_2845[CLO1313_RS14475]
pseudouridine synthesis	1	lcl|NC_017304.1_prot_WP_003516173.1_1517[CLO1313_RS07725]
biosynthetic process	1	lcl|NC_017304.1_prot_WP_003515290.1_386[CLO1313_RS01970]

'de novo' pyrimidine nucleobase biosynthetic process	1	lcl|NC_017304.1_prot_WP_003513826.1_2561[CLO1313_RS12995]
putrescine transport	1	lcl|NC_017304.1_prot_WP_003516284.1_1460[CLO1313_RS07445]
arginine biosynthetic process via ornithine	1	lcl|NC_017304.1_prot_WP_037295095.1_2558[CLO1313_RS12980]
S-adenosylmethioninamine biosynthetic process	1	lcl|NC_017304.1_prot_WP_003516223.1_1495[CLO1313_RS07620]
regulation of sporulation	1	lcl|NC_017304.1_prot_WP_003514453.1_383[CLO1313_RS01955]
S-adenosylmethionine biosynthetic process	1	lcl|NC_017304.1_prot_WP_003518486.1_2922[CLO1313_RS14860]
spermidine transmembrane transport	1	lcl|NC_017304.1_prot_WP_003516284.1_1460[CLO1313_RS07445]
'de novo' L-methionine biosynthetic process	1	lcl|NC_017304.1_prot_WP_003517355.1_2338[CLO1313_RS11850]
DNA recombination	1	lcl|NC_017304.1_prot_WP_003513683.1_2845[CLO1313_RS14475]
carbohydrate transport	1	lcl|NC_017304.1_prot_WP_003516704.1_2782[CLO1313_RS14145]
'de novo' CTP biosynthetic process	1	lcl|NC_017304.1_prot_WP_003515563.1_1205[CLO1313_RS06160]
negative regulation of transcription, DNA-templated	1	lcl|NC_017304.1_prot_WP_011837932.1_1272[CLO1313_RS06495]

Supplemental Table S5. Protein sequences for each Down-DEGs classified as molecular function.

GO	Number of Seqs	Proteins for Gene (nucleotide) Sequences involved in GO
ATP binding	20	lcl|NC_017304.1_prot_WP_003515609.1_1180[CLO1313_RS06035],<br>lcl|NC_017304.1_prot_WP_003516284.1_1460[CLO1313_RS07445],<br>lcl|NC_017304.1_prot_WP_003511717.1_720[CLO1313_RS03730],<br>lcl|NC_017304.1_prot_WP_003519349.1_614[CLO1313_RS03160],<br>lcl|NC_017304.1_prot_WP_003513826.1_2561[CLO1313_RS12995],<br>lcl|NC_017304.1_prot_WP_003514467.1_390[CLO1313_RS01990],<br>lcl|NC_017304.1_prot_WP_003519346.1_616[CLO1313_RS03170],<br>lcl|NC_017304.1_prot_WP_003518008.1_1697[CLO1313_RS08655],<br>lcl|NC_017304.1_prot_WP_003517348.1_2342[CLO1313_RS11870],<br>lcl|NC_017304.1_prot_WP_003516702.1_2783[CLO1313_RS14150],<br>lcl|NC_017304.1_prot_WP_003518486.1_2922[CLO1313_RS14860],<br>lcl|NC_017304.1_prot_WP_003518681.1_1811[CLO1313_RS09225],<br>lcl|NC_017304.1_prot_WP_037295095.1_2558[CLO1313_RS12980],<br>lcl|NC_017304.1_prot_WP_003518357.1_120[CLO1313_RS00635],<br>lcl|NC_017304.1_prot_WP_003517368.1_2332[CLO1313_RS11820],<br>lcl|NC_017304.1_prot_WP_003517350.1_2341[CLO1313_RS11865],<br>lcl|NC_017304.1_prot_WP_003513098.1_117[CLO1313_RS00620],<br>lcl|NC_017304.1_prot_WP_003515563.1_1205[CLO1313_RS06160],<br>lcl|NC_017304.1_prot_WP_003513827.1_2562[CLO1313_RS13000],<br>lcl|NC_017304.1_prot_WP_003512581.1_1872[CLO1313_RS09525]
structural constituent of ribosome	9	lcl|NC_017304.1_prot_WP_003514304.1_316[CLO1313_RS01630],<br>lcl|NC_017304.1_prot_WP_003515019.1_311[CLO1313_RS01605],<br>lcl|NC_017304.1_prot_WP_003515021.1_309[CLO1313_RS01595],<br>lcl|NC_017304.1_prot_WP_003513685.1_2844[CLO1313_RS14470],<br>lcl|NC_017304.1_prot_WP_003513680.1_2846[CLO1313_RS14480],<br>lcl|NC_017304.1_prot_WP_003515020.1_310[CLO1313_RS01600],<br>lcl|NC_017304.1_prot_WP_003514305.1_317[CLO1313_RS01635],<br>lcl|NC_017304.1_prot_WP_003515018.1_312[CLO1313_RS01610],<br>lcl|NC_017304.1_prot_WP_003516863.1_2827[CLO1313_RS14370]
metal ion binding	7	lcl|NC_017304.1_prot_WP_003518405.1_2159[CLO1313_RS10995],<br>lcl|NC_017304.1_prot_WP_003516177.1_1515[CLO1313_RS07715],<br>lcl|NC_017304.1_prot_WP_003517357.1_2337[CLO1313_RS11845],<br>lcl|NC_017304.1_prot_WP_003513827.1_2562[CLO1313_RS13000],<br>lcl|NC_017304.1_prot_WP_003517348.1_2342[CLO1313_RS11870],<br>lcl|NC_017304.1_prot_WP_003516863.1_2827[CLO1313_RS14370],<br>lcl|NC_017304.1_prot_WP_003512583.1_1870[CLO1313_RS09515]
pyridoxal phosphate binding	7	lcl|NC_017304.1_prot_WP_003515290.1_386[CLO1313_RS01970],<br>lcl|NC_017304.1_prot_WP_003517375.1_2328[CLO1313_RS11800],<br>lcl|NC_017304.1_prot_WP_003513825.1_2560[CLO1313_RS12990],<br>lcl|NC_017304.1_prot_WP_003512623.1_1855[CLO1313_RS09440],<br>lcl|NC_017304.1_prot_WP_003517355.1_2338[CLO1313_RS11850],<br>lcl|NC_017304.1_prot_WP_003514458.1_385[CLO1313_RS01965],<br>lcl|NC_017304.1_prot_WP_003515671.1_1150[CLO1313_RS05865]

ATPase activity	6	lcl|NC_017304.1_prot_WP_003511717.1_720[CLO1313_RS03730], lcl|NC_017304.1_prot_WP_003517368.1_2332[CLO1313_RS11820], lcl|NC_017304.1_prot_WP_003517350.1_2341[CLO1313_RS11865], lcl|NC_017304.1_prot_WP_003519349.1_614[CLO1313_RS03160], lcl|NC_017304.1_prot_WP_003514467.1_390[CLO1313_RS01990], lcl|NC_017304.1_prot_WP_003516702.1_2783[CLO1313_RS14150]
rRNA binding	6	lcl|NC_017304.1_prot_WP_003514304.1_316[CLO1313_RS01630], lcl|NC_017304.1_prot_WP_003513685.1_2844[CLO1313_RS14470], lcl|NC_017304.1_prot_WP_003513680.1_2846[CLO1313_RS14480], lcl|NC_017304.1_prot_WP_003515020.1_310[CLO1313_RS01600], lcl|NC_017304.1_prot_WP_003514305.1_317[CLO1313_RS01635], lcl|NC_017304.1_prot_WP_003516863.1_2827[CLO1313_RS14370]
pyruvate synthase activity	5	lcl|NC_017304.1_prot_WP_003513282.1_20[CLO1313_RS00115], lcl|NC_017304.1_prot_WP_003516452.1_22[CLO1313_RS00125], lcl|NC_017304.1_prot_WP_003513279.1_23[CLO1313_RS00130], lcl|NC_017304.1_prot_WP_003515293.1_382[CLO1313_RS01950], lcl|NC_017304.1_prot_WP_003516455.1_21[CLO1313_RS00120]
4 iron, 4 sulfur cluster binding	5	lcl|NC_017304.1_prot_WP_003515288.1_387[CLO1313_RS01975], lcl|NC_017304.1_prot_WP_003517357.1_2337[CLO1313_RS11845], lcl|NC_017304.1_prot_WP_003517348.1_2342[CLO1313_RS11870], lcl|NC_017304.1_prot_WP_003516455.1_21[CLO1313_RS00120], lcl|NC_017304.1_prot_WP_003512583.1_1870[CLO1313_RS09515]
transferase activity	5	lcl|NC_017304.1_prot_WP_003514893.1_556[CLO1313_RS02855], lcl|NC_017304.1_prot_WP_003517444.1_1013[CLO1313_RS05175], lcl|NC_017304.1_prot_WP_003513829.1_2564[CLO1313_RS13010], lcl|NC_017304.1_prot_WP_003518405.1_2159[CLO1313_RS10995], lcl|NC_017304.1_prot_WP_003517366.1_2333[CLO1313_RS11825]
ATPase-coupled sulfate transmembrane transporter activity	4	lcl|NC_017304.1_prot_WP_003513100.1_115[CLO1313_RS00610], lcl|NC_017304.1_prot_WP_003513101.1_114[CLO1313_RS00605], lcl|NC_017304.1_prot_WP_003518358.1_116[CLO1313_RS00615], lcl|NC_017304.1_prot_WP_003513098.1_117[CLO1313_RS00620]
hydrolase activity	4	lcl|NC_017304.1_prot_WP_003516144.1_705[CLO1313_RS03655], lcl|NC_017304.1_prot_WP_003514456.1_384[CLO1313_RS01960], lcl|NC_017304.1_prot_WP_003517058.1_913[CLO1313_RS04680], lcl|NC_017304.1_prot_WP_003517355.1_2338[CLO1313_RS11850]
oxidoreductase activity	3	lcl|NC_017304.1_prot_WP_003514859.1_571[CLO1313_RS02925], lcl|NC_017304.1_prot_WP_003514857.1_572[CLO1313_RS02930], lcl|NC_017304.1_prot_WP_003514855.1_573[CLO1313_RS02935]
hydrogen dehydrogenase (NADP+) activity	3	lcl|NC_017304.1_prot_WP_003512584.1_1869[CLO1313_RS09510], lcl|NC_017304.1_prot_WP_003512582.1_1871[CLO1313_RS09520], lcl|NC_017304.1_prot_WP_003512583.1_1870[CLO1313_RS09515]
cysteine synthase activity	3	lcl|NC_017304.1_prot_WP_003521330.1_2534[CLO1313_RS12835], lcl|NC_017304.1_prot_WP_003517374.1_2329[CLO1313_RS11805], lcl|NC_017304.1_prot_WP_003517355.1_2338[CLO1313_RS11850]
magnesium ion binding	3	lcl|NC_017304.1_prot_WP_003515609.1_1180[CLO1313_RS06035], lcl|NC_017304.1_prot_WP_003516264.1_1473[CLO1313_RS07510], lcl|NC_017304.1_prot_WP_003518486.1_2922[CLO1313_RS14860]

tRNA binding	3	lcl|NC_017304.1_prot_WP_003514304.1_316[CLO1313_RS01630], lcl|NC_017304.1_prot_WP_003515020.1_310[CLO1313_RS01600], lcl|NC_017304.1_prot_WP_003514305.1_317[CLO1313_RS01635]
nitrogenase activity	3	lcl|NC_017304.1_prot_WP_003517362.1_2335[CLO1313_RS11835], lcl|NC_017304.1_prot_WP_003517348.1_2342[CLO1313_RS11870], lcl|NC_017304.1_prot_WP_003517364.1_2334[CLO1313_RS11830]
GTPase activity	3	lcl|NC_017304.1_prot_WP_003512565.1_1884[CLO1313_RS09585], lcl|NC_017304.1_prot_WP_003518357.1_120[CLO1313_RS00635], lcl|NC_017304.1_prot_WP_003514309.1_319[CLO1313_RS01645]
GTP binding	3	lcl|NC_017304.1_prot_WP_003512565.1_1884[CLO1313_RS09585], lcl|NC_017304.1_prot_WP_003518357.1_120[CLO1313_RS00635], lcl|NC_017304.1_prot_WP_003514309.1_319[CLO1313_RS01645]
carbamoyl-phosphate synthase (glutamine-hydrolyzing) activity	2	lcl|NC_017304.1_prot_WP_003513826.1_2561[CLO1313_RS12995], lcl|NC_017304.1_prot_WP_003513827.1_2562[CLO1313_RS13000]
cystathionine gamma-synthase activity	2	lcl|NC_017304.1_prot_WP_003517355.1_2338[CLO1313_RS11850], lcl|NC_017304.1_prot_WP_003514458.1_385[CLO1313_RS01965]
large ribosomal subunit rRNA binding	2	lcl|NC_017304.1_prot_WP_003515019.1_311[CLO1313_RS01605], lcl|NC_017304.1_prot_WP_003515021.1_309[CLO1313_RS01595]
iron-sulfur cluster binding	2	lcl|NC_017304.1_prot_WP_003514859.1_571[CLO1313_RS02925], lcl|NC_017304.1_prot_WP_003512584.1_1869[CLO1313_RS09510]
translation elongation factor activity	2	lcl|NC_017304.1_prot_WP_003518357.1_120[CLO1313_RS00635], lcl|NC_017304.1_prot_WP_003514309.1_319[CLO1313_RS01645]
carbonyl sulfide nitrogenase activity	2	lcl|NC_017304.1_prot_WP_003517362.1_2335[CLO1313_RS11835], lcl|NC_017304.1_prot_WP_003517348.1_2342[CLO1313_RS11870]
sulfate adenylyltransferase (ATP) activity	2	lcl|NC_017304.1_prot_WP_003518357.1_120[CLO1313_RS00635], lcl|NC_017304.1_prot_WP_003513095.1_119[CLO1313_RS00630]
methyltransferase activity	2	lcl|NC_017304.1_prot_WP_003516178.1_1514[CLO1313_RS07710], lcl|NC_017304.1_prot_WP_003515671.1_1150[CLO1313_RS05865]
lyase activity	2	lcl|NC_017304.1_prot_WP_003514859.1_571[CLO1313_RS02925], lcl|NC_017304.1_prot_WP_003514857.1_572[CLO1313_RS02930]
NADH dehydrogenase (ubiquinone) activity	2	lcl|NC_017304.1_prot_WP_003512584.1_1869[CLO1313_RS09510], lcl|NC_017304.1_prot_WP_003512583.1_1870[CLO1313_RS09515]
phosphorelay sensor kinase activity	2	lcl|NC_017304.1_prot_WP_003519346.1_616[CLO1313_RS03170], lcl|NC_017304.1_prot_WP_003518008.1_1697[CLO1313_RS08655]
thiamine pyrophosphate binding	2	lcl|NC_017304.1_prot_WP_003513279.1_23[CLO1313_RS00130], lcl|NC_017304.1_prot_WP_003515293.1_382[CLO1313_RS01950]
cystathionine beta-lyase activity	2	lcl|NC_017304.1_prot_WP_003515290.1_386[CLO1313_RS01970], lcl|NC_017304.1_prot_WP_003517375.1_2328[CLO1313_RS11800]
ornithine-oxo-acid transaminase activity	1	lcl|NC_017304.1_prot_WP_003513825.1_2560[CLO1313_RS12990]
polyamine binding	1	lcl|NC_017304.1_prot_WP_003516280.1_1463[CLO1313_RS07460]
sulfur compound binding	1	lcl|NC_017304.1_prot_WP_003513101.1_114[CLO1313_RS00605]
linear malto-oligosaccharide phosphorylase activity	1	lcl|NC_017304.1_prot_WP_003512623.1_1855[CLO1313_RS09440]

carbon-monoxide dehydrogenase (ferredoxin) activity	1	lcl|NC_017304.1_prot_WP_003515288.1_387[CLO1313_RS01975]
protein tyrosine phosphatase activity	1	lcl|NC_017304.1_prot_WP_003511903.1_2219[CLO1313_RS11280]
UMP kinase activity	1	lcl|NC_017304.1_prot_WP_003515563.1_1205[CLO1313_RS06160]
spermidine synthase activity	1	lcl|NC_017304.1_prot_WP_003516175.1_1516[CLO1313_RS07720]
glycogen phosphorylase activity	1	lcl|NC_017304.1_prot_WP_003512623.1_1855[CLO1313_RS09440]
NADP binding	1	lcl|NC_017304.1_prot_WP_003513114.1_108[CLO1313_RS00575]
L-cysteine desulfhydrase activity	1	lcl|NC_017304.1_prot_WP_003514458.1_385[CLO1313_RS01965]
ferredoxin hydrogenase activity	1	lcl|NC_017304.1_prot_WP_003512584.1_1869[CLO1313_RS09510]
SHG alpha-glucan phosphorylase activity	1	lcl|NC_017304.1_prot_WP_003512623.1_1855[CLO1313_RS09440]
3-oxoacyl-[acyl-carrier-protein] synthase activity	1	lcl|NC_017304.1_prot_WP_003512155.1_2090[CLO1313_RS10640]
pseudouridine synthase activity	1	lcl|NC_017304.1_prot_WP_003516173.1_1517[CLO1313_RS07725]
decanoate-CoA ligase activity	1	lcl|NC_017304.1_prot_WP_003512157.1_2089[CLO1313_RS10635]
kinase activity	1	lcl|NC_017304.1_prot_WP_003518357.1_120[CLO1313_RS00635]
sequence-specific DNA binding	1	lcl|NC_017304.1_prot_WP_003516286.1_1459[CLO1313_RS07440]
glycine hydroxymethyltransferase activity	1	lcl|NC_017304.1_prot_WP_003515671.1_1150[CLO1313_RS05865]
translation initiation factor activity	1	lcl|NC_017304.1_prot_WP_003520198.1_1030[CLO1313_RS05260]
ATPase-coupled transmembrane transporter activity	1	lcl|NC_017304.1_prot_WP_003518681.1_1811[CLO1313_RS09225]
beta-ketoacyl-acyl-carrier-protein synthase III activity	1	lcl|NC_017304.1_prot_WP_003512155.1_2090[CLO1313_RS10640]
homocysteine desulfhydrase activity	1	lcl|NC_017304.1_prot_WP_003514458.1_385[CLO1313_RS01965]
amino acid binding	1	lcl|NC_017304.1_prot_WP_003513828.1_2563[CLO1313_RS13005]
carboxypeptidase activity	1	lcl|NC_017304.1_prot_WP_003513087.1_123[CLO1313_RS00650]
N-acetyl-gamma-glutamyl-phosphate reductase activity	1	lcl|NC_017304.1_prot_WP_003518903.1_2557[CLO1313_RS12975]
ATPase-coupled thiosulfate transmembrane transporter activity	1	lcl|NC_017304.1_prot_WP_003513098.1_117[CLO1313_RS00620]
transaminase activity	1	lcl|NC_017304.1_prot_WP_003515290.1_386[CLO1313_RS01970]

RNA binding	1	lcl|NC_017304.1_prot_WP_003516173.1_1517[CLO1313_RS07725]
NAD+ synthase (glutamine-hydrolyzing) activity	1	lcl|NC_017304.1_prot_WP_003518917.1_2535[CLO1313_RS12840]
NAD binding	1	lcl|NC_017304.1_prot_WP_003518903.1_2557[CLO1313_RS12975]
N2-acetyl-L-ornithine:2-oxoglutarate 5-aminotransferase activity	1	lcl|NC_017304.1_prot_WP_003513825.1_2560[CLO1313_RS12990]
DNA binding	1	lcl|NC_017304.1_prot_WP_003518360.1_107[CLO1313_RS00570]
single-stranded DNA binding	1	lcl|NC_017304.1_prot_WP_003513683.1_2845[CLO1313_RS14475]
sulfuric ester hydrolase activity	1	lcl|NC_017304.1_prot_WP_003518405.1_2159[CLO1313_RS10995]
methionine gamma-lyase activity	1	lcl|NC_017304.1_prot_WP_003517355.1_2338[CLO1313_RS11850]
catalytic activity	1	lcl|NC_017304.1_prot_WP_003517357.1_2337[CLO1313_RS11845]
acetylglutamate kinase activity	1	lcl|NC_017304.1_prot_WP_037295095.1_2558[CLO1313_RS12980]
protein histidine kinase activity	1	lcl|NC_017304.1_prot_WP_003512581.1_1872[CLO1313_RS09525]
phosphoadenylyl-sulfate reductase (thioredoxin) activity	1	lcl|NC_017304.1_prot_WP_003513096.1_118[CLO1313_RS00625]
sulfurtransferase activity	1	lcl|NC_017304.1_prot_WP_003518917.1_2535[CLO1313_RS12840]
phosphate acetyltransferase activity	1	lcl|NC_017304.1_prot_WP_003521237.1_1179[CLO1313_RS06030]
holo-[acyl-carrier-protein] synthase activity	1	lcl|NC_017304.1_prot_WP_003516264.1_1473[CLO1313_RS07510]
adenosylmethionine decarboxylase activity	1	lcl|NC_017304.1_prot_WP_003516223.1_1495[CLO1313_RS07620]
molybdopterin-synthase adenylyltransferase activity	1	lcl|NC_017304.1_prot_WP_003513089.1_122[CLO1313_RS00645]
translation release factor activity, codon specific	1	lcl|NC_017304.1_prot_WP_003512565.1_1884[CLO1313_RS09585]
O-acetylhomoserine aminocarboxypropyltransferase activity	1	lcl|NC_017304.1_prot_WP_003517355.1_2338[CLO1313_RS11850]
ATPase-coupled putrescine transmembrane transporter activity	1	lcl|NC_017304.1_prot_WP_003516284.1_1460[CLO1313_RS07445]
carbon-monoxide dehydrogenase (acceptor) activity	1	lcl|NC_017304.1_prot_WP_003515288.1_387[CLO1313_RS01975]
acetate kinase activity	1	lcl|NC_017304.1_prot_WP_003515609.1_1180[CLO1313_RS06035]
serine-type endopeptidase activity	1	lcl|NC_017304.1_prot_WP_003511670.1_687[CLO1313_RS03565]

hydrolase activity, hydrolyzing O-glycosyl compounds	1	lcl|NC_017304.1_prot_WP_003511670.1_687[CLO1313_RS03565]
helicase activity	1	lcl|NC_017304.1_prot_WP_003516279.1_1464[CLO1313_RS07465]
transmembrane signaling receptor activity	1	lcl|NC_017304.1_prot_WP_003512414.1_1950[CLO1313_RS09930]
DNA-binding transcription factor activity	1	lcl|NC_017304.1_prot_WP_011837932.1_1272[CLO1313_RS06495]
carbohydrate binding	1	lcl|NC_017304.1_prot_WP_003516263.1_1474[CLO1313_RS07515]
ubiquitin-like modifier activating enzyme activity	1	lcl|NC_017304.1_prot_WP_003513089.1_122[CLO1313_RS00645]
ornithine carbamoyltransferase activity	1	lcl|NC_017304.1_prot_WP_003513828.1_2563[CLO1313_RS13005]
ATPase-coupled spermidine transmembrane transporter activity	1	lcl|NC_017304.1_prot_WP_003516284.1_1460[CLO1313_RS07445]
agmatinase activity	1	lcl|NC_017304.1_prot_WP_003516177.1_1515[CLO1313_RS07715]
arsenate reductase (glutaredoxin) activity	1	lcl|NC_017304.1_prot_WP_003511903.1_2219[CLO1313_RS11280]
glutamyl-tRNA reductase activity	1	lcl|NC_017304.1_prot_WP_003513114.1_108[CLO1313_RS00575]
fatty acid synthase activity	1	lcl|NC_017304.1_prot_WP_003512162.1_2087[CLO1313_RS10625]
protein dimerization activity	1	lcl|NC_017304.1_prot_WP_003518903.1_2557[CLO1313_RS12975]
FMN binding	1	lcl|NC_017304.1_prot_WP_003512583.1_1870[CLO1313_RS09515]
long-chain fatty acid-CoA ligase activity	1	lcl|NC_017304.1_prot_WP_003512157.1_2089[CLO1313_RS10635]
iron ion binding	1	lcl|NC_017304.1_prot_WP_003512584.1_1869[CLO1313_RS09510]
electron transfer activity	1	lcl|NC_017304.1_prot_WP_003512584.1_1869[CLO1313_RS09510]
alanine-glyoxylate transaminase activity	1	lcl|NC_017304.1_prot_WP_003512411.1_1951[CLO1313_RS09935]
L-cystine L-cysteine-lyase (deaminating)	1	lcl|NC_017304.1_prot_WP_003514458.1_385[CLO1313_RS01965]
hydrolase activity, acting on ester bonds	1	lcl|NC_017304.1_prot_WP_003513453.1_2973[CLO1313_RS15115]
cystathionine gamma-lyase activity	1	lcl|NC_017304.1_prot_WP_003514458.1_385[CLO1313_RS01965]
nickel cation binding	1	lcl|NC_017304.1_prot_WP_003515288.1_387[CLO1313_RS01975]
oxidoreductase activity, acting on the CH-OH group of donors, NAD or NADP as acceptor	1	lcl|NC_017304.1_prot_WP_003512162.1_2087[CLO1313_RS10625]
methionine adenosyltransferase activity	1	lcl|NC_017304.1_prot_WP_003518486.1_2922[CLO1313_RS14860]

Supplemental Table S6. Protein sequences for each Down-DEGs classified as celular component.

GO	Number of Seqs	Proteins for Gene (nucleotide) Sequences involved in GO
integral component of membrane	33	lcl|NC_017304.1_prot_WP_003513277.1_25[CLO1313_RS00135], lcl|NC_017304.1_prot_WP_003516702.1_2783[CLO1313_RS14150], lcl|NC_017304.1_prot_WP_003513100.1_115[CLO1313_RS00610], lcl|NC_017304.1_prot_WP_003512414.1_1950[CLO1313_RS09930], lcl|NC_017304.1_prot_WP_003517461.1_1004[CLO1313_RS05130], lcl|NC_017304.1_prot_WP_003511526.1_615[CLO1313_RS03165], lcl|NC_017304.1_prot_WP_003513089.1_122[CLO1313_RS00645], lcl|NC_017304.1_prot_WP_003514855.1_573[CLO1313_RS02935], lcl|NC_017304.1_prot_WP_003517056.1_914[CLO1313_RS04685], lcl|NC_017304.1_prot_WP_003516263.1_1474[CLO1313_RS07515], lcl|NC_017304.1_prot_WP_003513496.1_2949[CLO1313_RS14995], lcl|NC_017304.1_prot_WP_003517372.1_2330[CLO1313_RS11810], lcl|NC_017304.1_prot_WP_003513552.1_2921[CLO1313_RS14855], lcl|NC_017304.1_prot_WP_003517114.1_888[CLO1313_RS04555], lcl|NC_017304.1_prot_WP_003518358.1_116[CLO1313_RS00615], lcl|NC_017304.1_prot_WP_003514857.1_572[CLO1313_RS02930], lcl|NC_017304.1_prot_WP_003516145.1_704[CLO1313_RS03650], lcl|NC_017304.1_prot_WP_003518405.1_2159[CLO1313_RS10995], lcl|NC_017304.1_prot_WP_003514372.1_338[CLO1313_RS01740], lcl|NC_017304.1_prot_WP_003519346.1_616[CLO1313_RS03170], lcl|NC_017304.1_prot_WP_003517046.1_916[CLO1313_RS04695], lcl|NC_017304.1_prot_WP_003516704.1_2782[CLO1313_RS14145], lcl|NC_017304.1_prot_WP_003514465.1_389[CLO1313_RS01985], lcl|NC_017304.1_prot_WP_003516281.1_1462[CLO1313_RS07455], lcl|NC_017304.1_prot_WP_003518681.1_1811[CLO1313_RS09225], lcl|NC_017304.1_prot_WP_003513101.1_114[CLO1313_RS00605], lcl|NC_017304.1_prot_WP_003520010.1_768[CLO1313_RS03970], lcl|NC_017304.1_prot_WP_003517055.1_915[CLO1313_RS04690], lcl|NC_017304.1_prot_WP_003517043.1_919[CLO1313_RS04710], lcl|NC_017304.1_prot_WP_003517352.1_2340[CLO1313_RS11860], lcl|NC_017304.1_prot_WP_003516282.1_1461[CLO1313_RS07450], lcl|NC_017304.1_prot_WP_003517370.1_2331[CLO1313_RS11815], lcl|NC_017304.1_prot_WP_003516128.1_721[CLO1313_RS03735]
cytoplasm	15	lcl|NC_017304.1_prot_WP_003516175.1_1516[CLO1313_RS07720], lcl|NC_017304.1_prot_WP_003515609.1_1180[CLO1313_RS06035], lcl|NC_017304.1_prot_WP_003520198.1_1030[CLO1313_RS05260], lcl|NC_017304.1_prot_WP_003521237.1_1179[CLO1313_RS06030], lcl|NC_017304.1_prot_WP_003513828.1_2563[CLO1313_RS13005], lcl|NC_017304.1_prot_WP_003518903.1_2557[CLO1313_RS12975], lcl|NC_017304.1_prot_WP_003515671.1_1150[CLO1313_RS05865], lcl|NC_017304.1_prot_WP_003518486.1_2922[CLO1313_RS14860], lcl|NC_017304.1_prot_WP_003512565.1_1884[CLO1313_RS09585], lcl|NC_017304.1_prot_WP_003513096.1_118[CLO1313_RS00625],

		lcl|NC_017304.1_prot_WP_037295095.1_2558[CLO1313_RS12980], lcl|NC_017304.1_prot_WP_003513825.1_2560[CLO1313_RS12990], lcl|NC_017304.1_prot_WP_003514309.1_319[CLO1313_RS01645], lcl|NC_017304.1_prot_WP_003515563.1_1205[CLO1313_RS06160], lcl|NC_017304.1_prot_WP_003515546.1_1214[CLO1313_RS06205]
ribosome	8	lcl|NC_017304.1_prot_WP_003515019.1_311[CLO1313_RS01605], lcl|NC_017304.1_prot_WP_003512202.1_2062[CLO1313_RS10500], lcl|NC_017304.1_prot_WP_003515021.1_309[CLO1313_RS01595], lcl|NC_017304.1_prot_WP_003513685.1_2844[CLO1313_RS14470], lcl|NC_017304.1_prot_WP_003513680.1_2846[CLO1313_RS14480], lcl|NC_017304.1_prot_WP_003513829.1_2564[CLO1313_RS13010], lcl|NC_017304.1_prot_WP_003515018.1_312[CLO1313_RS01610], lcl|NC_017304.1_prot_WP_003516863.1_2827[CLO1313_RS14370]
plasma membrane	5	lcl|NC_017304.1_prot_WP_003517352.1_2340[CLO1313_RS11860], lcl|NC_017304.1_prot_WP_003516704.1_2782[CLO1313_RS14145], lcl|NC_017304.1_prot_WP_003516282.1_1461[CLO1313_RS07450], lcl|NC_017304.1_prot_WP_003514465.1_389[CLO1313_RS01985], lcl|NC_017304.1_prot_WP_003516281.1_1462[CLO1313_RS07455]
ATP-binding cassette (ABC) transporter complex	2	lcl|NC_017304.1_prot_WP_003516284.1_1460[CLO1313_RS07445], lcl|NC_017304.1_prot_WP_003513098.1_117[CLO1313_RS00620]
small ribosomal subunit	2	lcl|NC_017304.1_prot_WP_003514304.1_316[CLO1313_RS01630], lcl|NC_017304.1_prot_WP_003514305.1_317[CLO1313_RS01635]
large ribosomal subunit	1	lcl|NC_017304.1_prot_WP_003515020.1_310[CLO1313_RS01600]
periplasmic space	1	lcl|NC_017304.1_prot_WP_003516280.1_1463[CLO1313_RS07460]
membrane	1	lcl|NC_017304.1_prot_WP_003512584.1_1869[CLO1313_RS09510]
molybdenum-iron nitrogenase complex	1	lcl|NC_017304.1_prot_WP_003517348.1_2342[CLO1313_RS11870]

Supplemental Table S7. Protein sequences for each up-DEGs classified as biological processes.

GO	Number of Seqs	Proteins for Gene (nucleotide) Sequences involved in GO
chemotaxis	23	lcl|NC_017304.1_prot_WP_003518714.1_1399[CLO1313_RS07140], lcl|NC_017304.1_prot_WP_014522631.1_2185[CLO1313_RS11120], lcl|NC_017304.1_prot_WP_003514493.1_401[CLO1313_RS02045], lcl|NC_017304.1_prot_WP_003517776.1_2259[CLO1313_RS11480], lcl|NC_017304.1_prot_WP_003518813.1_403[CLO1313_RS02055], lcl|NC_017304.1_prot_WP_003514847.1_577[CLO1313_RS02955], lcl|NC_017304.1_prot_WP_003518455.1_2957[CLO1313_RS15035], lcl|NC_017304.1_prot_WP_003517991.1_1713[CLO1313_RS08735], lcl|NC_017304.1_prot_WP_003518456.1_2956[CLO1313_RS15030], lcl|NC_017304.1_prot_WP_003518709.1_1402[CLO1313_RS07155], lcl|NC_017304.1_prot_WP_003517989.1_1715[CLO1313_RS08745], lcl|NC_017304.1_prot_WP_003517972.1_1728[CLO1313_RS08810], lcl|NC_017304.1_prot_WP_003519601.1_2184[CLO1313_RS11115], lcl|NC_017304.1_prot_WP_003517988.1_1716[CLO1313_RS08750], lcl|NC_017304.1_prot_WP_003517969.1_1730[CLO1313_RS08820], lcl|NC_017304.1_prot_WP_080552882.1_2183[CLO1313_RS11110], lcl|NC_017304.1_prot_WP_003516942.1_530[CLO1313_RS02725], lcl|NC_017304.1_prot_WP_003518814.1_402[CLO1313_RS02050], lcl|NC_017304.1_prot_WP_003518963.1_2186[CLO1313_RS11125], lcl|NC_017304.1_prot_WP_003516902.1_584[CLO1313_RS02990], lcl|NC_017304.1_prot_WP_003517987.1_1717[CLO1313_RS08755], lcl|NC_017304.1_prot_WP_003518457.1_2955[CLO1313_RS15025], lcl|NC_017304.1_prot_WP_003518712.1_1400[CLO1313_RS07145]
phosphorelay signal transduction system	18	lcl|NC_017304.1_prot_WP_003515795.1_2498[CLO1313_RS12655], lcl|NC_017304.1_prot_WP_003518714.1_1399[CLO1313_RS07140], lcl|NC_017304.1_prot_WP_003518707.1_1403[CLO1313_RS07160], lcl|NC_017304.1_prot_WP_003518716.1_1398[CLO1313_RS07135], lcl|NC_017304.1_prot_WP_003518706.1_1404[CLO1313_RS07165], lcl|NC_017304.1_prot_WP_003512286.1_2020[CLO1313_RS10280], lcl|NC_017304.1_prot_WP_003517974.1_1727[CLO1313_RS08805], lcl|NC_017304.1_prot_WP_003518456.1_2956[CLO1313_RS15030], lcl|NC_017304.1_prot_WP_003518225.1_870[CLO1313_RS04465], lcl|NC_017304.1_prot_WP_003518709.1_1402[CLO1313_RS07155], lcl|NC_017304.1_prot_WP_003517496.1_988[CLO1313_RS05055], lcl|NC_017304.1_prot_WP_003517988.1_1716[CLO1313_RS08750], lcl|NC_017304.1_prot_WP_003516246.1_1485[CLO1313_RS07570], lcl|NC_017304.1_prot_WP_003515794.1_2499[CLO1313_RS12660], lcl|NC_017304.1_prot_WP_003512366.1_1979[CLO1313_RS15640], lcl|NC_017304.1_prot_WP_003518814.1_402[CLO1313_RS02050], lcl|NC_017304.1_prot_WP_003517987.1_1717[CLO1313_RS08755], lcl|NC_017304.1_prot_WP_003517495.1_989[CLO1313_RS05060]
signal transduction	16	lcl|NC_017304.1_prot_WP_014522631.1_2185[CLO1313_RS11120], lcl|NC_017304.1_prot_WP_003514493.1_401[CLO1313_RS02045], lcl|NC_017304.1_prot_WP_003517776.1_2259[CLO1313_RS11480],

		lcl|NC_017304.1_prot_WP_003518813.1_403[CLO1313_RS02055], lcl|NC_017304.1_prot_WP_003514847.1_577[CLO1313_RS02955], lcl|NC_017304.1_prot_WP_003518455.1_2957[CLO1313_RS15035], lcl|NC_017304.1_prot_WP_003517989.1_1715[CLO1313_RS08745], lcl|NC_017304.1_prot_WP_003519601.1_2184[CLO1313_RS11115], lcl|NC_017304.1_prot_WP_080552882.1_2183[CLO1313_RS11110], lcl|NC_017304.1_prot_WP_003516942.1_530[CLO1313_RS02725], lcl|NC_017304.1_prot_WP_003518963.1_2186[CLO1313_RS11125], lcl|NC_017304.1_prot_WP_003516902.1_584[CLO1313_RS02990], lcl|NC_017304.1_prot_WP_003519116.1_1021[CLO1313_RS05215], lcl|NC_017304.1_prot_WP_003518457.1_2955[CLO1313_RS15025], lcl|NC_017304.1_prot_WP_003516356.1_1428[CLO1313_RS07285], lcl|NC_017304.1_prot_WP_003518712.1_1400[CLO1313_RS07145]
bacterial-type flagellum-dependent cell motility	12	lcl|NC_017304.1_prot_WP_003512930.1_208[CLO1313_RS01070], lcl|NC_017304.1_prot_WP_003517971.1_1729[CLO1313_RS08815], lcl|NC_017304.1_prot_WP_003518494.1_2914[CLO1313_RS14820], lcl|NC_017304.1_prot_WP_003517972.1_1728[CLO1313_RS08810], lcl|NC_017304.1_prot_WP_003513572.1_2913[CLO1313_RS14815], lcl|NC_017304.1_prot_WP_003512932.1_207[CLO1313_RS01065], lcl|NC_017304.1_prot_WP_003517965.1_1732[CLO1313_RS08830], lcl|NC_017304.1_prot_WP_003518492.1_2915[CLO1313_RS14825], lcl|NC_017304.1_prot_WP_003513581.1_2908[CLO1313_RS14790], lcl|NC_017304.1_prot_WP_080547380.1_2298[CLO1313_RS15685], lcl|NC_017304.1_prot_WP_003517969.1_1730[CLO1313_RS08820], lcl|NC_017304.1_prot_WP_003513583.1_2907[CLO1313_RS14785]
polysaccharide catabolic process	11	lcl|NC_017304.1_prot_WP_003513507.1_2942[CLO1313_RS14960], lcl|NC_017304.1_prot_WP_003513657.1_2857[CLO1313_RS14535], lcl|NC_017304.1_prot_WP_003519011.1_1977[CLO1313_RS10065], lcl|NC_017304.1_prot_WP_003519375.1_627[CLO1313_RS03220], lcl|NC_017304.1_prot_WP_003522420.1_2566[CLO1313_RS13020], lcl|NC_017304.1_prot_WP_003516854.1_2837[CLO1313_RS14425], lcl|NC_017304.1_prot_WP_003513660.1_2856[CLO1313_RS14530], lcl|NC_017304.1_prot_WP_003515281.1_397[CLO1313_RS02025], lcl|NC_017304.1_prot_WP_014522595.1_626[CLO1313_RS15440], lcl|NC_017304.1_prot_WP_003516694.1_2791[CLO1313_RS14190], lcl|NC_017304.1_prot_WP_003516835.1_2855[CLO1313_RS14525]
transmembrane transport	10	lcl|NC_017304.1_prot_WP_037294696.1_510[CLO1313_RS02625], lcl|NC_017304.1_prot_WP_003517330.1_2350[CLO1313_RS11910], lcl|NC_017304.1_prot_WP_003515800.1_2495[CLO1313_RS12640], lcl|NC_017304.1_prot_WP_003517320.1_2355[CLO1313_RS11935], lcl|NC_017304.1_prot_WP_003517459.1_1005[CLO1313_RS05135], lcl|NC_017304.1_prot_WP_003513175.1_78[CLO1313_RS00410], lcl|NC_017304.1_prot_WP_003521788.1_2349[CLO1313_RS11905], lcl|NC_017304.1_prot_WP_003514728.1_507[CLO1313_RS02610], lcl|NC_017304.1_prot_WP_003517571.1_1688[CLO1313_RS08605], lcl|NC_017304.1_prot_WP_003515799.1_2496[CLO1313_RS12645]
bacterial-type flagellum assembly	8	lcl|NC_017304.1_prot_WP_003518494.1_2914[CLO1313_RS14820], lcl|NC_017304.1_prot_WP_003513620.1_2888[CLO1313_RS14690],

		lcl|NC_017304.1_prot_WP_003517983.1_1721[CLO1313_RS08775], lcl|NC_017304.1_prot_WP_003518492.1_2915[CLO1313_RS14825], lcl|NC_017304.1_prot_WP_003513622.1_2887[CLO1313_RS14685], lcl|NC_017304.1_prot_WP_003517979.1_1724[CLO1313_RS08790], lcl|NC_017304.1_prot_WP_003513566.1_2916[CLO1313_RS14830], lcl|NC_017304.1_prot_WP_003517982.1_1722[CLO1313_RS08780]
signal transduction by protein phosphorylation	7	lcl|NC_017304.1_prot_WP_003518714.1_1399[CLO1313_RS07140], lcl|NC_017304.1_prot_WP_003518707.1_1403[CLO1313_RS07160], lcl|NC_017304.1_prot_WP_003518456.1_2956[CLO1313_RS15030], lcl|NC_017304.1_prot_WP_003518814.1_402[CLO1313_RS02050], lcl|NC_017304.1_prot_WP_003517496.1_988[CLO1313_RS05055], lcl|NC_017304.1_prot_WP_003517988.1_1716[CLO1313_RS08750], lcl|NC_017304.1_prot_WP_003515794.1_2499[CLO1313_RS12660]
oxidation-reduction process	7	lcl|NC_017304.1_prot_WP_003512275.1_2026[CLO1313_RS10310], lcl|NC_017304.1_prot_WP_003512660.1_1834[CLO1313_RS09335], lcl|NC_017304.1_prot_WP_003520287.1_788[CLO1313_RS04065], lcl|NC_017304.1_prot_WP_003518076.1_2025[CLO1313_RS10305], lcl|NC_017304.1_prot_WP_003513182.1_75[CLO1313_RS00395], lcl|NC_017304.1_prot_WP_003512281.1_2023[CLO1313_RS10295], lcl|NC_017304.1_prot_WP_003517337.1_2347[CLO1313_RS11895]
peptidyl-histidine phosphorylation	6	lcl|NC_017304.1_prot_WP_003518714.1_1399[CLO1313_RS07140], lcl|NC_017304.1_prot_WP_003518707.1_1403[CLO1313_RS07160], lcl|NC_017304.1_prot_WP_003518456.1_2956[CLO1313_RS15030], lcl|NC_017304.1_prot_WP_003518814.1_402[CLO1313_RS02050], lcl|NC_017304.1_prot_WP_003517496.1_988[CLO1313_RS05055], lcl|NC_017304.1_prot_WP_003517988.1_1716[CLO1313_RS08750]
regulation of transcription, DNA-templated	6	lcl|NC_017304.1_prot_WP_003519202.1_804[CLO1313_RS04140], lcl|NC_017304.1_prot_WP_003515795.1_2498[CLO1313_RS12655], lcl|NC_017304.1_prot_WP_003518707.1_1403[CLO1313_RS07160], lcl|NC_017304.1_prot_WP_003512366.1_1979[CLO1313_RS15640], lcl|NC_017304.1_prot_WP_003512374.1_1971[CLO1313_RS10035], lcl|NC_017304.1_prot_WP_003517495.1_989[CLO1313_RS05060]
cellulose catabolic process	5	lcl|NC_017304.1_prot_WP_003519374.1_628[CLO1313_RS03225], lcl|NC_017304.1_prot_WP_003518381.1_2179[CLO1313_RS11090], lcl|NC_017304.1_prot_WP_003519027.1_1795[CLO1313_RS09145], lcl|NC_017304.1_prot_WP_003517480.1_1000[CLO1313_RS05115], lcl|NC_017304.1_prot_WP_003516749.1_2744[CLO1313_RS13955]
regulation of DNA-templated transcription, initiation	4	lcl|NC_017304.1_prot_WP_003517993.1_1711[CLO1313_RS08725], lcl|NC_017304.1_prot_WP_003515819.1_2483[CLO1313_RS12580], lcl|NC_017304.1_prot_WP_003517500.1_984[CLO1313_RS05035], lcl|NC_017304.1_prot_WP_003513078.1_127[CLO1313_RS00670]
methylation	4	lcl|NC_017304.1_prot_WP_003511692.1_710[CLO1313_RS03680], lcl|NC_017304.1_prot_WP_003517259.1_1308[CLO1313_RS06675], lcl|NC_017304.1_prot_WP_003513592.1_2902[CLO1313_RS14760], lcl|NC_017304.1_prot_WP_003516030.1_2390[CLO1313_RS12105]
carbohydrate metabolic process	4	lcl|NC_017304.1_prot_WP_003517483.1_999[CLO1313_RS05110], lcl|NC_017304.1_prot_WP_014522588.1_90[CLO1313_RS15490],

		lcl|NC_017304.1_prot_WP_003520682.1_2383[CLO1313_RS12070], lcl|NC_017304.1_prot_WP_003517828.1_2232[CLO1313_RS11345]
biosynthetic process	4	lcl|NC_017304.1_prot_WP_003520835.1_378[CLO1313_RS01930], lcl|NC_017304.1_prot_WP_003513023.1_148[CLO1313_RS00770], lcl|NC_017304.1_prot_WP_003517625.1_1663[CLO1313_RS08480], lcl|NC_017304.1_prot_WP_003516296.1_1455[CLO1313_RS07420]
glutamine metabolic process	3	lcl|NC_017304.1_prot_WP_003512275.1_2026[CLO1313_RS10310], lcl|NC_017304.1_prot_WP_003517621.1_1665[CLO1313_RS08490], lcl|NC_017304.1_prot_WP_003517617.1_1667[CLO1313_RS08500]
intein-mediated protein splicing	3	lcl|NC_017304.1_prot_WP_014522651.1_2668[CLO1313_RS13550], lcl|NC_017304.1_prot_WP_003516020.1_2399[CLO1313_RS12150], lcl|NC_017304.1_prot_WP_014522653.1_2674[CLO1313_RS13580]
phosphorylation	3	lcl|NC_017304.1_prot_WP_003518716.1_1398[CLO1313_RS07135], lcl|NC_017304.1_prot_WP_003518706.1_1404[CLO1313_RS07165], lcl|NC_017304.1_prot_WP_003516413.1_72[CLO1313_RS00380]
proteolysis	3	lcl|NC_017304.1_prot_WP_003512399.1_1956[CLO1313_RS09960], lcl|NC_017304.1_prot_WP_003513147.1_91[CLO1313_RS00490], lcl|NC_017304.1_prot_WP_003517571.1_1688[CLO1313_RS08605]
protein secretion	3	lcl|NC_017304.1_prot_WP_003517983.1_1721[CLO1313_RS08775], lcl|NC_017304.1_prot_WP_003517979.1_1724[CLO1313_RS08790], lcl|NC_017304.1_prot_WP_003517982.1_1722[CLO1313_RS08780]
bacterial-type flagellum organization	3	lcl|NC_017304.1_prot_WP_003517961.1_1734[CLO1313_RS08840], lcl|NC_017304.1_prot_WP_003517984.1_1720[CLO1313_RS08770], lcl|NC_017304.1_prot_WP_003517976.1_1726[CLO1313_RS08800]
protein methylation	3	lcl|NC_017304.1_prot_WP_003518711.1_1401[CLO1313_RS07150], lcl|NC_017304.1_prot_WP_003514500.1_404[CLO1313_RS02060], lcl|NC_017304.1_prot_WP_003518458.1_2954[CLO1313_RS15020]
cation transmembrane transport	3	lcl|NC_017304.1_prot_WP_003519012.1_1972[CLO1313_RS10040], lcl|NC_017304.1_prot_WP_003517318.1_2356[CLO1313_RS11940], lcl|NC_017304.1_prot_WP_003515803.1_2493[CLO1313_RS12630]
peptidoglycan catabolic process	2	lcl|NC_017304.1_prot_WP_003515588.1_1192[CLO1313_RS06095], lcl|NC_017304.1_prot_WP_003515313.1_367[CLO1313_RS01880]
nitrogen compound metabolic process	2	lcl|NC_017304.1_prot_WP_003512274.1_2027[CLO1313_RS10315], lcl|NC_017304.1_prot_WP_003517405.1_2305[CLO1313_RS11700]
urea catabolic process	2	lcl|NC_017304.1_prot_WP_003515807.1_2491[CLO1313_RS12620], lcl|NC_017304.1_prot_WP_003515809.1_2490[CLO1313_RS12615]
protein demethylation	2	lcl|NC_017304.1_prot_WP_003518709.1_1402[CLO1313_RS07155], lcl|NC_017304.1_prot_WP_003517987.1_1717[CLO1313_RS08755]
peptide transport	2	lcl|NC_017304.1_prot_WP_003519421.1_509[CLO1313_RS02620], lcl|NC_017304.1_prot_WP_003514730.1_508[CLO1313_RS02615]
nucleic acid phosphodiester bond hydrolysis	2	lcl|NC_017304.1_prot_WP_003516053.1_764[CLO1313_RS03955], lcl|NC_017304.1_prot_WP_003516020.1_2399[CLO1313_RS12150]
lysine biosynthetic process via diaminopimelate	2	lcl|NC_017304.1_prot_WP_003511603.1_649[CLO1313_RS03355], lcl|NC_017304.1_prot_WP_003516678.1_1526[CLO1313_RS07770]

iron ion transmembrane transport	2	lcl|NC_017304.1_prot_WP_003520743.1_844[CLO1313_RS04340], lcl|NC_017304.1_prot_WP_014522619.1_1594[CLO1313_RS08125]
thiamine biosynthetic process	2	lcl|NC_017304.1_prot_WP_003516495.1_1615[CLO1313_RS08225], lcl|NC_017304.1_prot_WP_003516497.1_1614[CLO1313_RS08220]
protein deamination	2	lcl|NC_017304.1_prot_WP_003518709.1_1402[CLO1313_RS07155], lcl|NC_017304.1_prot_WP_003517987.1_1717[CLO1313_RS08755]
divalent inorganic cation transport	2	lcl|NC_017304.1_prot_WP_003520743.1_844[CLO1313_RS04340], lcl|NC_017304.1_prot_WP_014522619.1_1594[CLO1313_RS08125]
negative regulation of transcription, DNA-templated	2	lcl|NC_017304.1_prot_WP_003513565.1_2917[CLO1313_RS14835], lcl|NC_017304.1_prot_WP_003517022.1_930[CLO1313_RS04765]
iron ion homeostasis	2	lcl|NC_017304.1_prot_WP_003520743.1_844[CLO1313_RS04340], lcl|NC_017304.1_prot_WP_014522619.1_1594[CLO1313_RS08125]
long-chain fatty acid metabolic process	1	lcl|NC_017304.1_prot_WP_003520602.1_1982[CLO1313_RS10090]
cellular amino acid metabolic process	1	lcl|NC_017304.1_prot_WP_003512660.1_1834[CLO1313_RS09335]
monosaccharide transmembrane transport	1	lcl|NC_017304.1_prot_WP_003517334.1_2348[CLO1313_RS11900]
rRNA processing	1	lcl|NC_017304.1_prot_WP_003511692.1_710[CLO1313_RS03680]
cell redox homeostasis	1	lcl|NC_017304.1_prot_WP_003512363.1_1981[CLO1313_RS10085]
defense response to virus	1	lcl|NC_017304.1_prot_WP_003516053.1_764[CLO1313_RS03955]
pectin catabolic process	1	lcl|NC_017304.1_prot_WP_003518514.1_495[CLO1313_RS02535]
bacteriocin transport	1	lcl|NC_017304.1_prot_WP_003517571.1_1688[CLO1313_RS08605]
lysine biosynthetic process via diaminopimelate, diaminopimelate-aminotransferase pathway	1	lcl|NC_017304.1_prot_WP_003511604.1_650[CLO1313_RS03360]
cell wall modification	1	lcl|NC_017304.1_prot_WP_003518514.1_495[CLO1313_RS02535]
asparagine biosynthetic process	1	lcl|NC_017304.1_prot_WP_003517621.1_1665[CLO1313_RS08490]
amino acid transport	1	lcl|NC_017304.1_prot_WP_003515797.1_2497[CLO1313_RS12650]
iron import into cell	1	lcl|NC_017304.1_prot_WP_003517318.1_2356[CLO1313_RS11940]
regulation of catalytic activity	1	lcl|NC_017304.1_prot_WP_003517774.1_2260[CLO1313_RS11485]
glycerol metabolic process	1	lcl|NC_017304.1_prot_WP_003516413.1_72[CLO1313_RS00380]
organic phosphonate transport	1	lcl|NC_017304.1_prot_WP_003515802.1_2494[CLO1313_RS12635]
glutamine biosynthetic process	1	lcl|NC_017304.1_prot_WP_003512284.1_2021[CLO1313_RS10285]

thiamine diphosphate biosynthetic process	1	lcl|NC_017304.1_prot_WP_003516495.1_1615[CLO1313_RS08225]
SRP-dependent cotranslational protein targeting to membrane	1	lcl|NC_017304.1_prot_WP_003517984.1_1720[CLO1313_RS08770]
organophosphate ester transport	1	lcl|NC_017304.1_prot_WP_003515802.1_2494[CLO1313_RS12635]
cellular metabolic process	1	lcl|NC_017304.1_prot_WP_003522420.1_2566[CLO1313_RS13020]
leucine biosynthetic process	1	lcl|NC_017304.1_prot_WP_003518251.1_856[CLO1313_RS04395]
glycerol-3-phosphate transmembrane transport	1	lcl|NC_017304.1_prot_WP_003517318.1_2356[CLO1313_RS11940]
pantothenate biosynthetic process	1	lcl|NC_017304.1_prot_WP_003517259.1_1308[CLO1313_RS06675]
NAD biosynthetic process	1	lcl|NC_017304.1_prot_WP_003520327.1_1888[CLO1313_RS09620]
regulation of nitrogen utilization	1	lcl|NC_017304.1_prot_WP_003517774.1_2260[CLO1313_RS11485]
DNA methylation	1	lcl|NC_017304.1_prot_WP_003517294.1_2368[CLO1313_RS11995]
cadmium ion transmembrane transport	1	lcl|NC_017304.1_prot_WP_003519012.1_1972[CLO1313_RS10040]
electron transport chain	1	lcl|NC_017304.1_prot_WP_003512363.1_1981[CLO1313_RS10085]
aminoacyl-tRNA metabolism involved in translational fidelity	1	lcl|NC_017304.1_prot_WP_003519337.1_1889[CLO1313_RS09625]
anion transmembrane transport	1	lcl|NC_017304.1_prot_WP_003515802.1_2494[CLO1313_RS12635]
transcription initiation from bacterial-type RNA polymerase promoter	1	lcl|NC_017304.1_prot_WP_003517500.1_984[CLO1313_RS05035]
protein phosphorylation	1	lcl|NC_017304.1_prot_WP_003518726.1_1392[CLO1313_RS07105]
glutamate biosynthetic process	1	lcl|NC_017304.1_prot_WP_003518076.1_2025[CLO1313_RS10305]
carbohydrate transport	1	lcl|NC_017304.1_prot_WP_003516409.1_77[CLO1313_RS00405]
valyl-tRNA aminoacylation	1	lcl|NC_017304.1_prot_WP_003519337.1_1889[CLO1313_RS09625]
ammonium transmembrane transport	1	lcl|NC_017304.1_prot_WP_003517774.1_2260[CLO1313_RS11485]
polyamine transmembrane transport	1	lcl|NC_017304.1_prot_WP_003517318.1_2356[CLO1313_RS11940]

peptidoglycan metabolic process	1	lcl|NC_017304.1_prot_WP_003514333.1_333[CLO1313_RS01715]
xylan catabolic process	1	lcl|NC_017304.1_prot_WP_003517623.1_1664[CLO1313_RS08485]
DNA topological change	1	lcl|NC_017304.1_prot_WP_003513382.1_3010[CLO1313_RS15305]

Supplemental Table S8. Protein sequences for each up-DEGs classified as molecular function.

GO	Number of Seqs	Proteins for Gene (nucleotide) Sequences involved in GO
ATP binding	23	lcl|NC_017304.1_prot_WP_003519421.1_509[CLO1313_RS02620], lcl|NC_017304.1_prot_WP_003518714.1_1399[CLO1313_RS07140], lcl|NC_017304.1_prot_WP_003518707.1_1403[CLO1313_RS07160], lcl|NC_017304.1_prot_WP_003517621.1_1665[CLO1313_RS08490], lcl|NC_017304.1_prot_WP_003519337.1_1889[CLO1313_RS09625], lcl|NC_017304.1_prot_WP_003518456.1_2956[CLO1313_RS15030], lcl|NC_017304.1_prot_WP_003517318.1_2356[CLO1313_RS11940], lcl|NC_017304.1_prot_WP_003514730.1_508[CLO1313_RS02615], lcl|NC_017304.1_prot_WP_003518726.1_1392[CLO1313_RS07105], lcl|NC_017304.1_prot_WP_003517625.1_1663[CLO1313_RS08480], lcl|NC_017304.1_prot_WP_003517985.1_1719[CLO1313_RS08765], lcl|NC_017304.1_prot_WP_003517496.1_988[CLO1313_RS05055], lcl|NC_017304.1_prot_WP_003517988.1_1716[CLO1313_RS08750], lcl|NC_017304.1_prot_WP_003515802.1_2494[CLO1313_RS12635], lcl|NC_017304.1_prot_WP_003519012.1_1972[CLO1313_RS10040], lcl|NC_017304.1_prot_WP_003517334.1_2348[CLO1313_RS11900], lcl|NC_017304.1_prot_WP_003518165.1_1959[CLO1313_RS09975], lcl|NC_017304.1_prot_WP_003520327.1_1888[CLO1313_RS09620], lcl|NC_017304.1_prot_WP_003518814.1_402[CLO1313_RS02050], lcl|NC_017304.1_prot_WP_003511964.1_2180[CLO1313_RS11095], lcl|NC_017304.1_prot_WP_003515803.1_2493[CLO1313_RS12630], lcl|NC_017304.1_prot_WP_003516409.1_77[CLO1313_RS00405], lcl|NC_017304.1_prot_WP_003517571.1_1688[CLO1313_RS08605]
DNA binding	13	lcl|NC_017304.1_prot_WP_003518716.1_1398[CLO1313_RS07135], lcl|NC_017304.1_prot_WP_003518706.1_1404[CLO1313_RS07165], lcl|NC_017304.1_prot_WP_003517974.1_1727[CLO1313_RS08805], lcl|NC_017304.1_prot_WP_003516233.1_1491[CLO1313_RS07600], lcl|NC_017304.1_prot_WP_003519202.1_804[CLO1313_RS04140], lcl|NC_017304.1_prot_WP_003517993.1_1711[CLO1313_RS08725], lcl|NC_017304.1_prot_WP_003512366.1_1979[CLO1313_RS15640], lcl|NC_017304.1_prot_WP_003515819.1_2483[CLO1313_RS12580], lcl|NC_017304.1_prot_WP_003517500.1_984[CLO1313_RS05035], lcl|NC_017304.1_prot_WP_003517294.1_2368[CLO1313_RS11995], lcl|NC_017304.1_prot_WP_003517495.1_989[CLO1313_RS05060], lcl|NC_017304.1_prot_WP_003513078.1_127[CLO1313_RS00670], lcl|NC_017304.1_prot_WP_003517022.1_930[CLO1313_RS04765]
hydrolase activity	9	lcl|NC_017304.1_prot_WP_014522596.1_677[CLO1313_RS03515], lcl|NC_017304.1_prot_WP_003519434.1_1070[CLO1313_RS05460], lcl|NC_017304.1_prot_WP_003513438.1_2978[CLO1313_RS15140], lcl|NC_017304.1_prot_WP_003517994.1_1710[CLO1313_RS08720], lcl|NC_017304.1_prot_WP_003512336.1_1996[CLO1313_RS10155], lcl|NC_017304.1_prot_WP_003511964.1_2180[CLO1313_RS11095], lcl|NC_017304.1_prot_WP_003517251.1_1312[CLO1313_RS06695],

		lcl|NC_017304.1_prot_WP_004463183.1_1714[CLO1313_RS08740], lcl|NC_017304.1_prot_WP_003513162.1_84[CLO1313_RS00440]
hydrolase activity, hydrolyzing O-glycosyl compounds	9	lcl|NC_017304.1_prot_WP_003513507.1_2942[CLO1313_RS14960], lcl|NC_017304.1_prot_WP_003513657.1_2857[CLO1313_RS14535], lcl|NC_017304.1_prot_WP_003519011.1_1977[CLO1313_RS10065], lcl|NC_017304.1_prot_WP_003522420.1_2566[CLO1313_RS13020], lcl|NC_017304.1_prot_WP_003516854.1_2837[CLO1313_RS14425], lcl|NC_017304.1_prot_WP_003518514.1_495[CLO1313_RS02535], lcl|NC_017304.1_prot_WP_003517828.1_2232[CLO1313_RS11345], lcl|NC_017304.1_prot_WP_003516694.1_2791[CLO1313_RS14190], lcl|NC_017304.1_prot_WP_003516835.1_2855[CLO1313_RS14525]
carbohydrate binding	9	lcl|NC_017304.1_prot_WP_003519374.1_628[CLO1313_RS03225], lcl|NC_017304.1_prot_WP_003513657.1_2857[CLO1313_RS14535], lcl|NC_017304.1_prot_WP_003519375.1_627[CLO1313_RS03220], lcl|NC_017304.1_prot_WP_003516854.1_2837[CLO1313_RS14425], lcl|NC_017304.1_prot_WP_003513660.1_2856[CLO1313_RS14530], lcl|NC_017304.1_prot_WP_003518514.1_495[CLO1313_RS02535], lcl|NC_017304.1_prot_WP_014522595.1_626[CLO1313_RS15440], lcl|NC_017304.1_prot_WP_003516694.1_2791[CLO1313_RS14190], lcl|NC_017304.1_prot_WP_003516835.1_2855[CLO1313_RS14525]
metal ion binding	8	lcl|NC_017304.1_prot_WP_003516053.1_764[CLO1313_RS03955], lcl|NC_017304.1_prot_WP_003517259.1_1308[CLO1313_RS06675], lcl|NC_017304.1_prot_WP_003519012.1_1972[CLO1313_RS10040], lcl|NC_017304.1_prot_WP_003516986.1_947[CLO1313_RS04845], lcl|NC_017304.1_prot_WP_003519027.1_1795[CLO1313_RS09145], lcl|NC_017304.1_prot_WP_003515281.1_397[CLO1313_RS02025], lcl|NC_017304.1_prot_WP_003513449.1_2977[CLO1313_RS15135], lcl|NC_017304.1_prot_WP_003516749.1_2744[CLO1313_RS13955]
phosphorelay sensor kinase activity	7	lcl|NC_017304.1_prot_WP_003518714.1_1399[CLO1313_RS07140], lcl|NC_017304.1_prot_WP_003518707.1_1403[CLO1313_RS07160], lcl|NC_017304.1_prot_WP_003518456.1_2956[CLO1313_RS15030], lcl|NC_017304.1_prot_WP_003518814.1_402[CLO1313_RS02050], lcl|NC_017304.1_prot_WP_003517496.1_988[CLO1313_RS05055], lcl|NC_017304.1_prot_WP_003517988.1_1716[CLO1313_RS08750], lcl|NC_017304.1_prot_WP_003515794.1_2499[CLO1313_RS12660]
transmembrane signaling receptor activity	7	lcl|NC_017304.1_prot_WP_080552882.1_2183[CLO1313_RS11110], lcl|NC_017304.1_prot_WP_014522631.1_2185[CLO1313_RS11120], lcl|NC_017304.1_prot_WP_003517776.1_2259[CLO1313_RS11480], lcl|NC_017304.1_prot_WP_003518813.1_403[CLO1313_RS02055], lcl|NC_017304.1_prot_WP_003516942.1_530[CLO1313_RS02725], lcl|NC_017304.1_prot_WP_003516902.1_584[CLO1313_RS02990], lcl|NC_017304.1_prot_WP_003518457.1_2955[CLO1313_RS15025]
transmembrane transporter activity	6	lcl|NC_017304.1_prot_WP_003517330.1_2350[CLO1313_RS11910], lcl|NC_017304.1_prot_WP_003515800.1_2495[CLO1313_RS12640], lcl|NC_017304.1_prot_WP_003517459.1_1005[CLO1313_RS05135], lcl|NC_017304.1_prot_WP_003513175.1_78[CLO1313_RS00410],

		lcl|NC_017304.1_prot_WP_003521788.1_2349[CLO1313_RS11905], lcl|NC_017304.1_prot_WP_003515799.1_2496[CLO1313_RS12645]
transferase activity	6	lcl|NC_017304.1_prot_WP_003516017.1_2401[CLO1313_RS12160], lcl|NC_017304.1_prot_WP_003519199.1_805[CLO1313_RS04145], lcl|NC_017304.1_prot_WP_003513187.1_73[CLO1313_RS00385], lcl|NC_017304.1_prot_WP_041740090.1_1924[CLO1313_RS09800], lcl|NC_017304.1_prot_WP_003517446.1_1012[CLO1313_RS05170], lcl|NC_017304.1_prot_WP_003517337.1_2347[CLO1313_RS11895]
DNA-binding transcription factor activity	6	lcl|NC_017304.1_prot_WP_003515795.1_2498[CLO1313_RS12655], lcl|NC_017304.1_prot_WP_003517993.1_1711[CLO1313_RS08725], lcl|NC_017304.1_prot_WP_003512374.1_1971[CLO1313_RS10035], lcl|NC_017304.1_prot_WP_003515819.1_2483[CLO1313_RS12580], lcl|NC_017304.1_prot_WP_003517500.1_984[CLO1313_RS05035], lcl|NC_017304.1_prot_WP_003513078.1_127[CLO1313_RS00670]
structural molecule activity	6	lcl|NC_017304.1_prot_WP_003518494.1_2914[CLO1313_RS14820], lcl|NC_017304.1_prot_WP_003513572.1_2913[CLO1313_RS14815], lcl|NC_017304.1_prot_WP_003518492.1_2915[CLO1313_RS14825], lcl|NC_017304.1_prot_WP_003513581.1_2908[CLO1313_RS14790], lcl|NC_017304.1_prot_WP_080547380.1_2298[CLO1313_RS15685], lcl|NC_017304.1_prot_WP_003513583.1_2907[CLO1313_RS14785]
cellulose binding	5	lcl|NC_017304.1_prot_WP_003517483.1_999[CLO1313_RS05110], lcl|NC_017304.1_prot_WP_014522588.1_90[CLO1313_RS15490], lcl|NC_017304.1_prot_WP_003518381.1_2179[CLO1313_RS11090], lcl|NC_017304.1_prot_WP_003519027.1_1795[CLO1313_RS09145], lcl|NC_017304.1_prot_WP_003515281.1_397[CLO1313_RS02025]
cellulase activity	4	lcl|NC_017304.1_prot_WP_003518381.1_2179[CLO1313_RS11090], lcl|NC_017304.1_prot_WP_003519027.1_1795[CLO1313_RS09145], lcl|NC_017304.1_prot_WP_003515281.1_397[CLO1313_RS02025], lcl|NC_017304.1_prot_WP_003516749.1_2744[CLO1313_RS13955]
sigma factor activity	4	lcl|NC_017304.1_prot_WP_003517993.1_1711[CLO1313_RS08725], lcl|NC_017304.1_prot_WP_003515819.1_2483[CLO1313_RS12580], lcl|NC_017304.1_prot_WP_003517500.1_984[CLO1313_RS05035], lcl|NC_017304.1_prot_WP_003513078.1_127[CLO1313_RS00670]
lyase activity	4	lcl|NC_017304.1_prot_WP_003516854.1_2837[CLO1313_RS14425], lcl|NC_017304.1_prot_WP_003518334.1_147[CLO1313_RS00765], lcl|NC_017304.1_prot_WP_003513590.1_2903[CLO1313_RS14765], lcl|NC_017304.1_prot_WP_003517997.1_1707[CLO1313_RS08705]
pyridoxal phosphate binding	4	lcl|NC_017304.1_prot_WP_003516678.1_1526[CLO1313_RS07770], lcl|NC_017304.1_prot_WP_003520835.1_378[CLO1313_RS01930], lcl|NC_017304.1_prot_WP_003511604.1_650[CLO1313_RS03360], lcl|NC_017304.1_prot_WP_003516296.1_1455[CLO1313_RS07420]
GTP binding	4	lcl|NC_017304.1_prot_WP_003511964.1_2180[CLO1313_RS11095], lcl|NC_017304.1_prot_WP_003520743.1_844[CLO1313_RS04340], lcl|NC_017304.1_prot_WP_014522619.1_1594[CLO1313_RS08125], lcl|NC_017304.1_prot_WP_003517984.1_1720[CLO1313_RS08770]

hydrolase activity, acting on glycosyl bonds	3	lcl|NC_017304.1_prot_WP_003517483.1_999[CLO1313_RS05110], lcl|NC_017304.1_prot_WP_003517623.1_1664[CLO1313_RS08485], lcl|NC_017304.1_prot_WP_003520682.1_2383[CLO1313_RS12070]
glutamate-ammonia ligase activity	3	lcl|NC_017304.1_prot_WP_003512274.1_2027[CLO1313_RS10315], lcl|NC_017304.1_prot_WP_003517405.1_2305[CLO1313_RS11700], lcl|NC_017304.1_prot_WP_003512284.1_2021[CLO1313_RS10285]
kinase activity	3	lcl|NC_017304.1_prot_WP_003518716.1_1398[CLO1313_RS07135], lcl|NC_017304.1_prot_WP_003518706.1_1404[CLO1313_RS07165], lcl|NC_017304.1_prot_WP_003516413.1_72[CLO1313_RS00380]
oxidoreductase activity	3	lcl|NC_017304.1_prot_WP_003520287.1_788[CLO1313_RS04065], lcl|NC_017304.1_prot_WP_003513182.1_75[CLO1313_RS00395], lcl|NC_017304.1_prot_WP_003512281.1_2023[CLO1313_RS10295]
ATPase activity	3	lcl|NC_017304.1_prot_WP_003519421.1_509[CLO1313_RS02620], lcl|NC_017304.1_prot_WP_003514730.1_508[CLO1313_RS02615], lcl|NC_017304.1_prot_WP_003516409.1_77[CLO1313_RS00405]
transaminase activity	3	lcl|NC_017304.1_prot_WP_003512275.1_2026[CLO1313_RS10310], lcl|NC_017304.1_prot_WP_003520835.1_378[CLO1313_RS01930], lcl|NC_017304.1_prot_WP_003516296.1_1455[CLO1313_RS07420]
4 iron, 4 sulfur cluster binding	3	lcl|NC_017304.1_prot_WP_003516986.1_947[CLO1313_RS04845], lcl|NC_017304.1_prot_WP_003516497.1_1614[CLO1313_RS08220], lcl|NC_017304.1_prot_WP_003517337.1_2347[CLO1313_RS11895]
methyltransferase activity	3	lcl|NC_017304.1_prot_WP_003517259.1_1308[CLO1313_RS06675], lcl|NC_017304.1_prot_WP_003513592.1_2902[CLO1313_RS14760], lcl|NC_017304.1_prot_WP_003516030.1_2390[CLO1313_RS12105]
catalytic activity	3	lcl|NC_017304.1_prot_WP_003516986.1_947[CLO1313_RS04845], lcl|NC_017304.1_prot_WP_003513028.1_144[CLO1313_RS00750], lcl|NC_017304.1_prot_WP_003513173.1_79[CLO1313_RS00415]
protein-glutamate O-methyltransferase activity	3	lcl|NC_017304.1_prot_WP_003518711.1_1401[CLO1313_RS07150], lcl|NC_017304.1_prot_WP_003514500.1_404[CLO1313_RS02060], lcl|NC_017304.1_prot_WP_003518458.1_2954[CLO1313_RS15020]
protein-glutamine glutaminase activity	3	lcl|NC_017304.1_prot_WP_003517991.1_1713[CLO1313_RS08735], lcl|NC_017304.1_prot_WP_003518709.1_1402[CLO1313_RS07155], lcl|NC_017304.1_prot_WP_003517987.1_1717[CLO1313_RS08755]
ferrous iron transmembrane transporter activity	2	lcl|NC_017304.1_prot_WP_003520743.1_844[CLO1313_RS04340], lcl|NC_017304.1_prot_WP_014522619.1_1594[CLO1313_RS08125]
urease activity	2	lcl|NC_017304.1_prot_WP_003515807.1_2491[CLO1313_RS12620], lcl|NC_017304.1_prot_WP_003515809.1_2490[CLO1313_RS12615]
protein-glutamate methylesterase activity	2	lcl|NC_017304.1_prot_WP_003518709.1_1402[CLO1313_RS07155], lcl|NC_017304.1_prot_WP_003517987.1_1717[CLO1313_RS08755]
motor activity	2	lcl|NC_017304.1_prot_WP_003517971.1_1729[CLO1313_RS08815], lcl|NC_017304.1_prot_WP_003517972.1_1728[CLO1313_RS08810]
cyclic-guanylate-specific phosphodiesterase activity	2	lcl|NC_017304.1_prot_WP_003518225.1_870[CLO1313_RS04465], lcl|NC_017304.1_prot_WP_003516246.1_1485[CLO1313_RS07570]
RNA binding	2	lcl|NC_017304.1_prot_WP_003511692.1_710[CLO1313_RS03680], lcl|NC_017304.1_prot_WP_003512286.1_2020[CLO1313_RS10280]
glutamate synthase (NADPH) activity	2	lcl|NC_017304.1_prot_WP_003512275.1_2026[CLO1313_RS10310], lcl|NC_017304.1_prot_WP_003518076.1_2025[CLO1313_RS10305]

nucleotide binding	2	lcl|NC_017304.1_prot_WP_003512660.1_1834[CLO1313_RS09335], lcl|NC_017304.1_prot_WP_003516413.1_72[CLO1313_RS00380]
ATPase-coupled ferric iron transmembrane transporter activity	2	lcl|NC_017304.1_prot_WP_003517318.1_2356[CLO1313_RS11940], lcl|NC_017304.1_prot_WP_003515803.1_2493[CLO1313_RS12630]
N-acetylmuramoyl-L-alanine amidase activity	2	lcl|NC_017304.1_prot_WP_003515588.1_1192[CLO1313_RS06095], lcl|NC_017304.1_prot_WP_003515313.1_367[CLO1313_RS01880]
nickel cation binding	2	lcl|NC_017304.1_prot_WP_003515807.1_2491[CLO1313_RS12620], lcl|NC_017304.1_prot_WP_003515809.1_2490[CLO1313_RS12615]
phosphorelay response regulator activity	2	lcl|NC_017304.1_prot_WP_003518709.1_1402[CLO1313_RS07155], lcl|NC_017304.1_prot_WP_003517987.1_1717[CLO1313_RS08755]
coenzyme binding	1	lcl|NC_017304.1_prot_WP_003513028.1_144[CLO1313_RS00750]
scopolin beta-glucosidase activity	1	lcl|NC_017304.1_prot_WP_003517480.1_1000[CLO1313_RS05115]
cysteine-type peptidase activity	1	lcl|NC_017304.1_prot_WP_003517571.1_1688[CLO1313_RS08605]
oxidoreductase activity, acting on the CH-NH2 group of donors, NAD or NADP as acceptor	1	lcl|NC_017304.1_prot_WP_003512660.1_1834[CLO1313_RS09335]
ATPase-coupled glycerol-3-phosphate transmembrane transporter activity	1	lcl|NC_017304.1_prot_WP_003517318.1_2356[CLO1313_RS11940]
2 iron, 2 sulfur cluster binding	1	lcl|NC_017304.1_prot_WP_003520287.1_788[CLO1313_RS04065]
glucose-1-phosphate cytidylyltransferase activity	1	lcl|NC_017304.1_prot_WP_003513023.1_148[CLO1313_RS00770]
DNA-directed 5'-3' RNA polymerase activity	1	lcl|NC_017304.1_prot_WP_003517993.1_1711[CLO1313_RS08725]
gamma-glutamylaminecyclotransferase activity	1	lcl|NC_017304.1_prot_WP_003517756.1_2269[CLO1313_RS11525]
ATPase-coupled polyamine transmembrane transporter activity	1	lcl|NC_017304.1_prot_WP_003517318.1_2356[CLO1313_RS11940]
enzyme regulator activity	1	lcl|NC_017304.1_prot_WP_003517774.1_2260[CLO1313_RS11485]
protein disulfide oxidoreductase activity	1	lcl|NC_017304.1_prot_WP_003512363.1_1981[CLO1313_RS10085]
zinc ion binding	1	lcl|NC_017304.1_prot_WP_003513182.1_75[CLO1313_RS00395]
bacteriocin transmembrane transporter activity	1	lcl|NC_017304.1_prot_WP_003517571.1_1688[CLO1313_RS08605]
diaminopimelate epimerase activity	1	lcl|NC_017304.1_prot_WP_003511603.1_649[CLO1313_RS03355]
cadmium transmembrane transporter activity, phosphorylative mechanism	1	lcl|NC_017304.1_prot_WP_003519012.1_1972[CLO1313_RS10040]
iron-sulfur cluster binding	1	lcl|NC_017304.1_prot_WP_003516053.1_764[CLO1313_RS03955]

decanoate-CoA ligase activity	1	lcl|NC_017304.1_prot_WP_003520602.1_1982[CLO1313_RS10090]
cyclic-di-GMP binding	1	lcl|NC_017304.1_prot_WP_003517986.1_1718[CLO1313_RS08760]
phosphoribosylformylglycinamidine synthase activity	1	lcl|NC_017304.1_prot_WP_003517617.1_1667[CLO1313_RS08500]
ATPase-coupled monosaccharide transmembrane transporter activity	1	lcl|NC_017304.1_prot_WP_003517334.1_2348[CLO1313_RS11900]
sequence-specific DNA binding	1	lcl|NC_017304.1_prot_WP_003515795.1_2498[CLO1313_RS12655]
ATPase-coupled transmembrane transporter activity	1	lcl|NC_017304.1_prot_WP_003517571.1_1688[CLO1313_RS08605]
S-adenosylmethionine-dependent methyltransferase activity	1	lcl|NC_017304.1_prot_WP_003511692.1_710[CLO1313_RS03680]
N-methyltransferase activity	1	lcl|NC_017304.1_prot_WP_003517294.1_2368[CLO1313_RS11995]
alpha-L-arabinofuranosidase activity	1	lcl|NC_017304.1_prot_WP_003513660.1_2856[CLO1313_RS14530]
protein kinase activity	1	lcl|NC_017304.1_prot_WP_003518726.1_1392[CLO1313_RS07105]
endonuclease activity	1	lcl|NC_017304.1_prot_WP_003516020.1_2399[CLO1313_RS12150]
ammonium transmembrane transporter activity	1	lcl|NC_017304.1_prot_WP_003517774.1_2260[CLO1313_RS11485]
lytic transglycosylase activity	1	lcl|NC_017304.1_prot_WP_003514333.1_333[CLO1313_RS01715]
[formate-C-acetyltransferase]-activating enzyme activity	1	lcl|NC_017304.1_prot_WP_003517337.1_2347[CLO1313_RS11895]
NAD+ synthase (glutamine-hydrolyzing) activity	1	lcl|NC_017304.1_prot_WP_003520327.1_1888[CLO1313_RS09620]
exonuclease activity	1	lcl|NC_017304.1_prot_WP_003516053.1_764[CLO1313_RS03955]
protein binding	1	lcl|NC_017304.1_prot_WP_003517571.1_1688[CLO1313_RS08605]
phosphotransferase activity, alcohol group as acceptor	1	lcl|NC_017304.1_prot_WP_003516413.1_72[CLO1313_RS00380]
sulfurtransferase activity	1	lcl|NC_017304.1_prot_WP_003516495.1_1615[CLO1313_RS08225]
2-isopropylmalate synthase activity	1	lcl|NC_017304.1_prot_WP_003518251.1_856[CLO1313_RS04395]
valine-tRNA ligase activity	1	lcl|NC_017304.1_prot_WP_003519337.1_1889[CLO1313_RS09625]
double-stranded DNA binding	1	lcl|NC_017304.1_prot_WP_003513382.1_3010[CLO1313_RS15305]
cellulose 1,4-beta-cellobiosidase activity	1	lcl|NC_017304.1_prot_WP_003519027.1_1795[CLO1313_RS09145]
aspartyl esterase activity	1	lcl|NC_017304.1_prot_WP_003518514.1_495[CLO1313_RS02535]
nucleotidyltransferase activity	1	lcl|NC_017304.1_prot_WP_003516498.1_1613[CLO1313_RS08215]
glutaminyl-tRNA synthase (glutamine-hydrolyzing) activity	1	lcl|NC_017304.1_prot_WP_003516017.1_2401[CLO1313_RS12160]

cellulose 1,4-beta-cellobiosidase activity (reducing end)	1	lcl|NC_017304.1_prot_WP_003516749.1_2744[CLO1313_RS13955]
transporter activity	1	lcl|NC_017304.1_prot_WP_003514775.1_528[CLO1313_RS02715]
diaminopimelate decarboxylase activity	1	lcl|NC_017304.1_prot_WP_003516678.1_1526[CLO1313_RS07770]
NAD+ synthase activity	1	lcl|NC_017304.1_prot_WP_003520327.1_1888[CLO1313_RS09620]
serine-type endopeptidase activity	1	lcl|NC_017304.1_prot_WP_003512399.1_1956[CLO1313_RS09960]
hydrolase activity, acting on carbon-nitrogen (but not peptide) bonds	1	lcl|NC_017304.1_prot_WP_003517623.1_1664[CLO1313_RS08485]
peptidase activity	1	lcl|NC_017304.1_prot_WP_003513147.1_91[CLO1313_RS00490]
transition metal ion binding	1	lcl|NC_017304.1_prot_WP_003520966.1_1593[CLO1313_RS08120]
L,L-diaminopimelate aminotransferase activity	1	lcl|NC_017304.1_prot_WP_003511604.1_650[CLO1313_RS03360]
ubiquitin-like modifier activating enzyme activity	1	lcl|NC_017304.1_prot_WP_003516498.1_1613[CLO1313_RS08215]
ligase activity	1	lcl|NC_017304.1_prot_WP_003517625.1_1663[CLO1313_RS08480]
ATPase-coupled organic phosphonate transmembrane transporter activity	1	lcl|NC_017304.1_prot_WP_003515802.1_2494[CLO1313_RS12635]
3-deoxy-manno-octulosonate cytidylyltransferase activity	1	lcl|NC_017304.1_prot_WP_003513594.1_2901[CLO1313_RS14755]
3-methyl-2-oxobutanoate hydroxymethyltransferase activity	1	lcl|NC_017304.1_prot_WP_003517259.1_1308[CLO1313_RS06675]
pectinesterase activity	1	lcl|NC_017304.1_prot_WP_003518514.1_495[CLO1313_RS02535]
asparagine synthase (glutamine-hydrolyzing) activity	1	lcl|NC_017304.1_prot_WP_003517621.1_1665[CLO1313_RS08490]
protein dimerization activity	1	lcl|NC_017304.1_prot_WP_003517496.1_988[CLO1313_RS05055]
beta-glucosidase activity	1	lcl|NC_017304.1_prot_WP_003517480.1_1000[CLO1313_RS05115]
long-chain fatty acid-CoA ligase activity	1	lcl|NC_017304.1_prot_WP_003520602.1_1982[CLO1313_RS10090]
iron ion binding	1	lcl|NC_017304.1_prot_WP_003516497.1_1614[CLO1313_RS08220]
electron transfer activity	1	lcl|NC_017304.1_prot_WP_003512363.1_1981[CLO1313_RS10085]
gamma-glutamylcyclotransferase activity	1	lcl|NC_017304.1_prot_WP_003516017.1_2401[CLO1313_RS12160]
2-iminoacetate synthase activity	1	lcl|NC_017304.1_prot_WP_003516497.1_1614[CLO1313_RS08220]
GTPase activity	1	lcl|NC_017304.1_prot_WP_003517984.1_1720[CLO1313_RS08770]

transferase activity, transferring aldehyde or ketonic groups	1	lcl|NC_017304.1_prot_WP_003513185.1_74[CLO1313_RS00390]
aminoacyl-tRNA editing activity	1	lcl|NC_017304.1_prot_WP_003519337.1_1889[CLO1313_RS09625]
flavin adenine dinucleotide binding	1	lcl|NC_017304.1_prot_WP_003512281.1_2023[CLO1313_RS10295]

Supplemental Table S9. Protein sequences for each up-DEGs classified as cellular component.

GO	Number of Seqs	Proteins for Gene (nucleotide) Sequences involved in GO
integral component of membrane	59	lcl|NC_017304.1_prot_WP_003512275.1_2026[CLO1313_RS10310], lcl|NC_017304.1_prot_WP_003517459.1_1005[CLO1313_RS05135], lcl|NC_017304.1_prot_WP_003517992.1_1712[CLO1313_RS08730], lcl|NC_017304.1_prot_WP_014522651.1_2668[CLO1313_RS13550], lcl|NC_017304.1_prot_WP_003517496.1_988[CLO1313_RS05055], lcl|NC_017304.1_prot_WP_003512486.1_1913[CLO1313_RS09745], lcl|NC_017304.1_prot_WP_003517997.1_1707[CLO1313_RS08705], lcl|NC_017304.1_prot_WP_003515794.1_2499[CLO1313_RS12660], lcl|NC_017304.1_prot_WP_037294696.1_510[CLO1313_RS02625], lcl|NC_017304.1_prot_WP_003519012.1_1972[CLO1313_RS10040], lcl|NC_017304.1_prot_WP_003516942.1_530[CLO1313_RS02725], lcl|NC_017304.1_prot_WP_003513175.1_78[CLO1313_RS00410], lcl|NC_017304.1_prot_WP_003521788.1_2349[CLO1313_RS11905], lcl|NC_017304.1_prot_WP_003520743.1_844[CLO1313_RS04340], lcl|NC_017304.1_prot_WP_003517819.1_2237[CLO1313_RS11370], lcl|NC_017304.1_prot_WP_014522653.1_2674[CLO1313_RS13580], lcl|NC_017304.1_prot_WP_003516356.1_1428[CLO1313_RS07285], lcl|NC_017304.1_prot_WP_003517957.1_1736[CLO1313_RS08850], lcl|NC_017304.1_prot_WP_014522631.1_2185[CLO1313_RS11120], lcl|NC_017304.1_prot_WP_014522588.1_90[CLO1313_RS15490], lcl|NC_017304.1_prot_WP_003515066.1_2815[CLO1313_RS14310], lcl|NC_017304.1_prot_WP_003520978.1_1534[CLO1313_RS07810], lcl|NC_017304.1_prot_WP_003517976.1_1726[CLO1313_RS08800], lcl|NC_017304.1_prot_WP_003514775.1_528[CLO1313_RS02715], lcl|NC_017304.1_prot_WP_003516418.1_57[CLO1313_RS00305], lcl|NC_017304.1_prot_WP_003514774.1_527[CLO1313_RS02710], lcl|NC_017304.1_prot_WP_003512488.1_1912[CLO1313_RS09740], lcl|NC_017304.1_prot_WP_003512939.1_204[CLO1313_RS01050], lcl|NC_017304.1_prot_WP_003511605.1_651[CLO1313_RS03365], lcl|NC_017304.1_prot_WP_003516902.1_584[CLO1313_RS02990], lcl|NC_017304.1_prot_WP_003519188.1_812[CLO1313_RS04180], lcl|NC_017304.1_prot_WP_003515799.1_2496[CLO1313_RS12645], lcl|NC_017304.1_prot_WP_003517996.1_1708[CLO1313_RS08710], lcl|NC_017304.1_prot_WP_003513222.1_56[CLO1313_RS00300], lcl|NC_017304.1_prot_WP_003513833.1_2567[CLO1313_RS13025], lcl|NC_017304.1_prot_WP_003512303.1_2010[CLO1313_RS10230], lcl|NC_017304.1_prot_WP_003517979.1_1724[CLO1313_RS08790], lcl|NC_017304.1_prot_WP_003515820.1_2482[CLO1313_RS12575], lcl|NC_017304.1_prot_WP_080552882.1_2183[CLO1313_RS11110], lcl|NC_017304.1_prot_WP_003517320.1_2355[CLO1313_RS11935], lcl|NC_017304.1_prot_WP_014522619.1_1594[CLO1313_RS08125], lcl|NC_017304.1_prot_WP_003517571.1_1688[CLO1313_RS08605], lcl|NC_017304.1_prot_WP_003517982.1_1722[CLO1313_RS08780], lcl|NC_017304.1_prot_WP_003517483.1_999[CLO1313_RS05110],

		lcl|NC_017304.1_prot_WP_003515800.1_2495[CLO1313_RS12640], lcl|NC_017304.1_prot_WP_003517776.1_2259[CLO1313_RS11480], lcl|NC_017304.1_prot_WP_003517623.1_1664[CLO1313_RS08485], lcl|NC_017304.1_prot_WP_003517774.1_2260[CLO1313_RS11485], lcl|NC_017304.1_prot_WP_003517416.1_2300[CLO1313_RS11675], lcl|NC_017304.1_prot_WP_003517828.1_2232[CLO1313_RS11345], lcl|NC_017304.1_prot_WP_003517969.1_1730[CLO1313_RS08820], lcl|NC_017304.1_prot_WP_003517330.1_2350[CLO1313_RS11910], lcl|NC_017304.1_prot_WP_003512399.1_1956[CLO1313_RS09960], lcl|NC_017304.1_prot_WP_003518381.1_2179[CLO1313_RS11090], lcl|NC_017304.1_prot_WP_003517983.1_1721[CLO1313_RS08775], lcl|NC_017304.1_prot_WP_003519116.1_1021[CLO1313_RS05215], lcl|NC_017304.1_prot_WP_003517823.1_2235[CLO1313_RS11360], lcl|NC_017304.1_prot_WP_003512624.1_1854[CLO1313_RS09435], lcl|NC_017304.1_prot_WP_003518355.1_126[CLO1313_RS00665]
plasma membrane	18	lcl|NC_017304.1_prot_WP_014522631.1_2185[CLO1313_RS11120], lcl|NC_017304.1_prot_WP_003515800.1_2495[CLO1313_RS12640], lcl|NC_017304.1_prot_WP_003517979.1_1724[CLO1313_RS08790], lcl|NC_017304.1_prot_WP_003517969.1_1730[CLO1313_RS08820], lcl|NC_017304.1_prot_WP_037294696.1_510[CLO1313_RS02625], lcl|NC_017304.1_prot_WP_003519012.1_1972[CLO1313_RS10040], lcl|NC_017304.1_prot_WP_080552882.1_2183[CLO1313_RS11110], lcl|NC_017304.1_prot_WP_003517330.1_2350[CLO1313_RS11910], lcl|NC_017304.1_prot_WP_003517320.1_2355[CLO1313_RS11935], lcl|NC_017304.1_prot_WP_003512399.1_1956[CLO1313_RS09960], lcl|NC_017304.1_prot_WP_003513175.1_78[CLO1313_RS00410], lcl|NC_017304.1_prot_WP_003517341.1_2345[CLO1313_RS11885], lcl|NC_017304.1_prot_WP_003521788.1_2349[CLO1313_RS11905], lcl|NC_017304.1_prot_WP_003517983.1_1721[CLO1313_RS08775], lcl|NC_017304.1_prot_WP_003520743.1_844[CLO1313_RS04340], lcl|NC_017304.1_prot_WP_014522619.1_1594[CLO1313_RS08125], lcl|NC_017304.1_prot_WP_003517982.1_1722[CLO1313_RS08780], lcl|NC_017304.1_prot_WP_003515799.1_2496[CLO1313_RS12645]
cytoplasm	14	lcl|NC_017304.1_prot_WP_003511692.1_710[CLO1313_RS03680], lcl|NC_017304.1_prot_WP_003518714.1_1399[CLO1313_RS07140], lcl|NC_017304.1_prot_WP_003516495.1_1615[CLO1313_RS08225], lcl|NC_017304.1_prot_WP_003519337.1_1889[CLO1313_RS09625], lcl|NC_017304.1_prot_WP_003518456.1_2956[CLO1313_RS15030], lcl|NC_017304.1_prot_WP_003518709.1_1402[CLO1313_RS07155], lcl|NC_017304.1_prot_WP_003517988.1_1716[CLO1313_RS08750], lcl|NC_017304.1_prot_WP_003511603.1_649[CLO1313_RS03355], lcl|NC_017304.1_prot_WP_003517259.1_1308[CLO1313_RS06675], lcl|NC_017304.1_prot_WP_003515807.1_2491[CLO1313_RS12620], lcl|NC_017304.1_prot_WP_003518814.1_402[CLO1313_RS02050], lcl|NC_017304.1_prot_WP_003515809.1_2490[CLO1313_RS12615], lcl|NC_017304.1_prot_WP_003517500.1_984[CLO1313_RS05035], lcl|NC_017304.1_prot_WP_003517987.1_1717[CLO1313_RS08755]

extracellular region	9	lcl|NC_017304.1_prot_WP_003518494.1_2914[CLO1313_RS14820], lcl|NC_017304.1_prot_WP_003519374.1_628[CLO1313_RS03225], lcl|NC_017304.1_prot_WP_003519375.1_627[CLO1313_RS03220], lcl|NC_017304.1_prot_WP_003516854.1_2837[CLO1313_RS14425], lcl|NC_017304.1_prot_WP_003518492.1_2915[CLO1313_RS14825], lcl|NC_017304.1_prot_WP_014522595.1_626[CLO1313_RS15440], lcl|NC_017304.1_prot_WP_003513581.1_2908[CLO1313_RS14790], lcl|NC_017304.1_prot_WP_003516749.1_2744[CLO1313_RS13955], lcl|NC_017304.1_prot_WP_003513583.1_2907[CLO1313_RS14785]
bacterial-type flagellum basal body	6	lcl|NC_017304.1_prot_WP_003517971.1_1729[CLO1313_RS08815], lcl|NC_017304.1_prot_WP_003517972.1_1728[CLO1313_RS08810], lcl|NC_017304.1_prot_WP_003512932.1_207[CLO1313_RS01065], lcl|NC_017304.1_prot_WP_003517965.1_1732[CLO1313_RS08830], lcl|NC_017304.1_prot_WP_003517979.1_1724[CLO1313_RS08790], lcl|NC_017304.1_prot_WP_003517969.1_1730[CLO1313_RS08820]
membrane	5	lcl|NC_017304.1_prot_WP_003518813.1_403[CLO1313_RS02055], lcl|NC_017304.1_prot_WP_003517972.1_1728[CLO1313_RS08810], lcl|NC_017304.1_prot_WP_003514333.1_333[CLO1313_RS01715], lcl|NC_017304.1_prot_WP_003518457.1_2955[CLO1313_RS15025], lcl|NC_017304.1_prot_WP_003520287.1_788[CLO1313_RS04065]
bacterial-type flagellum hook	3	lcl|NC_017304.1_prot_WP_003518494.1_2914[CLO1313_RS14820], lcl|NC_017304.1_prot_WP_003513572.1_2913[CLO1313_RS14815], lcl|NC_017304.1_prot_WP_003518492.1_2915[CLO1313_RS14825]
bacterial-type flagellum filament	3	lcl|NC_017304.1_prot_WP_003513572.1_2913[CLO1313_RS14815], lcl|NC_017304.1_prot_WP_003513581.1_2908[CLO1313_RS14790], lcl|NC_017304.1_prot_WP_003513583.1_2907[CLO1313_RS14785]
S-layer	3	lcl|NC_017304.1_prot_WP_003519374.1_628[CLO1313_RS03225], lcl|NC_017304.1_prot_WP_003519375.1_627[CLO1313_RS03220], lcl|NC_017304.1_prot_WP_014522595.1_626[CLO1313_RS15440]
cell wall	3	lcl|NC_017304.1_prot_WP_003519374.1_628[CLO1313_RS03225], lcl|NC_017304.1_prot_WP_003519375.1_627[CLO1313_RS03220], lcl|NC_017304.1_prot_WP_014522595.1_626[CLO1313_RS15440]
ATP-binding cassette (ABC) transporter complex	2	lcl|NC_017304.1_prot_WP_003517318.1_2356[CLO1313_RS11940], lcl|NC_017304.1_prot_WP_003514728.1_507[CLO1313_RS02610]
bacterial-type flagellum	1	lcl|NC_017304.1_prot_WP_080547380.1_2298[CLO1313_RS15685]
cytosol	1	lcl|NC_017304.1_prot_WP_003513620.1_2888[CLO1313_RS14690]
cell	1	lcl|NC_017304.1_prot_WP_003512363.1_1981[CLO1313_RS10085]
bacterial-type flagellum basal body, distal rod	1	lcl|NC_017304.1_prot_WP_003512930.1_208[CLO1313_RS01070]
extracellular space	1	lcl|NC_017304.1_prot_WP_003519027.1_1795[CLO1313_RS09145]
cell outer membrane	1	lcl|NC_017304.1_prot_WP_003516418.1_57[CLO1313_RS00305]

Supplemental Table S10. Glycoside hydrolases-RsgI

9 RsgI-like proteins in ATCC 27405	DSM1313 locus - New	DSM1313 locus-Old	Log2FC	Notes	Reference
Cthe_0059	CLO1313_RS11010	clo1313_2173	0.03	GH10	Kahel-Raifer et al
Cthe_0267	CLO1313_RS09925	clo1313_1962	0.69	GH10	Kahel-Raifer et al
Cthe_0404	CLO1313_RS09190	clo1313_1817	0.42	GH10	Kahel-Raifer et al
Cthe_0260	CLO1313_RS09960	clo1313_1969	3.16	GH10; petidase SI	Kahel-Raifer et al
Cthe_0316	CLO1313_RS09665	clo1313_1910	-0.05	GH10	Kahel-Raifer et al
Cthe_1273	CLO1313_RS05030	clo1313_0985	0.93	RsgI6-GH10; alpha-L-arabinofuranosidase; contains a putative arabinose-binding domain	Kahel-Raifer et al
Cthe_2119	CLO1313_RS14110	clo1313_2777	0.29	GH10	Kahel-Raifer et al
Cthe_2522	CLO1313_RS00560	clo1313_0105	0.14	GH10	Kahel-Raifer et al
Cthe_2974	CLO1313_RS02680	clo1313_0524	0.42	GH10	Kahel-Raifer et al
**Related to Rsi24C**	**DSM1313 locus - New**	**DSM1313 locus-old**	**Log2FC**	**Notes**	**Reference**
Cthe_1471	CLO1313_RS11345	clo1313_2233	1.01	GH5; Rsi24C-GH5	Bahari et al

Supplemental Table S11. Redox relevant enzymes potentially mediated by a ferredoxin

Gene Product	DSM1313 locus - Old	DSM1313 locus - New	Log2FC
NfnA	clo1313_1848	CLO1313_RS09340	0.16
NfnB	clo1313_1849	CLO1313_RS09345	not found
RnfA-F	clo1313_0061	CLO1313_RS00325	-0.516
	clo1313_0062	CLO1313_RS00330	-0.31
	clo1313_0063	CLO1313_RS00335	-0.256
	clo1313_0064	CLO1313_RS00340	-0.162
	clo1313_0065	CLO1313_RS00345	-0.167
	clo1313_0066	CLO1313_RS00350	-0.179
PFOR1	clo1313_0020	CLO1313_RS00115	-2.161
	clo1313_0021	CLO1313_RS00120	-2.225
	clo1313_0022	CLO1313_RS00125	not found
	clo1313_0023	CLO1313_RS00130	-2.127
PFOR2	clo1313_1353	CLO1313_RS06855	0.184
	clo1313_1354	CLO1313_RS06860	0.195
	clo1313_1355	CLO1313_RS06865	0.27
	clo1313_1356	CLO1313_RS06870	0.367
PFOR3	clo1313_1615	CLO1313_RS08145	not found
	clo1313_1616	CLO1313_RS08150	not found
PFOR4	clo1313_0673	CLO1313_RS3485	not found
PFOR5	clo1313_0382	CLO1313_RS01935	0.676
	clo1313_0383	CLO1313_RS01940	0.0575
	clo1313_0384	CLO1313_RS01945	not found
	clo1313_0385	CLO1313_RS01950	-2.098
FeFe Hydrogenase (Hyd1) operon	clo1313_1881	CLO1313_RS09510	-1.142
(putative tetrameric hydrogenase)	clo1313_1882	CLO1313_RS09515	-1.2
	clo1313_1883	CLO1313_RS09520	-1.208
	clo1313_1884	CLO1313_RS09525	-1.08
	clo1313_1885	CLO1313_RS09530	-0.946
FeFe Hydrogenase (Hyd2) operon	clo1313_1790	CLO1313_RS09055	-0.889
(putative trimeric hydrognease)	clo1313_1791	CLO1313_RS09060	-0.863
	clo1313_1792	CLO1313_RS09065	-0.7
	clo1313_1793	CLO1313_RS09070	-0.519
	clo1313_1794	CLO1313_RS09075	-0.199

	clo1313_1795	CLO1313_RS09080	-0.289
FeFe Hydrogenase (Hyd3) operon	clo1313_0554	CLO1313_RS02840	0.123
(putative dimeric hydrognease)	clo1313_0555	CLO1313_RS02845	0.825
Hydrogenase expression/formation protein HypE	clo1313_0564	CLO1313_RS02885	not found
Hydrogenase expression/formation protein HypD	clo1313_0565	CLO1313_RS02890	-0.016
Hydrogenase assembly chaperone hypC/hupF	clo1313_0566	CLO1313_RS02895	not found
(NiFe) Hydrogenase maturation protein HypF	clo1313_0567	CLO1313_RS02900	not found
Hydrogenase accessory protein HypB	clo1313_0568	CLO1313_RS02905	not found
Hydrogenase expression/synthesis HypA	clo1313_0569	CLO1313_RS02910	not found
4Fe-4S Ferredoxin iron-sulfur binding domain-containing protein	clo1313_0570	CLO1313_RS02915	-0.757
Fd1	clo1313_0528	CLO1313_RS02705	7.664
Fd2	clo1313_1353	CLO1313_RS06855	0.184
Fd3	clo1313_2761	CLO1313_RS14030	-0.167
Fd4	clo1313_0375	CLO1313_RS01900	not found
Fd5	clo1313_0570	CLO1313_RS15535	not found
Fd6	clo1313_2067	CLO1313_RS10470	not found
Fd7	clo1313_2310	CLO1313_RS11735	0.309
polyFd	clo1313_0517	CLO1313_RS02645	-0.022
4Fe-4S Ferredoxin, in the same operon as glutamate synthase	clo1313_2034	CLO1313_RS10300	4.253
		CLO1313_RS10280-310	
transcriptional regulator	clo1313_2030	CLO1313_RS10280	1.028
	clo1313_2031	CLO1313_RS10285	not found
glutamate synthase	clo1313_2032	CLO1313_RS10290	0.650
pyridine nucleotide-disulfide oxidoreductase	clo1313_2033	CLO1313_RS10295	0.818
4Fe-4S ferredoxin	clo1313_2034	CLO1313_RS10300	4.253
glutamate synthase	clo1313_2035	CLO1313_RS10305	4.097
hypothetical protein	clo1313_2036	CLO1313_RS10310	4.133
glutamine synthetase	clo1313_2037	CLO1313_RS10315	1.624
